# Synthesis and Enzymatic Evaluation of a Small Library of Substituted Phenylsulfonamido-Alkyl Sulfamates towards Carbonic Anhydrase II

**DOI:** 10.3390/molecules29133015

**Published:** 2024-06-25

**Authors:** Toni C. Denner, Niels V. Heise, Ahmed Al-Harrasi, René Csuk

**Affiliations:** 1 Organic Chemistry, Martin-Luther University Halle-Wittenberg, Kurt-Mothes, Str. 2, D-06120 Halle (Saale), Germanyniels.heise@chemie.uni-halle.de (N.V.H.); 2 Natural & Medical Sciences Research Center, University of Nizwa, Nizwa 616, Oman; aharrasi@unizwa.edu.om

**Keywords:** sulfamates, carbonic anhydrase II, inhibitor

## Abstract

A small library of 79 substituted phenylsulfonamidoalkyl sulfamates, **1b**–**79b**, was synthesized starting from arylsulfonyl chlorides and amino alcohols with different numbers of methylene groups between the hydroxyl and amino moieties yielding intermediates **1a**–**79a**, followed by the reaction of the latter with sulfamoyl chloride. All compounds were screened for their inhibitory activity on bovine carbonic anhydrase II. Compounds **1a**–**79a** showed no inhibition of the enzyme, in contrast to sulfamates **1b**–**79b**. Thus, the inhibitory potential of compounds **1b**–**79b** towards this enzyme depends on the substituent and the substitution pattern of the phenyl group as well as the length of the spacer. Bulkier substituents in the *para* position proved to be better for inhibiting CAII than compounds with the same substituent in the *meta* or *ortho* position. For many substitution patterns, compounds with shorter spacer lengths were superior to those with long chain spacers. Compounds with shorter spacer lengths performed better than those with longer chain spacers for a variety of substitution patterns. The most active compound held inhibition constant as low as K_i_ = 0.67 μM (for **49b**) and a *tert*-butyl substituent in *para* position and acted as a competitive inhibitor of the enzyme.

## 1. Introduction

The scientific interest in enzymes from the family of carbonic anhydrases has increased significantly in recent years, as it has been recognized that these enzymes are involved in a large number of metabolic reactions in a wide variety of organisms. Recently, inhibitors of the enzyme CA II have again become the focus of scientific interest [1,2,3,4,5,6,7,8,9,10,11,12,13,14].

**Figure 1:**
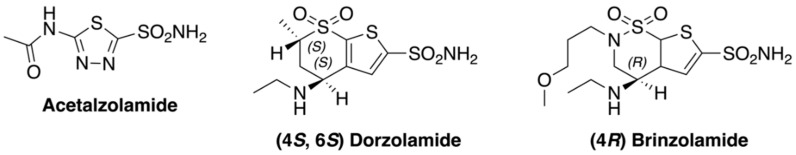
Structures of established CA inhibitors acetazolamide, dorzolamide, and brinzolamide.

Zinc-containing protein CA II (among other CAs) is involved in acid–base homeostasis [15,16,17,18,19,20,21,22,23,24], gluconeogenesis [22,23,24], lipogenesis [25,26,27,28,29,30,31], osteoclast development [32,33,34,35,36,37], and consequently in calcification. Several studies have revealed that CA II promotes the synthesis of calcium carbonate, and—as a result—the addition of a CA inhibitor. For example, acetazolamide (Figure 1) resulted in a notable reduction in calcium carbonate. This indicates that the synthesis of calcium carbonate depends on CA isoenzymes, which are acetazolamide-sensitive. Proton generation in osteoclasts is facilitated by CA II, too. This causes the resorption lacunae to become acidified and eventually dissolve the bone. Additionally, compared to normal artery tissue from the same person, human atheromatous plaques were shown to overexpress CA II. As a consequence, CA II seems highly interesting in future therapies for osteoporosis and atherosclerosis [11].

Furthermore, out of all the organs studied, the mammalian central nervous system (CNS) possesses the greatest number of CA isoforms (at least 9). Isoforms I, VB, VII, VIII, X, XI, XII, and XIV are also found, with *h*CA II being the most prevalent. Carbonic anhydrase inhibitors have been used therapeutically in a number of brain pathologies because of the broad expression range of CA isoforms in the brain. In epilepsy and idiopathic intracranial hypertension, where acetazolamide [38,39,40,41] is one of the medications now in clinical use, inhibition by CAIs has been shown to be clinically beneficial. Moreover, migraine, neuropathic pain, diabetes-induced blood–brain barrier failure, and amyloid β-induced mitochondrial dysfunction characteristic of Alzheimer’s disease are possible therapeutic uses of CAIs targeting CNS isoforms.

However, the treatment of primary open-angle glaucoma (POAG), a multifactorial optic neuropathy linked to progressive retinal ganglion cell death and visual field loss, is one of the primary uses of CA II inhibitors [42,43,44,45,46,47]. Elevated intraocular pressure is the primary risk factor for POAG, although there are numerous other related variables. Because of the unsatisfactory side effect profile of oral carbonic anhydrase inhibitors, topical CAIs took a long time to develop but have been shown to be a valuable adjunct to the treatment of primary open-angle glaucoma. They work by preventing the ciliary epithelium’s CA II from functioning. As a result, fewer bicarbonate ions are formed, which decreases intraocular pressure and fluid transfer. Inhibitor drugs such as dorzolamide or brinzolamide (Figure 1) have been in use for many years [48,49,50,51,52,53].

Furthermore, the treatment of cerebral oedema has once again become the focus of scientific interest, since brain swelling is a known side effect of some of the new drugs (e.g., lecanemab [54,55,56,57,58,59,60] and donanemab [61,62,63,64]) recently approved for the treatment of Alzheimer’s disease. Treatment of cerebral edema could include intravenous injection of a CA inhibitor.

## 2. Results

During the last few decades, numerous sulfamates have been described as inhibitors of CAs because the sulfamate group is ideally suited to interact with the central atom zinc in the active site of the enzyme [65,66,67,68,69,70,71,72]. Sulfamates are bio-isosteres to sulfonamides; the latter constitute one of the main classes of CA inhibitors. For several of them, very low inhibition constants in the range 3.1–4.8 nm have been reported [73].

**Figure 2:**
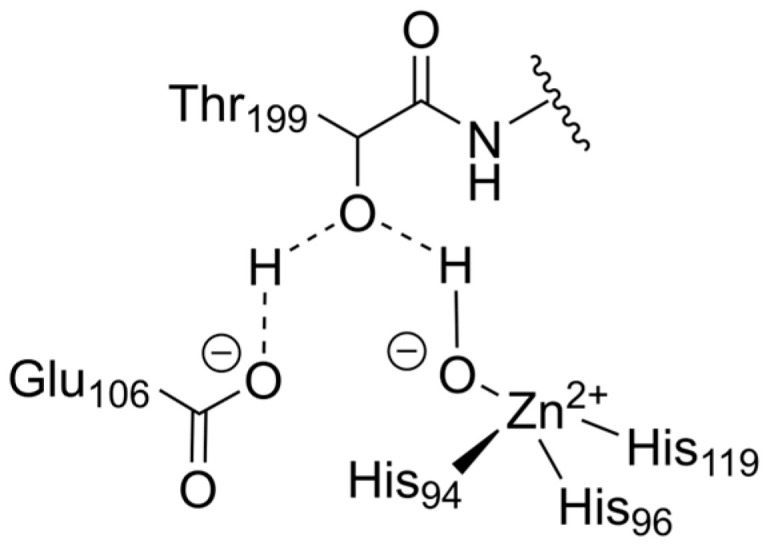
Zn(II) ion coordination in the CA II active site.

Figure 2 depicts the Zn(II) ion coordination in the CA II active site by three histidine ligands (His94, His96, and His119) and gate-keeping residues (Thr199, Glu106) [73].

**Scheme 1:**
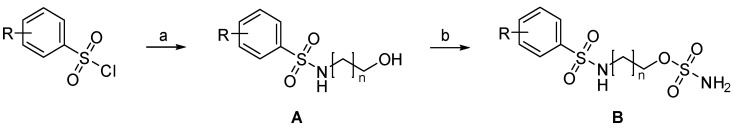
Synthesis of target sulfamates (structure **B**) from arylsulfonylchlorides via precursors **A**; (a) CH_2_Cl_2_, NEt_3_, 20 °C 3–24 h; (b) CH_2_Cl_2_, NEt_3_, sulfamoyl chloride, 0 °C → 20 °C, 3–24 h.

Evaluation of literature data on the potential of sulfamates but also our own studies showed, to our surprise, that compounds of structure **B** (Scheme 1) have not been investigated as CA inhibitors so far [65,66,67,68,69,70,71]. Very recently, we could present results showing that similar compounds derived from enantiomerically pure amino-alcohols might be efficient inhibitors of CA II [72].

**Figure 3:**
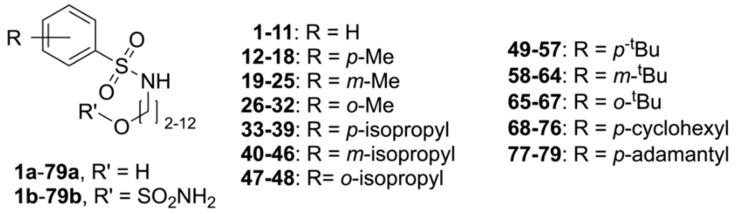
Structure of compounds **1a**–**79a** and **79b**–**79b**.

Target structure **B** can be easily synthesized by the reaction of 1,ω-aminoalcohols with arylsulfonyl chlorides, Furthermore, many arylsulfonyl chlorides are commercially available. This makes the synthesis of a small library of compounds immensely easier. Their reaction with corresponding amino alcohols led to type **A** compounds (Scheme 1), **1a**–**79a**. The compounds **1a**–**79a** (Figure 3) were reacted with sulfamoyl chloride and triethylamine in dichloromethane, resulting in the desired target compounds of type **B**, **1b**–**79b**. The latter compounds resemble a small library of substituted phenylsulfonamido alkyl sulfamates; they differ in the nature and position of substituents on the aromatic ring (*ortho*, *meta*, and *para*, as well as substituents: hydrogen, methyl, *iso*-propyl, *tert*-butyl, cyclohexyl, and adamantyl), and also in terms of the length of the spacer (n = 2 up to 10 methylene groups).

All of compounds **1a**–**79a** as well as **1b**–**79b** were fully characterized and subjected to biological testing employing *b*CA II. While no inhibitory activity for this enzyme was observed for compounds **1a**–**79a**, compounds **1b**–**79b** proved to be inhibitors of this enzyme. The results from the assays are summarized in Table 1 and the figures.

As a result, compounds **12b**, **49b**, **26b**, **30b**, **13b**, **40b**, **50b**, **33b**, and **34b** proved to be the best inhibitors of *b*CA II in this series of compounds.

With the exception of compounds **1b**–**11b**, derivatives with a shorter spacer length (preferably n = 2) show higher inhibitory activity than their longer-chain analogues. Only in group **1**–**11** is compound **7** (n = 8) the most active compound.

**Figure 4:**
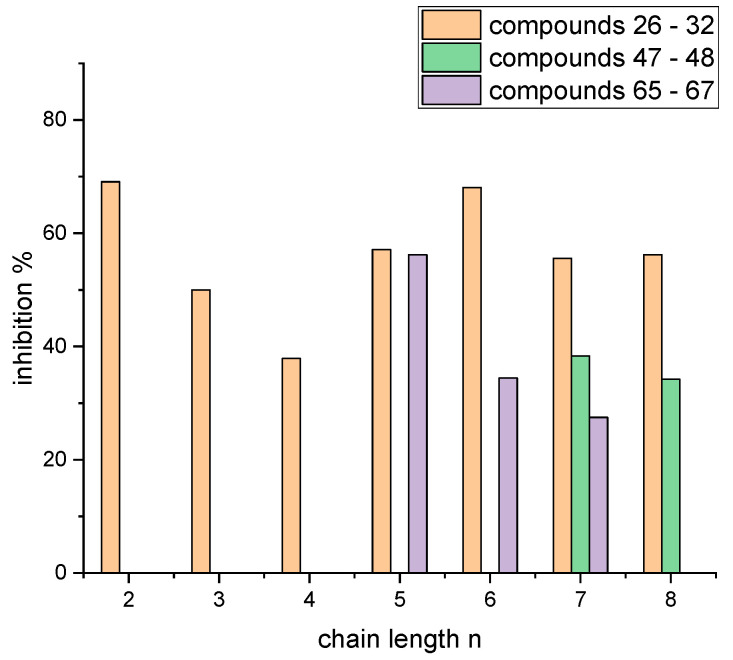
Inhibition (in %) of *ortho*-substituted compounds **26b**–**32b** (methyl), **47b**–**48b** (*iso*-propyl) and **65b**–**67b** (*tert*-butyl), respectively.

**Figure 5:**
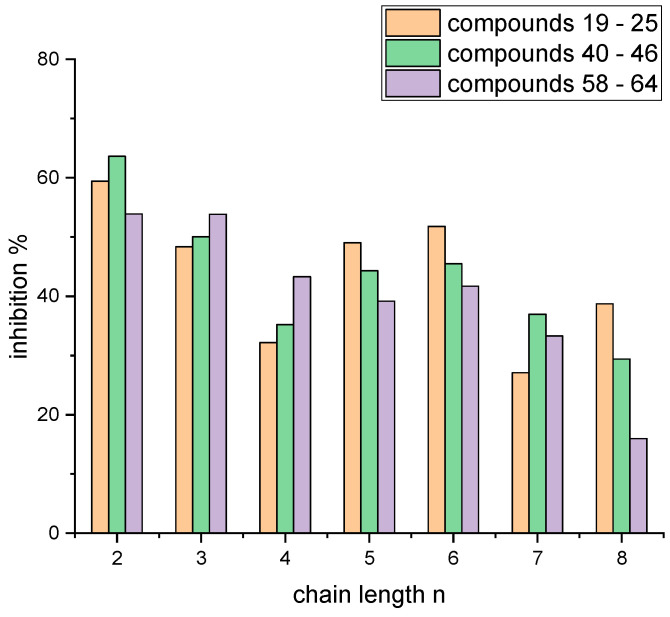
Inhibition (in %) of *meta*-substituted compounds **19b**–**25b** (methyl), **40b**–**46b** (*iso*-propyl), and **58b**–**64b** (*tert*-butyl), respectively.

**Figure 6:**
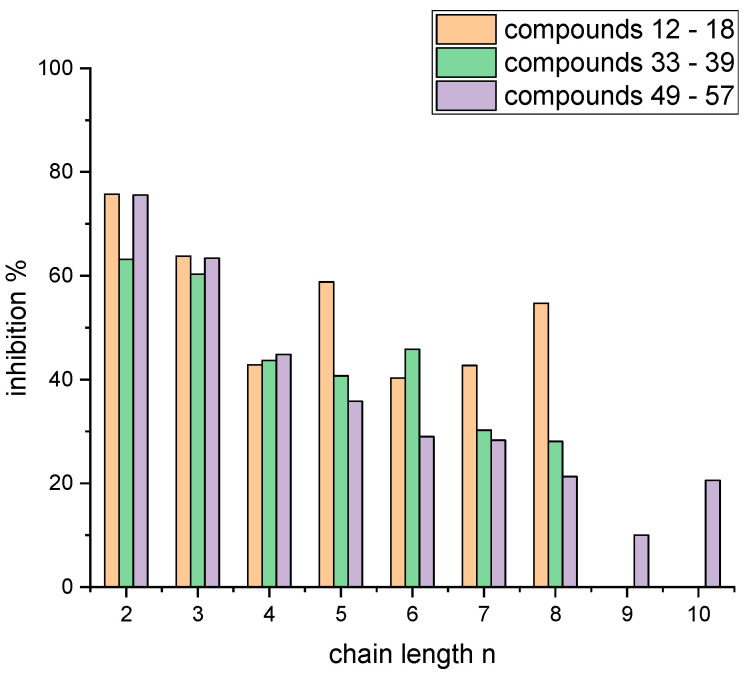
Inhibition (in %) of *para*-substituted compounds **12b**–**18b** (methyl), **33b**–**39b** (*iso*-propyl), and **49b**–**57b** (*tert*-butyl), respectively.

For the alkyl-substituted compounds **12**–**79**, an alkyl substituent in the *para* position proves to be superior to comparable compounds holding a substituent in the *meta* or *ortho* position (Figure 4, Figure 5 and Figure 6).

**Figure 7:**
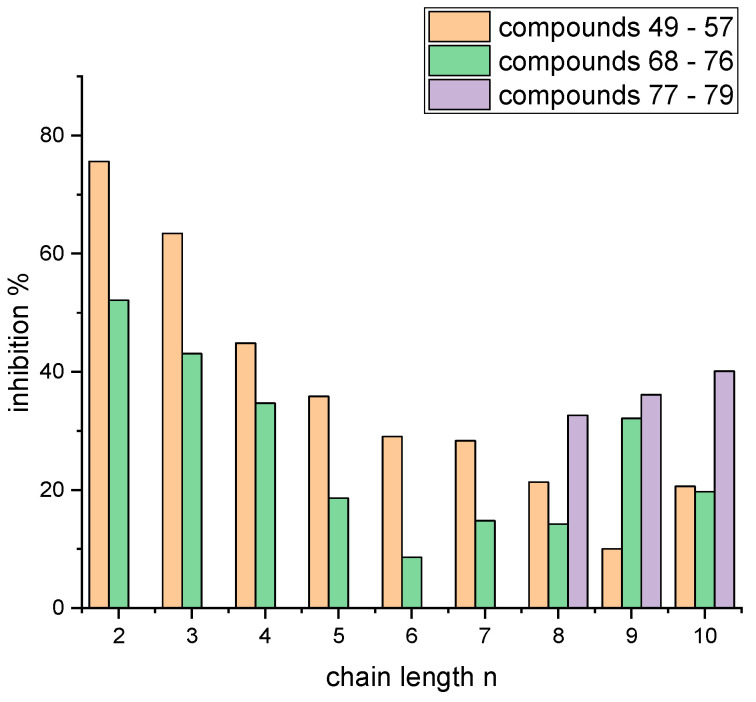
Inhibition (in %) of compounds holding the bulkiest substituents in the *para* position (^t^butyl (orange), cyclohexyl (green), adamantyl (violet)).

The large space occupancy of the *para* substituent alone is only of limited benefit in achieving higher inhibitory activity. For example, compound **12b** is significantly better than **1b**, and the activity decreases for **33b** (with the same chain length of the spacer), while ^t^Bu-substituted compound **49b** proves to be the strongest inhibitor of the whole series with I = 75.6%. Even bulkier substituents (as in **68b**–**76b** and **77b**–**79b**) with a cyclohexyl or adamanyl residue again drop significantly and prove to be poorer inhibitors for CA II (Figure 7).

**Figure 8:**
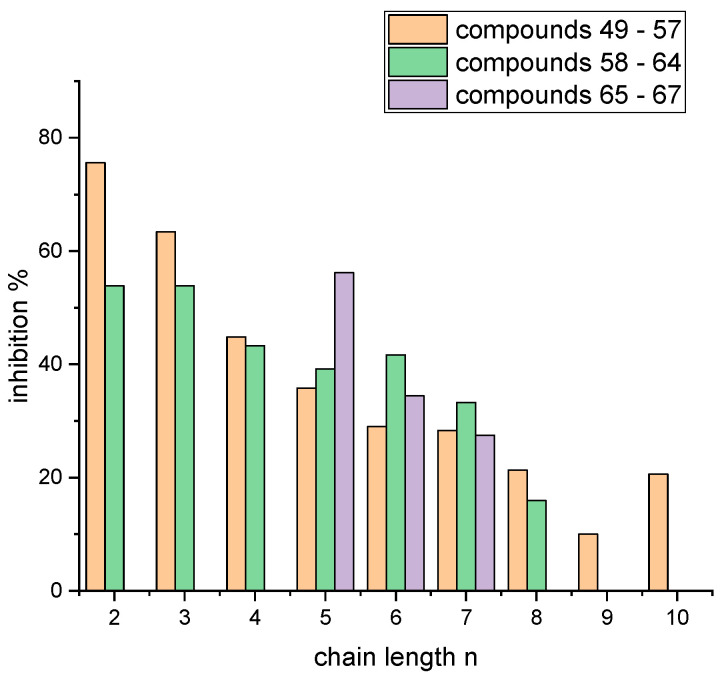
Inhibition (in %) of ^t^butyl-substituted compounds **49b**–**67b**.

A more detailed illustration of the ^t^Bu-substituted derivatives **49b**–**67b** can be found in Figure 8. 

**Figure 9:**
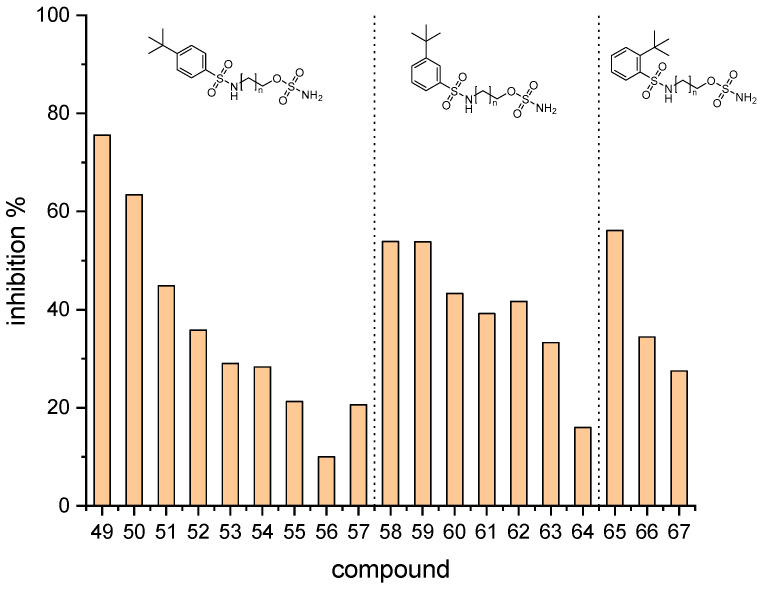
Inhibition (in %) of ^t^butyl-substituted compounds depends on position of the ^t^Bu group and on the chain length of the alkyl spacer.

For ^t^Bu-substituted compounds **49b**–**67b**, a correlation between the position of the substituent and chain length is depicted (vide supra). As already mentioned, compounds with a *para*- substituent are superior to those with *ortho*- or *meta*-terminal substituents (Figure 7, Figure 8 and Figure 9). At the same time, the dependence on the chain length of the spacer is also evident (Figure 9).

**Figure 10:**
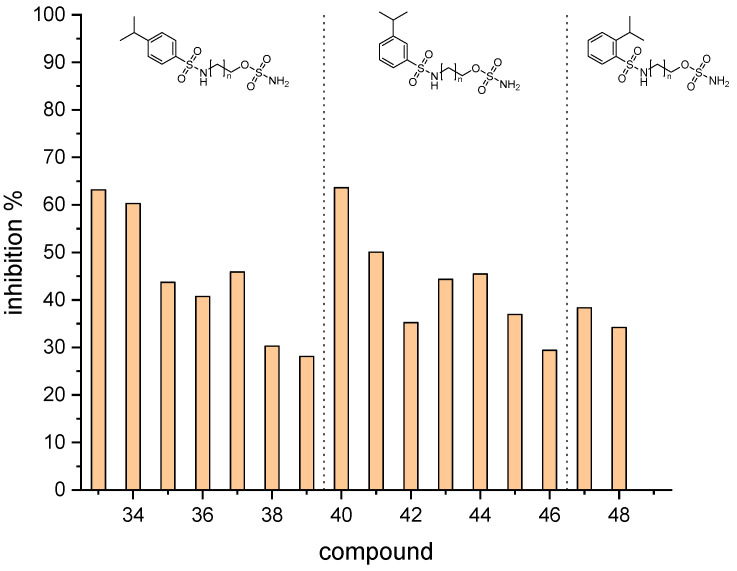
Inhibition (in %) of isopropyl-substituted compounds.

The isopropyl-substituted compounds also prove to be effective with a chain length of n = 2 (compounds **33b** and **40b**), and a decrease in inhibition can be observed with longer chains (Figure 10). 

**Figure 11:**
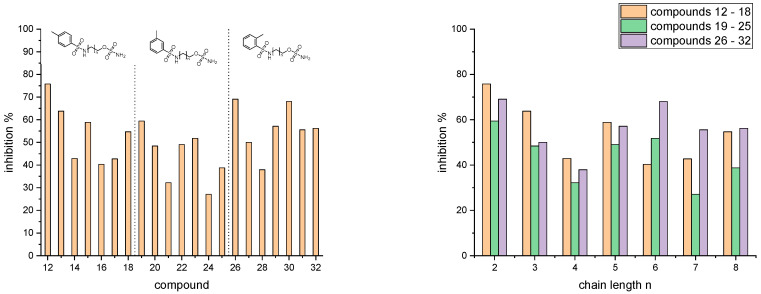
Inhibition (in %) of methyl-substituted compounds **12b**–**32b**.

**Figure 12:**
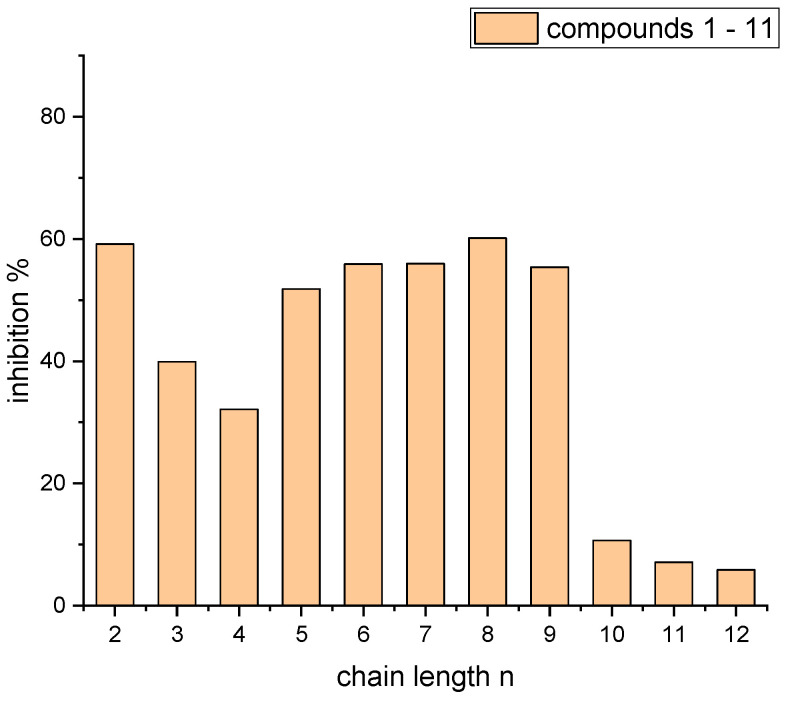
Inhibition (in %) of unsubstituted compounds **1b**–**11b**.

The same is generally true for the methyl-substituted compounds (Figure 11), although with these, the inhibition force usually increases again with longer chains (from n = 5), whereas for the unsubstituted compounds **1b**–**11b** (Figure 12), with the exception of chain lengths 3, 4 and 10–12, a fairly constant inhibition of the enzyme was observed.

**Figure 13:**
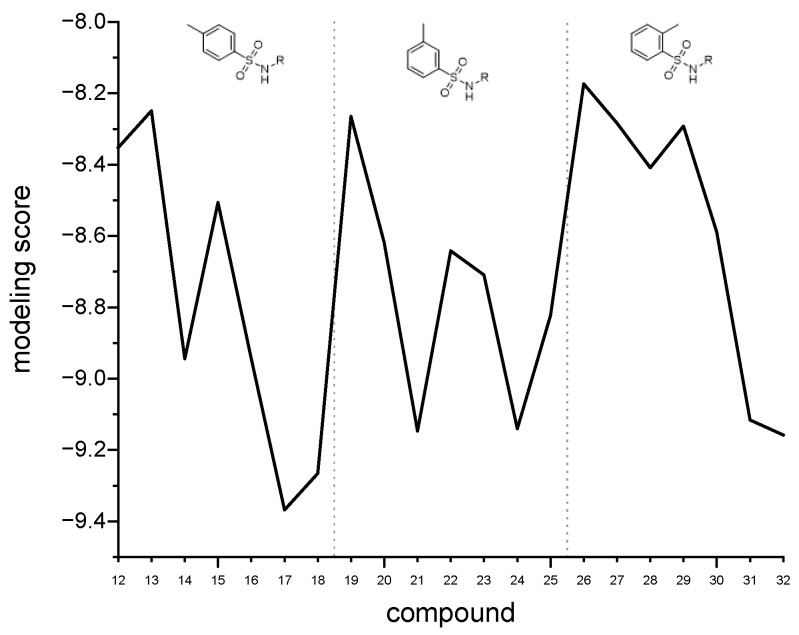
Modeling scores of the methyl-substituted compounds **12b**–**32b**.

Although we carried out many molecular modelling calculations attempting to better rationalize these empirical findings, these calculations were only partially helpful. Figure 13 depicts the calculated modeling scores for some of the compounds. By and large, the calculated scores agree with the measured biological activity (cf. Figure 11).

However, the limitations of calculations (especially for fine-tuning) are due to the fact that the molecules have relatively high degrees of (translational and rotational) freedom within the active site and the active site being relatively large. The calculations, however, confirm our earlier findings that further substitution of the spacer (especially in an α position, adjacent to the sulfonamide) should allow for better orientations of the inhibitor in the enzyme pocket, so that stronger (or weaker) interactions can be expected, depending on the absolute configuration. A depiction of the results of the modeling calculations (for compounds **12b**, **26b**, **30b**, **33b**, **40b**, and **49b**) is shown in the Supplementary Materials File. A depiction of the scores is given in Figure 13.

**Figure 14:**
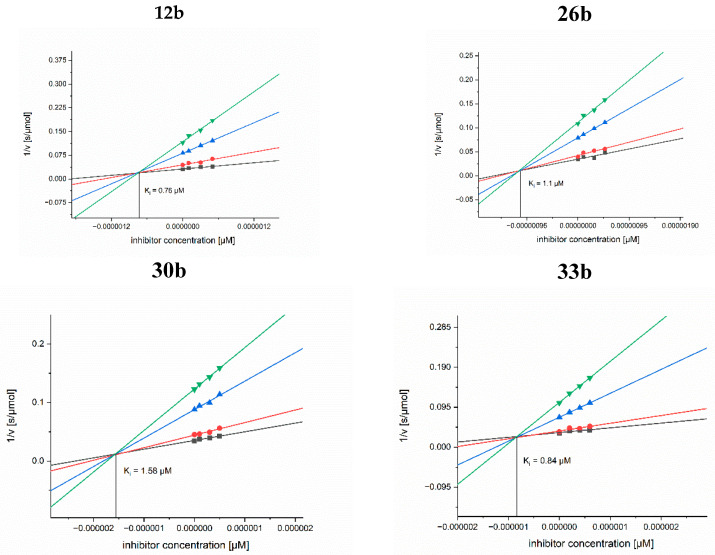

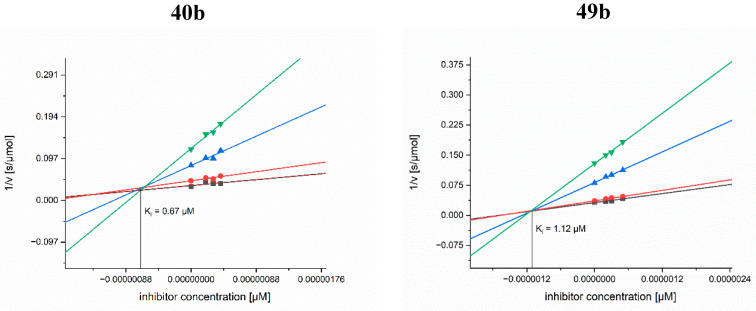
Dixon plots for compounds **12b**, **26b**, **30b**, **33b**, **40b**, and **49b**, respectively. The concentration of the inhibitor: for **12b**: 0.1, 03, 0.5 μM; for **26b**: 0.1, 0.3, 0.5 μM; for **30b**: 0.1, 0.3 0.5 μM; for **33b**: 0.2, 0.4, 0.6 μM; for **40b**: 0.1, 0.3, 0.5 μM; for **49b**: 0.2, 0.3, 0.4 μM.

Some extra measurements gave an insight into the mode of action of the most active compounds, and inhibition constants K_i_ were determined. The results from these experiments are summarized in Table 2 and depicted as Dixon plots in Figure 14.

As a result, all these compounds acted as efficient inhibitors for CA II with K_i_ values as low as 0.76 μM (for **12b**) or K_i_ = 0.67 μM (for **49b**).

Each of the sulfamates showed inhibitory activity towards *b*CA II. For every series of substituents, it is noticeable that the inhibitory activity decreases with an increase in the length of the spacer. Furthermore, substituents in the *para* position proved to be better inhibitors compared to their counterparts holding the substituent either in the *ortho* or in the *meta* position. Regarding the type of substituent, methyl and *tert*-butyl proved to be superior to the other substituents. For a better insight into the differences in the inhibition of the most active compounds, their respective inhibition constants were determined. Again, the *para* methyl and tert-butyl-substituted compounds with shorter chain lengths proved to be superior, with inhibition constants of K_i_ = 0.76 µM and 0.67 µM, respectively. With these additional measurements, the *tert*-butyl substituent proved to be the most active compound. To gain more insight into the advantages of bulkier substituents, another series of cyclohexyl- and adamantyl-substituted compounds was synthesized. However, even with short chain lengths, the inhibitory activity proved to be significantly worse than that of the *tert*-butyl or even methyl-substituted compounds. In conclusion, compounds with *tert*-butyl substituents in the *para* position and chain lengths around n = 2 proved to be the best inhibitors for *b*CA II in this library of compounds.

## 3. Conclusions

Starting with arylsulfonyl chlorides and amino alcohols with varying numbers of methylene groups between the hydroxyl and amino moieties (leading to **1a**–**79a**), a small library of 79 substituted phenylsulfonamido alkyl sulfamates **1b**–**79b** has been synthesized. These substances were tested using carbonic anhydrase II to determine whether they were inhibitive. Sulfamates **1b**–**79b** gave inhibition of the enzyme, but compounds **1a**–**79a** did not. In conclusion, the substituent, the phenyl group’s substitution pattern, and the spacer’s length all affect how inhibitive compounds **1b**–**79b** are of this enzyme. Compounds containing the same substituent in a *para* position demonstrated superior CAII inhibition than those containing the same substituent in a *meta* or *ortho* position. Compounds with shorter spacer lengths performed better than those with longer chain spacers for a variety of substitution patterns. The most active compounds held inhibition constants as low as K_i_ = 0.67 μM (for **49b**) and a *tert*-butyl substituent in a *para* position.

## 4. Experimental Procedure

### 4.1. General

Starting materials were obtained from local vendors; solvents were dried under the usual conditions; equipment and assays were as previously reported [72]. The absorbance was measured with a 96-well plate reader from BMG Labtech (BMG Labtech, Ortenberg, Germany).^1^H and ^13^C NMR spectra of all compounds as well as representative HRMS spectra can be found in the Supplementary Materials File. Chromatography was performed on silica gel; IR spectra were recorded as ATR spectra; UV/vis spectra were measured in MeOH. ^1^H NMR was measured in DMSO-d_6_ at 400 MHz; ^13^C NMR spectra were measured in DMSO-d_6_ at 101 MHz, if not stated otherwise. MS spectra were taken as ESI-MS in MeOH; for UV–Vis spectra, λ_max_ (log ε) values are reported.

### 4.2. Syntheses

#### 4.2.1. Preparation of **1a**–**79a** (General Procedure A, GPA)

Reaction of the amino-alcohol (1.5 equiv.) in dry CH_2_Cl_2_ (15 mL), with dry NEt_3_ (2 equiv.) and the sulfonyl chloride (1 equiv.) at 22 °C for 3 hours followed by evaporation of the solvents and chromatography gave **1a**–**79a**. For long-chain amino-alcohols (n = 8–12), the solvent composition was modified, and a 1:1 mixture of CH_2_Cl_2_ and acetonitrile was used instead.

#### 4.2.2. Preparation of **1b**–**79b** (General Procedure B, GPB)

Reaction of **1a**–**79a** (1 equiv.) in dry CH_2_Cl_2_ (6 mL), NEt_3_ (3 equiv.) with sulfamoyl chloride (3 equiv.) at 22 °C until completion of the reaction followed by evaporation and column chromatography gave **1b**–**79b**. 

#### 4.2.3. N-(2-Hydroxyethyl)benzene Sulfonamide (**1a**) [59724-42-4]

Applying GPA: from benzenesulfonyl chloride (500 mg, 2.83 mmol) and 2-amino-ethanol (260 mg, 4.25 mmol): **1a** [73,74,75,76,77,78,79,80] (542 mg, 94%); white solid; R_f_ = 0.07 (petrolether/EtOAc, 2:3); m.p. = 78–79 °C (lit.: [73,74] 79–80 °C); spectral data as previously reported [72]. 

#### 4.2.4. 2-(Phenylsulfonamido]ethyl Sulfamate (**1b**)

Applying GPB: from **1a** (175 mg, 0.87 mmol): **1b** (202 mg, 82%); white solid; R_f_ = 0.38 (CHCl_3_/EtOAc, 2:3); m.p. = 71–73 °C; spectral data as previously reported [72]. 

#### 4.2.5. N-(3-Hydroxypropyl)benzene Sulfonamide (**2a**) [3351-94-8]

Applying GPA: from benzenesulfonyl chloride (500 mg, 2.83 mmol) and 3-amino-propanol (319 mg, 4.25 mmol): **2a** [81,82,83] (594 mg, 97%); oil; R_f_ = 0.09 (petrolether/EtOAc, 2:3); UV–Vis: 221 nm (4.02); IR: ν = 3500*w*, 3276*m*, 2943*w*, 2881*w*, 1478*w*, 1447*m*, 1309*s*, 1154*vs*, 1092*s*, 1070*s*, 1007*w*, 959*w*, 871*w*, 754*m*, 720*m*, 689*s*, 586*s*, 469*w* cm^−1^; ^1^H NMR:δ = 7.81–7.76 (*m*, 2H, 2-H, 2′-H), 7.66–7.56 (*m*, 3H, 3-H, 3′-H, 4-H), 7.52 (*s*, 1H, NH), 4.40 (*s*, 1H, OH), 3.39–3.32 (*m*, 2H, 7-H), 2.79 *(t*, *J* = 7.3 Hz, 2H, 5-H), 1.57–1.46 (*m*, 2H, 6-H); ^13^C NMR:δ = 140.5 (C-1), 132.3 (C-4), 129.2 (C-3), 126.4 (C-2), 58.0 (C-7), 40.0 (C-5), 32.3 (C-6) ppm; MS: *m*/*z* = 238.1 (100%, [M + Na]^+^); anal. calcd. for C_9_H_13_NSO_3_ (215.27): C 50.22, H 6.09, N 6.51; found: C 49.97, H 6.34, N 6.38. 

#### 4.2.6. 3-(Phenylsulfonamido]propyl Sulfamate (**2b**)

Applying GPB: from **2a** (200 mg, 0.93 mmol): **2b** (241 mg, 88%); white solid; R_f_ = 0.40 (CHCl_3_/EtOAc, 2:3); m.p. = 64–66 °C; UV–Vis: 221 nm (3.78); IR: ν = 3344*m*, 3264*m*, 3255*m*, 1449*w*, 1373*s*, 1353*m*, 1311*s*, 1177*s*, 1155*s*, 1113*w*, 1090*m*, 1074*w*, 1052*m*, 1003*m*, 950*s*, 917*m*, 896*m*, 886*m*, 836*s*, 754*m*, 727*s*, 688*s*, 673*m*, 627*m*, 599*m*, 586*s*, 562*s*, 546*vs*, 503*m*, 490*m*, 476*w*, 441*w* cm^−1^; ^1^H NMR:δ = 7.82–7.77 (*m*, 2H, 2-H, 2′-H), 7.72–7.58 (*m*, 4H, 3-H, 3′-H, 4-H, NH), 7.41 (*s*, 2H, NH_2_), 4.02 (*t*, *J* = 6.3 Hz, 2H, 7-H), 2.85–2.78 (*m*, 2H, 5-H), 1.76 (*dt*, *J* = 13.2, 6.5 Hz, 2H, 6-H) ppm; ^13^C NMR (126 MHz, DMSO-*d*_6_): δ = 140.2 (C-1), 132.4 (C-4), 129.3 (C-3), 126.4 (C-2), 66.5 (C-7), 39.2 (C-5), 28.7 (C-6) ppm; MS: *m*/*z* = 316.9 (100%, [M + Na]^+^); anal. calcd. for C_9_H_14_N_2_S_2_O_5_ (294.34): 36.73, H 4.79, N 9.52; found: C 36.50, H 4.98, N 9.36.

#### 4.2.7. N-(4-Hydroxybutyl)benzene Sulfonamide (**3a**) [842146-77-4]

Applying GPA: from benzenesulfonyl chloride (500 mg, 2.83 mmol) and 4-aminobutanol (378 mg, 4.25 mmol): **3a** (621 mg, 96%); oil; R_f_ = 0.12 (petrolether/EtOAc, 2:3); UV–Vis: 221 nm (4.07); IR: ν = 3506*br*, 3278*br*, 2940*w*, 2872*w*, 1478*w*, 1447*m*, 1318*s*, 1309*s*, 1152*vs*, 1092*s*, 1055*m*, 1027*w*, 999*w*, 952*w*, 909*w*, 867*w*, 754*m*, 719*m*, 688*s*, 583*s*, 567*s*, 483*w* cm^−1^; ^1^H NMR (500 MHz, DMSO-*d*_6_): δ = 7.82–7.77 (*m*, 2H, 2-H, 2′-H), 7.65–7.53 (*m*, 4H, 3-H, 3′-H, 4-H, NH), 4.37 (*t*, *J* = 5.1 Hz, 1H, OH), 3.35–3.30 (*m*, 2H, 8-H), 2.74 (*q*, *J* = 6.0 Hz, 2H, 5-H), 1.44–1.33 (*m*, 4H, 6-H, 7-H) ppm; ^13^C NMR (126 MHz, DMSO-*d*_6_): δ = 140.6 (C-1), 132.3 (C-4), 129.2 (C-3), 126.4 (C-2), 60.2 (C-8), 42.6 (C-5), 29.5 (C-7), 25.8 (C-6) ppm; MS: *m*/*z* = 243.2 (90%, [M-H]^−^); anal. calcd. for C_10_H_15_NSO_3_ (229.29): C 50.22, H 6.09, N 6.51; found: C 49.97, H 6.31, N 6.37. 

#### 4.2.8. 4-(Phenylsulfonamido]butyl Sulfamate (**3b**)

Applying GPB: from **3a** (200 mg, 0.87 mmol): **3b** (215 mg, 80%); oil; R_f_ = 0.43 (CHCl_3_/EtOAc, 2:3); UV–Vis: 221 nm (3.95); IR: ν = 3345*m*, 3258*m*, 3257*m*, 1442*w*, 1370*s*, 1350*m*, 1316*s*, 1187*s*, 1157*s*, 1110*w*, 1093*m*, 1072*w*, 1056*m*, 1001*m*, 956*s*, 914*m*, 894*m*, 889*m*, 840*s*, 751*m*, 729*s*, 685*s*, 670*m*, 621*m*, 595*m*, 582*s*, 565*s*, 544*vs*, 501*m*, 496*m*, 470*w*, 440*w* cm^−1^; ^1^H NMR:δ = 7.83–7.76 (*m*, 2H, 2-H, 2′-H), 7.68–7.56 (*m*, 4H, 3-H, 3′-H, 4-H, NH), 7.38 (*s*, 2H, NH_2_), 3.96 (*t*, *J* = 6.3 Hz, 2H, 8-H), 2.76 (*q*, *J* = 6.6 Hz, 2H, 5-H), 1.66–1.56 (*m*, 2H, 7-H), 1.49–1.40 (*m*, 2H, 6-H) ppm; ^13^C NMR:δ = 140.5 (C-1), 132.4 (C-4), 129.2 (C-3), 126.4 (C-2), 68.6 (C-8), 42.0 (C-5), 25.6 (C-7), 24.7 (C-6) ppm; MS: *m*/*z* = 331.1 (100%, [M + Na]^+^); anal. calcd. for C_10_H_16_N_2_S_2_O_5_ (308.37): C 38.95, H 5.23, N 9.08; found: C 38.70, H 5.51, N 8.84.

#### 4.2.9. N-(5-Hydroxypentyl)benzene Sulfonamide (**4a**) [191529-31-4]

Applying GPA: from benzenesulfonyl chloride (500 mg, 2.83 mmol) and 5-amino-pentanol (438 mg, 4.25 mmol): **4a** [84,85] (647 mg, 94%); oil; R_f_ = 0.12 (petrolether/EtOAc, 2:3); UV–Vis: 221 nm (3.91); IR: ν = 3503*w*, 3281*m*, 2937*w*, 2865*w*, 1478*w*, 1447*m*, 1320*s*, 1309*s*, 1153*vs*, 1092*s*, 1071*s*, 1040*w*, 999*w*, 880*w*, 754*s*, 719*m*, 689*s*, 583*s*, 567*s*, 464*w* cm^−1^; ^1^H NMR:δ = 7.82–7.76 (*m*, 2H, 2-H, 2′-H), 7.66–7.56 (*m*, 3H, 3-H, 3′-H, 4-H), 7.54 (*t*, *J* = 5.8 Hz, 1H, NH), 4.30 (*t*, *J* = 5.1 Hz, 1H, OH), 3.36–3.28 (*m*, 2H, 9-H), 2.71 (*td*, *J* = 7.0, 5.7 Hz, 2H, 5-H), 1.40–1.27 (*m*, 4H, 6-H, 8-H), 1.27–1.16 (*m*, 2H, 7-H) ppm; ^13^C NMR:δ = 140.6 (C-1), 132.2 (C-4), 129.1 (C-3), 126.4 (C-2), 60.5 (C-9), 42.6 (C-5), 32.0 (C-8), 28.8 (C-6), 22.6 (C-7) ppm; MS: *m*/*z* = 243.2 (90%, [M-H]^−^); anal. calcd. for C_11_H_17_NSO_3_ (243.32): C 54.30, H 7.04, N 5.76; found: C 54.11, H 7.23, N 5.43.

#### 4.2.10. 5-(Phenylsulfonamido]pentyl Sulfamate (**4b**)

Applying GPB: from **4a** (200 mg, 0.82 mmol): **4b** (250 mg, 94%); oil; R_f_ = 0.46 (CHCl_3_/EtOAc, 2:3); UV–Vis: 221 nm (3.91); IR: ν = 3344*w*, 3307*m*, 3232*w*, 2953*w*, 2933*w*, 2854*w*, 1555*w*, 1469*w*, 1442*w*, 1421*w*, 1399*vw*, 1363*s*, 1311*s*, 1295*w*, 1276*w*, 1172*s*, 1153*vs*, 1092*m*, 1078*m*, 1067*w*, 1043*w*, 1025*w*, 993*m*, 945*m*, 922*s*, 911*s*, 808*m*, 766*m*, 750*m*, 722*s*, 693*s*, 599*s*, 570*s*, 553*vs*, 502*m*, 482*m*, 435*w* cm^−1^; ^1^H NMR:δ = 7.82–7.76 (*m*, 2H, 2-H, 2′-H), 7.67–7.55 (*m*, 4H, 3-H, 3′-H, 4-H, NH), 7.39–7.35 (*m*, 2H, NH_2_), 3.96 (*t*, *J* = 6.5 Hz, 2H, 9-H), 2.73 (*td*, *J* = 6.7, 5.8 Hz, 2H, 5-H), 1.55 (*p*, *J* = 6.7 Hz, 2H, 6-H), 1.44–1.34 (*m*, 2H, 8-H), 1.33–1.23 (*m*, 2H, 7-H) ppm; ^13^C NMR:δ = 140.6 (C-1), 132.2 (C-4), 129.1 (C-3), 126.4 (C-2), 60.5 (C-9), 42.6 (C-5), 32.0 (C-8), 28.8 (C-6), 22.6 (C-7) ppm; MS: *m*/*z* = 345.1 (100%, [M + Na]^+^); anal. calcd. for C_11_H_18_N_2_S_2_O_5_ (322.39): C 40.98, H 5.63, N 8.69; found: C 40.72, H 5.87, N 8.57. 

#### 4.2.11. N-(6-Hydroxyhexyl)benzene Sulfonamide (**5a**) [195003-60-2]

Applying GPA: from benzenesulfonyl chloride (500 mg, 2.83 mmol) and 6-amino-hexanol (438 g, 4.25 mmol): **5a** (701 mg, 96%); white solid; R_f_ = 0.14 (petrolether/EtOAc, 2:3); m.p. = 40–42 °C; UV–Vis: 221 nm (3.97); IR: ν = 3506*br*, 3260*br*, 2934*w*, 2860*w*, 1447*m*, 1320*m*, 1309*m*, 1153*vs*, 1092*m*, 1071*m*, 1025*w*, 1000*w*, 754*m*, 719*m*, 689*s*, 584*s*, 568*s*, 475*w* cm^−1^; ^1^H NMR (500 MHz, DMSO-*d*_6_): δ = 7.81–7.76 (*m*, 2H, 2-H, 2′-H), 7.65–7.56 (*m*, 3H, 3-H, 3′-H, 4-H), 7.54 (*t*, *J* = 5.8 Hz, 1H, NH), 4.30 (*t*, *J* = 5.2 Hz, 1H, OH), 3.36–3.31 (*m*, 2H, 10-H), 2.72 (*td*, *J* = 7.0, 5.7 Hz, 2H, 5-H), 1.38–1.30 (*m*, 4H, 6-H, 9-H), 1.38–1.14 (*m*, 4H, 7-H, 8-H) ppm; ^13^C NMR (126 MHz, DMSO-*d*_6_): δ = 140.6 (C-1), 132.2 (C-4), 129.1 (C-3), 126.4 (C-2), 60.6 (C-10), 42.5 (C-5), 32.3 (C-9), 29.0 (C-6), 25.9 (C-8), 25.0 (C-7) ppm; MS: *m*/*z* = 280 (100%, [M + Na]^+^); anal. calcd. for C_12_H_19_NSO_3_ (257.35): C 56.01, H 7.44, N 5.44; found: C 55.76, H 7.69, N 5.21.

#### 4.2.12. 6-(Phenylsulfonamido]hexyl Sulfamate (**5b**)

Applying GPB: from **5b** (230 mg, 0.89 mmol): **5b** (202 mg, 67%); white solid; R_f_ = 0.51 (CHCl_3_/EtOAc, 2:3); m.p. = 64–66 °C; UV–Vis: 221 nm (3.98); IR: ν = 3346*w*, 3306*m*, 3238*w*, 2955*w*, 2931*w*, 2858*w*, 1554*w*, 1465*w*, 1447*w*, 1421*w*, 1390*vw*, 1366*s*, 1314*s*, 1291*w*, 1279*w*, 1171*s*, 1155*vs*, 1095*m*, 1074*m*, 1063*w*, 1046*w*, 1025*w*, 993*m*, 946*m*, 926*s*, 912*s*, 808*m*, 761*m*, 751*m*, 720*s*, 690*s*, 598*s*, 571*s*, 550*vs*, 507*m*, 481*m*, 436*w* cm^−1^; ^1^H NMR:δ = 7.81–7.77 (*m*, 2H, 2-H, 2′-H), 7.66–7.53 (*m*, 4H, 3-H, 3′-H, 4-H, NH), 7.37 (*s*, 2H, NH_2_), 3.97 (*t*, *J* = 6.5 Hz, 2H, 10-H), 2.77–2.69 (*m*, 2H, 5-H), 1.56 (*p*, *J* = 6.7 Hz, 2H, 9-H), 1.35 (*p*, *J* = 6.9 Hz, 2H, 6-H), 1.30–1.17 (*m*, 4H, 7-H, 8-H) ppm; ^13^C NMR (126 MHz, DMSO-*d*_6_): δ = 140.6 (C-1), 132.3 (C-4), 129.2 (C-3), 126.4 (C-2), 68.9 (C-10), 42.4 (C-5), 28.8 (C-9), 28.2 (C-6), 25.5 (C-8), 24.6 (C-7) ppm; MS: *m*/*z* = 359.3 (100%, [M + Na]^+^); anal. calcd. for C_12_H_20_N_2_S_2_O_5_ (336.42): C 42.84, H 5.99, N 8.33; found: C 42.57, H 6.17, N 8.04.

#### 4.2.13. N-(7-Hydroxyheptyl)benzene Sulfonamide (**6a**) [2773002-10-9]

Applying GPA: from benzenesulfonyl chloride (500 mg, 2.83 mmol) and 7-amino-heptanol (558 mg, 4.25 mmol): **6a** (740 mg, 96%); oil; R_f_ = 0.19 (petrolether/EtOAc, 2:3); UV–Vis: 221 nm (3.97); IR: ν = 3504*br*, 3282*br*, 2931*m*, 2858*w*, 1447*m*, 1320*m*, 1310*m*, 1153*vs*, 1093*s*, 1071*m*, 1058*w*, 1025*m*, 1000*w*, 755*m*, 719*m*, 689*s*, 584*s*, 568*s*, 469*w* cm^−1^; ^1^H NMR (500 MHz, DMSO-*d*_6_): δ = 7.81–7.77 (*m*, 2H, 2-H, 2′-H), 7.65–7.56 (*m*, 3H, 3-H, 3′-H, 4-H), 7.54 (*t*, *J* = 5.7 Hz, 1H, NH), 4.30 (*t*, *J* = 5.1 Hz, 1H, OH), 3.37–3.32 (*m*, 3H, 11-H), 2.72 (*q*, *J* = 6.7 Hz, 2H, 5-H), 1.40–1.30 (*m*, 4H, 6-H, 10-H), 1.23–1.11 (*m*, 6H, 7-H, 8-H, 9-H) ppm; ^13^C NMR:δ = 140.7 (C-1), 132.2 (C-4), 129.1 (C-3), 126.4 (C-2), 60.7 (C-11), 42.5 (C-5), 32.4 (C-10), 28.9 (C-7), 28.4 (C-6), 26.0 (C-9), 25.3 (C-8) ppm; MS: *m*/*z* = 294.2 (100%, [M + Na]^+^); anal. calcd. for C_13_H_21_NSO_3_ (271.38): C 57.54, H 7.80, N 5.16; found: C 57.26, H 8.03, N 4.97.

#### 4.2.14. 7-(Phenylsulfonamido]heptyl Sulfamate (**6b**)

Applying GPB: from **6a** (300 mg, 1.11 mmol): **6b** (315 mg, 81%); white solid; R_f_ = 0.60 (CHCl_3_/EtOAc, 2:3); m.p. = 59–61 °C; UV–Vis: 221 nm (3.99); IR: ν = 3377*w*, 3273*m*, 2959*w*, 2938*m*, 2924*w*, 2860*w*, 1545*w*, 1477*w*, 1449*m*, 1423*m*, 1397*w*, 1374*s*, 1310*s*, 1291*m*, 1229*vw*, 1188*s*, 1152*vs*, 1115*vw*, 1091*s*, 1063*m*, 1049*w*, 1007*m*, 998*m*, 969*s*, 908*m*, 852*vw*, 820*s*, 754*m*, 722*s*, 686*s*, 590*s*, 567*s*, 555*vs*, 524*vs*, 473*w*, 448*w*, 441*w* cm^−1^; ^1^H NMR:δ = 7.81–7.77 (*m*, 2H, 2-H, 2′-H), 7.66–7.52 (*m*, 4H, 3-H, 3′-H, 4-H, NH), 7.37 (*s*, 2H, NH_2_), 3.98 (*t*, *J* = 6.5 Hz, 2H, 11-H), 2.76–2.69 (*m*, 2H, 5-H), 1.62–1.53 (*m*, 2H, 10-H), 1.39–1.30 (*m*, 2H, 6-H), 1.28–1.15 (*m*, 6H, 7-H, 8-H, 9-H) ppm; ^13^C NMR:δ = 140.7 (C-1), 132.3 (C-4), 129.2 (C-3), 126.4 (C-2), 68.9 (C-11), 42.5 (C-5), 28.8 (C-10), 28.2 (C-7), 28.0 (C-6), 25.8 (C-9), 24.9 (C-8) ppm; MS: *m*/*z* = 373.3 (100%, [M + Na]^+^); anal. calcd. for C_13_H_22_N_2_S_2_O_5_ (350.45): C 44.56, H 6.33, N 7.99; found: C 44.47, H 6.50, N 7.76. 

#### 4.2.15. N-(8-Hydroxyoctyl)benzene Sulfonamide (**7a**) [2771470-62-1]

Applying GPA: from benzenesulfonyl chloride (500 mg, 2.83 mmol) and 8-amino-octanol (617 mg, 4.25 mmol): **7a** (566 mg, 70%); white solid; R_f_ = 0.21 (petrolether/EtOAc, 2:3); m.p. = 59–60 °C; UV–Vis: 221 nm (3.99); IR: ν = 3280*m*, 2929*m*, 2854*m*, 1478*w*, 1447*m*, 1428*m*, 1326*s*, 1266*w*, 1158*vs*, 1092*s*, 1053*m*, 1031*w*, 982*w*, 905*m*, 755*m*, 720*m*, 687*s*, 596*s*, 563*s*, 530*m*, 500*w*, 481*w*, 455*w* cm^−1^; ^1^H NMR:δ = 7.81–7.76 (*m*, 2H, 2-H, 2′-H), 7.66–7.56 (*m*, 3H, 3-H, 3′-H, 4-H), 7.54 (*t*, *J* = 5.8 Hz, 1H, NH), 4.30 (*t*, *J* = 5.1 Hz, 1H, OH), 3.36 (*td*, *J* = 6.5, 5.1 Hz, 2H, 12-H), 2.72 (*q*, *J* = 6.7 Hz, 2H, 5-H), 1.42–1.27 (*m*, 4H, 6-H, 11-H), 1.27–1.09 (*m*, 8H, 7-H, 8-H, 9-H, 10-H) ppm; ^13^C NMR (126 MHz, DMSO-*d*_6_): δ = 140.7 (C-1), 132.2 (C-4), 129.1 (C-3), 126.4 (C-2), 60.7 (C-12), 42.5 (C-5), 32.5 (C-11), 28.9 (C-9), 28.7 (C-6), 28.5 (C-8), 25.9 (C-7), 25.4 (C-10) ppm; MS: *m*/*z* = 308.3 (100%, [M + Na]^+^); anal. calcd. for C_14_H_23_NSO_3_ (285.40): C 58.92, H 8.12, N 4.91; found: C 58.76, H 8.37, N 4.63.

#### 4.2.16. 8-(Phenylsulfonamido]octyl Sulfamate (**7b**)

Applying GPB: from **7a** (300 mg, 1.05 mmol): **7b** (203 mg, 53%); white solid; R_f_ = 0.64 (CHCl_3_/EtOAc, 2:3); m.p. = 77–78 °C; UV–Vis: 221 nm (3.95); IR: ν = 3338*w*, 3267*m*, 2934*w*, 2916*w*, 2856*w*, 1577*w*, 1469*w*, 1450*w*, 1427*w*, 1395*w*, 1381*s*, 1311*m*, 1179*s*, 1158*vs*, 1116*vw*, 1092*m*, 1065*w*, 1058*m*, 1039*w*, 1025*vw*, 997*m*, 965*s*, 931*m*, 898*m*, 858*m*, 821*s*, 794*m*, 753*s*, 726*s*, 688*s*, 588*vs*, 566*s*, 549*s*, 526*s*, 480*w*, 468*w*, 440*w* cm^−1^; ^1^H NMR:δ = 7.82–7.76 (*m*, 2H, 2-H, 2′-H), 7.67–7.56 (*m*, 3H, 3-H, 3′-H, 4-H), 7.40 (*s*, 3H, NH, NH_2_), 3.99 (*t*, *J* = 6.5 Hz, 2H, 12-H), 2.72 (*t*, *J* = 7.0 Hz, 2H, 5-H), 1.65–1.54 (*m*, 2H, 11-H), 1.39–1.23 (*m*, 4H, 6-H, 10-H), 1.23–1.14 (*m*, 6H, 7-H, 8-H, 9-H) ppm; ^13^C NMR:δ = 140.7 (C-1), 132.2 (C-4), 129.1 (C-3), 126.4 (C-2), 69.0 (C-12), 42.5 (C-5), 28.9 (C-11), 28.3 (C-9), 28.3 (C-6), 28.3 (C-8), 25.9 (C-7), 24.9 (C-10) ppm; MS: *m*/*z* = 387.3 (100%, [M + Na]^+^); anal. calcd. for C_14_H_24_N_2_S_2_O_5_ (364.48): C 46.14, H 6.64, N 7.69; found: C 45.87, H 6.84, N 7.55.

#### 4.2.17. N-(9-Hydroxynonyl)benzene Sulfonamide (**8a**)

Applying GPA: from benzenesulfonyl chloride (500 mg, 2.83 mmol) and 9-amino-nonanol (676 mg, 4.25 mmol): **8a** (745 mg, 88%); white solid; R_f_ = 0.23 (petrolether/EtOAc, 2:3); m.p. = 40–41 °C; UV–Vis: 221 nm (3.97); IR: ν = 3465*br*, 3263*br*, 2930*m*, 2917*w*, 2892*w*, 2849*w*, 1475*w*, 1445*m*, 1433*w*, 1313*s*, 1157*s*, 1094*s*, 1058*s*, 1051*s*, 1036*m*, 1023*w*, 974*m*, 928*w*, 904*m*, 878*m*, 824*m*, 752*m*, 726*m*, 689*s*, 665*m*, 595*w*, 571*s*, 560v*s*, 522*s*, 470*w*, 460*w*, 439*w* cm^−1^; ^1^H NMR:δ = 17.81–7.76 (*m*, 2H, 2-H, 2′-H), 7.66–7.56 (*m*, 3H, 3-H, 3′-H, 4-H), 7.54 (*s*, 1H, NH), 4.30 (*t*, *J* = 5.1 Hz, 1H, OH), 3.36 (*td*, *J* = 6.5, 4.7 Hz, 2H, 13-H), 2.72 (*t*, *J* = 7.0 Hz, 2H, 5-H), 1.43–1.28 (*m*, 4H, 6-H, 12-H), 1.28–1.11 (*m*, 10H, 7-H, 8-H, 9-H, 10-H, 11-H) ppm; ^13^C NMR:δ = 140.7 (C-1), 132.2 (C-4), 129.1 (C-3), 126.4 (C-2), 60.7 (C-13), 42.5 (C-5), 32.5 (C-12), 28.9 (C-9), 28.9 (C-10), 28.8 (C-6), 28.5 (C-8), 26.0 (C-7), 25.4 (C-11) ppm; MS: *m*/*z* = 322.3 (100%, [M + Na]^+^); anal. calcd. for C_15_H_25_NSO_3_ (299.43): C 60.17, H 8.42, N 4.68; found: C 59.87, H 8.70, N 4.39.

#### 4.2.18. 9-(Phenylsulfonamido]nonyl Sulfamate (**8b**)

Applying GPB: from **8a** (300 mg, 1.00 mmol): **8b** (267 mg, 70%); white solid; R_f_ = 0.68 (CHCl_3_/EtOAc, 2:3); m.p. = 82–84 °C; UV–Vis: 221 nm (3.92); IR: ν = 3378*w*, 3273*m*, 2937*w*, 2922*m*, 2855*w*, 1545*w*, 1476*w*, 1449*w*, 1424*w*, 1397*w*, 1375*s*, 1309*s*, 1286*w*, 1275*w*, 1188*s*, 1153*vs*, 1118*vw*, 1092*m*, 1063*m*, 1033*m*, 994*w*, 969*s*, 910*m*, 883*m*, 820*s*, 776*w*, 753*m*, 723*s*, 686*s*, 589*s*, 568*s*, 555*vs*, 534*m*, 524*m*, 500*w*, 489*m*, 465*w*, 453*w* cm^−1^; ^1^H NMR:δ = 7.81–7.76 (*m*, 2H, 2-H, 2′-H), 7.66–7.55 (*m*, 3H, 3-H, 3′-H, 4-H), 7.41 (*s*, 3H, NH, NH_2_), 4.00 (*t*, *J* = 6.5 Hz, 2H, 13-H), 2.72 (*t*, *J* = 7.0 Hz, 2H, 5-H), 1.65–1.56 (*m*, 2H, 12-H), 1.37–1.27 (*m*, 4H, 6-H, 11-H), 1.25–1.13 (*m*, 8H, 7-H, 8-H, 9-H, 10-H) ppm; ^13^C NMR:δ = 140.7 (C-1), 132.2 (C-4), 129.1 (C-3, C-3′), 126.4 (C-2, C-2′), 69.0 (C-13), 42.5 (C-5), 28.9 (C-6), 28.7 (C-8), 28.4 (C-9), 28.4 (C-10), 28.3 (C-12), 25.9 (C-7), 25.0 (C-11) ppm; MS: *m*/*z* = 401.3 (100%, [M + Na]^+^); anal. calcd. for C_15_H_26_N_2_S_2_O_5_ (378.50): C 47.60, H 6.92, N 7.40; found: C 47.40, H 7.11, N 7.06. 

#### 4.2.19. N-(10-Hydroxydecyl)benzene Sulfonamide (**9a**)

Applying GPA: from benzenesulfonyl chloride (220 mg, 1.25 mmol) and 10-amino-decanol (324 mg, 4.25 mmol): **9a** (350 mg, 90%); white solid; R_f_ = 0.28 (petrolether/EtOAc, 2:3); m.p. = 67–69 °C; UV–Vis: 221 nm (3.95); IR: ν = 3416*m*, 3279*m*, 2920*w*, 2889*w*, 2849*m*, 1465*m*, 1447*m*, 1426*m*, 1350*m*, 1318*s*, 1287*w*, 1153*vs*, 1094*m*, 1059*m*, 1033*w*, 1009*w*, 973*w*, 909*m*, 750*m*, 722*s*, 684*s*, 596v*s*, 581*s*, 531*w*, 515*m*, 429*w*, 475*w* cm^−1^; ^1^H NMR:δ = 7.80–7.76 (*m*, 2H, 2-H, 2′-H), 7.66–7.56 (*m*, 3H, 3-H, 3′-H, 4-H), 7.53 (*t*, *J* = 5.8 Hz, 1H, NH), 4.30 (*s*, 1H, OH), 3.40–3.34 (*m*, 2H, 15-H), 2.71 (*td*, *J* = 7.0, 5.8 Hz, 2H, 5-H), 1.43–1.28 (*m*, 4H, 6-H, 14-H), 1.27–1.10 (*m*, 12H, 7-H, 8-H, 9-H, 10-H, 11-H, 12-H) ppm; ^13^C NMR (126 MHz, DMSO-*d*_6_): δ = 140.7 (C-1), 132.2 (C-4), 129.1 (C-3), 126.4 (C-2), 60.7 (C-15), 42.5 (C-5), 32.5 (C-14), 29.0 (C-6), 28.9 (C-8, C-11), 28.8 (C-9), 28.5 (C-10), 26.0 (C-7), 25.5 (C-12) ppm; MS: *m*/*z* = 336.4 (100%, [M + Na]^+^); anal. calcd. for C_16_H_27_NSO_3_ (313.46): C 61.31, H 8.68, N 4.47; found: C 61.07, H 8.95, N 4.24.

#### 4.2.20. 10-(Phenylsulfonamido]decyl Sulfamate (**9b**)

Applying GPB: from **9a** (185 mg, 0.59 mmol): **9b** (172 mg, 74%); white solid; R_f_ = 0.71 (CHCl_3_/EtOAc, 2:3); m.p. = 89–91 °C; UV–Vis: 221 nm (4.02); IR: ν = 3339*w*, 3266*m*, 2962*vw*, 2933*w*, 2917*m*, 2849*w*, 1576*vw*, 1470*w*, 1450*w*, 1428*w*, 1395*w*, 1381*s*, 1336*vw*, 1313*m*, 1308*m*, 1287*w*, 1266*vw*, 1235*vw*, 1178*m*, 1159*vs*, 1118*vw*, 1093*m*, 1078*w*, 1059*m*, 1042*w*, 1020*w*, 990*m*, 965*s*, 932*m*, 918*m*, 892*m*, 855*w*, 822*s*, 764*w*, 753*m*, 725*s*, 688*s*, 590*s*, 567*s*, 549*s*, 534*s*, 511*w*, 483*w*, 458*w* cm^−1^; ^1^H NMR:δ = 7.81–7.76 (*m*, 2H, 2-H, 2′-H), 7.66–7.56 (*m*, 3H, 3-H, 3′-H, 4-H), 7.53 (*t*, *J* = 5.8 Hz, 1H, NH), 7.37 (*s*, 2H, NH_2_), 4.00 (*t*, *J* = 6.5 Hz, 2H, 15-H), 2.72 (*td*, *J* = 7.0, 5.8 Hz, 2H, 5-H), 1.65–1.57 (*m*, 2H, 14-H), 1.39–1.27 (*m*, 4H, 6-H, 12-H), 1.27–1.12 (*m*, 10H, 7-H, 8-H, 9-H, 10-H, 11-H) ppm; ^13^C NMR:δ = 140.7 (C-1), 132.2 (C-4), 129.1 (C-3), 126.4 (C-2), 69.0 (C-15), 42.5 (C-5), 28.9 (C-6), 28.8 (C-9, C-14), 28.5 (C-8, C-11), 28.3 (C-10), 25.9 (C-7), 25.0 (C-12) ppm; MS: *m*/*z* = 415.2 (100%, [M + Na]^+^); anal. calcd. for C_16_H_28_N_2_S_2_O_5_ (392.53): C 48.96, H 7.19, N 7.14; found: C 48.75, H 7.35, N 6.87.

#### 4.2.21. N-(11-Hydroxyundecyl)benzene Sulfonamide (**10a**)

Applying GPA: from benzenesulfonyl chloride (500 mg, 2.83 mmol) and 11-amino-undecanol (795 mg, 4.25 mmol): **10a** (901 mg, 97%); white solid; R_f_ = 0.33 (petrolether/EtOAc, 2:3); m.p. = 58–59 °C; UV–Vis: 221 nm (3.97); IR: ν = 3464*m*, 3265*m*, 2918*m*, 2848*m*, 1475*m*, 1466*m*, 1445*m*, 1432*w*, 1353*m*, 1314*s*, 1287*w*, 1158*vs*, 1094*s*, 1052*s*, 1008*m*, 928*w*, 894*w*, 885*m*, 819*m*, 753*s*, 725*s*, 689*s*, 664*m*, 595*m*, 560*s*, 529*w*, 510*m*, 493*w*, 410*w* cm^−1^; ^1^H NMR:δ = 7.81–7.76 (*m*, 2H, 2-H, 2′-H), 7.66–7.56 (*m*, 3H, 3-H, 3′-H, 4-H), 7.53 (*t*, *J* = 5.8 Hz, 1H, NH), 4.30 (*td*, *J* = 5.2, 1.0 Hz, 1H, OH), 3.37 (*td*, *J* = 6.5, 5.1 Hz, 2H, 15-H), 2.71 (*td*, *J* = 7.0, 5.8 Hz, 2H, 5-H), 1.45–1.28 (*m*, 4H, 6-H, 14-H), 1.28–1.11 (*m*, 14H, 7-H, 8-H, 9-H, 10-H, 11-H, 12-H, 13-H) ppm; ^13^C NMR (126 MHz, DMSO-*d*_6_): δ = 140.7 (C-1), 132.2 (C-4), 129.1 (C-3), 126.4 (C-2), 60.7 (C-15), 42.5 (C-5), 32.5 (C-14), 29.0 (C-6), 28.9 (C-12), 28.9 (C-11), 28.9 (C-10), 28.8 (C-9), 28.5 (C-8), 25.9 (C-7), 25.5 (C-13) ppm; MS: *m*/*z* = 350.2 (100%, [M + Na]^+^); anal. calcd. for C_17_H_29_NSO_3_ (327.48): C 62.35, H 8.93, N 4.28; found: C 62.17, H 9.16, N 4.01. 

#### 4.2.22. 11-(Phenylsulfonamido]undecyl Sulfamate (**10b**)

Applying GPB: from **10a** (300 mg, 0.92 mmol): **10b** (321 mg, 86%); white solid; R_f_ = 0.74 (CHCl_3_/EtOAc, 2:3); m.p. = 95–97 °C; UV–Vis: 221 nm (3.99); IR: ν = 3392*w*, 3269*m*, 2963*w*, 2936*w*, 2917*m*, 2852*w*, 1558*w*, 1476*m*, 1447*w*, 1429*w*, 1395*w*, 1369*s*, 1314*s*, 1258*vw*, 1183*m*, 1153*s*, 1120*w*, 1093*m*, 1066*m*, 1051*m*, 1028*w*, 991*m*, 970*s*, 928*m*, 903*m*, 869*w*, 824*s*, 755*m*, 719*s*, 690*m*, 639*m*, 590*s*, 565*s*, 555*vs*, 530*s*, 525*s*, 500*w*, 470*w*, 452*m*, 432*m*, 413*vw*, 511*w*, 483*w*, 458*w* cm^−1^; ^1^H NMR:δ = 7.82–7.75 (*m*, 2H, 2-H, 2′-H), 7.67–7.54 (*m*, 3H, 3-H, 3′-H, 4-H), 7.40 (*s*, 3H, NH, NH_2_), 4.00 (*t*, *J* = 6.5 Hz, 2H, 15-H), 2.72 (*t*, *J* = 6.9 Hz, 2H, 5-H), 1.67–1.57 (*m*, 2H, 14-H), 1.39–1.11 (*m*, 16H, 6-H, 7-H, 8-H, 9-H, 10-H, 11-H, 12-H, 13-H) ppm; ^13^C NMR:δ = 140.7 (C-1), 132.2 (C-4), 129.1 (C-3), 126.4 (C-2), 69.0 (C-15), 42.5 (C-5), 28.9 (C-6), 28.9 (C-9), 28.8 (C-10, C-11, C-13), 28.5 (C-8), 28.3 (C-14), 26.0 (C-12), 25.0 (C-7) ppm; MS: *m*/*z* = 429.3 (100%, [M + Na]^+^); anal. calcd. for C_17_H_30_N_2_S_2_O_5_ (406.56): C 50.22, H 7.44, N 6.89; found: C 49.96, H 7.69, N 6.58. 

#### 4.2.23. N-(12-Hydroxydodecyl)benzene Sulfonamide (**11a**)

Applying GPA: from benzenesulfonyl chloride (500 mg, 2.83 mmol) and 12-amino-dodecanol (855 mg, 4.25 mmol): **11a** (944 mg, 98%); white solid; R_f_ = 0.37 (petrolether/EtOAc, 2:3); m.p. = 76–77 °C; UV–Vis: 221 nm (3.95); IR: ν = 3412*m*, 3350*m*, 3278*s*, 2920*s*, 2848*m*, 1477*m*, 1464*m*, 1447*m*, 1425*w*, 1359*m*, 1320*s*, 1266*w*, 1154*vs*, 1095*s*, 1064*m*, 1048*s*, 1053*w*, 999*m*, 909*m*, 750*m*, 722*s*, 684*s*, 596*s*, 561*s*, 530*s*, 489*w*, 459*m* cm^−1^; ^1^H NMR:δ = 7.81–7.76 (*m*, 2H, 2-H, 2′-H), 7.66–7.56 (*m*, 3H, 3-H, 3′-H, 4-H), 7.53 (*t*, *J* = 5.8 Hz, 1H, NH), 4.30 (*t*, *J* = 5.1 Hz, 1H, OH), 3.37 (*td*, *J* = 6.5, 5.1 Hz, 2H, 16-H), 2.71 (*q*, *J* = 6.7 Hz, 2H, 5-H), 1.44–1.28 (*m*, 4H, 6-H, 15-H), 1.28–1.12 (*m*, 16H, 7-H, 8-H, 9-H, 10-H, 11-H, 12-H, 13-H, 14-H) ppm; ^13^C NMR (126 MHz, DMSO-*d*_6_): δ = 140.7 (C-1), 132.2 (C-4), 129.1 (C-3), 126.4 (C-2), 60.7 (C-16), 42.5 (C-5), 32.5 (C-15), 29.0 (C-12), 29.0 (C-11), 28.9 (C-9), 28.9 (C-10, C-13), 28.8 (C-6), 28.5 (C-8), 25.9 (C-7), 25.5 (C-14) ppm; MS: *m*/*z* = 364.3 (100%, [M + Na]^+^); anal. calcd. for C_18_H_31_NSO_3_ (341.51): C 63.31, H 9.15, N 4.10; found: C 63.00, H 9.43, N 3.97. 

#### 4.2.24. 12-(Phenylsulfonamido]dodecyl Sulfamate (**11b**)

Applying GPB: from **11a** (300 mg, 0.88 mmol): **11b** (229 mg, 62%); white solid; R_f_ = 0.77 (CHCl_3_/EtOAc, 2:3); m.p. = 102–103 °C; UV–Vis: λ_max_ (log ε) =; 221 nm (3.47); IR: ν = 3338*m*, 3265*m*, 2962*vw*, 2917*m*, 2849*m*, 1576*w*, 1470*w*, 1450*w*, 1428*w*, 1396*w*, 1381*s*, 1313*s*, 1289*w*, 1178*m*, 1158*vs*, 1094*m*, 1060*m*, 1043*m*, 1024*vw*, 996*w*, 983*m*, 964*s*, 931*m*, 905*m*, 880*m*, 854*w*, 838*m*, 821*s*, 794*w*, 768*w*, 753*s*, 726*s*, 688*s*, 589*s*, 567*s*, 550*s*, 535*vs*, 504*w*, 482*vw*, 433*m* cm^−1^; ^1^H NMR:δ = 7.79–7.74 (*m*, 2H, 2-H, 2′-H), 7.64–7.54 (*m*, 3H, 3-H, 3′-H, 4-H), 7.51 (*t*, *J* = 5.8 Hz, 1H, NH), 7.35 (*s*, 2H, NH_2_), 3.98 (*t*, *J* = 6.5 Hz, 2H, 16-H), 2.69 (*td*, *J* = 7.0, 5.8 Hz, 2H, 5-H), 1.64–1.55 (*m*, 2H, 15-H), 1.36–1.09 (*m*, 18H, 6-H, 7-H, 8-H, 9-H, 10-H, 11-H, 12-H, 13-H, 14-H) ppm; ^13^C NMR:δ = 140.7 (C-1), 132.2 (C-4), 129.1 (C-3), 126.4 (C-2), 69.0 (C-16), 42.5 (C-5), 28.9 (C-9), 28.9 (C-6, C-11, C-12), 28.9 (C-15), 28.5 (C-8), 28.3 (C-13), 26.0 (C-14), 25.1 (C-7) ppm; MS: *m*/*z* = 443.4 (100%, [M + Na]^+^); anal. calcd. for C_18_H_32_N_2_S_2_O_5_ (420.58): C 51.40, H 7.67, N 6.66; found: C 51.15, H 7.93, N 6.42.

#### 4.2.25. N-(2-Hydroxyethyl)-4-methylbenzene Sulfonamide (**12a**) [914083-49-1]

Applying GPA: from 4-methylbenzenesulfonyl chloride (300 mg, 1.57 mmol) and 2-amino-ethanol (144 mg, 2.36 mmol): **12a** [86,87,88,89,90,91,92] (315 mg, 93%); white solid; R_f_ = 0.54 (CHCl_3_/EtOAc, 2:3); m.p. = 55–57 °C; UV–Vis: 227 nm (3.68); IR: ν = 3497*br*, 3273*br*, 2926*w*, 2881*w*, 1598*w*, 1495*w*, 1424*m*, 1402*m*, 1318*s*, 1305*s*, 1290*m*, 1152*vs*, 1093*s*, 1055*s*, 1019*w*, 948*m*, 880*w*, 814*s*, 706*w*, 661*s*, 550*s*, 462*w* cm^−1^; ^1^H NMR:δ = 7.72–7.64 (*m*, 2H, 2-H, 2′-H), 7.48 (*s*, 1H, NH), 7.42–7.37 (*m*, 2H, 3-H, 3′-H), 4.65 (*s*, 1H, OH), 3.35 (*t*, *J* = 6.7 Hz, 2H, 7-H), 2.76 (*t*, *J* = 6.4 Hz, 2H, 6-H), 2.38 (*s*, 3H, 5-H) ppm; ^13^C NMR:δ = 142.5 (C-1), 137.7 (C-4), 129.6 (C-3), 126.5 (C-2), 59.9 (C-7), 45.0 (C-6), 20.9 (C-5) ppm; MS: *m*/*z* = 238 (80%, [M + Na]^+^); anal. calcd. for C_9_H_13_NSO_3_ (215.27): C 50.22, H 6.09, N 6.51; found: C 49.87, H 6.30, N 6.33.

#### 4.2.26. 2-[(4-Methylphenyl)sulfonamido]ethyl Sulfamate (**12b**) [914083-49-1]

Applying GPB: from **12a** (250 mg, 1.16 mmol): **12b** (330 mg, 96%) [93]; white solid; R_f_ = 0.49 (CHCl_3_/EtOAc, 2:3); m.p. = 79–81 °C; UV–Vis: 224 nm (3.91); IR: ν = 3358*w*, 3332*w*, 3265*m*, 1480*w*, 1448*w*, 1425*w*, 1377*m*, 1366*s*, 1322*s*, 1305*m*, 1238*w*, 1227*w*, 1178*s*, 1160*s*, 1148*s*, 1117*w*, 1098*m*, 1083*s*, 1033*m*, 1011*s*, 954*m*, 931*s*, 913*m*, 880*w*, 869*w*, 818*m*, 803*m*, 783*s*, 769*s*, 702*m*, 684*s*, 659*m*, 598*m*, 589*m*, 574*s*, 548*vs*, 529*m*, 509*m*, 487*m*, 471*s*, 448*m*, 431*w*, 411*w* cm^−1^; ^1^H NMR:δ = 7.82 (s, 1H, NH), 7.72–7.65 (*m*, 2H, 2-H, 2′-H), 7.58–7.46 (*m*, 2H, NH_2_), 7.44–7.37 (*m*, 2H, 3-H, 3′-H), 3.99 (*t*, *J* = 5.8 Hz, 2H, 7-H), 3.02 (*t*, *J* = 5.8 Hz, 2H, 6-H), 2.39 (*s*, 3H, 5-H) ppm; ^13^C NMR:δ = 142.8 (C-4), 137.3 (C-1), 129.7 (C-3), 126.5 (C-2), 67.5 (C-7), 41.5 (C-6), 21.0 (C-5) ppm; MS: *m*/*z* = 317.1 (100%, [M + Na]^+^); anal. calcd. for C_9_H_14_N_2_S_2_O_5_ (294.34): C 36.73, H 4.79, N 9.52; found: C 36.41, H 4.97, N 9.35. 

#### 4.2.27. N-(3-Hydroxypropyl)-4-methylbenzene Sulfonamide (**13a**) [13379-98-1]

Applying GPA: from 4-methylbenzenesulfonyl chloride (200 mg, 1.05 mmol) and 3-amino-propanol (118 mg, 1.57 mmol): **13a** (225 mg, 94%) [94,95,96,97,98,99]; white solid; R_f_ = 0.12 (petrolether/EtOAc, 2:3); m.p. = 55–57 °C (lit.: [98] 55–56 °C); UV–Vis: 227 nm (4.14); IR: ν = 3492*br*, 3274*br*, 2945*w*, 2879*w*, 1598*m*, 1495*w*, 1423*m*, 1318*s*, 1305*s*, 1296*m*, 1185*w*, 1153*vs*, 1091*s*, 1070*s*, 1019*w*, 1006*w*, 959*m*, 872*w*, 815*s*, 706*w*, 662*s*, 570*w*, 550*s*, 515*w* cm^−1^; ^1^H NMR:δ =7.69–7.64 (*m*, 2H, 2-H, 2′-H), 7.46–7.35 (*m*, 3H, 3-H, 3′-H, NH), 4.39 (*t*, *J* = 5.1 Hz, 1H, OH), 3.40–3.30 (*m*, 2H, 8-H), 2.76 (*td*, *J* = 7.6, 3.3 Hz, 2H, 6-H), 2.38 (*s*, 3H, 5-H), 1.50 (*p*, *J* = 6.4 Hz, 2H, 7-H) ppm; ^13^C NMR:δ = 142.5 (C-4), 137.6 (C-1), 129.6 (C-3), 126.5 (C-2), 58.1 (C-8), 40.0 (C-6), 32.3 (C-7), 20.9 (C-5) ppm; MS: *m*/*z* = 252.3 (90%, [M + Na]^+^); anal. calcd. for C_10_H_15_NSO_3_ (229.29): C 52.38, H 6.59, N 6.11; found: C 52.09, H 6.89, N 5.87.

#### 4.2.28. 3-[(4-Methylphenyl)sulfonamido]propyl Sulfamate (**13b**)

Applying GPB: from **13a** (300 mg, 1.31 mmol): **13b** (260 mg, 64%); white solid; R_f_ = 0.49 (CHCl_3_/EtOAc, 2:3); m.p. = 86–87 °C; UV–Vis: 227 nm (3.72); IR: ν = 3352*w*, 3317*w*, 3272*w*, 2959*vw*, 2902*vw*, 1597*vw*, 1554*w*, 1472*w*, 1434*vw*, 1409*w*, 1392*w*, 1362*m*, 1321*m*, 1309*m*, 1292*w*, 1239*vw*, 1211*vw*, 1181*m*, 1160*s*, 1122*w*, 1093*m*, 1047*m*, 1020*vw*, 980*w*, 956*s*, 919*m*, 893*w*, 854*m*, 841*m*, 818*m*, 799*w*, 773*m*, 706*w*, 667*s*, 590*m*, 573*m*, 547*vs*, 494*m*, 476*m*, 444*w*, 413*vw*, 511*w*, 483*w*, 458*w* cm^−1^; ^1^H NMR:δ = 7.71–7.64 (*m*, 2H, 2-H, 2′-H), 7.57–7.35 (*m*, 5H, 3-H, 3′-H, NH, NH_2_), 4.02 (*t*, *J* = 6.3 Hz, 2H, 8-H), 2.79 (*t*, *J* = 7.1 Hz, 2H, 6-H), 2.39 (*s*, 3H, 5-H), 1.75 (*p*, *J* = 6.7 Hz, 2H, 7-H) ppm; ^13^C NMR (126 MHz, DMSO-*d*_6_): δ = 142.7 (C-4), 137.4 (C-1), 129.7 (C-3), 126.5 (C-2), 66.5 (C-8), 39.2 (C-6), 28.7 (C-7), 20.9 (C-5) ppm; MS (ESI, MeOH) *m*/*z* = 331.2 (90%, [M + Na]^+^); anal. calcd. for C_10_H_16_N_2_S_2_O_5_ (308.37): C 38.95, H 5.23, N 9.08; found: C 38.77, H 5.54, N 8.78. 

#### 4.2.29. N-(4-Hydroxybutyl)-4-methylbenzene Sulfonamide (**14a**) [78521-69-4]

Applying GPA: from 4-methylbenzenesulfonyl chloride (400 mg, 2.1 mmol) and 4-amino-butanol (318 mg, 3.15 mmol): **14a** (477 mg, 93%); white solid; R_f_ = 0.12 (petrolether/EtOAc, 2:3); m.p. = 50–51 °C (lit.: [100] 50–52 °C); UV–Vis: 227 nm (4.18); IR: ν = 3502*br*, 3279*br*, 2940*w*, 2871*w*, 1597*m*, 1475*m*, 1454*m*, 1433*m*, 1314*s*, 1305*s*, 1289*m*, 1150*vs*, 1121*w*, 1091*s*, 1061*s*, 1027*m*, 1019*w*, 941*m*, 848*w*, 817*s*, 753*m*, 721*m*, 709*m*, 662*s*, 576*s*, 551*s*, 484*m*, 456*w* cm^−1^; ^1^H NMR (500 MHz, DMSO-*d*_6_): δ = 7.69–7.65 (*m*, 2H, 2-H, 2′-H), 7.48–7.43 (*m*, 1H, NH), 7.40–7.36 (*m*, 2H, 3-H, 3′-H), 4.36 (*t*, *J* = 5.1 Hz, 1H, OH), 3.32 (*q*, *J* = 5.5 Hz, 2H, 9-H), 2.73–2.67 (*m*, 2H, 6-H), 2.37 (*s*, 3H, 5-H), 1.43–1.32 (*m*, 4H, 7-H, 8-H) ppm; ^13^C NMR (126 MHz, DMSO-*d*_6_): δ = 142.4 (C-4), 137.7 (C-1), 129.6 (C-3), 126.5 (C-2), 60.2 (C-9), 42.5 (C-6), 29.6 (C-8), 25.8 (C-7), 20.9 (C-5) ppm; MS: *m*/*z* = 266.1 (100%, [M + Na]^+^); anal. calcd. for C_11_H_17_NSO_3_ (243.32): C 54.30, H 7.04, N 5.76; found: C 54.11, H 7.32, N 5.55. 

#### 4.2.30. 4-[(4-Methylphenyl)sulfonamido]butyl Sulfamate (**14b**)

Applying GPB: from **14a** (100 mg, 0.41 mmol): **14b** (90 mg, 68%); white solid; R_f_ = 0.52 (CHCl_3_/EtOAc, 2:3); m.p. = 66–68 °C; UV–Vis: 227 nm (3.81); IR: ν = 3354*w*, 3314*w*, 3275*w*, 2955*vw*, 2902*vw*, 1597*vw*, 1552*w*, 1474*w*, 1434*vw*, 1410*w*, 1398*w*, 1362*m*, 1321*m*, 1309*m*, 1294*w*, 1235*vw*, 1211*vw*, 1180*m*, 1162*s*, 1120*w*, 1098*m*, 1046*m*, 1020*vw*, 982*w*, 957*s*, 917*m*, 894*w*, 854*m*, 840*m*, 820*m*, 799*w*, 774*m*, 704*w*, 665*s*, 596*m*, 571*m*, 547*vs*, 495*m*, 473*m*, 444*w*, 413*vw*, 511*w* cm^−1^; ^1^H NMR:δ = 7.70–7.65 (*m*, 2H, 2-H, 2′-H), 7.52 (*t*, *J* = 5.9 Hz, 1H, NH), 7.45–7.36 (*m*, 4H, 3-H, 3′-H, NH_2_), 3.96 (*t*, *J* = 6.3 Hz, 2H, 9-H), 2.73 (*q*, *J* = 6.8 Hz, 2H, 6-H), 2.38 (*s*, 3H, 5-H), 1.65–1.55 (*m*, 2H, 8-H), 1.49–1.39 (*m*, 2H, 7-H) ppm; ^13^C NMR:δ = 142.6 (C-4), 137.7 (C-1), 129.6 (C-3), 126.5 (C-2, C-2), 68.6 (C-9), 42.0 (C-6), 25.6 (C-8), 25.4 (C-7), 21.0 (C-5) ppm; MS: *m*/*z* = 345.1 (100%, [M + Na]^+^); anal. calcd. for C_11_H_18_N_2_S_2_O_5_ (322.39): C 40.98, H 5.63, N 8.69; found: C 40.78, H 5.89, N 8.43. 

#### 4.2.31. N-(5-Hydroxypentyl)-4-methylbenzene Sulfonamide (**15a**) [16780-44-2]

Applying GPA: from 4-methylbenzenesulfonyl chloride (500 mg, 2.62 mmol) and and 5-amino-pentanol (405 mg, 3.93 mmol): **15a** (522 mg, 77%) [101,102,103,104,105,106,107,108]; white solid; R_f_ = 0.11 (petrolether/EtOAc, 2:3); m.p. = 60–61 °C; UV–Vis: 227 nm (3.99); IR: ν = 3456*m*, 3109*br*, 2920*w*, 2862*w*, 1597*m*, 1475*m*, 1454*m*, 1433*m*, 1314*s*, 1305*s*, 1289*m*, 1150*vs*, 1121*w*, 1091*s*, 1061*s*, 1027*m*, 1019*w*, 941*m*, 848*w*, 817*s*, 753*m*, 721*m*, 709*m*, 662*s*, 576*s*, 551*s*, 484*m*, 456*w* cm^−1^; ^1^H NMR:δ = 7.69–7.63 (*m*, 2H, 2-H, 2′-H), 7.44 (*t*, *J* = 5.9 Hz, 1H, NH), 7.41–7.36 (*m*, 2H, 3-H, 3′-H), 4.30 (*t*, *J* = 5.1 Hz, 1H, OH), 3.35–3.29 (*m*, 2H, 10-H), 2.68 (*td*, *J* = 7.0, 5.9 Hz, 2H, 6-H), 2.38 (*s*, 3H, 5-H), 1.39–1.27 (*m*, 4H, 7-H, 9-H), 1.27–1.17 (*m*, 2H, 8-H) ppm; ^13^C NMR:δ = 142.4 (C-4), 137.7 (C-1), 129.5 (C-3), 126.5 (C-2), 60.5 (C-10), 42.5 (C-6), 32.0 (C-9), 28.8 (C-7), 22.6 (C-8), 20.9 (C-5) ppm; MS: *m*/*z* = 280.0 (100%, [M + Na]^+^); anal. calcd. for C_12_H_19_NSO_3_ (257.35): C 56.01, H 7.44, N 5.44; found: C 55.76, H 7.78, N 5.16.

#### 4.2.32. 5-[(4-Methylphenyl)sulfonamido]pentyl Sulfamate (**15b**)

Applying GPB: from **15a** (200 mg, 0.78 mmol): **15b** (232 mg, 89%); white solid; R_f_ = 0.53 (CHCl_3_/EtOAc, 2:3); m.p. = 94–95 °C UV–Vis: 227 nm (3.92); IR: ν = 3351*w*, 3301*m*, 3250*w*, 2957*vw*, 2940*w*, 2930*w*, 2852*w*, 1554*w*, 1466*w*, 1422*w*, 1395*vw*, 1361*s*, 1316*s*, 1301*m*, 1294*w*, 1281*w*, 1177*s*, 1150*vs*, 1096*m*, 1074*w*, 1063*w*, 1044*w*, 1027*vw*, 1021*w*, 992*m*, 965*vw*, 947*m*, 915*s*, 870*w*, 815*s*, 810*s*, 749*w*, 722*w*, 708*w*, 671*s*, 630*w*, 598*m*, 574*m*, 550*vs* cm^−1^; ^1^H NMR:δ = 7.70–7.63 (*m*, 2H, 2-H, 2′-H), 7.47 (*t*, *J* = 5.9 Hz, 1H, NH), 7.42–7.34 (*m*, 4H, 3-H, 3′-H, NH_2_), 3.96 (*t*, *J* = 6.4 Hz, 2H, 10-H), 2.70 (*q*, *J* = 6.6 Hz, 2H, 6-H), 2.38 (*s*, 3H, 5-H), 1.55 (*p*, *J* = 6.7 Hz, 2H, 9-H), 1.43–1.33 (*m*, 2H, 7-H), 1.33–1.22 (*m*, 2H, 8-H) ppm; ^13^C NMR:δ = 142.5 (C-4), 137.7 (C-1), 129.6 (C-3), 126.5 (C-2), 68.8 (C-10), 42.3 (C-6), 28.5 (C-9), 27.8 (C-7), 22.2 (C-8), 20.9 (C-5) ppm; MS: *m*/*z* = 359.4 (100%, [M + Na]^+^); anal. calcd. for C_12_H_20_N_2_S_2_O_5_ (336.42): C 42.84, H 5.99, N 8.33; found: C 42.57, H 6.26, N 8.14.

#### 4.2.33. N-(6-Hydroxyhexyl)-4-methylbenzene Sulfonamide (**16a**) [385369-83-5]

Applying GPA: from 4-methylbenzenesulfonyl chloride (500 mg, 2.62 mmol) and 6-amino-hexanol (461 mg, 3.93 mmol): **16a** (551 mg, 77%) [104,109,110,111,112]; white solid; R_f_ = 0.15 (petrolether/EtOAc, 2:3); m.p. = 49–51 °C; UV–Vis: 227 nm (4.10); IR: ν = 3423*w*, 3364*w*, 3290*m*, 2936*m*, 2891*w*, 2860*w*, 1589*w*, 1495*w*, 1476*w*, 1422*m*, 1385*w*, 1319*m*, 1303*w*, 1290*w*, 1154*vs*, 1091*m*, 1067*m*, 1036*m*, 983*w*, 905*m*, 817*s*, 734*w*, 707*w*, 666*s*, 573*s*, 549*s*, 523*m*, 484*w*, 430*w* cm^−1^; ^1^H NMR:δ = 7.70–7.63 (*m*, 2H, 2-H, 2′-H), 7.47–7.41 (*m*, 1H, NH), 7.41–7.35 (*m*, 2H, 3-H, 3′-H), 4.30 (*t*, *J* = 5.1 Hz, 1H, OH), 3.38–3.30 (*m*, 2H, 11-H), 2.73–2.65 (*m*, 2H, 6-H), 2.37 (*s*, 3H, 5-H), 1.41–1.27 (*m*, 4H, 7-H, 10-H), 1.22–1.14 (*m*, 4H, 8-H, 9-H) ppm; ^13^C NMR:δ = 142.4 (C-4), 137.8 (C-1), 129.6 (C-3, C-3′), 126.5 (C-2, C-2′), 60.6 (C-11), 42.5 (C-6), 32.4 (C-10), 29.0 (C-7), 25.9 (C-9), 25.0 (C-8), 20.9 (C-5) ppm; MS: *m*/*z* = 294 (100%, [M + Na]^+^); anal. calcd. for C_13_H_21_NSO_3_ (271.38): C 57.54, H 7.80, N 5.16; found: C 57.24, H 8.02, N 4.96.

#### 4.2.34. 6-[(4-Methylphenyl)sulfonamido]hexyl Sulfamate (**16b**)

Applying GPB: from **16a** (200 mg, 0.74 mmol): **16b** (206 mg, 80%); white solid; R_f_ = 0.55 (CHCl_3_/EtOAc, 2:3); m.p. = 49–50 °C; UV–Vis: 227 nm (4.03); IR: ν = 3350*w*, 3300*m*, 3251*w*, 2959*vw*, 2939*w*, 2932*w*, 2854*w*, 1556*w*, 1464*w*, 1422*w*, 1391*vw*, 1363*s*, 1317*s*, 1308*m*, 1291*w*, 1280*w*, 1178*s*, 1151*vs*, 1094*m*, 1075*w*, 1062*w*, 1043*w*, 1027*vw*, 1020*w*, 992*m*, 968*vw*, 945*m*, 917*s*, 868*w*, 817*s*, 810*s*, 749*w*, 722*w*, 708*w*, 670*s*, 634*w*, 595*m*, 576*m*, 549*vs*, 513*m*, 487*m*, 473*m* cm^−1^; ^1^H NMR (500 MHz, DMSO-*d*_6_): δ = 7.69–7.64 (*m*, 2H, 2-H, 2′-H), 7.46 (*t*, *J* = 5.8 Hz, 1H, NH), 7.41–7.35 (*m*, 4H, 3-H, 3′-H, NH_2_), 3.97 (*t*, *J* = 6.5 Hz, 2H, 11-H), 2.70 (*q*, *J* = 6.6 Hz, 2H, 6-H), 2.38 (*s*, 3H, 5-H), 1.59–1.52 (*m*, 2H, 10-H), 1.35 (*p*, *J* = 6.9 Hz, 2H, 7-H), 1.27–1.17 (*m*, 4H, 8-H, 9-H) ppm; ^13^C NMR:δ = 142.5 (C-4), 137.7 (C-1), 129.6 (C-3), 126.5 (C-2), 68.9 (C-11), 42.4 (C-6), 28.8 (C-10), 28.2 (C-7), 25.5 (C-9), 24.6 (C-8), 20.9 (C-5) ppm; MS: *m*/*z* = 373.7 (100%, [M + Na]^+^); anal. calcd. for C_13_H_22_N_2_S_2_O_5_ (350.45): C 44.56, H 6.33, N 7.99; found: C 44.34, H 6.51, N 7.65. 

#### 4.2.35. N-(7-Hydroxyheptyl)-4-methylbenzene Sulfonamide (**17a**) [1669425-24-4]

Applying GPA: from 4-methylbenzenesulfonyl chloride (500 mg, 2.62 mmol) and 7-amino-heptanol (516 mg, 3.93 mmol): **17a** (557 mg, 74%) [113] oil; R_f_ = 0.19 (petrolether/EtOAc, 2:3); UV–Vis: 227 nm (4.15); IR: ν = 3503*w*, 3279*w*, 2930*m*, 2858*w*, 1598*w*, 1495*vw*, 1429*w*, 1320*m*, 1305*m*, 1289*m*, 1152*vs*, 1120*w*, 1092*s*, 1056*m*, 1019*w*, 814*m*, 723*w*, 707*m*, 660*s*, 635*w*, 571*m*, 549*vs*, 466*w* cm^−1^; ^1^H NMR:δ = 7.68–7.65 (*m*, 2H, 2-H, 2′-H), 7.44 (*t*, *J* = 5.8 Hz, 1H, NH), 7.40–7.36 (*m*, 2H, 3-H, 3′-H), 4.30 (*t*, *J* = 5.1 Hz, 1H, OH), 3.37–3.34 (*m*, 2H, 12-H), 2.69 (*td*, *J* = 7.0, 5.7 Hz, 2H, 6-H), 2.37 (*s*, 3H, 5-H), 1.40–1.29 (*m*, 4H, 7-H, 11-H), 1.25–1.10 (*m*, 6H, 8-H, 9-H, 10-H) ppm; ^13^C NMR:δ = 142.4 (C-4), 137.8 (C-1), 129.5 (C-3), 126.5 (C-2), 60.7 (C-12), 42.5 (C-6), 32.4 (C-11), 28.9 (C-7), 28.4 (C-9), 26.1 (C-8), 25.3 (C-10), 20.9 (C-5) ppm; MS: *m*/*z* = 308.2 (100%, [M + Na]^+^); anal. calcd. for C_14_H_23_NSO_3_ (285.40): C 58.92, H 8.12, N 4.91; found: C 58.69, H 8.33, N 4.65. 

#### 4.2.36. 7-[(4-Methylphenyl)sulfonamido]heptyl Sulfamate (**17b**)

Applying GPB: from **17a** (200 mg, 0.7 mmol): **17b** (94 mg, 37%); white solid; R_f_ = 0.60 (CHCl_3_/EtOAc, 2:3); m.p. = 85–86 °C; UV–Vis: 227 nm (4.07); IR: ν = 3351*w*, 3298*m*, 3252*w*, 2960*w*, 2940*w*, 2921*w*, 2853*w*, 1598*vw*, 1558*w*, 1477*vw*, 1466*w*, 1457*w*, 1439*w*, 1420*w*, 1392*w*, 1362*s*, 1319*s*, 1308*m*, 1292*w*, 1281*w*, 1180*s*, 1150*vs*, 1137*m*, 1094*m*, 1080*m*, 1048*w*, 1020*w*, 1009*w*, 996*w*, 982*vw*, 950*m*, 930*s*, 904*s*, 834*w*, 818*s*, 798*w*, 782*m*, 737*w*, 723*w*, 707*w*, 668*s*, 597*m*, 577*m*, 548*vs*, 519*m*, 495*m*, 474*m*, 448*w*, 404*vw* cm^−1^; ^1^H NMR:δ = 7.69–7.64 (*m*, 2H, 2-H, 2′-H), 7.45 (*t*, *J* = 5.8 Hz, 1H, NH), 7.41–7.35 (*m*, 4H, 3-H, 3′-H, NH_2_), 3.98 (*t*, *J* = 6.5 Hz, 2H, 12-H), 2.69 (*q*, *J* = 6.7 Hz, 2H, 6-H), 2.38 (*s*, 3H, 5-H), 1.62–1.54 (*m*, 2H, 11-H), 1.38–1.30 (*m*, 2H, 7-H), 1.30–1.14 (*m*, 6H, 8-H, 9-H, 10-H) ppm; ^13^C NMR:δ = 142.4 (C-4), 137.8 (C-1), 129.5 (C-3), 126.5 (C-2), 68.9 (C-12), 42.4 (C-6), 28.8 (C-11), 28.2 (C-7), 28.0 (C-9), 25.9 (C-10), 24.9 (C-8), 20.9 (C-5) ppm; MS: *m*/*z* = 387.3 (100%, [M + Na]^+^); anal. calcd. for C_14_H_24_N_2_S_2_O_5_ (364.48): C 46.14, H 6.64, N 7.69; found: C 45.87, H 6.92, N 7.46.

#### 4.2.37. N-(8-Hydroxyoctyl)-4-methylbenzene Sulfonamide (**18a**) [2772203-84-4]

Applying GPA: from 4-methylbenzenesulfonyl chloride (400 mg, 2.1 mmol) and 8-amino-octanol (457 mg, 3.15 mmol): **18a** (442 mg, 70%); white solid; R_f_ = 0.23 (petrolether/EtOAc, 2:3); m.p. = 87–88 °C; UV–Vis: 227 nm (4.00); IR: ν = 3418*w*, 3278*m*, 2933*m*, 2854*m*, 1598*w*, 1478*w*, 1466*w*, 1425*m*, 1384*w*, 1364*w*, 1333*m*, 1324*m*, 1305*m*, 1290*w*, 1157*vs*, 1109*w*, 1092*m*, 1064*m*, 1052*s*, 1031*w*, 1020*w*, 992*w*, 982*m*, 905*m*, 817*s*, 733*w*, 707*w*, 668*s*, 571*s*, 551*vs*, 530*s*, 500*m*, 493*m*, 465*w* cm^−1^; ^1^H NMR:δ = 7.69–7.65 (*m*, 2H, -H, 2′-H), 7.44 (*t*, *J* = 5.8 Hz, 1H, NH), 7.41–7.36 (*m*, 2H, 3-H, 3′-H), 4.31 (*t*, *J* = 5.2 Hz, 1H, OH), 3.40–3.34 (*m*, 2H, 13-H), 2.69 (*q*, *J* = 6.8 Hz, 2H, 6-H), 2.38 (*s*, 3H, 5-H), 1.42–1.28 (*m*, 4H, 7-H, 12-H), 1.26–1.12 (*m*, 8H, 8-H, 9-H, 10-H, 11-H) ppm; ^13^C NMR:δ = 142.9 (C-4), 138.3 (C-1), 130.0 (C-3), 127.0 (C-2), 61.2 (C-13), 43.0 (C-6), 33.0 (C-12), 29.4 (C-7), 29.3 (C-9), 29.0 (C-10), 26.4 (C-8), 25.9 (C-11), 21.4 (C-5).ppm; MS: *m*/*z* = 322 (100%, [M + Na]^+^); anal. calcd. for C_15_H_25_NSO_3_ (299.43): C 60.17, H 8.42, N 4.68; found: C 59.87, H 8.69, N 4.55. 

#### 4.2.38. 8-[(4-Methylphenyl)sulfonamido]octyl Sulfamate (**18b**)

Applying GPB: from **18a** (200 mg, 0.67 mmol): **18b** (140 mg, 55%); white solid; R_f_ = 0.64 (CHCl_3_/EtOAc, 2:3); m.p. = 98–100 °C; UV–Vis: 227 nm (4.00); IR: ν = 3344*m*, 3311*m*, 3250*m*, 2944*w*, 2928*w*, 2857*w*, 2844*w*, 1476*w*, 1430*m*, 1362*s*, 1332*w*, 1319*m*, 1309*m*, 1183*m*, 1147*vs*, 1119*w*, 1095*m*, 1074*w*, 1065*w*, 1054*m*, 1040*w*, 963*s*, 927*s*, 902*m*, 818*s*, 724*w*, 707*w*, 666*s*, 591*m*, 561*s*, 550*vs*, 526*m*, 511*s*, 499*s*, 474*m*, 405*w* cm^−1^; ^1^H NMR:δ = 7.68–7.63 (*m*, 2H, 2-H, 2′-H), 7.44 (*t*, *J* = 5.9 Hz, 1H, NH), 7.41–7.35 (*m*, 4H, 3-H, 3′-H. NH_2_), 3.99 (*t*, *J* = 6.5 Hz, 2H, 13-H), 2.69 (*td*, *J* = 7.0, 5.8 Hz, 2H, 6-H), 2.38 (s, 3H, 5-H), 1.64–1.55 (*m*, 2H, 12-H), 1.38–1.31 (*m*, 2H, 7-H), 1.30–1.12 (*m*, 8H, 8-H, 9-H, 10-H, 11-H) ppm; ^13^C NMR:δ = 142.4 (C-4), 137.8 (C-1), 129.5 (C-3), 126.5 (C-2), 69.0 (C-13), 42.4 (C-6), 28.9 (C-12), 28.4 (C-7), 28.3 (C-10), 28.3 (C-9), 25.9 (C-8), 24.9 (C-11), 20.9 (C-5) ppm; MS: *m*/*z* = 401.7 (100%, [M + Na]^+^); anal. calcd. for C_15_H_26_N_2_S_2_O_5_ (378.50): C 47.60, H 6.92, N 7.40; found: C 47.37, H 7.19, N 7.38. 

#### 4.2.39. N-(2-Hydroxyethyl)-3-methylbenzene Sulfonamide (**19a**) [1082883-27-9]

Applying GPA: from 3-methylbenzenesulfonyl chloride (500 mg, 2.62 mmol) and 2-amino-ethanol (240 mg, 3.93 mmol): **19a** (542 mg, 96%); oil; R_f_ = 0.1 (petrolether/EtOAc, 2:3); UV–Vis: 224 nm (3.92); IR: ν = 3493*w*, 3274*w*, 2927*w*, 2882*w*, 1601*vw*, 1478*w*, 1425*w*, 1321*s*, 1303*s*, 1220*w*, 1148*vs*, 1097*m*, 1087*m*, 1054*s*, 999*w*, 949*m*, 866*w*, 785*m*, 687*s*, 580*vs*, 524*m*, 492*w*, 459*m*, 435*w* cm^−1^; ^1^H NMR:δ = 7.63–7.61 (*m*, 1H, 6-H), 7.61–7.58 (*m*, 1H, 2-H), 7.52 (*t*, *J* = 5.8 Hz, 1H, NH), 7.50–7.42 (*m*, 2H, 4-H, 5-H), 4.66 (*t*, *J* = 5.6 Hz, 1H, OH), 3.37 (*q*, *J* = 6.3 Hz, 2H, 9-H), 2.79 (*q*, *J* = 6.2 Hz, 2H, 8-H), 2.39 (*s*, 3H, 7-H) ppm; ^13^C NMR:δ = 140.5 (C-1), 138.8 (C-3), 132.9 (C-4), 129.0 (C-5), 126.7 (C-6), 123.6 (C-2), 59.9 (C-9), 45.1 (C-8), 20.8 (C-7) ppm; MS: *m*/*z* = 238.4 (40%, [M + Na]^+^); anal. calcd. for C_9_H_13_NSO_3_ (215.27): C 50.22, H 6.09, N 6.51; found: C 49.97, H 6.34, N 6.27.

#### 4.2.40. 2-[(3-Methylphenyl)sulfonamido]ethyl Sulfamate (**19b**)

Applying GPB: from **19a** (73 mg, 0.4 mmol): **19b** (72 mg, 72%); white solid; R_f_ = 0.52 (CHCl_3_/EtOAc, 2:3); m.p. = 36–38 °C; UV–Vis: 224 nm (3.65); IR: ν = 3341*m*, 3259*m*, 3100*w*, 2988*w*, 1566*w*, 1473*w*, 1444*w*, 1421*w*, 1389*w*, 1371*vs*, 1345*m*, 1325*m*, 1303*s*, 1226*w*, 1174*s*, 1172*s*, 1147*vs*, 1078*m*, 1062*m*, 1001*m*, 951*s*, 932*s*, 905*m*, 879*m*, 865*w*, 837*s*, 791*m*, 765*m*, 701*s*, 683*s*, 639*s*, 591*s*, 571*s*, 546*vs*, 504*m*, 452*m*, 429*w*, 544*vs*, 526*m* cm^−1^; ^1^H NMR:δ = 7.86 (*t*, *J* = 5.9 Hz, 1H, NH), 7.64–7.57 (*m*, 2H, 2-H, 6-H), 7.53–7.44 (*m*, 4H, 4-H, 5-H, NH_2_), 3.99 (*t*, *J* = 5.7 Hz, 2H, 9-H), 3.04 (*q*, *J* = 5.6 Hz, 2H, 8-H), 2.39 (*s*, 3H, 7-H) ppm; ^13^C NMR:δ = 140.1 (C-1), 139.0 (C-2), 133.2 (C-4), 129.1 (C-5), 126.7 (C-6), 123.6 (C-3), 67.5 (C-9), 41.6 (C-8), 20.9 (C-7) ppm; MS: *m*/*z* = 317.1 (100%, [M + Na]^+^); anal. calcd. for C_9_H_14_N_2_S_2_O_5_ (294.34): C 36.73, H 4.79, N 9.52; found: C 36.55, H 5.00, N 9.35.

#### 4.2.41. N-(3-Hydroxypropyl)-3-methylbenzene Sulfonamide (**20a**) [1082805-58-0]

Applying GPA: from 3-methylbenzenesulfonyl chloride (500 mg, 2.62 mmol) and 3-amino-propanol (295 mg, 3.93 mmol): **20a** (522 mg, 87%) [114] oil; R_f_ = 0.09 (petrolether/EtOAc, 2:3); UV–Vis: 224 nm (3.91); IR: ν = 3502*w*, 3276*w*, 2947*w*, 2882*w*, 1477*w*, 1423*w*, 1320*m*, 1303*s*, 1222*w*, 1148*vs*, 1096*m*, 1085*s*, 1068*m*, 1008*w*, 998*w*, 959*w*, 879*w*, 787*m*, 688*s*, 579*vs*, 524*m*, 498*w*, 463*m*, 434*w* cm^−1^; ^1^H NMR:δ = 7.62–7.56 (*m*, 2H, 2-H, 6-H), 7.50–7.41 (*m*, 3H, 4-H, 5-H, NH), 4.40 (*t*, *J* = 5.1 Hz, 1H, OH), 3.40–3.35 (*m*, 2H, 10-H), 2.78 (*q*, *J* = 7.0 Hz, 2H, 8-H), 2.39 (*s*, 3H, 7-H), 1.52 (*p*, *J* = 6.4 Hz, 2H, 9-H) ppm; ^13^C NMR:δ = 140.4 (C-1), 138.8 (C-3), 132.9 (C-4), 129.0 (C-5), 126.7 (C-6), 123.6 (C-2), 58.1 (C-10), 40.0 (C-8), 32.4 (C-9), 20.9 (C-7) ppm; MS: *m*/*z* = 252.1 (100%, [M + Na]^+^); anal. calcd. for C_10_H_15_NSO_3_ (229.29): C 52.38, H 6.59, N 6.11; found: C 52.03, H 6.88, N 5.94.

#### 4.2.42. 3-[(3-Methylphenyl)sulfonamido]propyl Sulfamate (**20b**)

Applying GPB: from **20a** (300 mg, 1.31 mmol): **20b** (305 mg, 76%); white solid; R_f_ = 0.52 (CHCl_3_/EtOAc, 2:3); m.p. = 73–74 °C; UV–Vis: 224 nm (3.88); IR: ν = 3351*m*, 3256*m*, 3108*w*, 2983*w*, 2921*vw*, 1566*w*, 1473*w*, 1444*w*, 1421*w*, 1399*w*, 1373*vs*, 1352*m*, 1318*m*, 1303*s*, 1241*w*, 1226*w*, 1218*w*, 1176*s*, 1170*s*, 1147*vs*, 1113*w*, 1085*m*, 1056*m*, 1004*m*, 951*s*, 929*s*, 905*m*, 887*m*, 881*m*, 865*w*, 837*s*, 792*m*, 759*m*, 703*s*, 685*s*, 638*s*, 591*s*, 573*s*, 545*vs*, 508*m*, 486*m*, 450*m*, 426*w*, 548*vs*, 519*m*, 495*m*, 474*m*, 448*w*, 404*vw* cm^−1^; ^1^H NMR:δ = 7.67–7.57 (*m*, 3H, 2-H, 6-H, NH), 7.52–7.37 (*m*, 4H, 4-H, 5-H, NH_2_), 4.02 (*t*, *J* = 6.3 Hz, 2H, 10-H), 2.81 (*t*, *J* = 7.1 Hz, 2H, 8-H), 2.40 (*s*, 3H, 7-H), 1.76 (*p*, *J* = 6.7 Hz, 2H, 9-H) ppm; ^13^C NMR:δ = 140.2 (C-1), 138.9 (C-3), 133.0 (C-4), 129.1 (C-5), 126.7 (C-6), 123.6 (C-2), 66.5 (C-10), 39.2 (C-8), 28.7 (C-9), 20.8 (C-7) ppm; MS: *m*/*z* = 331.3 (100%, [M + Na]^+^); anal. calcd. for C_10_H_16_N_2_S_2_O_5_ (308.37): C 38.95, H 5.23, N 9.08; found: C 38.77, H 5.52, N 8.85.

#### 4.2.43. N-(4-Hydroxybutyl)-3-methylbenzene Sulfonamide (**21a**) [1082889-69-7]

Applying GPA: from 3-methylbenzenesulfonyl chloride (500 mg, 2.62 mmol) and 4-amino-butanol (351 mg, 3.93 mmol): **21a** (589 mg, 92%); oil; R_f_ = 0.56 (CHCl_3_/EtOAc, 2:3); UV–Vis: 224 nm (3.90); IR: ν = 3502*w*, 3279*w*, 2940*w*, 2871*w*, 1598*w*, 1428*w*, 1319*m*, 1305*m*, 1289*m*, 1152*vs*, 1120*w*, 1091*s*, 1054*m*, 1020*m*, 814*m*, 706*w*, 659*s*, 571*m*, 549*vs*, 491*w*, 469*w* cm^−1^; ^1^H NMR:δ = 7.62–7.56 (*m*, 2H, 2-H, 6-H), 7.52–7.41 (*m*, 3H, 4-H, 5-H, NH), 4.35 (*t*, *J* = 5.1 Hz, 1H, OH), 3.35–3.29 (*m*, 2H, 11-H), 2.72 (*q*, *J* = 6.7 Hz, 2H, 8-H), 2.39 (*s*, 3H, 7-H), 1.45–1.31 (*m*, 4H, 9-H, 10-H) ppm; ^13^C NMR:δ = 140.5 (C-1), 138.8 (C-3), 132.8 (C-4), 129.0 (C-5), 126.7 (C-6), 123.6 (C-2), 60.2 (C-11), 42.5 (C-8), 29.5 (C-10), 25.8 (C-9), 20.8 (C-7) ppm; MS: *m*/*z* = 266.2 (100%, [M + Na]^+^); anal. calcd. for C_11_H_17_NSO_3_ (243.32): C 54.30, H 7.04, N 5.76; found: C 54.00, H 7.31, N 5.52.

#### 4.2.44. 4-[(3-Methylphenyl)sulfonamido]butyl Sulfamate (**21b**)

Applying GPB: from **21a** (250 mg, 1.03 mmol): **21b** (317 mg, 96%); white solid; R_f_ = 0.55 (CHCl_3_/EtOAc, 2:3); m.p. = 62–64 °C; UV–Vis: 225 nm (3.84); IR: ν = 3351*w*, 3272*m*, 1474*w*, 1428*w*, 1375*s*, 1339*w*, 1315*m*, 1303*s*, 1198*w*, 1171*s*, 1153*vs*, 1097*m*, 1091*m*, 1065*m*, 1014*w*, 970*s*, 930*m*, 897*m*, 888*m*, 823*m*, 793*m*, 746*w*, 701*s*, 697*s*, 688*s*, 609*m*, 594*s*, 582*s*, 553*s*, 525*m*, 491*m*, 436*w* cm^−1^; ^1^H NMR:δ = 7.59–7.50 (*m*, 3H, 4-H, 5-H, NH), 7.49–7.39 (*m*, 2H, 2-H, 6-H), 7.36 (*s*, 2H, NH_2_), 3.94 (*t*, *J* = 6.3 Hz, 2H, 11-H), 2.72 (*q*, *J* = 6.7 Hz, 2H, 8-H), 2.36 (*s*, 3H, 7-H), 1.63–1.54 (*m*, 2H, 10-H), 1.47–1.38 (*m*, 2H, 9-H) ppm; ^13^C NMR:δ = 140.5 (C-1), 138.9 (C-3), 132.9 (C-4), 129.1 (C-5), 126.7 (C-6), 123.6 (C-2), 68.6 (C-11), 42.0 (C-8), 25.6 (C-10), 24.7 (C-9), 20.9 (C-7) ppm; MS: *m*/*z* = 345.6 (90%, [M + Na]^+^); anal. calcd. for C_11_H_18_N_2_S_2_O_5_ (322.39): C 40.98, H 5.63, N 8.69; found: C 40.76, H 8.92, N 8.38.

#### 4.2.45. N-(5-Hydroxypentyl)-3-methylbenzene Sulfonamide (**22a**) [1986639-01-3]

Applying GPA: from 3-methylbenzenesulfonyl chloride (500 mg, 2.62 mmol) and 5-amino-pentanol (406 mg, 3.93 mmol): **22a** (620 mg, 92%); oil; R_f_ = 0.13 (petrolether/EtOAc, 2:3); UV–Vis: 224 nm (3.89); IR: ν = 3454*w*, 3108*w*, 2954*w*, 2919*w*, 2880*w*, 2862*w*, 1597*vw*, 1474*w*, 1455*w*, 1437*w*, 1313*s*, 1305*m*, 1289*m*, 1245*w*, 1150*s*, 1122*m*, 1108*w*, 1091*s*, 1061*m*, 1041*w*, 1027*m*, 1019*w*, 940*m*, 849*w*, 817*m*, 801*w*, 755*m*, 721*m*, 709*m*, 662*s*, 636*w*, 577*s*, 551*vs*, 484*m*, 458*w* cm^−1^; ^1^H NMR:δ = 7.62–7.60 (*m*, 1H, 6-H), 7.60–7.56 (*m*, 1H, 2-H), 7.51–7.41 (*m*, 3H, 4-H, 5-H, NH), 4.31 (*t*, *J* = 5.1 Hz, 1H, OH), 3.36–3.30 (*m*, 2H, 12-H), 2.71 (*q*, *J* = 6.5 Hz, 2H, 8-H), 2.39 (*s*, 3H, 7-H), 1.41–1.29 (*m*, 4H, 9-H, 11-H), 1.28–1.18 (*m*, 2H, 10-H) ppm; ^13^C NMR:δ = 141.0 (C-1), 139.3 (C-3), 133.3 (C-4), 129.5 (C-5), 127.1 (C-6), 124.1 (C-2), 61.0 (C-12), 43.1 (C-8), 32.5 (C-11), 29.4 (C-9), 23.1 (C-10), 21.3 (C-7) ppm; MS: *m*/*z* = 280.3 (100%, [M + Na]^+^); anal. calcd. for C_12_H_19_NSO_3_ (257.35): C 56.01, H 7.44, N 5.44; found: C 55.76, H 7.69, N 5.20.

#### 4.2.46. 5-[(3-Methylphenyl)sulfonamido]pentyl Sulfamate (**22b**)

Applying GPB: from **22a** (300 mg, 1.17 mmol): **22b** (378 mg, 96%); white solid; R_f_ = 0.60 (CHCl_3_/EtOAc, 2:3); m.p. = 67–68 °C; UV–Vis: 224 nm (3.75); IR: ν = 3371*w*, 3265*m*, 2978*vw*, 2933*w*, 2867*vw*, 1536*vw*, 1475*w*, 1462*w*, 1439*w*, 1423*w*, 1404*w*, 1377*m*, 1358*s*, 1316*m*, 1302*s*, 1281*w*, 1227*w*, 1179*s*, 1150*vs*, 1138*m*, 1097*w*, 1085*m*, 1061*m*, 1049*m*, 1037*w*, 997*w*, 976*s*, 960*s*, 931*m*, 911*s*, 878*w*, 856*w*, 823*s*, 785*m*, 749*m*, 713*s*, 699*s*, 685*s*, 598*s*, 581*s*, 566*s*, 551*s*, 538*m*, 525*s*, 489*m*, 466*s*, 449*w*, 448*w*, 404*vw* cm^−1^; ^1^H NMR:δ = 7.61–7.59 (*m*, 1H, 6-H), 7.58–7.56 (*m*, 1H, 2-H), 7.54–7.42 (*m*, 3H, 4-H, 5-H, NH), 7.37 (*s*, 2H), 3.96 (*t*, *J* = 6.5 Hz, 2H, 12-H), 2.72 (*q*, *J* = 6.6 Hz, 2H, 8-H), 2.39 (*s*, 3H, 7-H), 1.56 (*p*, *J* = 6.7 Hz, 2H, 11-H), 1.44–1.35 (*m*, 2H, 9-H), 1.33–1.25 (*m*, 2H, 10-H) ppm; ^13^C NMR:δ = 140.5 (C-1), 138.8 (C-3), 132.9 (C-4), 129.0 (C-5), 126.6 (C-6), 123.6 (C-2), 68.8 (C-12), 42.3 (C-8), 28.5 (C-11), 27.8 (C-9), 22.2 (C-10), 20.8 (C-7) ppm; MS: *m*/*z* = 359.1 (100%, [M + Na]^+^); anal. calcd. for C_12_H_20_N_2_S_2_O_5_ (336.42): C 42.84, H 5.99, N 8.33; found: C 42.62, H 6.13, N 8.17.

#### 4.2.47. N-(6-Hydroxyhexyl)-3-methylbenzene Sulfonamide (**23a**) [1916290-41-9]

Applying GPA: from 3-methylbenzenesulfonyl chloride (500 mg, 2.62 mmol) and 6-amino-hexanol (461 mg, 3.93 mmol): **23a** (649 mg, 91%); oil; R_f_ = 0.15 (petrolether/EtOAc, 2:3); UV–Vis: 224 nm (3.90); IR: ν = 3503*w*, 3279*w*, 2933*m*, 2860*w*, 1477*w*, 1428*w*, 1321*m*, 1303*s*, 1221*w*, 1148*vs*, 1097*m*, 1086*m*, 1072*m*, 1054*m*, 882*w*, 787*m*, 688*s*, 591*s*, 581*vs*, 525*m*, 490*m*, 459*w*, 453*w*, 435*w* cm^−1^; ^1^H NMR:δ = 7.61–7.59 (*m*, 1H, 6-H), 7.59–7.56 (*m*, 1H, 2-H), 7.50–7.41 (*m*, 3H, 4-H, 5-H, NH), 4.30 (*t*, *J* = 5.2 Hz, 1H, OH), 3.38–3.31 (*m*, 2H, 13-H), 2.75–2.67 (*m*, 2H, 8-H), 2.39 (*s*, 3H, 7-H), 1.40–1.28 (*m*, 4H, 9-H, 12-H), 1.24–1.13 (*m*, 4H, 10-H, 11-H) ppm; ^13^C NMR:δ = 140.6 (C-1), 138.8 (C-3), 132.8 (C-4), 129.0 (C-5), 126.7 (C-6), 123.6 (C-2), 60.6 (C-13), 42.5 (C-8), 32.4 (C-12), 29.0 (C-9), 25.9 (C-10), 25.0 (C-11), 20.8 (C-7) ppm; MS: *m*/*z* = 294.4 (100%, [M + Na]^+^); anal. calcd. for C_13_H_21_NSO_3_ (271.38): C 57.54, H 7.80, N 5.16; found: C 57.26, H 8.01, N 4.97. 

#### 4.2.48. 6-[(3-Methylphenyl)sulfonamido]hexyl Sulfamate (**23b**)

Applying GPB: from **23a** (300 mg, 1.11 mmol): **23b** (364 mg, 94%); white solid; R_f_ = 0.64 (CHCl_3_/EtOAc, 2:3); m.p. = 74–75 °C; UV–Vis: 224 nm (3.84); IR: ν = 3371*w*, 3261*m*, 2975*vw*, 2958*vw*, 2943*w*, 2910*vw*, 2897*vw*, 2852*w*, 1556*w*, 1479*w*, 1472*w*, 1455*w*, 1431*w*, 1398*w*, 1369*s*, 1341*w*, 1323*s*, 1308*m*, 1282*w*, 1230*vw*, 1221*vw*, 1161*vs*, 1112*vw*, 1099*w*, 1086*w*, 1062*w*, 1053*w*, 1042*vw*, 1007*m*, 988*vw*, 963*s*, 940*m*, 912*m*, 881*w*, 861*vw*, 818*s*, 801*w*, 791*w*, 720*m*, 685*s*, 596*vs*, 580*s*, 550*m*, 529*m*, 505*w*, 494*m*, 457*vw*, 436*w*, 404*vw* cm^−1^; ^1^H NMR:δ = 7.62–7.55 (*m*, 2H, 2-H, 6-H), 7.52–7.42 (*m*, 3H, 4-H, 5-H), 7.37 (*s*, 2H, NH_2_), 3.97 (*t*, *J* = 6.5 Hz, 2H, 13-H), 2.72 (*q*, *J* = 6.6 Hz, 2H, 8-H), 2.39 (*s*, 3H, 7-H), 1.56 (*p*, *J* = 6.7 Hz, 2H, 12-H), 1.36 (*p*, *J* = 6.9 Hz, 2H, 9-H), 1.28–1.18 (*m*, 4H, 10-H, 11-H) ppm; ^13^C NMR:δ = 140.5 (C-1), 138.8 (C-3), 132.8 (C-4), 129.0 (C-5), 126.6 (C-6), 123.6 (C-2), 68.9 (C-13), 42.4 (C-8), 28.8 (C-12), 28.2 (C-9), 25.5 (C-10), 24.6 (C-11), 20.8 (C-7) ppm; MS: *m*/*z* = 373.3 (100%, [M + Na]^+^); anal. calcd. for C_13_H_22_N_2_S_2_O_5_ (350.45): C 44.56, H 6.33, N 7.99; found: C 44.26, H 6.58, N 8.19.

#### 4.2.49. N-(7-Hydroxyheptyl)-3-methylbenzene Sulfonamide (**24a**)

Applying GPA: from 3-methylbenzenesulfonyl chloride (500 mg, 2.62 mmol) and 7-amino-heptanol (516 mg, 3.93 mmol): **24a** (648 mg, 87%); oil; R_f_ = 0.19 (petrolether/EtOAc, 2:3); UV–Vis: 224 nm (3.78); IR: ν = 3474*m*, 3128*m*, 2930*m*, 2889*w*, 2850*m*, 1483*w*, 1465*m*, 1447*m*, 1402*w*, 1372*w*, 1317*m*, 1309*m*, 1298*m*, 1289*m*, 1222*m*, 1143*vs*, 1099*s*, 1083*s*, 1072*s*, 1020*m*, 1001*m*, 957*m*, 906*m*, 870*w*, 862*w*, 835*w*, 790*s*, 755*w*, 709*s*, 689*s*, 584*vs*, 555*m*, 545*m*, 524*m*, 483*s*, 472*m*, 439*m*, 408*w* cm^−1^; ^1^H NMR:δ = 7.61–7.56 (*m*, 2H, 2-H, 6-H), 7.50–7.41 (*m*, 3H, 4-H, 5-H, NH), 4.30 (*td*, *J* = 5.2, 1.1 Hz, 1H, OH), 3.38–3.33 (*m*, 2H, 14-H), 2.71 (*td*, *J* = 7.0, 5.8 Hz, 2H, 8-H), 2.39 (*s*, 3H, 7-H), 1.40–1.28 (*m*, 4H, 9-H, 13-H), 1.26–1.11 (*m*, 6H, 10-H, 11-H, 12-H) ppm; ^13^C NMR (126 MHz, DMSO-*d*_6_): δ = 140.6 (C-1), 138.8 (C-3), 132.8 (C-4), 129.0 (C-5), 126.6 (C-6), 123.6 (C-2), 60.7 (C-14), 42.5 (C-8), 32.4 (C-13), 28.9 (C-9), 28.4 (C-11), 26.0 (C-12), 25.3 (C-10), 20.8 (C-7) ppm; MS: *m*/*z* = 308.1 (100%, [M + Na]^+^); anal. calcd. for C_14_H_23_NSO_3_ (285.40): C 58.92, H 8.12, N 4.91; found: C 58.78, H 8.34, N 4.65. 

#### 4.2.50. 7-[(3-Methylphenyl)sulfonamido]heptyl Sulfamate (**24b**)

Applying GPB: from **24a** (300 mg, 1.05 mmol): **24b** (279 mg, 73%) a white waxy solid; R_f_ = 0.77 (CHCl_3_/EtOAc, 2:3); m.p. = 55–57 °C; UV–Vis: 224 nm (3.80); IR: ν = 3361*w*, 3265*m*, 2961*w*, 2937*w*, 2906*w*, 2895*w*, 2856*w*, 1566*w*, 1477*w*, 1429*m*, 1396*w*, 1370*s*, 1317*s*, 1301*m*, 1226*w*, 1183*s*, 1150*vs*, 1114*w*, 1096*w*, 1085*m*, 1061*m*, 1049*w*, 1000*m*, 972*s*, 927*m*, 922*m*, 904*m*, 878*w*, 825*s*, 783*m*, 741*w*, 723*w*, 702*s*, 685*s*, 665*m*, 597*s*, 578*s*, 556*vs*, 528*m*, 521*m*, 495*s*, 478*w*, 450*w*, 429*w*, 505*w*, 494*m*, 457*vw*, 436*w*, 404*vw* cm^−1^; ^1^H NMR:δ = 7.62–7.56 (*m*, 2H, 2-H, 6-H), 7.51–7.42 (*m*, 3H, 4-H, 5-H, NH), 7.37 (*s*, 2H, NH_2_), 3.98 (*t*, *J* = 6.5 Hz, 2H, 14-H), 2.72 (*td*, *J* = 7.0, 5.8 Hz, 2H, 8-H), 2.39 (*s*, 3H, 7-H), 1.63–1.54 (*m*, 2H, 13-H), 1.39–1.15 (*m*, 8H, 9-H, 10-H, 11-H, 12-H) ppm; ^13^C NMR (126 MHz, DMSO-*d*_6_): δ = 140.6 (C-1), 138.8 (C-3), 132.8 (C-4), 129.0 (C-5), 126.6 (C-6), 123.6 (C-2), 68.9 (C-14), 42.5 (C-8), 28.9 (C-9), 28.2 (C-13), 28.0 (C-11), 25.9 (C-10), 24.9 (C-12), 20.8 (C-7) ppm; MS: *m*/*z* = 387.2 (100%, [M + Na]^+^); anal. calcd. for C_14_H_24_N_2_S_2_O_5_ (364.48): C 46.14, H 6.64, N 7.69; found: C 45.97, H 6.90, N 7.47. 

#### 4.2.51. N-(8-Hydroxyoctyl)-3-methylbenzene Sulfonamide (**25a**)

Applying GPA: from 3-methylbenzenesulfonyl chloride (500 mg, 2.62 mmol) and 8-amino-octanol (571 mg, 3.93 mmol): **25a** (702 mg, 89%); oil; R_f_ = 0.25 (petrolether/EtOAc, 2:3); UV–Vis: 224 nm (3.90); IR: ν = 3508*w*, 3282*w*, 2928*m*, 2856*m*, 1477*w*, 1458*w*, 1429*w*, 1322*s*, 1303*s*, 1221*w*, 1149*vs*, 1097*m*, 1086*m*, 999*w*, 883*w*, 786*m*, 689*s*, 592*s*, 581*vs*, 525*m*, 492*m*, 436*w* cm^−1^; ^1^H NMR:δ = 7.61–7.59 (*m*, 1H, 6-H), 7.59–7.56 (*m*, 1H, 2-H), 7.50–7.41 (*m*, 3H, 4-H, 5-H, NH), 4.30 (*t*, *J* = 5.1 Hz, 1H, OH), 3.39–3.32 (*m*, 2H, 15-H), 2.71 (*td*, *J* = 7.0, 5.8 Hz, 2H, 8-H), 2.38 (*s*, 3H, 7-H), 1.42–1.28 (*m*, 4H, 9-H, 14-H), 1.26–1.11 (*m*, 8H, 10-H, 11-H, 12-H, 13-H) ppm; ^13^C NMR:δ = 140.6 (C-1), 138.8 (C-3), 132.8 (C-4), 129.0 (C-5), 126.7 (C-6), 123.6 (C-2), 60.7 (C-15), 42.5 (C-8), 32.5 (C-14), 28.9 (C-9), 28.8 (C-11), 28.6 (C-12), 26.0 (C-10), 25.4 (C-13), 20.8 (C-7) ppm; MS: *m*/*z* = 322.1 (100%, [M + Na]^+^); anal. calcd. for C_15_H_25_NSO_3_ (299.43): C 60.17, H 8.42, N 4.68; found: C 59.87, H 8.70, N 4.45.

#### 4.2.52. 8-[(3-Methylphenyl)sulfonamido]octyl Sulfamate (**25b**)

Applying GPB: from **25a** (300 mg, 1.00 mmol): **25b** (284 mg, 75%); white solid; R_f_ = 0.83 (CHCl_3_/EtOAc, 2:3); m.p. = 54–56 °C; UV–Vis: 224 nm (3.84); IR: ν = 3369*w*, 3254*m*, 2970*w*, 2938*m*, 2920*m*, 2859*m*, 2853*w*, 1562*w*, 1473*m*, 1458*w*, 1440*m*, 1431*w*, 1397*w*, 1324*s*, 1303*s*, 1217*w*, 1164*s*, 1150*vs*, 1116*w*, 1099*m*, 1087*m*, 1057*m*, 1053*m*, 1039*w*, 994*m*, 960*vs*, 920*m*, 896*s*, 864*w*, 849*vw*, 832*m*, 800*w*, 787*m*, 731*w*, 701*s*, 688*vs*, 602*m*, 578*s*, 569*s*, 524*m*, 517*m*, 493*s*, 478*m*, 446*w*, 431*w*, 415*w*, 457*vw*, 436*w*, 404*vw* cm^−1^; ^1^H NMR:δ = 7.61–7.58 (*m*, 2H, 6-H), 7.58–7.56 (*m*, 1H, 2-H), 7.50–7.42 (*m*, 3H, 4-H, 5-H, NH), 7.37 (*s*, 2H, NH_2_), 3.99 (*t*, *J* = 6.5 Hz, 2H, 15-H), 2.71 (*td*, *J* = 7.0, 5.8 Hz, 2H, 8-H), 2.39 (*s*, 3H, 7-H), 1.65–1.55 (*m*, 2H, 14-H), 1.40–1.24 (*m*, 2H, 9-H, 13-H), 1.23–1.14 (*m*, 6H, 10-H, 11-H, 12-H) ppm; ^13^C NMR (126 MHz, DMSO-*d*_6_): δ = 140.6 (C-1), 138.8 (C-3), 132.8 (C-4), 129.0 (C-5), 126.6 (C-6), 123.6 (C-2), 69.0 (C-15), 42.5 (C-8), 28.9 (C-14), 28.4 (C-9), 28.3 (C-11), 28.3 (C-12), 25.9 (C-10), 24.9 (C-13), 20.8 (C-7) ppm; MS (ESI, MeOH) *m*/*z* = 401.3 (100%, [M + Na]^+^); anal. calcd. for C_15_H_26_N_2_S_2_O_5_ (378.50): C 47.60, H 6.92, N 7.40; found: C 47.36, H 7.17, N 7.15.

#### 4.2.53. N-(2-Hydroxyethyl)-2-methylbenzene Sulfonamide (**26a**) [19829-14-2]

Applying GPA: from 2-methylbenzenesulfonyl chloride (500 mg, 2.62 mmol) and 2-amino-ethanol (3.93 mg, 3.93 mmol): **26a** (420 mg, 74%) [115,116,117]; white solid; R_f_ = 0.11 (petrolether/EtOAc, 2:3); m.p. = 73–74 °C (lit.: [76,116] 73–75 °C ^(4)^); UV–Vis: 222 nm (3.87); IR: ν = 3452*m*, 3189*m*, 3067*w*, 2959*w*, 2939*w*, 2867*w*, 2687*vw*, 1592*vw*, 1459*m*, 1421*m*, 1399*w*, 1381*w*, 1349*w*, 1301*s*, 1285*m*, 1261*m*, 1207*w*, 1154*vs*, 1133*s*, 1094*s*, 1070*m*, 1059*s*, 1037*m*, 995*w*, 963*s*, 899*w*, 880*vw*, 839*m*, 803*w*, 760*s*, 710*m*, 687*s*, 590*s*, 580*vs*, 542*s*, 511*m*, 491*m*, 468*s*, 443*w*, 415*m* cm^−1^; ^1^H NMR (500 MHz, DMSO-*d*_6_): δ = 7.81 (*dd*, *J* = 7.9, 1.4 Hz, 1H, 3-H), 7.63–7.58 (*m*, 1H, 6-H), 7.51 (*td*, *J* = 7.5, 1.4 Hz, 1H, 4-H), 7.41–7.35 (*m*, 2H, 5-H, NH), 4.65 (*t*, *J* = 5.5 Hz, 1H, OH), 3.36–3.32 (*m*, 2H, 9-H), 2.81 (*t*, *J* = 6.5 Hz, 2H, 8-H), 2.57 (*s*, 3H, 7-H) ppm; ^13^C NMR (126 MHz, DMSO-*d*_6_): δ = 138.8 (C-1), 136.5 (C-2), 132.4 (C-4), 132.3 (C-3), 128.3 (C-6), 126.1 (C-5), 59.9 (C-9), 44.7 (C-8), 19.7 (C-7) ppm; MS: *m*/*z* = 238.2 (100%, [M + Na]^+^); anal. calcd. for C_9_H_13_NSO_3_ (215.27): C 50.22, H 6.09, N 6.51; found: C 49.98, H 6.37, N 6.39.

#### 4.2.54. 2-[(2-Methylphenyl)sulfonamido]ethyl Sulfamate (**26b**)

Applying GPB: from **26a** (150 mg, 0.70 mmol): **26b** (185 mg, 90%); white solid; R_f_ = 0.52 (CHCl_3_/EtOAc, 2:3); m.p. = 70–72 °C; UV–Vis: 270 nm (3.08); IR: ν = 3394*w*, 3359*w*, 3311*m*, 3288*m*, 3256*m*, 1566*vw*, 1539*w*, 1479*vw*, 1455*w*, 1434*w*, 1417*w*, 1398*w*, 1370*s*, 1358*vs*, 1320*m*, 1309*vs*, 1290*m*, 1237*w*, 1190*m*, 1174*s*, 1155*vs*, 1127*m*, 1112*m*, 1091*w*, 1076*m*, 1026*m*, 962*s*, 933*s*, 909*s*, 872*w*, 845*w*, 803*m*, 759*vs*, 753*vs*, 708*m*, 691*m*, 590*s*, 567*m*, 548*vs*, 538*s*, 526*s*, 514*m*, 481*m*, 467*m*, 454*m*, 424*m*, 410*w* cm^−1^; ^1^H NMR:δ = 7.97 (*t*, *J* = 6.0 Hz, 1H, NH), 7.82 (*dd*, *J* = 7.8, 1.4 Hz, 1H, 3-H), 7.56–7.46 (*m*, 3H, 4-H, NH_2_), 7.44–7.35 (*m*, 2H, 5-H, 6-H), 3.97 (*t*, *J* = 5.8 Hz, 2H, 9-H), 3.08 (*q*, *J* = 5.7 Hz, 2H, 8-H), 2.58 (*s*, 3H, 7-H) ppm; ^13^C NMR (126 MHz, DMSO-*d*_6_): δ = 138.4 (C-1), 136.6 (C-2), 132.6 (C-4), 132.5 (C-3), 128.3 (C-6), 126.2 (C-5), 67.5 (C-9), 41.2 (C-8), 19.8 (C-7) ppm; MS: *m*/*z* = 317.2 (100%, [M + Na]^+^); anal. calcd. for C_9_H_14_N_2_S_2_O_5_ (294.34): C 36.73, H 4.79, N 9.52; found: C 36.54, H 4.44, N 9.38. 

#### 4.2.55. N-(3-Hydroxypropyl)-2-methylbenzene Sulfonamide (**27a**) [1082811-80-0]

Applying GPA: from 2-methylbenzenesulfonyl chloride (500 mg, 2.62 mmol) and 3-amino-propanol (240 mg, 3.93 mmol): **27a** (420 mg, 74%); oil; R_f_ = 0.15 (petrolether/EtOAc, 2:3); UV–Vis: 222 nm (3.84); IR: ν = 3498*w*, 3294*w*, 2939*w*, 2882*w*, 1472*w*, 1457*w*, 1311*s*, 1290*m*, 1153*vs*, 1131*m*, 1065*s*, 1006*w*, 957*w*, 872*w*, 806*w*, 759*m*, 710*m*, 689*m*, 591*vs*, 575*s*, 541*m*, 481*m*, 444*w*, 425*w*, 420*w* cm^−1^; ^1^H NMR:δ = 7.79 (*dd*, *J* = 7.8, 1.4 Hz, 1H, 3-H), 7.61–7.51 (*m*, 1H, 6-H), 7.48 (*td*, *J* = 7.5, 1.4 Hz, 1H, 4-H), 7.40–7.32 (*m*, 2H, 5-H, NH), 4.38 (*s*, 1H, OH), 3.36–3.30 (*m*, 2H, 10-H), 2.80 (*t*, *J* = 7.2 Hz, 2H, 8-H), 2.56 (*s*, 3H, 7-H), 1.55–1.44 (*m*, 2H, 9-H) ppm; ^13^C NMR:δ = 138.7 (C-1), 136.5 (C-2), 132.5 (C-4), 132.3 (C-3), 128.4 (C-6), 126.2 (C-5), 58.1 (C-10), 39.8 (C-8), 32.4 (C-9), 19.8 (C-7) ppm; MS: *m*/*z* = 252.2 (100%, [M + Na]^+^); anal. calcd. for C_10_H_15_NSO_3_ (229.29): C 52.38, H 6.59, N 6.11; found: C 52.04, H 6.80, N 5.96.

#### 4.2.56. 3-[(2-Methylphenyl)sulfonamido]propyl Sulfamate (**27b**)

Applying GPB: from **27a** (300 mg, 1.31 mmol): **27b** (328 mg, 81%); white solid; R_f_ = 0.54 (CHCl_3_/EtOAc, 2:3); m.p. = 68–70 °C; UV–Vis: 224 nm (3.90); IR: ν = 3314*m*, 3238*m*, 3115*w*, 2942*vw*, 1570*w*, 1470*w*, 1439*w*, 1418*w*, 1396*w*, 1361*s*, 1315*s*, 1281*w*, 1216*vw*, 1199*vw*, 1171*m*, 1155*vs*, 1134*m*, 1111*m*, 1095*m*, 1066*m*, 1056*w*, 1005*w*, 940*s*, 886*m*, 843*s*, 805*w*, 763*s*, 711*m*, 691*s*, 643*w*, 589*vs*, 574*s*, 549*vs*, 543*vs*, 508*m*, 486*m*, 457*m*, 436*w*, 415*w* cm^−1^; ^1^H NMR:δ = 7.85–7.77 (*m*, 1H, 3-H), 7.71 (*t*, *J* = 5.8 Hz, 1H, 6-H), 7.56–7.47 (*m*, 1H, 4-H), 7.44–7.34 (*m*, 4H, 5-H, NH, NH_2_), 4.01 (*t*, *J* = 6.3 Hz, 2H, 10-H), 2.84 (*td*, *J* = 7.2, 5.8 Hz, 2H, 8-H), 2.57 (*s*, 3H, 7-H), 1.76 (*p*, *J* = 6.6 Hz, 2H, 9-H) ppm; ^13^C NMR (126 MHz, DMSO-*d*_6_): δ = 138.5 (C-1), 136.5 (C-2), 132.5 (C-4), 132.4 (C-3), 128.3 (C-6), 126.2 (C-5), 66.5 (C-10), 39.0 (C-8), 28.9 (C-9), 19.8 (C-7) ppm; MS: *m*/*z* = 331.3 (100%, [M + Na]^+^); anal. calcd. for C_10_H_16_N_2_S_2_O_3_ (308.37): C 38.95, H 5.23, N 9.08; found: C 38.78, H 5.51, N 8.76.

#### 4.2.57. N-(4-Hydroxybutyl)-2-methylbenzene Sulfonamide (**28a**) [1082772-69-7]

Applying GPA: from 2-methylbenzenesulfonyl chloride (500 mg, 2.62 mmol) and 4-amino-butanol (351 mg, 3.93 mmol): **28a** (546 mg, 86%); oil; R_f_ = 0.15 (petrolether/EtOAc, 2:3); UV–Vis: 224 nm (3.85); IR: ν = 3502*w*, 3296*w*, 2939*w*, 2872*w*, 1472*w*, 1456*w*, 1311*s*, 1153*vs*, 1131*m*, 1065*s*, 1034*m*, 952*vw*, 867*w*, 808*w*, 760*m*, 711*m*, 688*m*, 591*vs*, 541*m*, 488*m*, 451*w*, 444*w* cm^−1^; ^1^H NMR:δ = 7.80 (*dd*, *J* = 7.8, 1.4 Hz, 1H, 3-H), 7.65–7.55 (*m*, 1H, 6-H), 7.50 (*td*, *J* = 7.5, 1.4 Hz, 1H, 4-H), 7.41–7.33 (*m*, 2H, 5-H, NH), 4.35 (*s*, 1H, OH), 3.30 (*t*, *J* = 6.0 Hz, 2H, 8-H), 2.76 (*t*, *J* = 6.7 Hz, 2H, 11-H), 2.57 (*s*, 3H, 7-H), 1.43–1.29 (*m*, 4H, 9-H, 10-H) ppm; ^13^C NMR:δ = 138.8 (C-1), 136.4 (C-2), 132.4 (C-4), 132.3 (C-3), 128.3 (C-6), 126.1 (C-5), 60.2 (C-11), 42.3 (C-8), 29.5 (C-10), 25.9 (C-9), 19.8 (C-7) ppm; MS: *m*/*z* = 266.3 (100%, [M + Na]^+^); anal. calcd. for C_11_H_17_NSO_3_ (243.32): C 54.30, H 7.04, N 5.76; found: C 53.99, H 7.34, N 5.31.

#### 4.2.58. 4-[(2-Methylphenyl)sulfonamido]butyl Sulfamate (**28b**)

Applying GPB: from **28a** (300 mg, 1.23 mmol): **28b** (312 mg, 79%); oil; R_f_ = 0.55 (CHCl_3_/EtOAc, 2:3); UV–Vis: 224 nm (3.72); IR: ν = 3317*m*, 3240*m*, 3112*w*, 2940*vw*, 1574*w*, 1471*w*, 1438*w*, 1417*w*, 1393*w*, 1360*s*, 1317*s*, 1280*w*, 1214*vw*, 1198*vw*, 1170*m*, 1154*vs*, 1130*m*, 1110*m*, 1096*m*, 1068*m*, 1050*w*, 1001*w*, 945*s*, 884*m*, 845*s*, 801*w*, 760*s*, 711*m*, 690*s*, 646*w*, 590*vs*, 572*s*, 548*vs*, 543*vs*, 508*m*, 484*m*, 456*m*, 433*w*, 415*w* cm^−1^; ^1^H NMR:δ = 7.82–7.78 (*m*, 1H, 3-H), 7.66 (*t*, *J* = 5.9 Hz, 1H, 6-H), 7.51 (*td*, *J* = 7.5, 1.4 Hz, 1H, 4-H), 7.42–7.35 (*m*, 4H, 5-H, NH, NH_2_), 3.95 (*t*, *J* = 6.4 Hz, 2H, 11-H), 2.79 (*q*, *J* = 6.7 Hz, 2H, 8-H), 2.57 (*s*, 3H, 7-H), 1.64–1.55 (*m*, 2H, 10-H), 1.50–1.39 (*m*, 2H, 9-H) ppm; ^13^C NMR:δ = 139.2 (C-1), 136.9 (C-2), 132.9 (C-4), 132.8 (C-3), 128.7 (C-6), 126.6 (C-5), 69.0 (C-11), 42.2 (C-8), 26.0 (C-10), 26.0 (C-9), 20.2 (C-7) ppm; MS: *m*/*z* = 345.3 (100%, [M + Na]^+^); anal. calcd. for C_11_H_18_N_2_S_2_O_5_ (322.39): C 40.98, H 5.63, N 8.69; found: C 40.77, H 5.90, N 8.41.

#### 4.2.59. N-(5-Hydroxypentyl)-2-methylbenzene Sulfonamide (**29a**) [1965509-68-5]

Applying GPA: from 2-methylbenzenesulfonyl chloride (500 mg, 2.62 mmol) and 5-amino-pentanol (406 mg, 3.93 mmol): **29a** (396 mg, 59%); oil; R_f_ = 0.17 (petrolether/EtOAc, 2:3); UV–Vis: 222 nm (3.87); IR: ν = 3501*w*, 3295*w*, 2937*w*, 2864*w*, 1472*w*, 1457*w*, 1313*s*, 1154*vs*, 1131*m*, 1066*m*, 1046*m*, 1039*m*, 877*w*, 806*w*, 760*m*, 733*w*, 711*m*, 688*m*, 592*vs*, 541*m*, 488*m*, 423*w*, 418*w* cm^−1^; ^1^H NMR:δ = 7.80 (*dd*, *J* = 7.8, 1.4 Hz, 1H, 3-H), 7.62–7.56 (*m*, 1H, 6-H), 7.50 (*td*, *J* = 7.5, 1.4 Hz, 1H, 4-H), 7.41–7.33 (*m*, 2H, 5-H, NH), 4.30 (*t*, *J* = 5.1 Hz, 1H, OH), 3.30 (*td*, *J* = 6.4, 5.1 Hz, 2H, 12-H), 2.74 (*td*, *J* = 7.0, 5.5 Hz, 2H, 8-H), 2.57 (*s*, 3H, 7-H), 1.39–1.25 (*m*, 4H, 9-H, 11-H), 1.25–1.15 (*m*, 2H, 10-H) ppm; ^13^C NMR (126 MHz, DMSO-*d*_6_): δ = 138.9 (C-1), 136.4 (C-2), 132.4 (C-4), 132.2 (C-3), 128.3 (C-6), 126.1 (C-5), 60.5 (C-12), 42.3 (C-8), 31.9 (C-11), 29.0 (C-9), 22.5 (C-10), 19.8 (C-7) ppm; MS: *m*/*z* = 280.2 (100%, [M + Na]^+^); anal. calcd. for C_12_H_19_NSO_3_ (257.35): C 56.01, H 7.44, N 5.44; found: C 55.76, H 7.69, N 5.16. 

#### 4.2.60. 5-[(2-Methylphenyl)sulfonamido]pentyl Sulfamate (**29b**)

Applying GPB: from **29a** (300 mg, 1.17 mmol): **29b** (282 mg, 72%); oil; R_f_ = 0.64 (CHCl_3_/EtOAc, 2:3); UV–Vis: 224 nm (4.02); IR: ν = 3277*w*, 2941*w*, 2868*vw*, 1567*w*, 1472*w*, 1458*w*, 1360*m*, 1312*s*, 1177*s*, 1154*vs*, 1130*m*, 1082*m*, 1066*m*, 1048*w*, 1034*w*, 919*s*, 813*m*, 761*m*, 729*w*, 710*m*, 689*m*, 592*s*, 551*s*, 542*s*, 494*m*, 480*m* cm^−1^; ^1^H NMR:δ = 7.83–7.78 (*m*, 1H, 3-H), 7.62 (*t*, *J* = 5.9 Hz, 1H, 6-H), 7.51 (*td*, *J* = 7.5, 1.4 Hz, 1H, 4-H), 7.42–7.34 (*m*, 4H, NH, 5-H, NH_2_), 3.94 (*t*, *J* = 6.5 Hz, 2H, 12-H), 2.76 (*q*, *J* = 6.8 Hz, 2H, 8-H), 2.57 (*s*, 3H, 7-H), 1.57–1.48 (*m*, 2H, 11-H), 1.42–1.33 (*m*, 2H, 9-H), 1.33–1.22 (*m*, 2H, 10-H) ppm; ^13^C NMR:δ = 138.8 (C-1), 136.4 (C-2), 132.5 (C-4), 132.3 (C-3), 128.3 (C-6), 126.2 (C-5), 68.8 (C-12), 42.0 (C-8), 28.6 (C-11), 27.8 (C-9), 22.2 (C-10), 19.8 (C-7) ppm; MS: *m*/*z* = 359.4 (100%, [M + Na]^+^); anal. calcd. for C_12_H_20_N_2_S_2_O_5_ (336.42): C 42.84, H 5.99, N 8.33; found: C 42.56, H 6.23, N 8.04.

#### 4.2.61. N-(6-Hydroxyhexyl)-2-methylbenzene Sulfonamide (**30a**) [1914655-85-8]

Applying GPA: from 2-methylbenzenesulfonyl chloride (500 mg, 2.62 mmol) and 6-amino-hexanol (461 mg, 3.93 mmol): **30a** (666 mg, 94%); oil; R_f_ = 0.19 (petrolether/EtOAc, 2:3); UV–Vis: 223 nm (3.97); IR: ν = 3503*w*, 3296*w*, 2934*m*, 2860*w*, 1471*w*, 1458*w*, 1314*s*, 1154*vs*, 1131*m*, 1066*m*, 807*w*, 759*m*, 728*w*, 711*m*, 688*m*, 592*vs*, 541*m*, 490*m* cm^−1^; ^1^H NMR:δ = 7.80 (*dd*, *J* = 7.8, 1.4 Hz, 1H, 3-H), 7.59 (*t*, *J* = 5.7 Hz, 1H, 6-H), 7.50 (*td*, *J* = 7.5, 1.4 Hz, 1H, 4-H), 7.41–7.33 (*m*, 2H, 5-H, NH), 4.29 (*t*, *J* = 5.1 Hz, 1H, OH), 3.36–3.29 (*m*, 2H, 13-H), 2.75 (*td*, *J* = 7.1, 5.8 Hz, 2H, 8-H), 2.57 (*s*, 3H, 7-H), 1.38–1.26 (*m*, 4H, 9-H, 12-H), 1.21–1.10 (*m*, 4H, 10-H, 11-H) ppm; ^13^C NMR:δ = 138.9 (C-1), 136.4 (C-2), 132.4 (C-4), 132.2 (C-3), 128.3 (C-6), 126.1 (C-5), 60.6 (C-13), 42.2 (C-8), 32.3 (C-12), 29.1 (C-9), 25.9 (C-10), 25.0 (C-11), 19.8 (C-7) ppm; MS: *m*/*z* = 294.2 (100%, [M + Na]^+^); anal. calcd. for C_13_H_21_NSO_3_ (271.38): C 57.54, H 7.80, N 5.16; found: C 76.26, H 8.03, N 4.76.

#### 4.2.62. 6-[(2-Methylphenyl)sulfonamido]hexyl Sulfamate (**30b**)

Applying GPB: from **30a** (300 mg, 1.11 mmol): **30b** (333 mg, 86%); oil; R_f_ = 0.71 (CHCl_3_/EtOAc, 2:3); UV–Vis: 223 nm (3.05); IR: ν = 3277*w*, 3113*vw*, 2938*w*, 2863*w*, 1564*w*, 1471*w*, 1459*w*, 1360*s*, 1312*s*, 1177*s*, 1154*vs*, 1131*m*, 1082*m*, 1066*m*, 1048*w*, 922*s*, 807*m*, 761*m*, 726*w*, 710*m*, 689*m*, 592*s*, 551*s*, 543*s*, 492*m* cm^−1^; ^1^H NMR:δ = 7.80 (*dd*, *J* = 7.8, 1.4 Hz, 1H, 3-H), 7.60 (*t*, *J* = 5.8 Hz, 1H, 6-H), 7.50 (*td*, *J* = 7.4, 1.4 Hz, 1H, 4-H), 7.41–7.34 (*m*, 4H, 5-H, NH, NH_2_), 3.96 (*t*, *J* = 6.5 Hz, 2H, 13-H), 2.76 (*q*, *J* = 6.6 Hz, 2H, 8-H), 2.57 (*s*, 3H, 7-H), 1.59–1.47 (*m*, 2H, 12-H), 1.40–1.28 (*m*, 2H, 9-H), 1.26–1.15 (*m*, 4H, 10-H, 11-H) ppm; ^13^C NMR:δ = 139.3 (C-1), 136.9 (C-2), 132.9 (C-4), 132.7 (C-3), 128.8 (C-6), 126.6 (C-5), 69.3 (C-13), 42.6 (C-8), 29.4 (C-12), 28.6 (C-9), 25.9 (C-10), 25.0 (C-11), 20.2 (C-7) ppm; MS: *m*/*z* = 373.4 (100%, [M + Na]^+^); anal. calcd. for C_13_H_22_N_2_S_2_O_5_ (350.45): C 44.56, H 6.33, N 7.99; found: C 44.31, H 6.68, N 7.69.

#### 4.2.63. N-(7-Hydroxyheptyl)-2-methylbenzene Sulfonamide (**31a**)

Applying GPA: from 2-methylbenzenesulfonyl chloride (500 mg, 2.62 mmol) and 7-amino-heptanol (516 mg, 3.93 mmol): **31a** (394 mg, 53%); oil; R_f_ = 0.20 (petrolether/EtOAc, 2:3); UV–Vis: 223 nm (3.90); IR: ν = 3504*w*, 3295*w*, 3064*vw*, 2931*m*, 2858*w*, 1458*w*, 1314*s*, 1154*vs*, 1131*m*, 1066*m*, 871*w*, 806*w*, 760*m*, 725*w*, 711*m*, 688*m*, 592*vs*, 541*m*, 491*m*, 419*vw* cm^−1^; ^1^H NMR:δ = 7.80 (*dd*, *J* = 7.8, 1.4 Hz, 1H, 3-H), 7.62–7.56 (*m*, 1H, 6-H), 7.50 (*td*, *J* = 7.5, 1.4 Hz, 1H, 4-H), 7.40–7.33 (*m*, 2H, 5, NH), 4.30 (*t*, *J* = 5.1 Hz, 1H, OH), 3.37–3.31 (*m*, 2H, 14-H), 2.78–2.70 (*m*, 2H, 8-H), 2.57 (*s*, 3H, 7-H), 1.40–1.26 (*m*, 4H, 9-H, 13-H), 1.23–1.07 (*m*, 6H, 10-H, 11-H, 12-H) ppm; ^13^C NMR (126 MHz, DMSO-*d*_6_): δ = 138.9 (C-1), 136.4 (C-2), 132.4 (C-4), 132.2 (C-3), 128.3 (C-6), 126.1 (C-5), 60.7 (C-14), 42.2 (C-8), 32.4 (C-13), 29.0 (C-9), 28.4 (C-11), 26.0 (C-10), 25.3 (C-12), 19.8 (C-7) ppm; MS: *m*/*z* = 308.3 (100%, [M + Na]^+^); anal. calcd. for C_14_H_23_NSO_3_ (285.40): C 58.92, H 8.12, N 4.91; found: C 58.76, H 8.34, N 4.78.

#### 4.2.64. 7-[(2-Methylphenyl)sulfonamido]heptyl Sulfamate (**31b**)

Applying GPB: from **31a** (300 mg, 1.05 mmol): **31b** (294 mg, 77%); oil; R_f_ = 0.74 (SiO_2_ CHCl_3_/EtOAc, 2:3); UV–Vis: 269 nm (3.16); IR: ν = 3504*w*, 3295*w*, 3064*vw*, 2931*m*, 2858*w*, 1458*w*, 1314*s*, 1154*vs*, 1131*m*, 1066*m*, 871*w*, 806*w*, 760*m*, 725*w*, 711*m*, 688*m*, 592*vs*, 541*m*, 491*m*, 419*vw* cm^−1^; ^1^H NMR (500 MHz, DMSO-*d*_6_): δ = 7.80 (*dd*, *J* = 7.8, 1.4 Hz, 1H, 3-H), 7.60 (*t*, *J* = 5.8 Hz, 1H, 6-H), 7.50 (*td*, *J* = 7.5, 1.4 Hz, 1H, 4-H), 7.41–7.34 (*m*, 4H, 5-H, NH, NH_2_), 3.98 (*t*, *J* = 6.5 Hz, 2H, 14-H), 2.75 (*td*, *J* = 7.0, 5.8 Hz, 2H, 8-H), 2.57 (*s*, 3H, 7-H), 1.60–1.53 (*m*, 2H, 13-H), 1.37–1.28 (*m*, 2H, 9-H), 1.27–1.20 (*m*, 2H, 12-H), 1.19–1.13 (*m*, 4H, 10-H, 11-H) ppm; ^13^C NMR:δ = 138.9 (C-1), 136.4 (C-2), 132.4 (C-4), 132.3 (C-3), 128.3 (C-6), 126.1 (C-5), 68.9 (C-14), 42.2 (C-8), 28.9 (C-13), 28.2 (C-9), 27.9 (C-11), 25.8 (C-12), 24.9 (C-10), 19.8 (C-7) ppm; MS: *m*/*z* = 387.4 (100%, [M + Na]^+^); anal. calcd. for C_14_H_24_N_2_S_2_O_5_ (364.48): C 46.14, H 6.64, N 7.69; found: C 45.96, H 6.90, N 7.36.

#### 4.2.65. N-(8-Hydroxyoctyl)-2-methylbenzene Sulfonamide (**32a**)

Applying GPA: from 2-methylbenzenesulfonyl chloride (500 mg, 2.62 mmol) and 8-amino-octanol (571 mg, 3.93 mmol): **32a** (766 mg, 98%); oil; R_f_ = 0.24 (petrolether/EtOAc, 2:3); UV–Vis: 223 nm (3.91); IR: ν = 3504*w*, 3295*w*, 2929*m*, 2856*m*, 1458*m*, 1315*s*, 1154*vs*, 1131*m*, 1066*m*, 876*vw*, 807*w*, 759*m*, 723*w*, 711*m*, 688*m*, 592*vs*, 541*m*, 489*m* cm^−1^; ^1^H NMR:δ = 7.80 (*dd*, *J* = 7.8, 1.4 Hz, 1H, 3-H), 7.62–7.55 (*m*, 1H, 6-H), 7.50 (*td*, *J* = 7.5, 1.4 Hz, 1H, 4-H), 7.41–7.34 (*m*, 2H, 5-H, NH), 4.29 (*t*, *J* = 5.1 Hz, 1H, OH), 3.35 (*td*, *J* = 6.6, 5.0 Hz, 2H, 15-H), 2.74 (*t*, *J* = 7.0 Hz, 2H, 8-H), 2.57 (*s*, 3H, 7-H), 1.41–1.26 (*m*, 4H, 9-H, 14-H), 1.25–1.07 (*m*, 8H, 10-H, 11-H, 12-H, 13-H) ppm; ^13^C NMR:δ = 139.4 (C-1), 136.9 (C-2), 132.9 (C-4), 132.7 (C-3), 128.8 (C-6), 126.6 (C-5), 61.2 (C-15), 42.7 (C-8), 32.9 (C-14), 29.4 (C-9), 29.2 (C-11), 29.0 (C-12), 26.3 (C-10), 25.8 (C-13), 20.2 (C-7) ppm; MS: *m*/*z* = 322.1 (100%, [M + Na]^+^); anal. calcd. for C_15_H_25_NSO_3_ (299.43): C 60.17, H 8.42, N 4.68; found: C 59.84, H 8.66, N 4.32.

#### 4.2.66. 8-[(2-Methylphenyl)sulfonamido]octyl Sulfamate (**32b**)

Applying GPB: from **32a** (300 mg, 1.00 mmol): **32b** (283 mg, 75%); oil; R_f_ = 0.76 (CHCl_3_/EtOAc, 2:3); UV–Vis: 270 nm (3.16); IR: ν = 3280*w*, 3114*vw*, 2931*w*, 2857*w*, 1563*w*, 1460*w*, 1361*m*, 1314*m*, 1178*s*, 1154*vs*, 1131*m*, 1066*m*, 1048*w*, 924*s*, 807*w*, 761*m*, 723*w*, 711*m*, 689*m*, 593*s*, 551*s*, 542*s*, 491*m* cm^−1^; ^1^H NMR (500 MHz, DMSO-*d*_6_): δ = 7.80 (*dd*, *J* = 7.8, 1.4 Hz, 1H, 3-H), 7.59 (*t*, *J* = 5.7 Hz, 1H, 6-H), 7.50 (*td*, *J* = 7.5, 1.4 Hz, 1H, 4-H), 7.40–7.34 (*m*, 4H, 5-H, NH, NH_2_), 3.99 (*t*, *J* = 6.5 Hz, 2H, 15-H), 2.75 (*td*, *J* = 7.0, 5.8 Hz, 2H, 8-H), 2.57 (*s*, 3H, 7-H), 1.62–1.54 (*m*, 2H, 14-H), 1.36–1.22 (*m*, 4H, 9-H, 13-H), 1.21–1.09 (*m*, 6H, 10-H, 11-H, 12-H) ppm; ^13^C NMR:δ = 138.9 (C-1), 136.4 (C-2), 132.4 (C-4), 132.3 (C-3), 128.3 (C-6), 126.1 (C-5), 69.0 (C-15), 42.2 (C-8), 29.0 (C-14), 28.3 (C-9), 28.3 (C-11), 28.3 (C-12), 25.8 (C-13), 24.9 (C-10), 19.8 (C-7) ppm; MS: *m*/*z* = 401.3 (100%, [M + Na]^+^); anal. calcd. for C_15_H_26_N_2_S_2_O_5_ (378.50): C 47.60, H 6.92, N 7.40; found: C 47.42, H 7.18, N 7.20.

#### 4.2.67. N-(2-Hydroxyethyl)-4-isopropylbenzene Sulfonamide (**33a**) [117867-88-6]

Applying GPA: from 4-isopropylbenzenesulfonyl chloride (500 mg, 2.29 mmol) and 2-amino-ethanol (209 mg, 3.43 mmol): **33a** (441 mg, 79%) [115,116,117] oil; R_f_ = 0.16 (petrolether/EtOAc, 2:3); UV–Vis: 228 nm (4.08); IR: ν = 3516*m*, 3168*w*, 2955*m*, 2891*w*, 2870*w*, 1599*w*, 1494*w*, 1464*w*, 1453*w*, 1433*w*, 1411*m*, 1354*vw*, 1323*s*, 1282*w*, 1261*w*, 1211*w*, 1187*w*, 1158*vs*, 1107*w*, 1090*m*, 1070*m*, 1051*s*, 1018*w*, 956*s*, 905*w*, 851*w*, 845*w*, 824*m*, 777*s*, 738*m*, 724*m*, 646*m*, 632*w*, 580*s*, 562*s*, 532*w*, 496*w*, 484*w*, 462*m*, 451*w* cm^−1^; ^1^H NMR:δ = 7.73–7.69 (*m*, 2H, 2-H, 2′-H), 7.53–7.43 (*m*, 3H, 3H-, 3′-H, NH), 4.66 (*s*, 1H, OH), 3.37 (*t*, *J* = 6.4 Hz, 2H, 8-H), 2.97 (*hept*, *J* = 6.9 Hz, 1H, 5-H), 2.77 (*t*, *J* = 6.4 Hz, 2H, 7-H), 1.22 (*d*, *J* = 6.9 Hz, 6H, 6-H, 6′-H) ppm; ^13^C NMR:δ = 153.0 (C-4), 138.0 (C-1), 127.0 (C-2), 126.6 (C-3), 59.9 (C-8), 45.1 (C-7), 33.3 (C-5), 23.5 (C-6) ppm; MS: *m*/*z* = 266.2 (100%, [M + Na]^+^); anal. calcd. for C_11_H_17_NSO_3_ (243.32): C 54.30, H 7.04, N 5.76; found: C 54.03, H 7.28, N 5.44.

#### 4.2.68. 2-[(4-Isopropylphenyl)sulfonamido]ethyl Sulfamate (**33b**)

Applying GPB: from **33a** (300 mg, 1.23 mmol): **33b** (241 mg, 61%); white solid; R_f_ = 0.59 (CHCl_3_/EtOAc, 2:3); m.p. = 81–82 °C; UV–Vis: 228 nm (4.03); IR: ν = 3276*m*, 2963*w*, 1598*w*, 1559*w*, 1411*w*, 1364*s*, 1319*s*, 1284*w*, 1179*s*, 1157*vs*, 1091*m*, 1054*w*, 1015*m*, 924*s*, 832*m*, 775*m*, 753*m*, 649*s*, 632*w*, 549*s*, 488*w*, 436*w* cm^−1^; ^1^H NMR:δ = 7.83 (*t*, *J* = 6.0 Hz, 1H, NH), 7.76–7.70 (*m*, 2H, 2-H, 2′-H), 7.53–7.44 (*m*, 4H, 3-H, 3′-H, NH_2_), 4.01 (*t*, *J* = 5.7 Hz, 2H, 8-H), 3.03 (*q*, *J* = 5.4 Hz, 2H, 7-H), 3.00–2.92 (*m*, 1H, 5-H), 1.22 (*d*, *J* = 6.9 Hz, 6H, 6-H, 6′-H) ppm; ^13^C NMR:δ = 153.3 (C-4), 137.7 (C-1), 127.2 (C-2), 126.6 (C-3), 67.6 (C-8), 41.6 (C-7), 33.4 (C-5), 23.5 (C-6) ppm; MS: *m*/*z* = 345.2 (100%, [M + Na]^+^); anal. calcd. for C_11_H_18_N_2_S_2_O_5_ (322.39): C 40.98, H 5.63, N 8.69; found: C 40.71, H 5.98, N 8.43.

#### 4.2.69. N-(3-Hydroxypropyl)-4-isopropylbenzene Sulfonamide (**34a**) [920113-99-1]

Applying GPA: from 4-isopropylbenzenesulfonyl chloride (500 mg, 2.29 mmol) and 3-amino-propanol (258 mg, 3.43 mmol): **34a** (558 mg, 95%); oil; R_f_ = 0.16 (petrolether/EtOAc, 2:3); UV–Vis: 228 nm (4.22); IR: ν = 3501*w*, 3278*w*, 2962*w*, 2874*w*, 1598*w*, 1464*w*, 1410*m*, 1386*w*, 1364*w*, 1317*s*, 1283*m*, 1156*vs*, 1091*s*, 1053*s*, 1016*w*, 1008*w*, 959*w*, 833*m*, 774*m*, 648*s*, 632*m*, 579*s*, 565*s*, 486*w* cm^−1^; ^1^H NMR:δ = 7.73–7.68 (*m*, 2H, 2-H, 2′-H), 7.48–7.40 (*m*, 3H, 3-H, 3′-H, NH), 4.40 (*t*, *J* = 5.1 Hz, 1H, OH), 3.37 (*td*, *J* = 6.2, 5.0 Hz, 2H, 9-H), 2.97 (*hept*, *J* = 7.0 Hz, 1H, 5-H), 2.77 (*td*, *J* = 7.3, 5.8 Hz, 2H, 7-H), 1.57–1.49 (*m*, 2H, 8-H), 1.22 (*d*, *J* = 6.9 Hz, 6H, 6-H, 6′-H) ppm; ^13^C NMR:δ = 153.0 (C-4), 137.9 (C-1), 127.0 (C-2), 126.6 (C-3), 58.1 (C-9), 40.0 (C-7), 33.3 (C-5), 32.4 (C-8), 23.5 (C-6) ppm; MS: *m*/*z* = 280.1 (100%, [M + Na]^+^); anal. calcd. for C_12_H_19_NSO_3_ (257.35): C 56.01, H 7.44, N 5.44; found: C 55.76, H 7.62, N 5.18.

#### 4.2.70. 3-[(4-Isopropylphenyl)sulfonamido]propyl Sulfamate (**34b**)

Applying GPB: from **34a** (200 mg, 0.78 mmol): **34b** (226 mg, 86%); white solid; R_f_ = 0.60 (CHCl_3_/EtOAc, 2:3); m.p. = 83–84 °C; UV–Vis: 228 nm (4.11); IR: ν = 3359*m*, 3285*m*, 3262*m*, 2963*w*, 1599*w*, 1565*w*, 1474*w*, 1468*w*, 1435*m*, 1402*m*, 1372*vs*, 1337*w*, 1311*s*, 1283*m*, 1255*w*, 1176*s*, 1157*vs*, 1111*w*, 1091*m*, 1068*m*, 1054*m*, 1039*m*, 1017*w*, 943*s*, 920*s*, 887*m*, 835*s*, 827*s*, 774*m*, 756*m*, 733*w*, 676*s*, 635*m*, 596*m*, 579*s*, 561*s*, 546*vs*, 520*s*, 486*m*, 426*m* cm^−1^; ^1^H NMR:δ = 7.73–7.68 (*m*, 2H, 2-H, 2′-H), 7.60 (*t*, *J* = 5.9 Hz, 1H, NH), 7.49–7.44 (*m*, 2H, 3-H, 3′-H), 7.41 (*s*, 2H, NH_2_), 4.03 (*t*, *J* = 6.3 Hz, 2H, 9-H), 2.98 (*hept*, *J* = 7.0 Hz, 1H, 5-H), 2.81 (*td*, *J* = 7.1, 5.8 Hz, 2H, 7-H), 1.77 (*p*, *J* = 6.7 Hz, 2H, 8-H), 1.22 (*d*, *J* = 6.9 Hz, 6H, 6-H, 6′-H) ppm; ^13^C NMR:δ = 153.2 (C-4), 137.7 (C-1), 127.3 (C-2), 126.6 (C-3), 66.5 (C-9), 39.2 (C-7), 33.3 (C-5), 28.8 (C-8), 23.5 (C-6) ppm; MS: *m*/*z* = 359.3 (100%, [M + Na]^+^); anal. calcd. for C_12_H_20_N_2_S_2_O_5_ (336.42): C 42.84, H 5.99, N 8.33; found: C 42.67, H 6.20, N 8.02.

#### 4.2.71. N-(4-Hydroxybutyl)-4-isopropylbenzene Sulfonamide (**35a**) [1082772-50-6]

Applying GPA: from 4-isopropylbenzenesulfonyl chloride (500 mg, 2.29 mmol) and 4-amino-butanol (306 mg, 3.43 mmol): **35a** (540 mg, 87%); oil; R_f_ = 0.14 (petrolether/EtOAc, 2:3); UV–Vis: 228 nm (4.32); IR: ν = 3501*w*, 3281*w*, 2961*m*, 2871*w*, 1598*w*, 1463*w*, 1410*m*, 1386*w*, 1364*w*, 1318*s*, 1283*w*, 1156*vs*, 1091*s*, 1053*s*, 1017*w*, 991*vw*, 833*m*, 773*w*, 735*w*, 648*s*, 632*m*, 581*s*, 565*s*, 488*w*, 471*w* cm^−1^; ^1^H NMR:δ = 7.72–7.68 (*m*, 2H, 2-H, 2′-H), 7.48–7.43 (*m*, 3H, 3-H, 3′-H, NH), 4.39–4.31 (*m*, 1H, OH), 3.37–3.27 (*m*, 2H, 10-H), 2.97 (*hept*, *J* = 6.9 Hz, 1H, 5-H), 2.75–2.67 (*m*, 2H, 7-H), 1.44–1.31 (*m*, 4H, 8-H, 9-H), 1.22 (*d*, *J* = 6.9 Hz, 6H, 6-H, 6′-H) ppm; ^13^C NMR:δ = 152.9 (C-4), 138.1 (C-1), 127.0 (C-2), 126.6 (C-3), 60.2 (C-10), 42.5 (C-7), 33.3 (C-5), 29.5 (C-9), 25.8 (C-8), 23.5 (C-6) ppm; MS: *m*/*z* = 294.3 (100%, [M + Na]^+^); anal. calcd. for C_13_H_21_NSO_3_ (271.38): C 57.54, H 7.80, N 5.16; found: C 57.21, H 8.04, N 4.87.

#### 4.2.72. 4-[(4-Isopropylphenyl)sulfonamido]butyl Sulfamate (**35b**)

Applying GPB: from **35a** (200 mg, 0.74 mmol): **35b** (233 mg, 90%); white solid; R_f_ = 0.61 (CHCl_3_/EtOAc, 2:3); m.p. = 95–97 °C; UV–Vis: 228 nm (4.16); IR: ν = 3356*m*, 3286*m*, 3262*m*, 2965*w*, 2879*w*, 1558*w*, 1481*w*, 1476*w*, 1431*w*, 1409*w*, 1398*w*, 1384*w*, 1366*s*, 1337*m*, 1320*s*, 1284*w*, 1203*w*, 1175*s*, 1157*vs*, 1143*m*, 1093*m*, 1063*m*, 1047*w*, 969*s*, 942*w*, 922*s*, 901*s*, 840*m*, 824*s*, 773*m*, 750*w*, 732*w*, 666*s*, 632*m*, 588*s*, 580*s*, 552*vs*, 513*m*, 505*m*, 415*w* cm^−1^; ^1^H NMR:δ = 7.73–7.68 (*m*, 2H, 2-H, 2′-H), 7.53 (*t*, *J* = 5.9 Hz, 1H, NH), 7.48–7.43 (*m*, 2H, 3-H, 3′-H), 7.38 (*s*, 2H, NH_2_), 3.96 (*t*, *J* = 6.3 Hz, 2H, 10-H), 2.98 (*hept*, *J* = 6.9 Hz, 1H, 5-H), 2.75 (*td*, *J* = 6.9, 6.0 Hz, 2H, 7-H), 1.66–1.56 (*m*, 2H, 9-H), 1.50–1.41 (*m*, 2H, 8-H), 1.22 (*d*, *J* = 6.9 Hz, 6H, 6-H, 6′-H) ppm; ^13^C NMR (126 MHz, DMSO-*d*_6_): δ = 153.0 (C-4), 138.0 (C-1), 127.1 (C-2), 126.6 (C-3), 68.5 (C-10), 42.0 (C-7), 33.3 (C-5), 25.6 (C-8), 25.4 (C-9), 23.5 (6) ppm; MS: *m*/*z* = 373.3 (100%, [M + Na]^+^); anal. calcd. for C_13_H_22_N_2_S_2_O_5_ (350.45): C 44.56, H 6.33, N 7.99; found: C 44.27, H 6.68, N 7.65.

#### 4.2.73. N-(5-Hydroxypentyl)-4-isopropylbenzene Sulfonamide (**36a**) [1925596-35-5]

Applying GPA: from 4-isopropylbenzenesulfonyl chloride (500 mg, 2.29 mmol) and 5-amino-pentanol (354 mg, 3.43 mmol): **36a** (604 mg, 93%); oil; R_f_ = 0.18 (petrolether/EtOAc, 2:3); UV–Vis: 228 nm (4.42); IR: ν = 3503*w*, 3278*w*, 2960*w*, 2936*m*, 2869*w*, 1598*w*, 1460*w*, 1410*m*, 1386*w*, 1364*w*, 1318*s*, 1283*w*, 1156*vs*, 1091*s*, 1053*m*, 1016*w*, 833*m*, 774*w*, 733*w*, 648*s*, 632*m*, 581*s*, 565*s* cm^−1^; ^1^H NMR:δ = 7.72–7.67 (*m*, 2H, 2-H, 2′-H), 7.48–7.42 (*m*, 3H, 3-H, 3′-H, NH), 4.30 (*t*, *J* = 5.1 Hz, 1H, OH), 3.35–3.29 (*m*, 2H, 11-H), 2.97 (*hept*, *J* = 7.0 Hz, 1H, 5-H), 2.70 (*td*, *J* = 6.8, 6.0 Hz, 2H, 7-H), 1.40–1.27 (*m*, 4H, 8-H, 10-H), 1.27–1.16 (*m*, 8H, 6-H, 6′-H, 9-H) ppm; ^13^C NMR:δ = 152.9 (C-4), 138.1 (C-1), 127.0 (C-2), 126.6 (C-3), 60.5 (C-11), 42.6 (C-7), 33.3 (C-5), 32.0 (C-10), 28.9 (C-8), 23.5 (C-6), 22.6 (C-9) ppm; MS: *m*/*z* = 308.1 (100%, [M + Na]^+^); anal. calcd. for C_14_H_23_NSO_3_ (285.40): C 58.92, H 8.12, N 4.91; found: C 58.76, H 8.47, N 4.59.

#### 4.2.74. 5-[(4-Isopropylphenyl)sulfonamido]pentyl Sulfamate (**36b**)

Applying GPB: from **36a** (200 mg, 0.7 mmol): **36b** (227 mg, 89%); white solid; R_f_ = 0.66 (CHCl_3_/EtOAc, 2:3); m.p. = 95–97 °C; UV–Vis: 228 nm (4.14); IR: ν = 3350*w*, 3280*m*, 2963*w*, 2946*w*, 2869*w*, 2859*vw*, 1475*vw*, 1432*w*, 1407*w*, 1400*w*, 1386*w*, 1366*s*, 1328*s*, 1310*m*, 1286*w*, 1191*w*, 1174*m*, 1157*vs*, 1142*m*, 1112*w*, 1094*m*, 1068*w*, 1058*w*, 1038*m*, 963*s*, 922*m*, 900*m*, 843*w*, 825*s*, 771*w*, 737*w*, 733*w*, 661*s*, 632*m*, 584*s*, 552*vs*, 525*m*, 517*m*, 501*w*, 444*w* cm^−1^; ^1^H NMR:δ = 7.72–7.68 (*m*, 2H, 2-H, 2′-H), 7.51–7.43 (*m*, 3H, 3-H, 3′-H, NH), 7.37 (*s*, 2H, NH_2_), 3.96 (*t*, *J* = 6.5 Hz, 2H, 11-H), 2.97 (*hept*, *J* = 7.0 Hz, 1H, 5-H), 2.72 (*q*, *J* = 6.5 Hz, 2H, 7-H), 1.55 (*p*, *J* = 6.7 Hz, 2H, 10-H), 1.43–1.34 (*m*, 2H, 8-H), 1.34–1.26 (*m*, 2H, 9-H), 1.22 (*d*, *J* = 6.9 Hz, 6H, 6-H, 6′-H) ppm; ^13^C NMR:δ = 153.0 (C-4), 138.0 (C-1), 127.1 (C-2), 126.6 (C-3), 68.8 (C-11), 42.3 (C-7), 28.5 (C-10), 27.8 (C-8), 23.5 (C-6), 22.2 (C-9) ppm; MS: *m*/*z* = 387.3 (100%, [M + Na]^+^); anal. calcd. for C_14_H_24_N_2_S_2_O_5_ (364.48): C 46.14, H 6.64, N 7.69; found: C 45.84, H 6.99, N 7.44.

#### 4.2.75. N-(6-Hydroxyhexyl)-4-isopropylbenzene Sulfonamide (**37a**) [1912844-40-6]

Applying GPA: from 4-isopropylbenzenesulfonyl chloride (500 mg, 2.29 mmol) and 6-amino-hexanol (402 mg, 3.43 mmol): **37a** (612 mg, 89%); oil; R_f_ = 0.20 (petrolether/EtOAc, 2:3); UV–Vis: 228 nm (4.08); IR: ν = 3505*w*, 3282*w*, 2960*w*, 2933*m*, 2864*w*, 1598*w*, 1462*w*, 1410*m*, 1386*w*, 1364*w*, 1319*s*, 1283*w*, 1157*vs*, 1092*s*, 1073*m*, 1053*m*, 1016*w*, 891*vw*, 833*m*, 773*w*, 727*w*, 649*s*, 633*m*, 581*s*, 565*s*, 493*w*, 456*w* cm^−1^; ^1^H NMR:δ = 7.73–7.68 (*m*, 2H, 2-H, 2′-H), 7.48–7.43 (*m*, 3H, 3-H, 3′-H, NH), 4.32–4.27 (*m*, 1H, OH), 3.34 (*td*, *J* = 6.3, 2.9 Hz, 2H, 12-H), 2.97 (*hept*, *J* = 6.9 Hz, 1H, 5-H), 2.71 (*td*, *J* = 7.0, 5.9 Hz, 2H, 7-H), 1.38–1.29 (*m*, 4H, 8-H, 11-H), 1.22 (*d*, *J* = 6.9 Hz, 6H, 6-H, 6′-H), 1.20–1.15 (*m*, 4H, 9-H, 10-H) ppm; ^13^C NMR:δ = 152.9 (C-4), 138.2 (C-1), 127.0 (C-2), 126.6 (C-3), 60.6 (C-12), 42.5 (C-7), 33.3 (C-5), 32.3 (C-11), 29.0 (C-8), 25.9 (C-10), 25.0 (C-9), 23.5 (C-6) ppm; MS: *m*/*z* = 322.1 (100%, [M + Na]^+^); anal. calcd. for C_15_H_25_NSO_3_ (299.43): C 60.17, H 8.42, N 4.68; found: C 59.76, H 8.72, N 4.43.

#### 4.2.76. 6-[(4-Isopropylphenyl)sulfonamido]hexyl Sulfamate (**37b**)

Applying GPB: from **37a** (300 mg, 1.00 mmol): **37b** (260 mg, 69%); white solid; R_f_ = 0.72 (CHCl_3_/EtOAc, 2:3); m.p. = 102–103 °C; UV–Vis: 228 nm (4.19); IR: ν = 3384*w*, 3278*m*, 2962*m*, 2933*w*, 2860*w*, 1600*w*, 1544*w*, 1477*w*, 1469*w*, 1420*m*, 1396*w*, 1374*s*, 1320*s*, 1312*s*, 1283*w*, 1179*s*, 1157*vs*, 1146*s*, 1112*w*, 1092*m*, 1065*m*, 1054*m*, 1003*s*, 977*s*, 951*w*, 924*m*, 907*m*, 873*m*, 845*w*, 830*m*, 814*s*, 802*s*, 774*m*, 757*w*, 697*s*, 647*m*, 633*m*, 579*s*, 564*s*, 551*vs*, 537*s*, 505*m*, 484*w*, 456*w* cm^−1^; ^1^H NMR:δ = 7.72–7.67 (*m*, 2H, 2-H, 2′-H), 7.49–7.43 (*m*, 3H, 3-H, 3′-H, NH), 7.37 (*s*, 2H, NH_2_), 3.97 (*t*, *J* = 6.5 Hz, 2H, 12-H), 2.97 (*hept*, *J* = 6.9 Hz, 1H, 5-H), 2.71 (*q*, *J* = 6.7 Hz, 2H, 7-H), 1.56 (*p*, *J* = 6.6 Hz, 2H, 11-H), 1.35 (*p*, *J* = 6.8 Hz, 2H, 8-H), 1.28–1.19 (*m*, 10H, 6-H, 6′-H, 9-H, 10-H) ppm; ^13^C NMR:δ = 153.0 (C-4), 138.1 (C-1), 127.0 (C-2), 126.6 (C-3), 68.9 (C-12), 42.4 (C-7), 33.3 (C-5), 28.8 (C-11), 28.2 (C-8), 25.5 (C-10), 24.6 (C-9), 23.5 (C-6) ppm; MS: *m*/*z* = 401.2 (100%, [M + Na]^+^); anal. calcd. for C_15_H_26_N_2_S_2_O_5_ (378.50): C 47.60, H 6.92, N 7.40; found: C 47.36, H 7.20, N 7.05.

#### 4.2.77. N-(7-Hydroxyheptyl)-4-isopropylbenzene Sulfonamide (**38a**)

Applying GPA: from 4-isopropylbenzenesulfonyl chloride (500 mg, 2.29 mmol) and 7-amino-heptanol (450 mg, 3.43 mmol): **38a** (658 mg, 92%); oil; R_f_ = 0.26 (petrolether/EtOAc, 2:3); UV–Vis: 228 nm (4.11); IR: ν = 3503*w*, 3281*w*, 2960*w*, 2931*m*, 2859*m*, 1598*w*, 1495*vw*, 1463*w*, 1410*m*, 1386*w*, 1364*w*, 1319*s*, 1283*w*, 1157*vs*, 1092*s*, 1053*m*, 1016*w*, 891*vw*, 833*m*, 773*w*, 724*w*, 648*s*, 633*m*, 581*s*, 565*s*, 493*w*, 485*w* cm^−1^; ^1^H NMR:δ = 7.70–7.66 (*m*, 2H, 2-H, 2′-H), 7.45–7.40 (*m*, 3H, 3-H, 3′-H, NH), 3.97 (*s*, 1H, OH), 3.33 (*t*, *J* = 6.6 Hz, 2H, 13-H), 2.94 (*hept*, *J* = 6.9 Hz, 1H, 5-H), 2.69 (*td*, *J* = 7.0, 5.9 Hz, 2H, 7-H), 1.39–1.27 (*m*, 4H, 8-H, 12-H), 1.19 (*d*, *J* = 6.9 Hz, 6H, 6-H, 6′-H), 1.17–1.10 (*m*, 6H, 9-H, 10-H, 11-H) ppm; ^13^C NMR (126 MHz, DMSO-*d*_6_): δ = 152.9 (C-4), 138.2 (C-1), 127.0 (C-2), 126.6 (C-3), 60.7 (C-13), 42.5 (C-7), 33.3 (C-5), 32.4 (C-12), 28.9 (C-8), 28.4 (C-10), 26.1 (C-9), 25.3 (C-11), 23.5 (C-6) ppm; MS: *m*/*z* = 336.2 (100%, [M + Na]^+^); anal. calcd. for C_16_H_27_NSO_3_ (313.46): C 61.31, H 8.68, N 4.47; found: C 61.00, H 8.95, N 4.18.

#### 4.2.78. 7-[(4-Isopropylphenyl)sulfonamido]heptyl Sulfamate (**38b**)

Applying GPB: from **38a** (200 mg, 0.67 mmol): **38b** (230 mg, 90%); white solid; R_f_ = 0.75 (CHCl_3_/EtOAc, 2:3); m.p. = 70–72 °C; UV–Vis: 228 nm (4.10); IR: ν = 3382*w*, 3274*m*, 2966*w*, 2921*m*, 2911*w*, 2852*w*, 1605*w*, 1543*w*, 1475*w*, 1467*w*, 1420*m*, 1392*w*, 1376*vs*, 1320*s*, 1281*w*, 1181*s*, 1159*s*, 1110*w*, 1090*m*, 1065*w*, 1056*m*, 1040*m*, 1018*w*, 1000*m*, 978*s*, 945*w*, 926*m*, 892*m*, 852*w*, 843*w*, 831*m*, 815*s*, 776*m*, 762*w*, 733*m*, 698*s*, 645*m*, 639*w*, 580*s*, 566*s*, 551*vs*, 530*s*, 510*m*, 486*w*, 477*w*, 445*w* cm^−1^; ^1^H NMR:δ = 7.72–7.68 (*m*, 2H, 2-H, 2′-H), 7.48–7.43 (*m*, 3H, 3-H, 3′-H, NH), 7.37 (*s*, 2H, NH_2_), 3.98 (*t*, *J* = 6.5 Hz, 2H, 13-H), 2.97 (*hept*, *J* = 6.9 Hz, 1H, 5-H), 2.71 (*q*, *J* = 6.8 Hz, 2H, 7-H), 1.63–1.53 (*m*, 2H, 12-H), 1.38–1.31 (*m*, 2H, 8-H), 1.22 (*d*, *J* = 6.9 Hz, 6H, 6-H, 6′-H), 1.30–1.14 (*m*, 6H, 9-H, 10-H, 11-H) ppm; ^13^C NMR (126 MHz, DMSO-*d*_6_): δ = 153.0 (C-4), 138.1 (C-1), 127.0 (C-2), 126.6 (C-3), 68.9 (C-13), 42.5 (C-7), 33.3 (C-5), 28.9 (C-12), 28.2 (C-8), 28.0 (C-10), 25.8 (C-11), 24.9 (C-9), 23.5 (C-6) ppm; MS: *m*/*z* = 415.3 (100%, [M + Na]^+^); anal. calcd. for C_16_H_28_N_2_S_2_O_5_ (392.53): C 48.96, H 7.19, N 7.14; found: C 48.73, H 7.41, N 6.87.

#### 4.2.79. N-(8-Hydroxyoctyl)-4-isopropylbenzene Sulfonamide (**39a**)

Applying GPA: from 4-isopropylbenzenesulfonyl chloride (500 mg, 2.29 mmol) and 8-amino-octanol (498 mg, 3.43 mmol): **39a** (721 mg, 96%); oil; R_f_ = 0.28 (petrolether/EtOAc, 2:3); UV–Vis: 228 nm (4.23); IR: ν = 3500*w*, 3281*w*, 2929*m*, 2857*m*, 1599*w*, 1463*w*, 1410*w*, 1364*w*, 1320*m*, 1283*m*, 1158*s*, 1092*m*, 1053*m*, 1017*w*, 924*w*, 892*w*, 833*m*, 809*w*, 773*w*, 722*m*, 649*m*, 582*w*, 566*m*, 514*w*, 488*w* cm^−1^; ^1^H NMR (500 MHz, DMSO-*d*_6_): δ = 7.71–7.68 (*m*, 2H, 2-H, 2′-H), 7.47–7.43 (*m*, 3H, 3-H, 3′-H, NH), 4.30 (*t*, *J* = 5.1 Hz, 1H, OH), 3.36 (*td*, *J* = 6.5, 5.1 Hz, 2H, 14-H), 2.97 (*hept*, *J* = 6.9 Hz, 1H, 5-H), 2.71 (*td*, *J* = 7.0, 5.8 Hz, 2H, 7-H), 1.41–1.29 (*m*, 4H, 8-H, 13-H), 1.21 (*d*, *J* = 6.9 Hz, 6H, 6-H, 6′-H), 1.20–1.11 (*m*, 8H, 9-H, 10-H, 11-H, 12-H) ppm; ^13^C NMR (126 MHz, DMSO-*d*_6_): δ = 152.9 (C-4), 138.2 (C-1), 127.0 (C-2), 126.6 (C-3), 60.7 (C-14), 42.5 (C-7), 33.3 (C-5), 32.5 (C-13), 28.9 (C-8), 28.8 (C-12), 28.6 (C-9), 25.9 (C-11), 25.4 (C-10), 23.5 (C-6) ppm; MS: *m*/*z* = 350.2 (100%, [M + Na]^+^); anal. calcd. for C_17_H_29_NSO_3_ (327.48): C 62.35, H 8.93, N 4.28; found: C 62.03, H 9.22, N 3.93.

#### 4.2.80. 8-[(4-Isopropylphenyl)sulfonamido]octyl Sulfamate (**39b**)

Applying GPB: from **39a** (200 mg, 0.61 mmol): **39b** (220 mg, 89%); white solid; R_f_ = 0.80 (CHCl_3_/EtOAc, 2:3); m.p. = 69–70 °C; UV–Vis: 229 nm (4.08); IR: ν = 3385*w*, 3278*m*, 2961*w*, 2929*m*, 2915*w*, 2853*w*, 1600*w*, 1543*w*, 1477*w*, 1468*w*, 1419*m*, 1396*w*, 1376*vs*, 1319*s*, 1283*w*, 1182*s*, 1158*s*, 1112*w*, 1093*m*, 1069*w*, 1055*m*, 1038*m*, 1016*w*, 1001*m*, 977*s*, 945*w*, 925*m*, 894*m*, 858*w*, 845*w*, 830*m*, 814*s*, 775*m*, 762*w*, 731*m*, 699*s*, 648*m*, 633*w*, 582*s*, 564*s*, 550*vs*, 532*s*, 513*m*, 485*w*, 477*w*, 443*w* cm^−1^; ^1^H NMR:δ = 7.72–7.66 (*m*, 2H, 2-H, 2′-H), 7.47–7.43 (*m*, 3H, 3-H, 3′-H, NH), 7.37 (*s*, 2H, NH_2_), 3.99 (*t*, *J* = 6.5 Hz, 2H, 14-H), 2.97 (*hept*, *J* = 6.9 Hz, 1H, 5-H), 2.75–2.66 (*m*, 2H, 7-H), 1.64–1.54 (*m*, 2H, 13-H), 1.40–1.11 (*m*, 10H, 8-H, 9-H, 10-H, 11-H, 12-H), 1.22 (*d*, *J* = 6.9 Hz, 6H, 6-H, 6′-H) ppm; ^13^C NMR (126 MHz, DMSO-*d*_6_): δ = 153.0 (C-4), 138.2 (C-1), 127.0 (C-2), 126.6 (C-3), 69.0 (C-14), 42.5 (C-7), 33.3 (C-5), 28.9 (C-13), 28.4 (C-11), 28.3 (C-8), 28.3 (C-10), 25.9 (C-9), 24.9 (C-12), 23.5 (C-6) ppm; MS: *m*/*z* = 429.4 (100%, [M + Na]^+^); anal. calcd. for C_17_H_30_N_2_S_2_O_5_ (406.56): C 50.22, H 7.44, N 6.89; found: C 49.93, H 7.71, N 6.65.

#### 4.2.81. N-(2-Hydroxyethyl)-3-isopropylbenzene Sulfonamide (**40a**)

Applying GPA: from 3-isopropylbenzenesulfonyl chloride (500 mg, 2.29 mmol) and 2-amino-ethanol (209 mg, 3.43 mmol): **40a** (548 mg, 98%); oil; R_f_ = 0.16 (petrolether/EtOAc, 2:3); UV–Vis: 222 nm (3.94); IR: ν = 3492*w*, 3275*w*, 2962*w*, 2874*w*, 1478*w*, 1461*w*, 1428*m*, 1386*w*, 1365*w*, 1323*s*, 1306*s*, 1217*w*, 1156*vs*, 1144*s*, 1090*m*, 1052*m*, 998*vw*, 949*m*, 902*w*, 798*m*, 695*s*, 623*m*, 586*vs*, 564*m*, 542*m*, 517*m*, 470*m*, 460*w* cm^−1^; ^1^H NMR:δ = 7.69–7.66 (*m*, 1H, 5-H), 7.64–7.60 (*m*, 1H, 6-H), 7.55 (*t*, *J* = 5.9 Hz, 1H, NH), 7.53–7.48 (*m*, 2H, 2-H, 4-H), 4.67 (*t*, *J* = 5.6 Hz, 1H, OH), 3.38 (*td*, *J* = 6.3, 5.5 Hz, 2H, 10-H), 2.98 (*hept*, *J* = 6.9 Hz, 1H, 7-H), 2.80 (*q*, *J* = 6.2 Hz, 2H, 9-H), 1.22 (*d*, *J* = 6.9 Hz, 6H, 8-H, 8′-H) ppm; ^13^C NMR (126 MHz, DMSO-*d*_6_): δ = 149.6 (C-3), 140.7 (C-1), 130.4 (C-4), 129.1 (C-5), 124.1 (C-2), 124.0 (C-6), 59.9 (C-10), 45.1 (C-9), 33.3 (C-7), 23.6 (C-8) ppm; MS: *m*/*z* = 266.2 (100%, [M + Na]^+^); anal. calcd. for C_11_H_17_NSO_3_ (243.32): C 54.30, H 7.04, N 5.76; found: C 53.99, H 7.36, N 5.41.

#### 4.2.82. 2-[(3-Isopropylphenyl)sulfonamido]ethyl Sulfamate (**40b**)

Applying GPB: from **40a** (200 mg, 0.82 mmol): **40b** (238 mg, 90%); oil; R_f_ = 0.64 (CHCl_3_/EtOAc, 2:3); UV–Vis: 269 nm (2.95); IR: ν = 3275*m*, 2963*w*, 2873*vw*, 1558*w*, 1479*w*, 1461*w*, 1430*w*, 1364*s*, 1325*s*, 1307*s*, 1179*s*, 1157*vs*, 1101*m*, 1091*m*, 1070*w*, 1021*m*, 998*w*, 924*s*, 798*m*, 757*m*, 694*s*, 624*m*, 585*vs*, 549*s*, 491*w*, 445*w*, 432*w* cm^−1^; ^1^H NMR:δ = 7.88 (*t*, *J* = 6.0 Hz, 1H, NH), 7.69–7.66 (*m*, 1H, 5-H), 7.63 (*dt*, *J* = 6.7, 2.0 Hz, 1H, 6-H), 7.56–7.47 (*m*, 4H, 2-H, 4-H, NH_2_), 4.01 (*t*, *J* = 5.7 Hz, 2H, 10-H), 3.05 (*q*, *J* = 5.0, 4.3 Hz, 2H, 9-H), 3.03–2.94 (*m*, 1H, 7-H), 1.23 (*d*, *J* = 6.9 Hz, 6H, 8-H, 8′-H) ppm; ^13^C NMR (126 MHz, DMSO-*d*_6_): δ = 149.7 (C-3), 140.3 (C-1), 130.6 (C-4), 129.3 (C-5), 124.1 (C-2), 124.0 (C-6), 67.6 (C-10), 41.6 (C-9), 33.3 (C-7), 23.6 (C-8) ppm; MS: *m*/*z* = 345.3 (100%, [M + Na]^+^); anal. calcd. for C_11_H_18_N_2_S_2_O_5_ (322.39): C 40.98, H 5.63, N 8.69; found: C 40.66, H 6.01, N 8.40.

#### 4.2.83. N-(3-Hydroxypropyl)-3-isopropylbenzene Sulfonamide (**41a**)

Applying GPA: from 3-isopropylbenzenesulfonyl chloride (500 mg, 2.29 mmol) and 3-amino-propanol (258 mg, 3.43 mmol): **41a** (562 mg, 95%); oil; R_f_ = 0.16 (petrolether/EtOAc, 2:3); UV–Vis: 222 nm (3.91); IR: ν = 3496*w*, 3279*w*, 2962*m*, 2875*w*, 1478*w*, 1462*w*, 1426*m*, 1386*w*, 1365*w*, 1323*m*, 1305*s*, 1217*w*, 1156*vs*, 1144*s*, 1084*m*, 1068*s*, 1008*w*, 998*w*, 960*w*, 904*w*, 871*w*, 799*m*, 696*s*, 623*m*, 587*vs*, 530*m*, 488*w* cm^−1^; ^1^H NMR:δ = 7.67–7.64 (*m*, 1H, 5-H), 7.62–7.58 (*m*, 1H, 6-H), 7.54–7.45 (*m*, 3H, 2-H, 4-H, NH), 4.39 (*t*, *J* = 5.1 Hz, 1H, OH), 3.36 (*td*, *J* = 6.3, 5.0 Hz, 2H, 11-H), 2.99 (*hept*, *J* = 6.9 Hz, 1H, 7-H), 2.79 (*td*, *J* = 7.1, 5.2 Hz, 2H, 9-H), 1.57–1.47 (*m*, 2H, 10-H), 1.23 (*d*, *J* = 6.9 Hz, 6H, 8-H, 8′-H) ppm; ^13^C NMR:δ = 149.6 (C-3), 140.5 (C-1), 130.4 (C-4), 129.1 (C-5), 124.1 (C-2), 124.0 (C-6), 58.1 (C-11), 40.0 (C-9), 33.3 (C-7), 32.3 (C-10), 23.6 (C-8) ppm; MS: *m*/*z* = 280.2 (100%, [M + Na]^+^); anal. calcd. for C_12_H_19_NSO_3_ (257.35): C 56.01, H 7.44, N 5.44; found: C 55.71, H 7.73, N 5.18.

#### 4.2.84. 3-[(3-Isopropylphenyl)sulfonamido]propyl Sulfamate (**41b**)

Applying GPB: from **41a** (200 mg, 0.78 mmol): **41b** (176 mg, 67%); oil; R_f_ = 0.64 (CHCl_3_/EtOAc, 2:3); UV–Vis: 269 nm (2.90); IR: ν = 3273*m*, 2964*w*, 2874*w*, 1560*w*, 1478*w*, 1464*w*, 1427*w*, 1364*s*, 1324*s*, 1306*s*, 1217*w*, 1177*s*, 1156*vs*, 1145*s*, 1090*m*, 1070*w*, 1051*w*, 940*s*, 824*w*, 798*m*, 738*w*, 695*s*, 623*m*, 585*vs*, 550*s*, 494*m* cm^−1^; ^1^H NMR:δ = 7.69–7.63 (*m*, 2H, 5-H, NH), 7.63–7.59 (*m*, 1H, 6-H), 7.56–7.49 (*m*, 2H, 2-H, 4-H), 7.41 (*s*, 2H, NH_2_), 4.03 (*t*, *J* = 6.3 Hz, 2H, 11-H), 3.00 (*hept*, *J* = 6.9 Hz, 1H, 7-H), 2.83 (*q*, *J* = 6.7 Hz, 2H, 9-H), 1.80–1.72 (*m*, 2H, 10-H), 1.23 (*d*, *J* = 6.9 Hz, 6H, 8-H, 8′-H) ppm; ^13^C NMR (126 MHz, DMSO-*d*_6_): δ = 149.7 (C-3), 140.3 (C-1), 130.5 (C-4), 129.2 (C-5), 124.1 (C-2), 124.0 (C-6), 66.5 (C-11), 39.2 (C-9), 33.3 (C-7), 28.7 (C-10), 23.6 (C-8) ppm; MS: *m*/*z* = 359.3 (100%, [M + Na]^+^); anal. calcd. for C_12_H_20_N_2_S_2_O_5_ (336.42): C 42.84, H 5.99, N 8.33; found: C 42.57, H 6.21, N 8.02.

#### 4.2.85. N-(4-Hydroxybutyl)-3-isopropylbenzene Sulfonamide (**42a**)

Applying GPA: from 3-isopropylbenzenesulfonyl chloride (500 mg, 2.29 mmol) and 4-amino-butanol (306 mg, 3.43 mmol): **42a** (593 mg, 95%); oil; R_f_ = 0.18 (petrolether/EtOAc, 2:3); UV–Vis: 223 nm (3.91); IR: ν = 3500*w*, 3277*w*, 2961*w*, 2871*w*, 1478*w*, 1462*w*, 1427*m*, 1386*w*, 1365*w*, 1323*m*, 1305*s*, 1217*w*, 1156*s*, 1144*s*, 1087*m*, 1067*m*, 1054*m*, 1034*w*, 998*w*, 904*w*, 866*vw*, 798*m*, 737*w*, 696*s*, 623*m*, 587*vs*, 564*m*, 517*m*, 493*w*, 477*w*, 464*w* cm^−1^; ^1^H NMR:δ = 7.66–7.64 (*m*, 1H, 5-H), 7.62–7.58 (*m*, 1H, 6-H), 7.54–7.47 (*m*, 3H, 2-H, 4-H, NH), 4.35 (*t*, *J* = 5.1 Hz, 1H, OH), 3.35–3.28 (*m*, 2H, 12-H), 2.99 (*hept*, *J* = 6.9 Hz, 1H, 7-H), 2.78–2.69 (*m*, 2H, 9-H), 1.44–1.31 (*m*, 4H, 10-H, 11-H), 1.22 (*d*, *J* = 6.9 Hz, 6H, 8-H, 8′-H) ppm; ^13^C NMR (126 MHz, DMSO-*d*_6_): δ = 149.6 (C-3), 140.7 (C-1), 130.3 (C-4), 129.1 (C-5), 124.0 (C-2), 124.0 (C-6), 60.2 (C-12), 42.5 (C-9), 33.3 (C-7), 29.5 (C-11), 25.8 (C-10), 23.6 (C-8) ppm; MS: *m*/*z* = 294.3 (100%, [M + Na]^+^); anal. calcd. for C_13_H_21_NSO_3_ (271.38): C 57.54, H 7.80, N 5.16; found: C 57.27, H 8.05, N 4.92.

#### 4.2.86. 4-[(3-Isopropylphenyl)sulfonamido]butyl Sulfamate (**42b**)

Applying GPB: from **42a** (200 mg, 0.68 mmol): **42b** (240 mg, 93%); oil; R_f_ = 0.64 (CHCl_3_/EtOAc, 2:3); UV–Vis: 269 nm (3.01); IR: ν = 3274*m*, 2963*w*, 2874*w*, 1562*w*, 1478*w*, 1463*w*, 1452*w*, 1428*w*, 1364*m*, 1324*s*, 1306*s*, 1268*w*, 1217*vw*, 1178*s*, 1156*vs*, 1145*s*, 1089*m*, 1069*m*, 1051*w*, 997*w*, 922*s*, 800*m*, 735*w*, 696*s*, 626*m*, 587*vs*, 551*s* cm^−1^; ^1^H NMR:δ = 7.67–7.64 (*m*, 1H, 5-H), 7.63–7.56 (*m*, 2H, 6-H, NH), 7.55–7.48 (*m*, 2H, 2-H, 4-H), 7.38 (*s*, 2H, NH_2_), 3.95 (*t*, *J* = 6.3 Hz, 2H, 12-H), 2.99 (*hept*, *J* = 6.9 Hz, 1H, 7-H), 2.81–2.72 (*m*, 2H, 9-H), 1.66–1.55 (*m*, 2H, 11-H), 1.49–1.39 (*m*, 2H, 10-H), 1.23 (*d*, *J* = 7.0 Hz, 6H, 8-H, 8′-H) ppm; ^13^C NMR (126 MHz, DMSO-*d*_6_): δ = 149.6 (C-3), 140.6 (C-1), 130.4 (C-4), 129.2 (C-5), 124.0 (C-2), 123.9 (C-6), 68.5 (C-12), 42.0 (C-9), 33.3 (C-7), 25.6 (C-10), 25.4 (C-11), 23.6 (C-8) ppm; MS: *m*/*z* = 373.3 (100%, [M + Na]^+^); anal. calcd. for C_13_H_22_N_2_S_2_O_5_ (350.45): C 44.56, H 6.33, N 7.99; found: C 44.18, H 6.67, N 7.68.

#### 4.2.87. N-(5-Hydroxypentyl)-3-isopropylbenzene Sulfonamide (**43a**)

Applying GPA: from 3-isopropylbenzenesulfonyl chloride (500 mg, 2.29 mmol) and 5-amino-pentanol (354 mg, 3.43 mmol): **43a** (611 mg, 94%); oil; R_f_ = 0.20 (petrolether/EtOAc, 2:3; UV–Vis: 223 nm (4.03); IR: ν = 3503*w*, 3279*w*, 2960*w*, 2936*w*, 2868*w*, 1478*w*, 1460*w*, 1427*w*, 1386*w*, 1365*w*, 1323*m*, 1305*s*, 1217*w*, 1156*s*, 1144*s*, 1087*m*, 1069*m*, 1050*m*, 998*w*, 905*w*, 799*m*, 730*w*, 696*s*, 623*m*, 587*vs*, 564*m*, 526*m*, 500*m*, 469*w*, 465*w*, 458*w*, 449*w* cm^−1^; ^1^H NMR:δ = 7.66–7.64 (*m*, 1H, 5-H), 7.62–7.58 (*m*, 1H, 6-H), 7.53–7.47 (*m*, 3H, 2-H, 4-H, NH), 4.30 (*t*, *J* = 5.1 Hz, 1H, OH), 3.34–3.29 (*m*, 2H, 13-H), 2.99 (*hept*, *J* = 6.9 Hz, 1H, 7-H), 2.77–2.67 (*m*, 2H, 9-H), 1.39–1.27 (*m*, 4H, 10-H, 12-H), 1.22 (*d*, *J* = 6.9 Hz, 8H, 8-H, 8′-H, 11-H) ppm; ^13^C NMR:δ = 149.6 (C-3), 140.7 (C-1), 130.3 (C-4), 129.1 (C-5), 124.0 (C-2), 124.0 (C-6), 60.5 (C-13), 42.6 (C-9), 33.3 (C-7), 32.0 (C-12), 28.8 (C-10), 23.6 (C-8), 22.6 (C-11) ppm; MS: *m*/*z* = 308.1 (100%, [M + Na]^+^); anal. calcd. for C_14_H_23_NSO_3_ (285.40): C 58.92, H 8.12, N 4.91; found: C 58.66, H 8.32, N 4.63.

#### 4.2.88. 5-[(3-Isopropylphenyl)sulfonamido]pentyl Sulfamate (**43b**)

Applying GPB: from **43a** (200 mg, 0.70 mmol): **43b** (160 mg, 63%); oil; R_f_ = 0.65 (CHCl_3_/EtOAc, 2:3); UV–Vis: 269 nm (2.99); IR: ν = 3275*m*, 2962*w*, 2871*w*, 1561*w*, 1478*w*, 1463*w*, 1428*w*, 1363*s*, 1323*s*, 1305*s*, 1217*vw*, 1176*s*, 1156*vs*, 1089*m*, 1070*m*, 1032*w*, 998*w*, 919*s*, 816*m*, 799*m*, 769*w*, 728*w*, 696*s*, 624*m*, 587*vs*, 551*s* cm^−1^; ^1^H NMR:δ = 7.66–7.64 (*m*, 1H, 5-H), 7.63–7.57 (*m*, 1H, 6-H), 7.56–7.47 (*m*, 3H, 2-H, 4-H, NH), 7.37 (*s*, 2H, NH_2_), 3.95 (*t*, *J* = 6.5 Hz, 2H, 13-H), 2.99 (*hept*, *J* = 7.0 Hz, 1H, 7-H), 2.73 (*q*, *J* = 6.6 Hz, 2H, 9-H), 1.55 (*p*, *J* = 6.7 Hz, 2H, 12-H), 1.43–1.34 (*m*, 2H, 10-H), 1.33–1.26 (*m*, 2H, 11-H), 1.23 (*d*, *J* = 6.9 Hz, 6H, 8-H, 8′-H) ppm; ^13^C NMR:δ = 149.6 (C-3), 140.6 (C-1), 130.4 (C-4), 129.2 (C-5), 124.0 (C-2), 124.0 (C-6), 68.8 (C-13), 42.3 (C-9), 33.3 (C-7), 28.5 (C-12), 27.8 (C-10), 23.6 (C-8), 22.2 (C-11) ppm; MS: *m*/*z* = 387.4 (100%, [M + Na]^+^); anal. calcd. for C_14_H_24_N_2_S_2_O_5_ (364.48): C 46.14, H 6.64, N 7.69; found: C 45.86, H 6.98, N 7.41.

#### 4.2.89. N-(6-Hydroxyhexyl)-3-isopropylbenzene Sulfonamide (**44a**)

Applying GPA: from 3-isopropylbenzenesulfonyl chloride (500 mg, 2.29 mmol) and 6-amino-hexanol (402 mg, 3.43 mmol): **44a** (658 mg, 96%); oil; R_f_ = 0.24 (petrolether/EtOAc, 2:3); UV–Vis: 223 nm (3.88); IR: ν 3502*w*, 3281*w*, 2960*w*, 2933*m*, 2863*w*, 1598*vw*, 1478*w*, 1462*w*, 1427*w*, 1386*w*, 1365*w*, 1323*m*, 1305*s*, 1217*vw*, 1156*s*, 1144*s*, 1087*m*, 1069*m*, 1051*m*, 999*w*, 904*w*, 798*m*, 726*w*, 696*s*, 624*m*, 587*vs*, 563*m*, 529*m*, 454*w* cm^−1^; ^1^H NMR:δ = 7.66–7.64 (*m*, 1H, 5-H), 7.62–7.57 (*m*, 1H, 6-H), 7.53–7.46 (*m*, 3H, 2-H, 4-H, NH), 4.29 (*t*, *J* = 5.1 Hz, 1H, OH), 3.33 (*td*, *J* = 6.5, 5.1 Hz, 2H, 14-H), 2.99 (*hept*, *J* = 6.9 Hz, 1H, 7-H), 2.77–2.69 (*m*, 2H, 9-H), 1.38–1.28 (*m*, 4H, 10-H, 13-H), 1.22 (*d*, *J* = 6.9 Hz, 6H, 8-H, 8′-H), 1.21–1.14 (*m*, 4H, 11-H, 12-H) ppm; ^13^C NMR:δ = 149.5 (C-3), 140.7 (C-1), 130.3 (C-4), 129.1 (C-5), 124.0 (C-2), 124.0 (C-6), 60.6 (C-14), 42.5 (C-9), 33.3 (C-7), 32.3 (C-13), 29.0 (C-10), 25.9 (C-12), 25.0 (C-11), 23.6 (C-8) ppm; MS: *m*/*z* = 322.1 (100%, [M + Na]^+^); anal. calcd. for C_15_H_25_NSO_3_ (299.43): C 60.17, H 8.42, N 4.68; found: C 59.84, H 8.76, N 4.37.

#### 4.2.90. 6-[(3-Isopropylphenyl)sulfonamido]hexyl Sulfamate (**44b**)

Applying GPB: from **44a** (300 mg, 1.00 mmol): **44b** (260 mg, 69%); oil; R_f_ = 0.66 (CHCl_3_/EtOAc, 2:3); UV–Vis: 269 nm (2.99); IR: ν = 3276*m*, 2961*w*, 2866*w*, 1561*w*, 1478*w*, 1463*w*, 1428*w*, 1363*s*, 1323*s*, 1306*s*, 1217*vw*, 1177*s*, 1156*vs*, 1145*s*, 1088*m*, 1069*m*, 1050*w*, 922*s*, 799*m*, 725*w*, 696*s*, 624*m*, 587*vs*, 551*s* cm^−1^; ^1^H NMR:δ = 7.66–7.64 (*m*, 1H, 5-H), 7.63–7.58 (*m*, 1H, 6-H), 7.54–7.47 (*m*, 3H, 2-H, 4-H, NH), 7.37 (*s*, 2H, NH_2_), 3.97 (*t*, *J* = 6.5 Hz, 2H, 14-H), 2.99 (*hept*, *J* = 6.9 Hz, 1H, 7-H), 2.77–2.69 (*m*, 2H, 9-H), 1.55 (*p*, *J* = 6.7 Hz, 2H, 13-H), 1.34 (*p*, *J* = 7.0 Hz, 2H, 10-H), 1.22 (*d*, *J* = 6.9 Hz, 10H, 8-H, 8′-H, 11-H, 12-H) ppm; ^13^C NMR:δ = 149.6 (C-3), 140.7 (C-1), 130.4 (C-4), 129.1 (C-5), 124.0 (C-2), 124.0 (C-6), 68.9 (C-14), 42.4 (C-9), 33.3 (C-7), 28.8 (C-13), 28.2 (C-10), 25.5 (C-12), 24.6 (C-11), 23.6 (C-8) ppm; MS: *m*/*z* = 401.4 (100%, [M + Na]^+^); anal. calcd. for C_15_H_26_N_2_S_2_O_5_ (378.50): C 47.60, H 6.92, N 7.40; found: C 47.29, H 7.18, N 7.35.

#### 4.2.91. N-(7-Hydroxyheptyl)-3-isopropylbenzene Sulfonamide (**45a**)

Applying GPA: from 3-isopropylbenzenesulfonyl chloride (500 mg, 2.29 mmol), and 7-amino-heptanol (450 mg, 3.43 mmol): **45a** (667 mg, 93%); oil; R_f_ = 0.23 (petrolether/EtOAc, 2:3); UV–Vis: 223 nm (3.99); IR: ν = 3504*w*, 3280*w*, 2960*w*, 2930*m*, 2859*w*, 1478*w*, 1462*w*, 1427*w*, 1385*w*, 1365*w*, 1324*m*, 1306*s*, 1217*vw*, 1157*s*, 1144*s*, 1088*m*, 1069*m*, 999*w*, 904*w*, 798*m*, 696*s*, 624*m*, 587*vs*, 564*m*, 527*m*, 462*w* cm^−1^; ^1^H NMR:δ = 7.66–7.64 (*m*, 1H, 2-H), 7.63–7.57 (*m*, 1H, 6-H), 7.52–7.46 (*m*, 3H, 4-H, 5-H, NH), 4.29 (*t*, *J* = 5.1 Hz, 1H, OH), 3.37–3.32 (*m*, 2H, 15-H), 2.98 (*hept*, *J* = 6.9 Hz, 1H, 7-H), 2.72 (*q*, *J* = 6.9 Hz, 2H, 9-H), 1.40–1.27 (*m*, 4H, 10-H, 14-H), 1.22 (*d*, *J* = 6.9 Hz, 6H, 8-H, 8′-H), 1.20–1.11 (*m*, 6H, 11-H, 12-H, 13-H) ppm; ^13^C NMR:δ = 149.5 (C-3), 140.8 (C-1), 130.3 (C-4), 129.1 (C-5), 124.0 (C-2), 124.0 (C-6), 60.7 (C-15), 42.5 (C-9), 33.3 (C-7), 32.4 (C-14), 28.9 (C-10), 28.4 (C-12), 26.0 (C-11), 25.3 (C-13), 23.6 (C-8) ppm; MS: *m*/*z* = 336.3 (100%, [M + Na]^+^); anal. calcd. for C_16_H_27_N_2_S_2_O_5_ (313.46): C 61.31, H 8.68, N 4.47; found: C 61.07, H 8.97, N 4.29.

#### 4.2.92. 7-[(3-Isopropylphenyl)sulfonamido]heptyl Sulfamate (**45b**)

Applying GPB: from **45a** (200 mg, 0.67 mmol): **45b** (227 mg, 90%); oil; R_f_ = 0.68 (CHCl_3_/EtOAc, 2:3); UV–Vis: 269 nm (2.93); IR: ν = 3276*m*, 2961*w*, 2933*w*, 2862*w*, 1561*w*, 1478*w*, 1464*w*, 1428*w*, 1363*s*, 1323*s*, 1306*s*, 1217*vw*, 1178*s*, 1157*vs*, 1145*s*, 1089*m*, 1069*m*, 1052*w*, 997*w*, 923*s*, 799*m*, 724*w*, 696*s*, 624*m*, 587*vs*, 551*s* cm^−1^; ^1^H NMR:δ = 7.66–7.64 (*m*, 1H, 5-H), 7.62–7.58 (*m*, 1H, 6-H), 7.53–7.47 (*m*, 3H, 2-H, 4-H, NH), 7.37 (*s*, 2H, NH_2_), 3.98 (*t*, *J* = 6.5 Hz, 2H, 15-H), 2.99 (*hept*, *J* = 6.9 Hz, 1H, 7-H), 2.76–2.69 (*m*, 2H, 9-H), 1.63–1.52 (*m*, 2H, 14-H), 1.39–1.28 (*m*, 2H, 10-H), 1.23 (*d*, *J* = 6.9 Hz, 6H, 8-H, 8′-H), 1.29–1.13 (*m*, 6H, 11-H, 12-H, 13-H) ppm; ^13^C NMR:δ = 149.6 (C-3), 140.7 (C-1), 130.3 (C-4), 129.1 (C-5), 124.0 (C-2), 124.0 (C-6), 68.9 (C-15), 42.5 (C-9), 33.3 (C-7), 28.8 (C-14), 28.2 (C-11), 28.0 (C-10), 25.8 (C-13), 24.9 (C-12), 23.6 (C-8) ppm; MS: *m*/*z* = 415.4 (100%, [M + Na]^+^); anal. calcd. for C_16_H_28_N_2_S_2_O_5_ (392.53): C 48.96, H 7.19, N 7.14; found: C 48.75, H 7.34, N 6.86.

#### 4.2.93. N-(8-Hydroxyoctyl)-3-isopropylbenzene Sulfonamide (**46a**)

Applying GPA: from 3-isopropylbenzenesulfonyl chloride (500 mg, 2.29 mmol) and 8-amino-octanol (498 mg, 3.43 mmol): **46a** (649 mg, 87%); oil; R_f_ = 0.30 (petrolether/EtOAc, 2:3; UV–Vis: 223 nm (3.88); IR: ν = 3504*vw*, 3281*w*, 2960*w*, 2929*m*, 2857*m*, 1478*w*, 1462*w*, 1428*w*, 1385*w*, 1365*w*, 1324*m*, 1306*m*, 1217*vw*, 1157*s*, 1144*s*, 1088*m*, 1069*m*, 1051*m*, 998*vw*, 904*w*, 798*m*, 722*w*, 696*s*, 624*m*, 587*vs*, 564*m*, 511*m*, 456*w* cm^−1^; ^1^H NMR (500 MHz, DMSO-*d*_6_): δ = 7.66–7.64 (*m*, 1H, 2-H), 7.62–7.58 (*m*, 1H, 6-H), 7.52–7.47 (*m*, 3H, 4-H, 5-H, NH), 4.29 (*td*, *J* = 5.2, 0.8 Hz, 1H, OH), 3.35 (*td*, *J* = 6.6, 5.1 Hz, 2H, 16-H), 2.99 (*hept*, *J* = 6.9 Hz, 1H, 7-H), 2.72 (*td*, *J* = 7.0, 5.8 Hz, 2H, 9-H), 1.41–1.28 (*m*, 4H, 10-H, 15-H), 1.22 (*d*, *J* = 6.9 Hz, 6H, 8-H, 8′-H), 1.20–1.11 (*m*, 8H, 11-H, 12-H, 13-H, 14-H) ppm; ^13^C NMR (126 MHz, DMSO-*d*_6_): δ = 149.5 (C-3), 140.8 (C-1), 130.3 (C-4), 129.1 (C-5), 124.0 (C-2), 124.0 (C-6), 60.7 (C-16), 42.5 (C-9), 33.3 (C-7), 32.5 (C-15), 28.9 (C-10), 28.8 (C-12), 28.6 (C-13), 25.9 (C-11), 25.4 (C-14), 23.6 (C-8) ppm; MS: *m*/*z* = 350.3 (100%, [M + Na]^+^); anal. calcd. for C_17_H_29_NSO_3_ (327.48): C 62.35, H 8.93, N 4.28; found: C 62.17, H 9.20, N 3.97.

#### 4.2.94. 8-[(3-Isopropylphenyl)sulfonamido]octyl Sulfamate (**46b**)

Applying GPB: from **46a** (500 mg, 2.29 mmol): **46b** (196 mg, 79%); oil; R_f_ = 0.73 (CHCl_3_/EtOAc, 2:3); UV–Vis: 268 nm (3.00); IR: ν = 3276*m*, 2961*w*, 2930*m*, 2859*w*, 1561*w*, 1478*w*, 1464*w*, 1428*w*, 1363*s*, 1324*m*, 1306*s*, 1217*vw*, 1178*s*, 1157*vs*, 1145*s*, 1089*m*, 1070*m*, 1051*w*, 998*w*, 922*s*, 799*m*, 723*w*, 696*s*, 624*m*, 587*vs*, 552*s*, 461*w* cm^−1^; ^1^H NMR:δ = 7.66–7.64 (*m*, 1H, 5-H), 7.61–7.58 (*m*, 1H, 6-H), 7.53–7.48 (*m*, 3H, 2-H, 4-H, NH), 7.37 (*s*, 2H, NH_2_), 3.99 (*t*, *J* = 6.5 Hz, 2H, 17-H), 2.99 (*hept*, *J* = 6.9 Hz, 1H, 7-H), 2.73 (*td*, *J* = 7.0, 5.8 Hz, 2H, 9-H), 1.63–1.55 (*m*, 2H, 16-H), 1.38–1.10 (*m*, 10H, 10-H, 11-H, 12-H, 13-H, 14-H), 1.23 (*d*, *J* = 6.9 Hz, 6H, 8-H, 8′-H) ppm; ^13^C NMR:δ = 149.6 (C-3), 140.7 (C-1), 130.3 (C-4), 129.1 (C-5), 124.0 (C-2), 124.0 (C-6), 69.0 (C-17), 42.5 (C-9), 33.3 (C-7), 28.8 (C-16), 28.4 (C-13), 28.3 (C-10), 28.3 (C-12), 25.9 (C-11), 24.9 (C-14), 23.6 (C-8) ppm; MS: *m*/*z* = 429.5 (100%, [M + Na]^+^); anal. calcd. for C_17_H_30_N_2_S_2_O_5_ (406.56): C 50.22, H 7.44, N 6.89; found: C 49.97, H 7.70, N 6.54.

#### 4.2.95. N-(7-Hydroxyheptyl)-2-isopropylbenzene Sulfonamide (**47a**)

Applying GPA: from 2-isopropylbenzenesulfonyl chloride (500 mg, 2.29 mmol) and 7-amino-heptanol (450 mg, 3.43 mmol): **47a** (634 mg, 88%); oil; R_f_ = 0.32 (petrolether/EtOAc, 2:3); UV–Vis: 224 nm (4.02); IR: ν = 3502*w*, 3292*w*, 2931*m*, 2859*m*, 1474*m*, 1461*w*, 1444*m*, 1385*w*, 1363*w*, 1316*s*, 1204*w*, 1148*vs*, 1115*m*, 1056*s*, 1031*m*, 878*w*, 763*s*, 725*w*, 686*m*, 595*vs*, 565*s*, 545*s*, 462*w*, 448*w* cm^−1^; ^1^H NMR:δ = 7.80–7.76 (*m*, 1H, 3-H), 7.66 (*t*, *J* = 5.7 Hz, 1H, NH), 7.60–7.53 (*m*, 2H, 6-H, 4-H), 7.33 (*ddd*, *J* = 8.0, 5.7, 2.9 Hz, 1H, 5-H), 4.29 (*t*, *J* = 5.1 Hz, 1H, OH), 3.85 (*hept*, *J* = 6.8 Hz, 1H, 7-H), 3.35 (*td*, *J* = 6.5, 5.2 Hz, 2H, 15-H), 2.79 (*td*, *J* = 7.0, 5.7 Hz, 2H, 9-H), 1.40–1.30 (*m*, 4H, 10-H, 14-H), 1.25–1.11 (*m*, 12H, 8-H, 8′-H, 11-H, 12-H, 13-H) ppm; ^13^C NMR:δ = 147.7 (C-2), 138.1 (C-1), 132.5 (C-4), 128.0 (C-6), 127.9 (C-4), 125.8 (C-5), 60.7 (C-15), 42.4 (C-9), 32.4 (C-14), 29.2 (C-10), 28.4 (C-12), 28.3 (C-7), 26.0 (C-11), 25.3 (C-13), 23.9 (C-8, C-8′) ppm; MS: *m*/*z* = 336.3 (100%, [M + Na]^+^); anal. calcd. for C_16_H_27_NSO_3_ (313.46): C 61.31, H 8.68, N 4.47; found: C 61.07, H 9.02, N 4.16.

#### 4.2.96. 7-[(2-Isopropylphenyl)sulfonamido]heptyl Sulfamate (**47b**)

Applying GPB: from **47a** (200 mg, 0.64 mmol): **47b** (131 mg, 52%); oil; R_f_ = 0.76 (CHCl_3_/EtOAc, 2:3); UV–Vis: 224 nm (4.04); IR: ν = 3283*m*, 2933*w*, 2862*w*, 1593*vw*, 1570*w*, 1474*w*, 1444*w*, 1361*s*, 1315*s*, 1202*w*, 1179*s*, 1162*s*, 1148*vs*, 1115*m*, 1083*m*, 1071*m*, 1056*m*, 1031*w*, 924*s*, 815*m*, 764*s*, 724*w*, 687*m*, 596*s*, 563*s*, 550*vs*, 444*w* cm^−1^; ^1^H NMR:δ = 7.80–7.75 (*m*, 1H, 3-H), 7.68 (*t*, *J* = 5.7 Hz, 1H, NH), 7.61–7.54 (*m*, 2H, 4-H, 6-H), 7.37 (*s*, 2H, NH_2_), 7.34 (*ddd*, J = 7.9, 5.9, 2.8 Hz, 1H, 5-H), 3.98 (*t*, *J* = 6.5 Hz, 2H, 15-H), 3.84 (*hept*, *J* = 6.9 Hz, 1H, 7-H), 2.79 (*td*, *J* = 7.0, 5.7 Hz, 2H, 9-H), 1.63–1.52 (*m*, 2H, 14-H), 1.42–1.31 (*m*, 2H, 10-H), 1.21 (*d*, *J* = 6.8 Hz, 6H, 8-H, 8′-H), 1.31–1.13 (*m*, 6H, 11-H, 12-H, 13-H) ppm; ^13^C NMR:δ = 147.7 (C-2), 138.1 (C-1), 132.6 (C-4), 128.0 (C-3), 127.9 (C-6), 125.8 (C-5), 68.9 (C-15), 42.3 (C-9), 29.1 (C-10), 28.3 (C-7), 28.2 (C-14), 28.0 (C-12), 25.8 (C-11), 24.9 (C-13), 23.9 (C-8) ppm; MS: *m*/*z* = 415.6 (100%, [M + Na]^+^); anal. calcd. for C_16_H_28_N_2_S_2_O_5_ (392.53): C 48.96, H 7.19, N 7.14; found: C 48.75, H 7.37, N 6.87.

#### 4.2.97. N-(8-Hydroxyoctyl)-2-isopropylbenzene Sulfonamide (**48a**)

Applying GPA: from 2-isopropylbenzenesulfonyl chloride (500 mg, 2.29 mmol) and 8-amino-octanol (498 mg, 3.43 mmol): **48a** (720 mg, 96%); oil; R_f_ = 0.34 (petrolether/EtOAc, 2:3); UV–Vis: 224 nm (3.90); IR: ν = 3498*w*, 3294*w*, 2929*m*, 2856*m*, 1474*m*, 1444*m*, 1385*w*, 1363*w*, 1316*s*, 1204*w*, 1148*vs*, 1115*m*, 1080*m*, 1071*m*, 1056*s*, 1031*m*, 890*w*, 763*s*, 723*w*, 686*m*, 595*vs*, 565*vs*, 545*s*, 450*w*, 419*vw* cm^−1^; ^1^H NMR:δ = 7.80–7.75 (*m*, 1H, 3-H), 7.66 (*s*, 1H, NH), 7.60–7.54 (*m*, 2H, 4-H, 6-H), 7.33 (*ddd*, *J* = 8.0, 5.8, 2.8 Hz, 1H, 5-H), 4.30 (*t*, *J* = 5.2 Hz, 1H, OH), 3.84 (*hept*, *J* = 6.8 Hz, 1H, 7-H), 3.38–3.33 (*m*, 2H, 16-H), 2.78 (*t*, *J* = 7.1 Hz, 2H, 9-H), 1.41–1.29 (*m*, 4H, 10-H, 15-H), 1.21 (*d*, *J* = 6.8 Hz, 6H, 8-H, 8′-H), 1.18–1.12 (*m*, 8H, 11-H, 12-H, 13-H, 14-H) ppm; ^13^C NMR:δ = 147.7 (C-2), 138.1 (C-1), 132.5 (C-4), 128.0 (C-3), 127.9 (C-6), 125.8 (C-5), 60.7 (C-16), 42.4 (C-9), 32.5 (C-15), 29.2 (C-13), 28.8 (C-10), 28.5 (C-12), 28.3 (C-7), 25.9 (C-11), 25.4 (C-14) 23.9 (C-8) ppm; MS: *m*/*z* = 350.3 (100%, [M + Na]^+^); anal. calcd. for C_17_H_29_NSO_3_ (327.48): C 62.35, H 8.93, N 4.28; found: C 62.6, H 9.24, N 3.96.

#### 4.2.98. 8-[(2-Isopropylphenyl)sulfonamido]octyl Sulfamate (**48b**)

Applying GPB: from **47a** (200 mg, 0.61 mmol): **47b** (202 mg, 82%); oil; R_f_ = 0.82 (CHCl_3_/EtOAc, 2:3); UV–Vis: 224 nm (3.86); IR: ν = 3285*w*, 2931*m*, 2858*w*, 1593*vw*, 1570*w*, 1474*w*, 1444*w*, 1362*s*, 1315*s*, 1202*w*, 1179*s*, 1161*s*, 1149*vs*, 1115*m*, 1056*m*, 1031*w*, 925*s*, 819*w*, 764*s*, 724*w*, 687*m*, 596*s*, 564*s*, 550*s*, 450*w*, 446*w* cm^−1^; ^1^H NMR:δ = 7.80–7.75 (*m*, 1H, 3-H), 7.67 (*t*, *J* = 5.7 Hz, 1H, NH), 7.60–7.54 (*m*, 2H, 4-H, 6-H), 7.37 (*s*, 2H, NH_2_), 7.34 (*ddd*, *J* = 7.9, 5.9, 2.8 Hz, 1H, 5-H), 3.99 (*t*, *J* = 6.5 Hz, 2H, 16-H), 3.84 (*hept*, *J* = 6.8 Hz, 1H, 7-H), 2.79 (*td*, *J* = 7.0, 5.7 Hz, 2H, 9-H), 1.63–1.55 (*m*, 2H, 15-H), 1.39–1.31 (*m*, 2H, 10-H), 1.30–1.24 (*m*, 2H, 14-H), 1.21 (*d*, *J* = 6.8 Hz, 6H, 8-H, 8′-H), 1.22–1.14 (*m*, 6H, 11-H, 12-H, 13-H) ppm; ^13^C NMR (126 MHz, DMSO-*d*_6_): δ = 147.7 (C-2), 138.1 (C-1), 132.5 (C-4), 128.0 (C-3), 127.9 (C-6), 125.8 (C-5), 69.0 (C-16), 42.3 (C-9), 29.2 (C-15), 28.4 (C-10), 28.3 (C-7, C-13), 28.3 (C-12), 25.9 (C-11), 24.9 (C-14), 23.9 (C-8) ppm; MS: *m*/*z* = 429.6 (100%, [M + Na]^+^); anal. calcd. for C_17_H_30_N_2_S_2_O_5_ (406.56): C 50.22, H 7.44, N 6.89; found: C 49.96, H 7.70, N 6.55.

#### 4.2.99. 4-(tert-Butyl)-N-(2-hydroxyethyl)benzene Sulfonamide (**49a**) [477483-08-2]

Applying GPA: from 4-(tert-butyl)benzenesulfonyl chloride (500 mg, 2.15 mmol) and 2-amino-ethanol (197 mg, 3.22 mmol): **49a** (435 mg, 79%); white solid; R_f_ = 0.20 (petrolether/EtOAc, 2:3); m.p. = 75–77 °C; UV–Vis: 228 nm (4.10); IR: ν = 3522*w*, 3162*w*, 3072*vw*, 2952*w*, 2903*w*, 2891*w*, 2868*w*, 1596*w*, 1463*w*, 1450*w*, 1432*w*, 1400*w*, 1364*w*, 1354*vw*, 1325*s*, 1308*m*, 1291*w*, 1261*w*, 1207*vw*, 1198*w*, 1161*vs*, 1115*m*, 1087*m*, 1071*m*, 1057*s*, 1016*w*, 955*m*, 905*w*, 844*w*, 831*vw*, 823*m*, 763*s*, 726*m*, 624*s*, 580*vs*, 550*s*, 515*vw*, 498*vw*, 473*w*, 457*w*, 421*w*, 411*w* cm^−1^; ^1^H NMR:δ = 7.75–7.70 (*m*, 2H, 2-H, 2′-H), 7.63–7.58 (*m*, 2H, 3-H, 3′-H), 7.50 (*s*, 1H, NH), 4.66 (*t*, *J* = 5.5 Hz, 1H, OH), 3.37 (*q*, *J* = 6.0 Hz, 2H, 8-H), 2.77 (*t*, *J* = 6.4 Hz, 2H, 7-H), 1.30 (*s*, 9H, 6-H, 6′-H, 6′-H) ppm; ^13^C NMR:δ = 155.2 (C-4), 137.7 (C-1), 126.4 (C-2), 125.9 (C-3), 59.9 (C-8), 45.1 (C-7), 34.8 (C-5), 30.8 (C-6) ppm; MS: *m*/*z* = 280.4 (100%, [M + Na]^+^); anal. calcd. for C_12_H_19_NSO_3_ (257.35): C 56.01, H 7.44, N 5.44; found: C 55.76, H 7.72, N 5.16. 

#### 4.2.100. 2-[(4-(tert-Butyl)phenyl)sulfonamido]ethyl Sulfamate (**49b**)

Applying GPB: from **49a** (200 mg, 0.78 mmol): **49b** (228 mg, 87%); white solid; R_f_ = 0.60 (CHCl_3_/EtOAc, 2:3); m.p. = 92–93 °C; UV–Vis: 228 nm (4.02); IR: ν = 3392*w*, 3292*m*, 3267*m*, 3075*vw*, 2951*w*, 2902*w*, 2865*w*, 1596*w*, 1542*w*, 1462*w*, 1445*m*, 1434*w*, 1401*w*, 1375*s*, 1325*s*, 1309*m*, 1292*w*, 1268*w*, 1228*vw*, 1197*w*, 1181*s*, 1159*s*, 1115*m*, 1088*s*, 1021*m*, 954*s*, 923*s*, 853*s*, 841*s*, 833*m*, 786*m*, 773*m*, 751*m*, 656*m*, 621*m*, 601*vs*, 578*m*, 549*vs*, 487*m*, 461*w*, 427*w*, 413*w* cm^−1^; ^1^H NMR:δ = 7.84 (*t*, *J* = 6.0 Hz, 1H, NH), 7.77–7.71 (*m*, 2H, 2-H, 2′-H), 7.64–7.60 (*m*, 2H, 3-H, 3′-H), 7.50 (*s*, 2H, NH_2_), 4.01 (*t*, *J* = 5.7 Hz, 2H, 8-H), 3.03 (*q*, *J* = 5.6 Hz, 2H, 7-H), 1.31 (*s*, 9H, 6-H, 6′-H, 6′-H) ppm; ^13^C NMR (126 MHz, DMSO-*d*_6_): δ = 155.9 (C-1), 137.8 (C-4), 126.8 (C-2), 126.5 (C-3), 68.0 (C-8), 42.0 (C-7), 35.3 (C-5), 31.3 (C-6) ppm; MS: *m*/*z* = 359.4 (100%, [M + Na]^+^); anal. calcd. for C_12_H_20_N_2_S_2_O_5_ (336.42): C 42.84, H 5.99, N 8.33; found: C 42.55, H 6.25, N 8.03. 

#### 4.2.101. 4-(tert-Butyl)-N-(3-hydroxypentyl)benzene Sulfonamide (**50a**) [1017436-75-7]

Applying GPA: from 4-(tert-butyl)benzenesulfonyl chloride (500 mg, 2.15 mmol) and 3-amino-propanol (242 mg, 3.22 mmol): **50a** (530 mg, 91%); white solid; R_f_ = 0.20 (petrolether/EtOAc, 2:3); m.p. = 63–65 °C; UV–Vis: 228 nm (4.15); IR: ν = 3311*m*, 3241*m*, 2963*m*, 2870*w*, 1597*w*, 1472*w*, 1426*m*, 1402*m*, 1363*w*, 1334*m*, 1313*s*, 1292*m*, 1270*w*, 1203*w*, 1160*vs*, 1112*m*, 1088*m*, 1069*m*, 1027*m*, 1008*m*, 960*m*, 908*w*, 847*w*, 834*m*, 826*m*, 756*s*, 704*m*, 625*s*, 578*vs*, 550*s*, 512*m*, 487*m*, 475*w*, 461*w* cm^−1^; ^1^H NMR:δ = 7.73–7.68 (*m*, 2H, 2-H, 2′-H), 7.63–7.58 (*m*, 2H, 3-H, 3′-H), 7.43 (*s*, 1H, NH), 4.40 (*s*, 1H, OH), 3.37 (*td*, *J* = 6.2, 2.8 Hz, 2H, 9-H), 2.77 (*t*, *J* = 7.3 Hz, 2H, 7-H), 1.58–1.49 (*m*, 2H, 8-H), 1.30 (*s*, 9H, 6-H, 6′-H, 6′-H) ppm; ^13^C NMR:δ = 155.2 (C-4), 137.6 (C-1), 126.4 (C-2), 126.0 (C-3), 58.1 (C-9), 40.0 (C-7), 34.8 (C-5), 32.4 (C-8), 30.8 (C-6) ppm; MS: *m*/*z* = 294.1 (100%, [M + Na]^+^); anal. calcd. for C_13_H_21_NSO_3_ (271.38): C 57.54, H 7.80, N 5.16; found: C 57.21, H 8.03, N 5.96.

#### 4.2.102. 3-[(4-(tert-Butyl)phenyl)sulfonamido]propyl Sulfamate (**50b**)

Applying GPB: from **50a** (200 mg, 0.74 mmol): **50b** (251 mg, 97%); white solid; R_f_ = 0.61 (CHCl_3_/EtOAc, 2:3); m.p. = 85–86 °C; UV–Vis: 228 nm (4.18); IR: ν = 3338*w*, 3293*w*, 3252*m*, 3121*vw*, 2964*w*, 2906*w*, 2870*vw*, 1597*w*, 1571*w*, 1476*w*, 1463*w*, 1437*w*, 1421*vw*, 1401*w*, 1373*s*, 1363*s*, 1329*m*, 1318*s*, 1294*m*, 1270*w*, 1242*vw*, 1169*s*, 1156*vs*, 1133*w*, 1111*m*, 1085*m*, 1060*w*, 1016*vw*, 982*s*, 949*s*, 891*m*, 877*w*, 839*m*, 827*s*, 757*s*, 678*m*, 631*m*, 595*m*, 589*m*, 573*s*, 564*s*, 548*vs*, 501*w*, 491*m*, 463*w*, 413*vw* cm^−1^; ^1^H NMR:δ = 7.74–7.69 (*m*, 2H, 2-H, 2′-H), 7.64–7.58 (*m*, 3H, 3-H, 3′-H, NH), 7.41 (*s*, 2H, NH_2_), 4.03 (*td*, *J* = 6.7, 5.0 Hz, 2H, 9-H), 2.81 (*td*, *J* = 7.1, 5.8 Hz, 2H, 7-H), 1.78 (*p*, *J* = 6.7 Hz, 2H, 8-H), 1.31 (*s*, 9H, 6-H, 6′-H, 6′-H) ppm; ^13^C NMR (126 MHz, DMSO-*d*_6_): δ = 155.4 (C-4), 137.4 (C-1), 126.3 (C-2), 126.1 (C-3), 66.5 (C-9), 39.2 (C-7), 34.8 (C-5), 30.8 (C-6), 28.8 (C-8) ppm; MS: *m*/*z* = 373.4 (100%, [M + Na]^+^); anal. calcd. for C_13_H_22_N_2_S_2_O_5_ (350.45): C 44.56, H 6.33, N 7.99; found: C 44.21, H 6.65, N 7.68.

#### 4.2.103. 4-(tert-Butyl)-N-(4-hydroxybutyl)benzene Sulfonamide (**51a**) [1082935-75-8]

Applying GPA: from 4-(tert-butyl)benzenesulfonyl chloride (500 mg, 2.15 mmol) and 4-amino-butanol (287 mg, 3.22 mmol): **51a** (518 mg, 84%) [118]; white solid; R_f_ = 0.20 (petrolether/EtOAc, 2:3); m.p. = 58–60 °C; UV–Vis: 228 nm (4.15); IR: ν = 3452*w*, 3248*w*, 3115*w*, 2962*w*, 2944*m*, 2867*w*, 1594*w*, 1471*w*, 1463*w*, 1435*w*, 1400*w*, 1364*w*, 1316*s*, 1291*m*, 1267*w*, 1197*w*, 1154*s*, 1111*m*, 1084*m*, 1065*m*, 1038*m*, 1018*w*, 988*w*, 909*w*, 839*m*, 753*m*, 735*w*, 685*w*, 627*s*, 581*vs*, 549*s*, 514*w*, 489*w*, 472*w*, 464*w* cm^−1^; ^1^H NMR:δ = 7.73–7.68 (*m*, 2H, 2-H, 2′-H), 7.62–7.58 (*m*, 2H, 3-H, 3′-H), 7.47 (*s*, 1H, NH), 4.35 (*s*, 1H, OH), 3.35–3.29 (*m*, 2H, 10-H), 2.75–2.69 (*m*, 2H, 7-H), 1.44–1.33 (*m*, 4H, 8-H, 9-H), 1.30 (*s*, 9H, 6-H, 6′-H, 6′-H) ppm; ^13^C NMR:δ = 155.1 (C-4), 137.8 (C-1), 126.3 (C-2), 125.9 (C-3), 60.2 (C-10), 42.5 (C-7), 34.8 (C-5), 30.8 (C-6), 29.5 (C-9), 25.8 (C-8) ppm; MS: *m*/*z* = 308.3 (100%, [M + Na]^+^); anal. calcd. for C_14_H_23_NSO_3_ (285.40): C 58.92, H 8.12, N 4.91; found: C 59.76, H 8.32, N 4.67.

#### 4.2.104. 4-[(4-(tert-Butyl)phenyl)sulfonamido]butyl Sulfamate (**51b**)

Applying GPB: from **51a** (200 mg, 0.7 mmol): **51b** (218 mg, 85%); white solid; R_f_ = 0.63 (CHCl_3_/EtOAc, 2:3); m.p. = 100–102 °C; UV–Vis: 228 nm (4.12); IR: ν = 3358*m*, 3288*m*, 3262*w*, 2965*w*, 1597*vw*, 1558*w*, 1483*vw*, 1476*w*, 1427*w*, 1399*w*, 1383*w*, 1368*s*, 1338*m*, 1321*s*, 1310*m*, 1292*w*, 1269*w*, 1205*w*, 1176*s*, 1158*vs*, 1122*w*, 1114*m*, 1108*m*, 1089*m*, 1065*m*, 1044*w*, 970*s*, 941*w*, 919*s*, 902*s*, 838*m*, 826*s*, 809*w*, 754*m*, 750*m*, 660*s*, 628*s*, 580*s*, 552*vs*, 533*m*, 514*w*, 507*m*, 413*w* cm^−1^; ^1^H NMR:δ = 7.74–7.68 (*m*, 2H, 2-H, 2′-H), 7.63–7.58 (*m*, 2H, 3-H, 3′-H), 7.54 (*t*, *J* = 5.9 Hz, 1H, NH), 7.38 (*s*, 2H, NH_2_), 3.96 (*t*, *J* = 6.3 Hz, 2H, 10-H), 2.79–2.71 (*m*, 2H, 7-H), 1.66–1.56 (*m*, 2H, 9-H), 1.50–1.40 (*m*, 2H, 8-H), 1.30 (*s*, 9H, 6-H, 6′-H, 6′-H) ppm; ^13^C NMR (126 MHz, DMSO-*d*_6_): δ = 155.2 (C-4), 137.7 (C-1), 126.3 (C-2), 126.0 (C-3), 68.5 (C-10), 42.0 (C-7), 34.8 (C-5), 30.8 (C-6), 25.6 (C-9), 25.4 (C-8) ppm; MS: *m*/*z* = 387.4 (100%, [M + Na]^+^); anal. calcd. for C_14_H_24_N_2_S_2_O_5_ (364.48): C 46.14, H 6.64, N 7.69; found: C 45.86, H 6.98, N 7.42.

#### 4.2.105. 4-(tert-Butyl)-N-(5-hydroxypentyl)benzene Sulfonamide (**52a**) [1925550-01-1]

Applying GPA: from 4-(tert-butyl)benzenesulfonyl chloride (500 mg, 2.15 mmol) and 5-amino-pentanol (332 mg, 3.22 mmol): **52a** (603 mg, 93%); white solid; R_f_ = 0.20 (petrolether/EtOAc, 2:3); m.p. = 53–54 °C; UV–Vis: 228 nm (4.02); IR: ν = 3286*m*, 2933*m*, 2863*w*, 1597*w*, 1473*w*, 1462*w*, 1424*m*, 1399*w*, 1362*w*, 1331*m*, 1318*s*, 1292*m*, 1268*w*, 1199*w*, 1158*s*, 1112*m*, 1088*m*, 1048*s*, 1016*w*, 887*m*, 843*m*, 826*m*, 755*m*, 735*w*, 689*m*, 638*m*, 630*s*, 573*vs*, 552*s*, 525*m*, 502*w* cm^−1^; ^1^H NMR (500 MHz, DMSO-*d*_6_): δ = 7.72–7.68 (*m*, 2H, 2-H, 2′-H), 7.62–7.58 (*m*, 2H, 3-H, 3′-H), 7.46 (*t*, *J* = 5.7 Hz, 1H, NH), 4.30 (*t*, *J* = 5.1 Hz, 1H, OH), 3.34–3.29 (*m*, 2H, 11-H), 2.73–2.68 (*m*, 2H, 7-H), 1.39–1.31 (*m*, 4H, 8-H, 10-H), 1.30 (*s*, 9H, 6-H, 6′-H, 6′-H), 1.26–1.15 (*m*, 2H, 9-H) ppm; ^13^C NMR:δ = 155.2 (C-4), 137.8 (C-1), 126.3 (C-2), 125.9 (C-3), 60.5 (C-11), 42.6 (C-7), 34.8 (C-5), 32.0 (C-10), 30.8 (C-6), 28.9 (C-8), 22.6 (C-9) ppm; MS: *m*/*z* = 322.4 (100%, [M + Na]^+^); anal. calcd. for C_15_H_25_NSO_3_ (299.43): C 60.17, H 8.42, N 4.68; found: C 59.87, H 8.70, N 4.39.

#### 4.2.106. 5-[(4-(tert-Butyl)phenyl)sulfonamido]pentyl Sulfamate (**52b**)

Applying GPB: from **52a** (500 mg, 2.15 mmol): **52b** (196 mg, 78%); white solid; R_f_ = 0.65 (CHCl_3_/EtOAc, 2:3); m.p. = 88–89 °C; UV–Vis: 228 nm (4.17); IR: ν = 3356*m*, 3278*m*, 3254*m*, 2968*w*, 2954*w*, 2860*w*, 1597*w*, 1557*w*, 1475*w*, 1463*w*, 1427*w*, 1398*w*, 1363*s*, 1326*s*, 1311*m*, 1292*w*, 1269*w*, 1175*s*, 1156*vs*, 1124*w*, 1111*m*, 1089*m*, 1068*w*, 1032*m*, 1000*m*, 965*s*, 918*s*, 900*s*, 852*w*, 840*m*, 821*s*, 775*m*, 754*m*, 738*w*, 627*s*, 579*vs*, 552*vs*, 533*s*, 520*m*, 501*m*, 443*w*, 412*w* cm^−1^; ^1^H NMR:δ = 7.73–7.68 (*m*, 2H, 2-H, 2′-H), 7.63–7.57 (*m*, 2H, 3-H, 3′-H), 7.49 (*t*, *J* = 5.9 Hz, 1H, NH), 7.37 (*s*, 2H, NH_2_), 3.96 (*t*, *J* = 6.4 Hz, 2H, 11-H), 2.72 (*q*, *J* = 6.6 Hz, 2H, 7-H), 1.55 (*p*, *J* = 6.7 Hz, 2H, 10-H), 1.44–1.35 (*m*, 2H, 8-H), 1.30 (*s*, 11H, 6-H, 6′-H, 6′-H, 9-H) ppm; ^13^C NMR:δ = 155.2 (C-4), 137.7 (C-1), 126.3 (C-2), 126.0 (C-3), 68.8 (C-11), 42.3 (C-7), 34.8 (C-5), 30.8 (C-6), 28.5 (C-8), 27.8 (C-10), 22.2 (C-9) ppm; MS: *m*/*z* = 401.6 (100%, [M + Na]^+^); anal. calcd. for C_15_H_26_N_2_S_2_O_5_ (378.50): C 47.60, H 6.92, N 7.40; found: C 47.32, H 7.24, N 7.09.

#### 4.2.107. 4-(tert-Butyl)-N-(6-hydroxyhexyl)benzene Sulfonamide (**53a**) [1787706-38-0]

Applying GPA: from 4-(tert-butyl)benzenesulfonyl chloride (500 mg, 2.15 mmol) and 6-amino-hexanol (378 mg, 3.22 mmol): **53a** (635 mg, 94%) [119,120]; white solid; R_f_ = 0.45 (petrolether/EtOAc 1: 1); m.p. = 53–54 °C; UV–Vis: 229 nm (4.21); IR: ν = 3504*br*, 3281*br*, 2935*m*, 2864*w*, 1596*w*, 1475*w*, 1462*w*, 1398*w*, 1365*w*, 1321*s*, 1269*w*, 1198*w*, 1157*vs*, 1112*s*, 1087*s*, 1056*m*, 1026*w*, 1014*w*, 835*m*, 754*m*, 726*w*, 734*w*, 626*s*, 581*s*, 549*s*, 455*w* cm^−1^; ^1^H NMR (500 MHz, DMSO-*d*_6_): δ = 7.72–7.68 (*m*, 2H, 2-H, 2′-H), 7.62–7.58 (*m*, 2H, 3-H, 3′-H), 7.49–7.44 (*m*, 1H, NH), 4.29 (*t*, *J* = 5.1 Hz, 1H, OH), 3.36–3.32 (*m*, 2H, 12-H), 2.71 (*td*, *J* = 7.2, 3.7 Hz, 2H, 7-H), 1.37–1.31 (*m*, 4H, 8-H, 11-H), 1.30 (*s*, 9H, 6-H, 6′-H, 6′-H), 1.22–1.13 (*m*, 4H, 9-H, 10-H) ppm; ^13^C NMR:δ = 155.2 (C-4), 137.8 (C-1), 126.3 (C-2), 125.9 (C-3), 60.6 (C-12), 42.5 (C-7), 34.8 (C-5), 32.3 (C-11), 30.8 (C-6), 29.0 (C-8), 25.9 (C-10), 25.0 (C-9) ppm; MS: *m*/*z* = 336.2 (100%, [M + Na]^+^); anal. calcd. for C_16_H_27_NSO_3_ (313.46): C 61.31, H 8.68, N 4.47; found: C 59.97, H 8.98, N 4.16.

#### 4.2.108. 6-[(4-(tert-Butyl)phenyl)sulfonamido]hexyl Sulfamate (**53b**)

Applying GPB: from **53a** (196 mg, 0.62 mmol): **53b** (152 mg, 62%); white solid; R_f_ = 0.69 (CHCl_3_/EtOAc, 2:3); m.p. = 89–90 °C; UV–Vis: 228 nm (4.12); IR: ν = 3364*w*, 3292*m*, 3253*m*, 2964*w*, 2952*w*, 2941*w*, 2863*w*, 1596*w*, 1571*w*, 1481*w*, 1427*w*, 1399*w*, 1364*s*, 1338*m*, 1323*s*, 1308*m*, 1293*w*, 1285*w*, 1270*w*, 1158*vs*, 1110*m*, 1088*m*, 1062*m*, 1023*m*, 1011*w*, 963*m*, 942*m*, 897*m*, 884*m*, 826*s*, 805*w*, 755*m*, 733*w*, 658*s*, 633*m*, 577*s*, 568*s*, 552*m*, 536*m*, 528*m*, 504*w* cm^−1^; ^1^H NMR:δ = 7.70–7.65 (*m*, 2H, 2-H, 2′-H), 7.60–7.55 (*m*, 2H, 3-H, 3′-H), 7.45 (*t*, *J* = 5.9 Hz, 1H, NH), 7.34 (*s*, 2H, NH_2_), 3.94 (*t*, *J* = 6.5 Hz, 2H, 12-H), 2.69 (*q*, *J* = 6.7 Hz, 2H, 7-H), 1.57–1.48 (*m*, 2H, 11-H), 1.37–1.29 (*m*, 2H, 8-H), 1.28 (*s*, 9H, 6-H, 6′-H, 6′-H), 1.25–1.17 (*m*, 4H, 9-H, 10-H) ppm; ^13^C NMR (126 MHz, DMSO-*d*_6_): δ = 155.2 (C-4), 137.8 (C-1), 126.3 (C-2), 125.9 (C-3), 68.9 (C-12), 42.4 (C-7), 34.8 (C-5), 30.8 (C-6), 28.8 (C-11), 28.1 (C-8), 25.5 (C-10), 24.6 (C-9) ppm; MS: *m*/*z* = 415.6 (100%, [M + Na]^+^); anal. calcd. for C_16_H_28_N_2_S_2_O_5_ (392.53): C 48.96, H 7.19, N 7.14; found: C 48.71, H 7.32, N 6.91. 

#### 4.2.109. 4-(tert-Butyl)-N-(7-hydroxyheptyl)benzene Sulfonamide (**54a**)

Applying GPA: from 4-(tert-butyl)benzenesulfonyl chloride (500 mg, 2.15 mmol) and 7-amino-heptanol (423 mg, 3.22 mmol): **54a** (688 mg, 98%); white solid; R_f_ = 0.27 (petrolether/EtOAc, 2:3); m.p. = 51–52 °C; UV–Vis: 228 nm (4.20); IR: ν = 3296*w*, 3248*m*, 2927*m*, 2856*m*, 1597*w*, 1475*w*, 1464*w*, 1436*w*, 1401*m*, 1364*w*, 1319*s*, 1292*m*, 1269*w*, 1202*w*, 1156*s*, 1113*m*, 1089*m*, 1059*s*, 1030*m*, 1015*w*, 1006*w*, 891*w*, 843*w*, 831*m*, 758*m*, 695*m*, 627*m*, 573*vs*, 551*s*, 524*w*, 509*w* cm^−1^; ^1^H NMR:δ = 7.73–7.68 (*m*, 2H, 2-H, 2′-H), 7.62–7.57 (*m*, 2H, 3-H, 3′-H), 7.49–7.43 (*m*, 1H, NH), 4.29 (*t*, *J* = 5.1 Hz, 1H, OH), 3.34 (*td*, *J* = 6.8, 5.3 Hz, 2H, 13-H), 2.71 (*q*, *J* = 6.4 Hz, 2H, 7-H), 1.40–1.31 (*m*, 4H, 8-H, 12-H), 1.30 (*s*, 9H, 6-H, 6′-H, 6′-H), 1.23–1.13 (*m*, 6H, 9-H, 10-H, 11-H) ppm; ^13^C NMR (126 MHz, DMSO-*d*_6_): δ = 155.1 (C-4), 137.8 (C-1), 126.3 (C-2), 125.9 (C-3), 60.6 (C-13), 42.5 (C-7), 34.8 (C-5), 32.4 (C-12), 30.8 (C-6), 28.9 (C-8), 28.4 (C-10), 26.0 (C-9), 25.3 (C-11) ppm; MS: *m*/*z* = 350.1 (100%, [M + Na]^+^); anal. calcd. for C_17_H_29_NSO_3_ (327.48): C 62.35, H 8.93, N 4.28; found: C 62.06, H 4.55, N 3.96.

#### 4.2.110. 7-[(4-(tert-Butyl)phenyl)sulfonamido]heptyl Sulfamate (**54b**)

Applying GPB: from **54a** (300 mg, 0.92 mmol): **54b** (269 mg, 72%); white solid; R_f_ = 0.75 (CHCl_3_/EtOAc, 2:3); m.p. = 95–96 °C; UV–Vis: 228 nm (4.10); IR: ν = 3337*w*, 3248*m*, 3125*vw*, 2953*w*, 2931*m*, 2856*w*, 1596*w*, 1572*w*, 1476*w*, 1463*w*, 1432*w*, 1397*w*, 1373*s*, 1366*s*, 1324*s*, 1315*s*, 1311*s*, 1291*m*, 1269*w*, 1202*vw*, 1169*s*, 1158*vs*, 1121*w*, 1111*m*, 1088*m*, 1074*w*, 1057*m*, 1047*w*, 1015*w*, 1000*m*, 972*m*, 949*s*, 895*m*, 854*vw*, 833*m*, 824*s*, 775*w*, 760*s*, 726*m*, 699*s*, 626*m*, 590*m*, 575*vs*, 564*s*, 550*s*, 535*m*, 529*m*, 517*m*, 502*w*, 477*w*, 411*vw* cm^−1^; ^1^H NMR:δ = 7.72–7.68 (*m*, 2H, 2-H, 2′-H), 7.62–7.58 (*m*, 2H, 3-H, 3′-H), 7.47 (*t*, *J* = 5.8 Hz, 1H, NH), 7.37 (*s*, 2H, NH_2_), 3.98 (*t*, *J* = 6.5 Hz, 2H, 13-H), 2.71 (*td*, *J* = 7.0, 5.9 Hz, 2H, 7-H), 1.63–1.54 (*m*, 2H, 12-H), 1.30 (*s*, 9H, 6-H, 6′-H, 6′-H), 1.39–1.16 (*m*, 8H, 8-H, 9-H, 10-H, 11-H) ppm; ^13^C NMR (126 MHz, DMSO-*d*_6_): δ = 155.2 (C-4), 137.8 (C-1), 126.3 (C-2), 125.9 (C-3), 68.9 (C-13), 42.5 (C-7), 34.8 (C-5), 30.8 (C-6), 28.9 (C-8), 28.2 (C-12), 28.0 (C-10), 25.8 (C-11), 24.9 (C-9) ppm; MS: *m*/*z* = 429.2 (100%, [M + Na]^+^); anal. calcd. for C_17_H_30_N_2_S_2_O_5_ (406.56): C 50.22, H 7.44, N 6.89; found: C 49.96, H 7.77, N 6.53.

#### 4.2.111. 4-(tert-Butyl)-N-(8-hydroxyoctyl)benzene Sulfonamide (**55a**)

Applying GPA: from 4-(tert-butyl)benzenesulfonyl chloride (500 mg, 2.15 mmol) and 8-amino-octanol (468 mg, 3.22 mmol): **55a** (653 mg, 89%); white solid; R_f_ = 0.30 (petrolether/EtOAc, 2:3); m.p. = 69–71 °C; UV–Vis: 228 nm (4.13); IR: ν = 3263*m*, 2927*m*, 2854*m*, 1597*w*, 1479*w*, 1467*w*, 1428*m*, 1401*w*, 1363*w*, 1325*s*, 1292*m*, 1270*w*, 1202*w*, 1156*vs*, 1122*w*, 1111*m*, 1088*m*, 1057*m*, 1046*m*, 1016*w*, 999*w*, 950*vw*, 896*w*, 881*w*, 841*w*, 827*m*, 757*s*, 736*w*, 723*w*, 675*m*, 634*m*, 575*vs*, 550*s*, 535*w*, 525*w*, 511*m* cm^−1^; ^1^H NMR:δ = 7.72–7.68 (*m*, 2H, 2-H, 2′-H), 7.62–7.58 (*m*, 2H, 3-H, 3′-H), 7.48–7.43 (*m*, 1H), 4.30 (*t*, *J* = 5.2 Hz, 1H), 3.35 (*td*, *J* = 6.6, 5.1 Hz, 2H, 14-H), 2.71 (*q*, *J* = 6.5 Hz, 2H, 7-H), 1.41–1.32 (*m*, 4H, 8-H, 13-H), 1.30 (*s*, 9H, 6-H, 6′-H, 6′-H), 1.26–1.12 (*m*, 8H, 9-H, 10-H, 11-H, 12-H) ppm; ^13^C NMR (126 MHz, DMSO-*d*_6_): δ = 155.1 (C-4), 137.8 (C-1), 126.3 (C-2), 125.9 (C-3), 60.7 (C-14), 42.5 (C-7), 34.8 (C-5), 32.5 (C-13), 30.8 (C-6), 28.9 (C-8), 28.7 (C-11), 28.6 (C-10), 25.9 (C-9), 25.4 (C-12) ppm; MS: *m*/*z* = 364.4 (100%, [M + Na]^+^); anal. calcd. for C_18_H_31_NSO_3_ (341.51): C 63.31, H 9.15, N 4.10; found: C 63.11, H 4.40, N 3.86.

#### 4.2.112. 8-[(4-(tert-Butyl)phenyl)sulfonamido]octyl Sulfamate (**55b**)

Applying GPB: from **55a** (200 mg, 0.59 mmol): **55b** (240 mg, 98%); white solid; R_f_ = 0.79 (CHCl_3_/EtOAc, 2:3); m.p. = 79–81 °C; UV–Vis: 228 nm (4.10); IR: ν = 3334*w*, 3256*m*, 2950*w*, 293*m*, 2856*w*, 1596*w*, 1476*w*, 1463*w*, 1432*w*, 1392*w*, 1370*s*, 1360*s*, 1328*s*, 1314*s*, 1312*s*, 1285*m*, 1285*w*, 1169*s*, 1154*vs*, 1120*w*, 1110*m*, 1090*m*, 1060*m*, 1041*w*, 1011*w*, 1005*m*, 970*m*, 949*s*, 891*m*, 830*m*, 825*s*, 778*w*, 762*s*, 732*m*, 698*s*, 626*m*, 591*m*, 576*vs*, 562*s*, 555*s*, 531*m*, 530*m*, 514*m* cm^−1^; ^1^H NMR:δ = 7.72–7.68 (*m*, 2H, 2-H, 2′-H), 7.62–7.58 (*m*, 2H, 3-H, 3′-H), 7.46 (*t*, *J* = 5.8 Hz, 1H, NH), 7.37 (*s*, 2H, NH_2_), 3.99 (*t*, *J* = 6.5 Hz, 2H, 14-H), 2.71 (*td*, *J* = 7.0, 5.8 Hz, 2H, 7-H), 1.59 (*dt*, *J* = 8.2, 6.5 Hz, 2H, 13-H), 1.39–1.24 (*m*, 4H, 8-H, 12-H), 1.30 (*s*, 9H, 6-H, 6′-H, 6′-H), 1.26–1.12 (*m*, 6H, 9-H, 10-H, 11-H) ppm; ^13^C NMR:δ = 155.2 (C-4), 137.8 (C-1), 126.3 (C-2), 125.9 (C-3), 68.9 (C-14), 42.5 (C-7), 34.8 (C-5), 30.8 (C-6), 28.9 (C-13), 28.4 (C-11), 28.3 (C-8), 28.3 (C-10), 25.9 (C-12), 24.9 (C-9) ppm; MS: *m*/*z* = 443.8 (100%, [M + Na]^+^); anal. calcd. for C_18_H_32_N_2_S_2_O_5_ (420.58): C 51.40, H 7.67, N 6.66; found: C 51.17, H 7.90, N 6.38.

#### 4.2.113. 4-(tert-Butyl)-N-(9-hydroxynonyl)benzene Sulfonamide (**56a**)

Applying GPA: from 4-(tert-butyl)benzenesulfonyl chloride (500 mg, 2.15 mmol) and 9-amino-nonanol (513 mg, 3.22 mmol): **56a** (636 mg, 83%); white solid; R_f_ = 0.78 (CHCl_3_/EtOAc, 2:3); m.p. = 69–70 °C; UV–Vis: 228 nm (4.07); IR: ν = 3459*vw*, 3285*m*, 2963*w*, 2925*m*, 2856*m*, 1598*w*, 1484*vw*, 1471*m*, 1438*w*, 1400*w*, 1363*w*, 1323*vs*, 1294*m*, 1270*w*, 1243*vw*, 1199*vw*, 1154*vs*, 1120*w*, 1110*m*, 1090*s*, 1072*s*, 1045*m*, 1037*m*, 1013*m*, 973*w*, 902*w*, 863*m*, 836*m*, 820*m*, 776*w*, 754*m*, 723*w*, 681*m*, 627*m*, 574*vs*, 551*s*, 534*m*, 521*m*, 473*vw*, 461*w*, 438*w* cm^−1^; ^1^H NMR:δ = 7.73–7.67 (*m*, 2H, 2-H, 2′-H), 7.62–7.57 (*m*, 2H, 3-H, 3′-H), 7.46 (*t*, *J* = 5.8 Hz, 1H, NH), 4.29 (*td*, *J* = 5.2, 1.0 Hz, 1H, OH), 3.36 (*td*, *J* = 6.6, 5.1 Hz, 2H, 15-H), 2.71 (*td*, *J* = 7.0, 5.8 Hz, 2H, 7-H), 1.43–1.35 (*m*, 2H, 14-H), 1.35–1.31 (*m*, 2H, 8-H), 1.30 (*s*, 9H, 6-H, 6′-H, 6′-H), 1.27–1.11 (*m*, 10H, 9-H, 10-H, 11-H, 12-H, 13-H) ppm; ^13^C NMR:δ = 155.1 (C-4), 137.8 (C-1), 126.3 (C-2), 125.9 (C-3), 60.7 (C-15), 42.5 (C-7), 34.8 (C-5), 32.5 (C-14), 30.8 (C-6), 28.9 (C-8), 28.9 (C-12), 28.8 (C-10), 28.5 (C-11), 26.0 (C-9), 25.4 (C-13) ppm; MS: *m*/*z* = 378.1 (100%, [M + Na]^+^); anal. calcd. for C_19_H_33_NSO_3_ (355.54): C 64.19, H 9.36, N 3.94; found: C 63.76, H 9.51, N 3.68.

#### 4.2.114. 9-[(4-(tert-Butyl)phenyl)sulfonamido]nonyl Sulfamate (**56b**)

Applying GPB: from **56a** (200 mg, 0.56 mmol): **56b** (187 mg, 76%); white solid; R_f_ = 0.88 (CHCl_3_/EtOAc, 2:3); m.p. = 106–107 °C; UV–Vis: 228 nm (4.06); IR: ν = 3358*w*, 3270*m*, 3124*vw*, 2963*w*, 2918*w*, 2853*w*, 1597*w*, 1571*w*, 1475*w*, 1466*w*, 1432*w*, 1397*w*, 1376*s*, 1314*s*, 1295*m*, 1272*w*, 1201*vw*, 1181*m*, 1157*vs*, 1111*m*, 1090*m*, 1062*w*, 1033*m*, 1015*vw*, 991*w*, 968*m*, 933*m*, 904*w*, 882*w*, 837*w*, 818*s*, 778*vw*, 758*m*, 733*vw*, 721*w*, 692*m*, 628*w*, 590*m*, 576*s*, 566*s*, 551*m*, 537*m*, 529*w*, 513*w*, 484*m*, 456*vw*, 411*vw* cm^−1^; ^1^H NMR:δ = 7.72–7.68 (*m*, 2H, 2-H, 2′-H), 7.62–7.58 (*m*, 2H, 3-H, 3′-H), 7.46 (*t*, *J* = 5.8 Hz, 1H, NH), 7.37 (*s*, 2H, NH_2_), 3.99 (*t*, *J* = 6.5 Hz, 2H, 15-H), 2.71 (*td*, *J* = 7.0, 5.8 Hz, 2H, 7-H), 1.65–1.55 (*m*, 2H, 14-H), 1.39–1.25 (*m*, 4H, 8-H, 13-H), 1.30 (*s*, 9H, 6-H, 6′-H, 6′-H), 1.26–1.12 (*m*, 8H, 9-H, 10-H, 11-H, 12-H) ppm; ^13^C NMR:δ = 155.16 (C-4), 137.82 (C-1), 126.31 (C-2), 125.92 (C-3), 68.96 (C-15), 42.48 (C-7), 34.78 (C-5), 30.80 (C-6), 28.92 (C-14), 28.71 (C-8), 28.43 (C-10), 28.39 (C-12), 28.29 (C-11), 25.94 (C-9), 25.01 (C-13) ppm; MS: *m*/*z* = 457.1 (100%, [M + Na]^+^); anal. calcd. for C_19_H_34_N_2_S_2_O_5_ (434.61): C 52.51, H 7.89, N 6.45; found: C 52.31, H 8.02, N 6.15.

#### 4.2.115. 4-(tert-Butyl)-N-(10-hydroxydecyl)benzene Sulfonamide (**57a**)

Applying GPA: from 4-(tert-butyl)benzenesulfonyl chloride (300 mg, 1.29 mmol) and 10-amino-decanol (335 mg, 1.93 mmol): **57a** (415 mg, 87%); white solid; R_f_ = 0.77 (CHCl_3_/EtOAc, 2:3); m.p. = 75–76 °C; UV–Vis: 228 nm (4.13); IR: ν = 3497*w*, 3131*w*, 2961*w*, 2915*s*, 2876*w*, 2849*m*, 1596*w*, 1476*w*, 1465*m*, 1447*w*, 1441*w*, 1398*m*, 1363*w*, 1317*s*, 1289*m*, 1267*w*, 1198*w*, 1154*vs*, 1110*s*, 1091*m*, 1074*s*, 1043*w*, 1031*w*, 1020*m*, 915*m*, 843*m*, 826*m*, 756*m*, 721*w*, 635*s*, 580*vs*, 554*m*, 521*m*, 505*w*, 468*m* cm^−1^; ^1^H NMR:δ = 7.72–7.68 (*m*, 2H, 2-H, 2′-H), 7.62–7.57 (*m*, 2H, 3-H, 3′-H), 7.46 (*t*, *J* = 5.7 Hz, 1H, NH), 4.30 (*td*, *J* = 5.2, 1.0 Hz, 1H, OH), 3.36 (*td*, *J* = 6.5, 5.1 Hz, 2H, 16-H), 2.74–2.67 (*m*, 2H, 7-H), 1.43–1.35 (*m*, 2H, 15-H), 1.36–1.26 (*m*, 2H, 8-H), 1.30 (*s*, 9H, 6-H, 6′-H, 6′-H), 1.27–1.11 (*m*, 12H, 9-H, 10-H, 11-H, 12-H, 13-H, 14-H) ppm; ^13^C NMR:δ = 155.1 (C-4), 137.9 (C-1), 126.3 (C-2), 125.9 (C-3), 60.7 (C-16), 42.5 (C-7), 34.8 (C-5), 32.5 (C-15), 30.8 (C-6), 29.0 (C-8), 28.9 (C-10, C-13), 28.8 (C-11), 28.5 (C-12), 26.0 (C-9), 25.5 (C-14) ppm; MS: *m*/*z* = 457.1 (100%, [M + Na]^+^); anal. calcd. for C_20_H_35_NSO_3_ (369.56): C 65.00, H 9.55, N 3.79; found: C 64.76, H 9.76, N 3.46. 

#### 4.2.116. 10-[(4-(tert-Butyl)phenyl)sulfonamido]decyl Sulfamate (**57b**)

Applying GPB: from **57a** (180 mg, 0.48 mmol): **57b** (140 mg, 64%); white solid; R_f_ = 0.87 (CHCl_3_/EtOAc, 2:3); m.p. = 92–93 °C; UV–Vis: 228 nm (4.11); IR: ν = 3354*w*, 3294*w*, 3264*m*, 2920*m*, 2852*m*, 1475*w*, 1466*w*, 1433*w*, 1393*w*, 1366*s*, 1319*s*, 1292*w*, 1158*vs*, 1113*m*, 1089*m*, 1055*m*, 1025*w*, 937*m*, 895*w*, 835*m*, 824*m*, 756*m*, 721*w*, 670*m*, 628*m*, 578*s*, 552*s*, 532*m* cm ^−1^; ^1^H NMR:δ = 7.72–7.67 (*m*, 2H, 2-H, 2′-H), 7.62–7.57 (*m*, 2H, 3-H, 3′-H), 7.45 (*t*, *J* = 5.8 Hz, 1H, NH), 7.37 (*s*, 2H, NH_2_), 4.00 (*t*, *J* = 6.5 Hz, 2H, 16-H), 2.74–2.67 (*m*, 2H, 7-H), 1.61 (*p*, *J* = 6.6 Hz, 2H, 15-H), 1.37–1.26 (*m*, 2H, 8-H), 1.30 (*s*, 9H, 6-H, 6′-H, 6′-H), 1.27–1.11 (*m*, 12H, 9-H, 10-H, 11-H, 12-H, 13-H, 14-H) ppm; ^13^C NMR:δ = 155.2 (C-4), 137.8 (C-1), 126.3 (C-2, 2′), 125.9 (C-3, 3′), 69.0 (C-16), 42.5 (C-7), 34.8 (C-5), 30.8 (C-6, 6′, 6′), 28.9 (C-8), 28.8 (C-11), 28.8 (C-15), 28.5 (C-10), 28.5 (C-13), 28.3 (C-12), 26.0 (C-14), 25.0 (C-9) ppm; MS: *m*/*z* = 457.1 (100%, [M + Na]^+^); anal. calcd. for C_20_H_36_N_2_S_2_O_5_ (448.64): C 53.54, H 8.09, N 6.24; found: C 53.34, H 8.37, N 5.99.

#### 4.2.117. 3-(tert-Butyl)-N-(2-hydroxyethyl)benzene Sulfonamide (**58a**)

Applying GPA: from 3-(tert-butyl)benzenesulfonyl chloride (500 mg, 2.15 mmol) and 2-amino-ethanol (197 mg, 3.22 mmol): **58a** (522 mg, 94%); oil; R_f_ = 0.20 (petrolether/EtOAc, 2:3); UV–Vis: 224 nm (4.04); IR: ν = 3500*w*, 3276*w*, 2962*w*, 2872*w*, 1481*m*, 1460*w*, 1416*m*, 1398*w*, 1366*w*, 1325*m*, 1307*s*, 1267*w*, 1205*vw*, 1156*vs*, 1125*m*, 1096*m*, 1057*m*, 998*vw*, 949*m*, 896*w*, 865*w*, 796*m*, 775*w*, 696*s*, 678*m*, 625*m*, 586*vs*, 545*m*, 534*m*, 524*m*, 481*w*, 456*w* cm^−1^; ^1^H NMR:δ = 7.82–7.80 (*m*, 1H, 2-H), 7.67 (*ddd*, *J* = 7.8, 2.0, 1.1 Hz, 1H, 6-H), 7.62 (*ddd*, *J* = 7.8, 1.8, 1.1 Hz, 1H, 4-H), 7.58 (*s*, 1, NH), 7.51 (*td*, *J* = 7.8, 0.5 Hz, 1H, 5-H), 4.67 (*t*, *J* = 5.5 Hz, 1H, OH), 3.37 (*q*, *J* = 6.1 Hz, 2H, 10-H), 2.79 (*t*, *J* = 6.4 Hz, 2H, 9-H), 1.31 (*s*, 9H, 8-H, 8′-H, 8′-H) ppm; ^13^C NMR:δ = 151.9 (C-3), 140.4 (C-1), 129.3 (C-4), 128.9 (C-5), 123.7 (C-2), 122.9 (C-6), 59.9 (C-10), 45.1 (C-9), 34.7 (C-7), 30.9 (C-8) ppm; MS: *m*/*z* = 280.3 (100%, [M + Na]^+^); anal. calcd. for C_12_H_19_NSO_3_ (257.35): C 56.01, H 7.44, N 5.44; found: C 55.76, H 7.69, N 55.16.

#### 4.2.118. 2-[(3-(tert-Butyl)phenyl)sulfonamido]ethyl Sulfamate (**58b**)

Applying GPB: from **58a** (200 mg, 0.78 mmol): **58b** (194 mg, 74%); oil; R_f_ = 0.68 (CHCl_3_/EtOAc, 2:3); UV–Vis: 224 nm (4.02); IR: ν = 3276*w*, 2963*w*, 2871*w*, 1560*w*, 1482*w*, 1460*w*, 1417*w*, 1365*s*, 1326*m*, 1309*m*, 1288*w*, 1267*w*, 1179*s*, 1156*vs*, 1125*m*, 1097*m*, 1021*m*, 998*w*, 925*s*, 867*w*, 797*w*, 775*m*, 753*m*, 695*m*, 678*m*, 626*m*, 585*s*, 549*s*, 493*w*, 434*w* cm^−1^; ^1^H NMR:δ = 7.94–7.87 (*m*, 1H, NH), 7.81–7.78 (*m*, 1H, 2-H), 7.73–7.67 (*m*, 1H, 6-H), 7.65–7.61 (*m*, 1H, 4-H), 7.56–7.47 (*m*, 3H, 5-H, NH_2_), 4.00 (*t*, *J* = 5.7 Hz, 2H, 10-H), 3.08–2.99 (*m*, 2H, 9-H), 1.32 (s, 9H, 8-H, 8′-H, 8′-H) ppm; ^13^C NMR:δ = 152.1 (3), 140.0 (1), 129.6 (4), 129.0 (5), 123.7 (2), 122.8 (6), 67.5 (10), 41.5 (9), 34.7 (7), 30.9 (8, 8′, 8′) ppm; MS: *m*/*z* = 359.6 (100%, [M + Na]^+^); anal. calcd. for C_12_H_20_N_2_S_2_O_5_ (336.42): C 42.84, H 5.99, N 8.33; found: C 42.63, H 6.24, N 8.01.

#### 4.2.119. 3-(tert-Butyl)-N-(3-hydroxypropyl)benzene Sulfonamide (**59a**)

Applying GPA: from 3-(tert-butyl)benzenesulfonyl chloride (500 mg, 2.15 mmol) and 3-amino-propanol (242 mg, 3.22 mmol): **59a** (572 mg, 98%); oil; R_f_ = 0.23 (petrolether/EtOAc, 2:3); UV–Vis: 224 nm (4.15); IR: ν = 3501*w*, 3276*w*, 2961*m*, 2873*w*, 1481*m*, 1460*w*, 1416*w*, 1398*w*, 1366*w*, 1324*m*, 1307*s*, 1267*w*, 1178*w*, 1155*vs*, 1125*m*, 1084*m*, 1069*m*, 1008*w*, 998*w*, 960*w*, 876*w*, 798*m*, 774*m*, 696*s*, 678*m*, 625*m*, 586*vs*, 534*m*, 491*m*, 460*w* cm^−1^; ^1^H NMR:δ = 7.79 (*td*, *J* = 1.9, 0.5 Hz, 1H, 2-H), 7.67 (*ddd*, *J* = 7.8, 2.0, 1.1 Hz, 1H, 6-H), 7.61 (*ddd*, *J* = 7.7, 1.8, 1.2 Hz, 1H, 4-H), 7.52 (*td*, *J* = 7.5, 1.8 Hz, 2H, 5-H, NH), 4.39 (*t*, *J* = 5.1 Hz, 1H, OH), 3.36 (*td*, *J* = 6.2, 4.5 Hz, 2H, 11-H), 2.79 (*td*, *J* = 7.5, 3.6 Hz, 2H, 9-H), 1.55–1.46 (*m*, 2H, 10-H), 1.31 (*s*, 9H, 8-H, 8′-H, 8′-H) ppm; ^13^C NMR:δ = 151.9 (C-3), 140.3 (C-1), 129.3 (C-4), 128.9 (C-5), 123.7 (C-2), 122.9 (C-6), 58.0 (C-11), 40.0 (C-9), 34.7 (C-7), 32.3 (C-10), 30.9 (C-8) ppm; MS: *m*/*z* = 294.1 (100%, [M + Na]^+^); anal. calcd. for C_13_H_21_NSO_3_ (271.38): C 57.54, H 7.80, N 5.16; found: C 57.16, H 8.03, N 4.91.

#### 4.2.120. 3-[(3-(tert-Butyl)phenyl)sulfonamido]propyl Sulfamate (**59b**)

Applying GPB: from **59a** (500 mg, 2.15 mmol): **59b** (212 mg, 82%); oil; R_f_ = 0.68 (CHCl_3_/EtOAc, 2:3); UV–Vis: 224 nm (3.85); IR: ν = 3274*w*, 2964*w*, 1482*w*, 1417*w*, 1364*s*, 1325*m*, 1308*m*, 1177*s*, 1156*vs*, 1125*m*, 1096*m*, 939*m*, 798*m*, 772*m*, 696*m*, 678*m*, 625*m*, 585*s*, 550*s*, 536*m*, 496*m* cm^−1^; ^1^H NMR:δ = 7.79 (*t*, *J* = 1.9 Hz, 1H, 2-H), 7.72–7.59 (*m*, 3H, 4-H, 6-H, NH), 7.53 (*t*, *J* = 7.8 Hz, 1H, 5-H), 7.41 (*s*, 2H, NH_2_), 4.02 (*t*, *J* = 6.3 Hz, 2H, 11-H), 2.81 (*t*, *J* = 7.2 Hz, 2H, 9-H), 1.75 (*p*, *J* = 6.7 Hz, 2H, 10-H), 1.31 (*s*, 9H, 8-H, 8′-H, 8′-H) ppm; ^13^C NMR:δ = 152.1 (C-3), 140.1 (C-1), 129.5 (C-4), 129.0 (C-5), 123.7 (C-2), 122.8 (C-6), 66.5 (C-11), 39.2 (C-9), 34.7 (C-7), 30.9 (C-8), 28.7 (C-10) ppm; MS: *m*/*z* = 373.5 (100%, [M + Na]^+^); anal. calcd. for C_13_H_22_N_2_S_2_O_5_ (350.45): C 44.56, H 6.33, N 7.99; found: C 44.16, H 6.68, N 7.51.

#### 4.2.121. 3-(tert-Butyl)-N-(4-hydroxybutyl)benzene Sulfonamide (**60a**)

Applying GPA: from 3-(tert-butyl)benzenesulfonyl chloride (500 mg, 2.15 mmol) and 4-amino-butanol (287 mg, 3.22 mmol): **60a** (589 mg, 96%); oil; R_f_ = 0.20 (petrolether/EtOAc, 2:3); UV–Vis: 224 nm (3.90); IR: ν = 3499*w*, 3280*w*, 2959*m*, 2870*w*, 1481*m*, 1460*w*, 1416*w*, 1398*w*, 1366*w*, 1324*m*, 1307*m*, 1267*w*, 1156*vs*, 1125*m*, 1088*m*, 1056*m*, 1034*w*, 998*w*, 872*w*, 797*m*, 773*w*, 696*s*, 678*m*, 626*m*, 586*vs*, 535*m*, 520*m*, 496*w*, 464*w* cm^−1^; ^1^H NMR:δ = 7.81–7.78 (*m*, 1H, 2-H), 7.66 (*ddd*, *J* = 7.8, 2.0, 1.1 Hz, 1H, 6-H), 7.61 (*ddd*, *J* = 7.8, 1.8, 1.1 Hz, 1H, 4-H), 7.56–7.48 (*m*, 2H, 5-H, NH), 4.35 (*s*, 1H, OH), 3.37–3.27 (*m*, 2H, 12-H), 2.77–2.70 (*m*, 2H, 9-H), 1.43–1.32 (*m*, 4H, 10-H, 11-H), 1.31 (*s*, 9H, 8-H, 8′-H, 8′-H) ppm; ^13^C NMR:δ = 151.9 (C-3), 140.5 (C-1), 129.2 (C-4), 128.9 (C-5), 123.7 (C-2), 122.9 (C-6), 60.2 (C-12), 42.5 (C-9), 34.7 (C-7), 30.9 (C-8), 29.5 (C-11), 25.7 (C-10) ppm; MS: *m*/*z* = 308.4 (100%, [M + Na]^+^); anal. calcd. for C_14_H_23_NSO_3_ (285.40): C 58.92, H 8.12, N 4.91; found: C 58.68, H 8.33, N 4.78.

#### 4.2.122. 4-[(3-(tert-Butyl)phenyl)sulfonamido]butyl Sulfamate (**60b**)

Applying GPB: from **60a** (183 mg, 0.64 mmol): **60b** (206 mg, 88%); oil; R_f_ = 0.69 (CHCl_3_/EtOAc, 2:3); UV–Vis: 225 nm (3.85); IR: ν = 3276*w*, 2962*w*, 2872*w*, 1481*w*, 1460*w*, 1438*w*, 1415*w*, 1366*m*, 1329*m*, 1308*m*, 1287*w*, 1266*w*, 1180*m*, 1157*vs*, 1125*m*, 1098*m*, 1089*m*, 1068*m*, 1010*w*, 996*w*, 923*m*, 865*w*, 799*m*, 778*m*, 741*w*, 698*s*, 677*s*, 631*m*, 588*vs*, 552*s*, 535*m*, 458*vw* cm^−1^; ^1^H NMR:δ = 7.78 (*t*, *J* = 1.9 Hz, 1H, 2-H), 7.74–7.58 (*m*, 3H, 4-H, 6-H, NH), 7.52 (*t*, *J* = 7.7 Hz, 1H, 5-H), 7.38 (*s*, 2H, NH_2_), 3.95 (*t*, *J* = 6.3 Hz, 2H, 12-H), 2.76 (*q*, *J* = 6.6 Hz, 2H, 9-H), 1.66–1.56 (*m*, 2H, 11-H), 1.48–1.39 (*m*, 2H, 10-H), 1.32 (*s*, 9H, 8-H, 8′-H, 8′-H) ppm; ^13^C NMR:δ = 152.0 (C-3), 140.4 (C-1), 129.3 (C-4), 129.0 (C-5), 123.7 (C-6), 122.8 (C-2), 34.7 (C-7), 30.9 (C-8), 25.6 (C-11), 25.4 (C-10) ppm; MS: *m*/*z* = 387.1 (100%, [M + Na]^+^); anal. calcd. for C_14_H_24_N_2_S_2_O_5_ (364.48): C 46.14, H 6.64, N 7.69; found: C 45.85, H 6.99, N 7.41. 

#### 4.2.123. 3-(tert-Butyl)-N-(5-hydroxypentyl)benzene Sulfonamide (**61a**)

Applying GPA: from 3-(tert-butyl)benzenesulfonyl chloride (500 mg, 2.15 mmol) and 5-amino-pentanol (332 mg, 3.22 mmol): **61a** (610 mg, 95%); oil; R_f_ = 0.23 (petrolether/EtOAc, 2:3); UV–Vis: 224 nm (3.90); IR: ν = 3503*w*, 3278*w*, 2939*m*, 2868*w*, 1481*m*, 1459*w*, 1416*w*, 1398*w*, 1366*w*, 1325*m*, 1307*m*, 1267*w*, 1156*vs*, 1125*m*, 1087*m*, 1040*m*, 998*w*, 898*w*, 797*w*, 774*w*, 697*s*, 678*m*, 626*m*, 586*vs*, 534*m*, 501*w*, 478*w*, 461*w* cm^−1^; ^1^H NMR:δ = 7.79 (*td*, *J* = 1.9, 0.5 Hz, 1H, 2-H), 7.66 (*ddd*, *J* = 7.8, 2.0, 1.1 Hz, 1H, 6-H), 7.61 (*ddd*, *J* = 7.7, 1.8, 1.2 Hz, 1H, 4-H), 7.55–7.48 (*m*, 2H, 5-H, NH), 4.30 (*t*, *J* = 5.1 Hz, 1H, OH), 3.35–3.27 (*m*, 2H, 13-H), 2.72 (*q*, *J* = 6.2 Hz, 2H, 9-H), 1.38–1.31 (*m*, 4H, 10-H, 12-H), 1.31 (*s*, 9H, 8-H, 8′-H, 8′-H), 1.26–1.17 (*m*, 2H, 11-H) ppm; ^13^C NMR (126 MHz, DMSO-*d*_6_): δ = 151.9 (C-3), 140.5 (C-1), 129.2 (C-4), 128.9 (C-5), 123.7 (C-2), 122.8 (C-6), 60.5 (C-13), 42.6 (C-9), 34.7 (C-7), 32.0 (C-12), 30.9 (C-8), 28.8 (C-10), 22.6 (C-11) ppm; MS: *m*/*z* = 322.3 (100%, [M + Na]^+^); anal. calcd. for C_15_H_25_NSO_3_ (299.43): C 60.17, H 8.42, N 4.68; found: C 59.86, H 8.85, N 4.32.

#### 4.2.124. 5-[(3-(tert-Butyl)phenyl)sulfonamido]pentyl Sulfamate (**61b**)

Applying GPB: from **61a** (200 mg, 0.67 mmol): **61b** (150 mg, 59%); oil; R_f_ = 0.69 (CHCl_3_/EtOAc, 2:3); UV–Vis: 224 nm (4.15); IR: ν = 3420*w*, 3368*w*, 3287*m*, 2920*m*, 2859*w*, 2869*w*, 1478*w*, 1410*m*, 1388*w*, 1332*m*, 1315*w*, 1299*w*, 1152*vs*, 1078*m*, 1062*m*, 1013*m*, 963*w*, 900*m*, 817*s*, 735*w*, 701*w*, 668*s*, 570*s*, 552*s*, 516*m*, 464*w*, 415*w* cm^−1^; ^1^H NMR:δ = 7.91–7.83 (*m*, 2H, 2-H, NH), 7.66 (*dd*, *J* = 8.1, 1.4 Hz, 1H, 6-H), 7.52 (*ddd*, *J* = 8.1, 7.2, 1.5 Hz, 1H, 4-H), 7.43–7.40 (*m*, 1H, 5-H), 7.39–7.37 (*m*, 2H, NH_2_), 3.99 (*t*, *J* = 6.5 Hz, 2H, 13-H), 2.86–2.78 (*m*, 2H, 9-H), 1.65–1.55 (*m*, 2H, 12-H), 1.51 (*s*, 9H, 8-H, 8′-H, 8′-H), 1.49–1.45 (*m*, 2H, 10-H), 1.41–1.30 (*m*, 2H, 11-H) ppm; ^13^C NMR:δ = 148.9 (C-3), 140.6 (C-1), 131.8 (C-4), 129.4 (C-5), 129.0 (C-2), 126.4 (C-6), 68.9 (C-13), 42.6 (C-9), 36.7 (C-7), 31.8 (C-8), 28.7 (C-12), 27.9 (C-10), 22.3 (C-11) ppm; MS: *m*/*z* = 401.6 (100%, [M + Na]^+^); anal. calcd. for C_15_H_26_N_2_S_2_O_5_ (378.50): C 47.60, H 6.92, N 7.40; found: C 47.38, H 7.17, N 7.36. 

#### 4.2.125. 3-(tert-Butyl)-N-(6-hydroxyhexyl)benzene Sulfonamide (**62a**)

Applying GPA: from 3-(tert-butyl)benzenesulfonyl chloride (500 mg, 2.15 mmol) and 6-amino-hexanol (378 mg, 3.22 mmol): **62a** (621 mg, 92%); oil; R_f_ = 0.25 (petrolether/EtOAc, 2:3); UV–Vis: 225 nm (3.94); IR: ν = 3505*vw*, 3278*w*, 2935*m*, 2864*w*, 1597*vw*, 1481*w*, 1460*w*, 1416*w*, 1397*w*, 1366*w*, 1325*m*, 1307*m*, 1287*w*, 1267*w*, 1156*vs*, 1125*m*, 1097*m*, 1086*m*, 1073*m*, 1055*m*, 899*w*, 869*w*, 797*m*, 773*w*, 697*s*, 678*m*, 626*m*, 587*vs*, 534*m*, 500*w*, 469*w*, 457*w*, 445*w* cm^−1^; ^1^H NMR:δ = 7.80–7.78 (*m*, 1H, 2-H), 7.66 (*ddd*, *J* = 7.8, 2.0, 1.1 Hz, 1H, 6-H), 7.60 (*ddd*, *J* = 7.7, 1.8, 1.2 Hz, 1H, 4-H), 7.55–7.48 (*m*, 2H, 5-H, NH), 4.29 (*t*, *J* = 5.1 Hz, 1H, OH), 3.36–3.30 (*m*, 2H, 14-H), 2.72 (*td*, *J* = 7.0, 5.7 Hz, 2H, 9-H), 1.37–1.32 (*m*, 4H, 10-H, 13-H), 1.31 (*s*, 9H, 8-H, 8′-H, 8′-H), 1.22–1.12 (*m*, 4H, 11-H, 12-H) ppm; ^13^C NMR:δ = 151.9 (C-3), 140.5 (C-1), 129.2 (C-4), 128.9 (C-5), 123.7 (C-2), 122.8 (C-6), 60.6 (C-14), 42.5 (C-9), 34.7 (C-7), 32.3 (C-13), 30.9 (C-8), 28.9 (C-10), 25.9 (C-11), 25.0 (C-12) ppm; MS: *m*/*z* = 336.2 (100%, [M + Na]^+^); anal. calcd. for C_16_H_27_NSO_3_ (313.46): C 61.31, H 8.68, N 4.47; found: C 61.07, H 9.01, N 4.15.

#### 4.2.126. 6-[(3-(tert-Butyl)phenyl)sulfonamido]hexyl Sulfamate (**62b**)

Applying GPB: from **62a** (200 mg, 0.64 mmol): **62b** (160 mg, 64%); oil; R_f_ = 0.73 (CHCl_3_/EtOAc, 2:3); UV–Vis: 225 nm (3.84); IR: ν = 3276*w*, 2960*w*, 2867*w*, 1561*vw*, 1481*w*, 1462*w*, 1417*w*, 1364*s*, 1325*m*, 1307*m*, 1267*w*, 1177*s*, 1156*vs*, 1125*m*, 1098*m*, 1088*m*, 923*s*, 798*m*, 773*w*, 725*w*, 697*m*, 678*m*, 626*m*, 587*vs*, 551*s*, 535*m*, 465*w* cm^−1^; ^1^H NMR:δ = 7.80–7.78 (*m*, 1H, 2-H), 7.67 (*ddd*, *J* = 7.8, 2.1, 1.2 Hz, 1H, 6-H), 7.62–7.58 (*m*, 1H, 4-H), 7.57–7.48 (*m*, 2H, 5-H, NH), 7.36 (*s*, 2H, NH_2_), 3.96 (*t*, *J* = 6.5 Hz, 2H, 14-H), 2.73 (*q*, *J* = 6.6 Hz, 2H, 9-H), 1.60–1.50 (*m*, 2H, 13-H), 1.39–1.28 (*m*, 2H, 10-H), 1.31 (*s*, 9H, 8-H, 8′-H, 8′-H), 1.27–1.17 (*m*, 4H, 11-H, 12-H) ppm; ^13^C NMR (126 MHz, DMSO-*d*_6_): δ = 151.9 (C-3), 140.5 (C-1), 129.2 (C-4), 128.9 (C-5), 123.7 (C-2), 122.8 (C-6), 68.9 (C-14), 42.4 (C-9), 34.7 (C-7), 30.9 (C-8), 28.8 (C-13), 28.2 (C-10), 25.5 (C-12), 24.6 (C-11) ppm; MS: *m*/*z* = 415.6 (100%, [M + Na]^+^); anal. calcd. for C_16_H_28_N_2_S_2_O_5_ (392.53): C 48.96, H 7.19, N 7.14; found: C 48.71, H 7.32, N 6.84.

#### 4.2.127. 3-(tert-Butyl)-N-(7-hydroxyheptyl)benzene Sulfonamide (**63a**)

Applying GPA: from 3-(tert-butyl)benzenesulfonyl chloride (500 mg, 2.15 mmol) and 7-amino-heptanol (422 mg, 3.22 mmol): **63a** (635 mg, 90%); oil; R_f_ = 0.25 (petrolether/EtOAc, 2:3); UV–Vis: 225 nm (4.00); IR: ν = 3503*vw*, 3279*w*, 2932*m*, 2860*w*, 1481*w*, 1461*w*, 1416*w*, 1397*w*, 1366*w*, 1325*m*, 1308*m*, 1267*w*, 1156*vs*, 1125*m*, 1098*m*, 1087*m*, 1056*m*, 999*w*, 897*w*, 797*w*, 773*w*, 724*w*, 697*s*, 678*m*, 626*m*, 587*vs*, 534*m*, 500*w*, 461*w*, 445*w* cm^−1^; ^1^H NMR:δ = 7.80–7.77 (*m*, 1H, 2-H), 7.66 (*ddd*, *J* = 7.8, 2.0, 1.1 Hz, 1H, 6-H), 7.60 (*ddd*, *J* = 7.7, 1.8, 1.1 Hz, 1H, 4-H), 7.55–7.47 (*m*, 2H, 5-H, NH), 4.29 (*t*, *J* = 5.1 Hz, 1H, OH), 3.40–3.31 (*m*, 2H, 15-H), 2.76–2.68 (*m*, 2H, 9-H), 1.40–1.32 (*m*, 4H, 10-H, 14-H), 1.31 (*s*, 9H, 8-H, 8′-H, 8′-H), 1.24–1.09 (*m*, 6H, 11-H, 12-H, 13-H) ppm; ^13^C NMR (126 MHz, DMSO-*d*_6_): δ = 151.8 (C-3), 140.5 (C-1), 129.2 (C-4), 128.9 (C-5), 123.7 (C-2), 122.8 (C-6), 60.7 (C-15), 42.5 (C-9), 34.7 (C-7), 32.4 (C-14), 30.9 (C-8), 28.8 (C-10), 28.4 (C-12), 26.0 (C-11), 25.3 (C-13) ppm; MS: *m*/*z* = 350.3 (100%, [M + Na]^+^); anal. calcd. for C_17_H_29_NSO_3_ (327.48): C 62.35, H 8.93, N 4.28; found: C 62.03, H 9.21, N 3.96.

#### 4.2.128. 7-[(3-(tert-Butyl)phenyl)sulfonamido]heptyl Sulfamate (**63b**)

Applying GPB: from **63a** (200 mg, 0.61 mmol): **63b** (134 mg, 54%); oil; R_f_ = 0.78 (CHCl_3_/EtOAc, 2:3); UV–Vis: 225 nm (4.10); IR: ν = 3277*w*, 2937*w*, 2862*w*, 1562*w*, 1481*w*, 1417*w*, 1364*s*, 1325*m*, 1307*m*, 1267*w*, 1177*s*, 1156*vs*, 1125*m*, 1098*m*, 1088*m*, 923*s*, 799*m*, 773*m*, 724*w*, 697*m*, 678*m*, 626*m*, 587*vs*, 551*s*, 495*w*, 460*w*, 443*vw* cm^−1^; ^1^H NMR:δ = 7.79 (*td*, *J* = 1.9, 0.5 Hz, 1H, 2-H), 7.67 (*ddd*, *J* = 7.8, 2.0, 1.1 Hz, 1H, 6-H), 7.60 (*ddd*, *J* = 7.7, 1.8, 1.2 Hz, 1H, 4-H), 7.56–7.49 (*m*, 2H, 5-H, NH), 7.37 (*s*, 2H, NH_2_), 3.98 (*t*, *J* = 6.5 Hz, 2H, 15-H), 2.73 (*td*, *J* = 6.9, 5.8 Hz, 2H, 9-H), 1.61–1.53 (*m*, 2H, 14-H), 1.31 (*s*, 9H, 8-H, 8′-H, 8′-H), 1.35–1.28 (*m*, 2H, 10-H), 1.27–1.14 (*m*, 6H, 11-H, 12-H, 13-H) ppm; ^13^C NMR:δ = 151.9 (C-3), 140.5 (C-1), 129.2 (C-4), 128.9 (C-5), 123.7 (C-2), 122.8 (C-6), 68.9 (C-15), 42.5 (C-9), 34.7 (C-7), 30.9 (C-8), 28.8 (C-14), 28.2 (C-11), 28.0 (C-10), 25.8 (C-13), 24.9 (C-12) ppm; MS: *m*/*z* = 429.7 (100%, [M + Na]^+^); anal. calcd. for C_17_H_30_N_2_S_2_O_5_ (406.56): C 50.22, H 7.44, N 6.89; found: C 49.96, H 7.79, N 6.65.

#### 4.2.129. 3-(tert-Butyl)-N-(8-hydroxyoctyl)benzene Sulfonamide (**64a**)

Applying GPA: from 3-(tert-butyl)benzenesulfonyl chloride (500 mg, 2.15 mmol) and 8-amino-octanol (468 mg, 3.22 mmol): **64a** (703 mg, 96%); oil; R_f_ = 0.30 (petrolether/EtOAc, 2:3); UV–Vis: 225 nm (3.92); IR: ν = 3503*vw*, 3279*w*, 2930*m*, 2857*m*, 1481*w*, 1461*w*, 1416*w*, 1397*w*, 1366*w*, 1325*m*, 1308*m*, 1267*w*, 1157*vs*, 1125*m*, 1098*m*, 1086*m*, 998*vw*, 898*w*, 797*m*, 774*w*, 722*w*, 697*s*, 678*m*, 626*m*, 587*vs*, 534*m*, 515*m* cm^−1^; ^1^H NMR:δ = 7.79 (*td*, *J* = 1.9, 0.5 Hz, 1H, 2-H), 7.66 (*ddd*, *J* = 7.8, 2.0, 1.1 Hz, 1H, 6-H), 7.60 (*ddd*, *J* = 7.7, 1.8, 1.1 Hz, 1H, 4-H), 7.54–7.48 (*m*, 2H, 5-H, NH), 4.29 (*t*, *J* = 5.1 Hz, 1H, OH), 3.38–3.33 (*m*, 2H, 16-H), 2.72 (*td*, *J* = 6.9, 2.7 Hz, 2H, 9-H), 1.41–1.32 (*m*, 4H, 10-H, 15-H), 1.31 (*s*, 9H, 8-H, 8′-H, 8′-H), 1.25–1.09 (*m*, 8H, 11-H, 12-H, 13-H, 14-H) ppm; ^13^C NMR:δ = 151.9 (C-3), 140.6 (C-1), 129.2 (C-4), 128.9 (C-5), 123.7 (C-2), 122.9 (C-6), 60.7 (C-16), 42.5 (C-9), 34.7 (C-7), 32.5 (C-15), 30.9 (C-8), 28.8 (C-10), 28.8 (C-12), 28.6 (C-13), 25.9 (C-11), 25.4 (C-14) ppm; MS: *m*/*z* = 364.4 (100%, [M + Na]^+^); anal. calcd. for C_18_H_31_NSO_3_ (341.51): C 63.31, H 9.15, N 4.10; found: C 63.07, H 9.42, N 3.91.

#### 4.2.130. 8-[(3-(tert-Butyl)phenyl)sulfonamido]octyl Sulfamate (**64b**)

Applying GPB: from **64a** (200 mg, 0.59 mmol): **64b** (221 mg, 90%); white solid; R_f_ = 0.83 (CHCl_3_/EtOAc, 2:3); m.p. = 57–59 °C; UV–Vis: 225 nm (3.99); IR: ν = 3364*w*, 3279*m*, 2967*w*, 2928*m*, 2858*w*, 1558*w*, 1481*w*, 1475*w*, 1466*w*, 1428*w*, 1399*w*, 1378*s*, 1322*m*, 1310*s*, 1289*m*, 1270*w*, 1178*s*, 1170*s*, 1158*vs*, 1127*m*, 1099*w*, 1070*w*, 1057*m*, 1037*m*, 999*w*, 959*vs*, 909*s*, 896*s*, 820*s*, 793*m*, 775*m*, 761*w*, 725*m*, 706*m*, 692*vs*, 667*m*, 628*m*, 621*m*, 588*vs*, 554*s*, 535*s*, 510*s*, 485*m* cm^−1^; ^1^H NMR:δ = 7.79 (*td*, *J* = 1.9, 0.5 Hz, 1H, 2-H), 7.67 (*ddd*, *J* = 7.8, 2.0, 1.1 Hz, 1H, 6-H), 7.60 (*ddd*, *J* = 7.7, 1.8, 1.2 Hz, 1H, 4-H), 7.56–7.48 (*m*, 2H, 5-H, NH), 7.37 (*s*, 2H, NH_2_), 3.99 (*t*, *J* = 6.5 Hz, 2H, 16-H), 2.72 (*td*, *J* = 7.0, 5.8 Hz, 2H, 9-H), 1.63–1.54 (*m*, 2H, 15-H), 1.36–1.21 (*m*, 4H, 10-H, 14-H), 1.31 (*s*, 9H, 8-H, 8′-H, 8′-H), 1.22–1.11 (*m*, 6H, 11-H, 12-H, 13-H) ppm; ^13^C NMR (126 MHz, DMSO-*d*_6_): δ = 151.9 (C-3), 140.5 (C-1), 129.2 (C-4), 128.9 (C-5), 123.7 (C-2), 122.8 (C-6), 68.9 (C-16), 42.5 (C-9), 34.7 (C-7), 30.9 (C-8), 28.8 (C-15), 28.4 (C-13), 28.3 (C-10), 28.3 (C-12), 25.9 (C-11), 24.9 (C-14) ppm; MS: *m*/*z* = 443.8 (100%, [M + Na]^+^); anal. calcd. for C_18_H_32_N_2_S_2_O_5_ (420.58): C 51.40, H 7.67, N 6.66; found: C 51.17, H 7.96, N 6.45.

#### 4.2.131. 2-(tert-Butyl)-N-(5-hydroxypentyl)benzene Sulfonamide (**65a**)

Applying GPA: from 2-(tert-butyl)benzenesulfonyl chloride (330 mg, 1.42 mmol) and 5-amino-pentanol (219 mg, 2.13 mmol): **65a** (352 mg, 83%); oil; R_f_ = 0.23 (petrolether/EtOAc, 2:3); UV–Vis: 221 nm (3.83); IR: ν = 3493*vw*, 3294*w*, 3060*vw*, 2938*m*, 2867*w*, 1463*w*, 1432*w*, 1400*w*, 1366*w*, 1314*s*, 1260*w*, 1243*w*, 1199*vw*, 1175*w*, 1152*vs*, 1128*m*, 1111*m*, 1082*m*, 1057*m*, 1044*m*, 934*vw*, 874*vw*, 841*vw*, 760*m*, 734*m*, 662*m*, 592*vs*, 546*s*, 475*w*, 434*vw* cm^−1^; ^1^H NMR (500 MHz, DMSO-*d*_6_): δ = 7.89 (*dd*, *J* = 8.0, 1.5 Hz, 1H, 3-H), 7.83 (*t*, *J* = 5.6 Hz, 1H, NH), 7.66 (*dd*, *J* = 8.2, 1.4 Hz, 1H, 6-H), 7.51 (*td*, *J* = 7.6, 1.5 Hz, 1H, 4-H), 7.40 (*td*, *J* = 7.6, 1.4 Hz, 1H, 5-H), 4.32 (*t*, *J* = 5.1 Hz, 1H, OH), 3.36 (*td*, *J* = 6.4, 5.0 Hz, 2H, 13-H), 2.81 (*q*, *J* = 6.6 Hz, 2H, 9-H), 1.53–1.50 (*m*, 9H, 8-H, 8′-H, 8′-H), 1.48–1.25 (*m*, 6H, 10-H, 11-H, 12-H) ppm; ^13^C NMR (126 MHz, DMSO-*d*_6_): δ = 148.9 (C-2), 140.7 (C-1), 131.7 (C-4), 129.4 (C-3), 128.9 (C-6), 126.4 (C-5), 60.5 (C-13), 42.8 (C-9), 36.7 (C-7), 32.0 (C-12), 31.8 (C-8), 29.1 (C-10), 22.6 (C-11) ppm; MS: *m*/*z* = 322.3 (100%, [M + Na]^+^); anal. calcd. for C_15_H_25_NSO_3_ (299.43): C 60.17, H 8.42, N 4.68; found: C 59.82, H 8.69, N 4.32.

#### 4.2.132. 5-[(2-(tert-Butyl)phenyl)sulfonamido]pentyl Sulfamate (**65b**)

Applying GPB: from **65a** (120 mg, 0.4 mmol): **65b** (88 mg, 58%); oil; R_f_ = 0.66 (CHCl_3_/EtOAc, 2:3); UV–Vis: 221 nm (3.98); IR: ν = 3283*w*, 2941*w*, 2867*w*, 1566*w*, 1464*w*, 1432*w*, 1362*s*, 1313*s*, 1260*w*, 1243*w*, 1176*s*, 1152*vs*, 1128*m*, 1111*m*, 1083*w*, 1059*w*, 923*s*, 839*w*, 822*w*, 761*s*, 733*m*, 662*m*, 593*vs*, 550*vs* cm^−1^; ^1^H NMR:δ = 7.80–7.77 (*m*, 1H, 6-H), 7.67 (*ddd*, *J* = 7.8, 2.0, 1.1 Hz, 1H, 3-H), 7.60 (*ddd*, *J* = 7.7, 1.8, 1.2 Hz, 1H, 5-H), 7.56 (*t*, *J* = 5.9 Hz, 1H, NH), 7.52 (*t*, *J* = 7.8 Hz, 1H, 4-H), 7.37 (*s*, 2H, NH_2_), 3.95 (*t*, *J* = 6.5 Hz, 2H, 13-H), 2.73 (*q*, *J* = 6.6 Hz, 2H, 9-H), 1.58–1.50 (*m*, 2H, 12-H), 1.42–1.33 (*m*, 2H, 10-H), 1.31 (*s*, 9H, 8-H, 8′-H, 8′-H), 1.34–1.22 (*m*, 2H, 11-H) ppm; ^13^C NMR:δ = 151.9 (C-2), 140.4 (C-1), 129.3 (C-4), 128.9 (C-3), 123.7 (C-6), 122.8 (C-5), 68.8 (C-13), 42.3 (C-9), 34.7 (C-7), 30.9 (C-8), 28.4 (C-12), 27.8 (C-10), 22.2 (C-11) ppm; MS: *m*/*z* = 401.6 (100%, [M + Na]^+^); anal. calcd. for C_15_H_26_N_2_S_2_O_5_ (378.50): C 47.60, H 6.92, N 7.40; found: C 47.26, H 7.17, N 7.13.

#### 4.2.133. 2-(tert-Butyl)-N-(6-hydroxyhexyl)benzene Sulfonamide (**66a**)

Applying GPA: from 2-(tert-butyl)benzenesulfonyl chloride (330 mg, 1.42 mmol) and 6-amino-hexanol (249 mg, 2.13 mmol): **66a** (419 mg, 94%); oil; R_f_ = 0.30 (petrolether/EtOAc, 2:3); UV–Vis: 221 nm (3.78); IR: ν = 3497*vw*, 3295*w*, 2934*m*, 2863*w*, 1463*w*, 1432*w*, 1400*w*, 1366*w*, 1314*s*, 1260*w*, 1242*w*, 1199*vw*, 1175*w*, 1152*vs*, 1128*m*, 1111*m*, 1056*m*, 842*vw*, 760*m*, 734*m*, 662*m*, 592*vs*, 546*m*, 476*w* cm ^−1^; ^1^H NMR:δ = 7.89 (*dd*, *J* = 8.0, 1.5 Hz, 1H, 3-H), 7.82 (*t*, *J* = 5.8 Hz, 1H, NH), 7.66 (*dd*, *J* = 8.1, 1.3 Hz, 1H, 6-H), 7.51 (*ddd*, *J* = 8.0, 7.2, 1.5 Hz, 1H, 4-H), 7.43–7.37 (*m*, 1H, 5-H), 4.31 (*t*, *J* = 5.2 Hz, 1H, OH), 3.36 (*td*, *J* = 6.5, 5.2 Hz, 2H, 14-H), 2.81 (*td*, *J* = 7.1, 5.8 Hz, 2H, 9-H), 1.51 (*s*, 9H, 8-H, 8′-H, 8′-H), 1.48–1.33 (*m*, 4H, 10-H, 13-H), 1.31–1.19 (*m*, 4H, 11-H, 12-H) ppm; ^13^C NMR (126 MHz, DMSO-*d*_6_): δ = 148.8 (C-2), 140.7 (C-1), 131.7 (C-4), 129.4 (C-3), 128.9 (C-6), 126.4 (C-5), 60.6 (C-14), 42.8 (C-9), 36.7 (C-7), 32.4 (C-13), 31.8 (C-8), 29.3 (C-10), 26.0 (C-12), 25.1 (C-11) ppm; MS: *m*/*z* = 336.3 (100%, [M + Na]^+^); anal. calcd. for C_16_H_27_NSO_3_ (313.46): C 61.31, H 8.68, N 4.47; found: C 61.04, H 8.94, N 4.11.

#### 4.2.134. 6-[(2-(tert-Butyl)phenyl)sulfonamido]hexyl Sulfamate (**66b**)

Applying GPB: from **66a** (120 mg, 0.38 mmol): **66b** (141 mg, 94%); oil; R_f_ = 0.70 (CHCl_3_/EtOAc, 2:3); UV–Vis: 221 nm (4.00); IR: ν = 3283*w*, 2941*w*, 2867*w*, 1566*w*, 1464*w*, 1432*w*, 1362*s*, 1313*s*, 1260*w*, 1243*w*, 1176*s*, 1152*vs*, 1128*m*, 1111*m*, 1083*w*, 1059*w*, 923*s*, 839*w*, 822*w*, 761*s*, 733*m*, 662*m*, 593*vs*, 550*vs* cm ^−1^; ^1^H NMR:δ = 7.89 (*dd*, *J* = 8.0, 1.5 Hz, 1H, 3-H), 7.84 (*t*, *J* = 5.8 Hz, 1H, NH), 7.66 (*dd*, *J* = 8.1, 1.4 Hz, 1H, 6-H), 7.51 (*ddd*, *J* = 7.9, 7.2, 1.5 Hz, 1H, 4-H), 7.44–7.39 (*m*, 1H, 5-H), 7.38 (*s*, 2H, NH_2_), 3.99 (*t*, *J* = 6.5 Hz, 2H, 14-H), 2.81 (*td*, *J* = 7.0, 5.8 Hz, 2H, 9-H), 1.64–1.54 (*m*, 2H, 13-H), 1.51 (*s*, 9H, 8-H, 8′-H, 8′-H), 1.49–1.41 (*m*, 2H, 10-H), 1.33–1.25 (*m*, 4H, 11-H, 12-H) ppm; ^13^C NMR:δ = 148.9 (C-2), 140.7 (C-1), 131.8 (C-4), 129.4 (C-3), 129.0 (C-6), 126.4 (C-5), 68.9 (C-14), 42.7 (C-9), 36.7 (C-7), 31.8 (C-8), 29.1 (C-13), 28.2 (C-10), 25.6 (C-12), 24.6 (C-11) ppm; MS: *m*/*z* = 415.6 (100%, [M + Na]^+^); anal. calcd. for C_16_H_28_N_2_S_2_O_5_ (392.53): C 48.96, H 7.19, N 7.14; found: C 48.75, H 7.43, N 6.87.

#### 4.2.135. 2-(tert-Butyl)-N-(7-hydroxyheptyl)benzene Sulfonamide (**67a**)

Applying GPA: from 2-(tert-butyl)benzenesulfonyl chloride (330 mg, 1.42 mmol) and 7-amino-heptanol (279 mg, 2.13 mmol): **67a** (411 mg, 89%); oil; R_f_ = 0.34 (petrolether/EtOAc, 2:3); UV–Vis: 221 nm (4.03); IR: ν = 3496*vw*, 3295*w*, 2931*m*, 2859*w*, 1463*w*, 1432*w*, 1400*w*, 1366*w*, 1315*s*, 1260*w*, 1242*w*, 1199*vw*, 1175*vw*, 1152*s*, 1128*m*, 1111*m*, 1057*m*, 866*vw*, 840*vw*, 760*m*, 734*m*, 662*m*, 593*vs*, 547*m*, 477*w*, 428*vw* cm^−1^; ^1^H NMR:δ = 7.90 (*dt*, *J* = 8.0, 1.3 Hz, 1H, 3-H), 7.82 (*s*, 1H, NH), 7.66 (*dt*, *J* = 8.1, 1.3 Hz, 1H, 6-H), 7.54–7.47 (*m*, 1H, 4-H), 7.44–7.37 (*m*, 1H, 5-H), 4.23 (*s*, 1H, OH), 3.40–3.33 (*m*, 2H, 15-H), 2.85–2.76 (*m*, 2H, 9-H), 1.56–1.48 (*m*, 9H, 8-H, 8′-H, 8′-H), 1.47–1.33 (*m*, 4H, 10-H, 14-H), 1.30–1.16 (*m*, 6H, 11-H, 12-H, 13-H) ppm; ^13^C NMR (126 MHz, DMSO-*d*_6_): δ = 148.8 (C-2), 140.7 (C-1), 131.7 (C-4), 129.5 (C-3), 128.9 (C-6), 126.4 (C-5), 60.7 (C-15), 42.7 (C-9), 36.7 (C-7), 32.4 (C-14), 31.8 (C-8), 29.2 (C-10), 28.5 (C-12), 26.1 (C-11), 25.4 (C-13) ppm; MS: *m*/*z* = 350.3 (100%, [M + Na]^+^); anal. calcd. for C_17_H_29_NSO_3_ (327.48): C 62.35, H 8.93, N 4.28; found: C 62.02, H 9.25, N 3.96. 

#### 4.2.136. 7-[(2-(tert-Butyl)phenyl)sulfonamido]heptyl Sulfamate (**67b**)

Applying GPB: from **67a** (150 mg, 0.46 mmol): **67b** (165 mg, 89%); oil; R_f_ = 0.76 (CHCl_3_/EtOAc, 2:3); UV–Vis: 221 nm (3.87); IR: ν = 3283*w*, 2936*w*, 2862*w*, 1566*w*, 1464*w*, 1432*w*, 1362*s*, 1314*s*, 1177*s*, 1152*vs*, 1128*m*, 1111*m*, 1083*w*, 1058*w*, 1000*w*, 924*s*, 838*w*, 814*w*, 762*s*, 734*m*, 662*m*, 593*vs*, 550*vs*, 500*m* cm^−1^; ^1^H NMR:δ = 7.89 (*dd*, *J* = 8.0, 1.5 Hz, 1H, 3-H), 7.83 (*t*, *J* = 5.8 Hz, 1H, NH), 7.66 (*dd*, *J* = 8.1, 1.3 Hz, 1H, 6-H), 7.51 (*td*, *J* = 8.1, 7.7, 1.5 Hz, 1H, 4-H), 7.44–7.38 (*m*, 1H, 5-H), 7.37 (*s*, 2H, NH_2_), 3.99 (*t*, *J* = 6.5 Hz, 2H, 15-H), 2.81 (*td*, *J* = 7.0, 5.8 Hz, 2H, 9-H), 1.64–1.55 (*m*, 2H, 14-H), 1.51 (*s*, 9H, 8-H, 8′-H, 8′-H), 1.48–1.39 (*m*, 2H, 10-H), 1.35–1.21 (*m*, 6H, 11-H, 12-H, 13-H) ppm; ^13^C NMR:δ = 148.8 (C-2), 140.7 (C-1), 131.8 (C-4), 129.5 (C-3), 129.0 (C-6), 126.4 (C-5), 69.0 (C-15), 42.7 (C-9), 36.7 (C-7), 31.8 (C-8), 29.1 (C-14), 28.2 (C-11), 28.0 (C-10), 25.9 (C-13), 25.0 (C-12) ppm; MS: *m*/*z* = 429.6 (100%, [M + Na]^+^); anal. calcd. for C_17_H_30_N_2_S_2_O_5_ (406.56): C 50.22, H 7.44, N 6.89; found: C 49.94, H 7.76, N 6.53. 

#### 4.2.137. 4-Cyclohexyl-N-(2-hydroxyethyl)benzene Sulfonamide (**68a**) [919974-61-1]

Applying GPA: from 4-cyclohexylbenzenesulfonyl chloride (500 mg, 1.93 mmol) and 2-amino-ethanol (177 mg, 2.90 mmol): **68a** (453 mg, 83%); oil; R_f_ = 0.56 (CHCl_3_/EtOAc, 2:3); UV–Vis: 229 nm (4.19); IR: ν = 3514*vw*, 3264*w*, 3146*w*, 2920*m*, 2848*m*, 1597*w*, 1496*vw*, 1483*vw*, 1461*w*, 1451*m*, 1427*w*, 1409*w*, 1348*w*, 1319*s*, 1278*w*, 1260*w*, 1216*vw*, 1188*w*, 1156*s*, 1134*w*, 1093*s*, 1059*m*, 1036*m*, 1018*w*, 996*w*, 953*m*, 890*w*, 864*vw*, 842*w*, 826*s*, 802*w*, 782*vw*, 731*w*, 701*s*, 631*w*, 597*s*, 574*vs*, 527*m*, 504*w*, 491*w*, 479*w*, 458*m* cm^−1^; ^1^H NMR:δ = 7.72–7.68 (*m*, 2H, 2-H, 2′-H), 7.51–7.46 (*m*, 1H, NH), 7.45–7.40 (*m*, 2H, 3-H, 3′-H), 4.66 (*t*, *J* = 5.6 Hz, 1H, OH), 3.37 (*q*, *J* = 6.3 Hz, 2H, 10-H), 2.77 (*q*, *J* = 5.9 Hz, 2H, 9-H), 2.64–2.55 (*m*, 1H, 5-H), 1.84–1.74 (*m*, 4H, 6-H_a_, 6′-H_a_, 7-H_a_, 7′-H_a_), 1.74–1.66 (*m*, 1H, 8-H_a_), 1.50–1.30 (*m*, 4H, 6-H_b_, 6′-H_b_, 7-H_b_, 7′-H_b_), 1.30–1.17 (*m*, 1H, 8-H_b_) ppm; ^13^C NMR:δ = 152.1 (C-4), 138.0 (C-1), 127.4 (C-2), 126.6 (C-3), 59.9 (C-10), 45.1 (C-9), 43.6 (C-5), 33.5 (C-6), 26.2 (C-7), 25.4 (C-8) ppm; MS: *m*/*z* = 305.9 (90%, [M + Na]^+^); anal. calcd. for C_14_H_21_NSO_3_ (283.39): C 59.34, H 7.47, N 4.94; found: C 59.11, H 7.85, N 4.65.

#### 4.2.138. 2-[(4-Cyclohexylphenyl)sulfonamido]ethyl Sulfamate (**68b**)

Applying GPB: from **68a** (200 mg, 0.71 mmol): **68b** (232 mg, 91%); white solid; R_f_ = 0.77 (CHCl_3_/EtOAc, 2:3); m.p. = 109–110 °C; UV–Vis: 229 nm (4.28); IR: ν = 3358*m*, 3256*w*, 2922*m*, 2849*w*, 1599*w*, 1549*w*, 1462*w*, 1452*w*, 1412*m*, 1363*s*, 1314*s*, 1279*w*, 1252*vw*, 1181*s*, 1154*vs*, 1095*m*, 1078*w*, 1010*m*, 998*m*, 957*s*, 903*m*, 828*m*, 781*m*, 748*s*, 723*m*, 702*s*, 634*w*, 610*s*, 563*s*, 549*vs*, 525*m*, 501*m*, 465*m*, 449*m* cm^−1^; ^1^H NMR:δ = 7.82 (*t*, *J* = 6.0 Hz, 1H, NH), 7.73–7.69 (*m*, 2H, 2-H, 2′-H), 7.50 (*s*, 2H, NH_2_), 7.47–7.42 (*m*, 2H, 3-H, 3′-H), 4.00 (*t*, *J* = 5.7 Hz, 2H, 10-H), 3.03 (*q*, *J* = 5.8 Hz, 2H, 9-H), 2.64–2.55 (*m*, 1H, 5-H), 1.84–1.76 (*m*, 4H, 6-H_a_, 6′-H_a_, 7-H_a_, 7′-H_a_), 1.75–1.67 (*m*, 1H, 8-H_a_), 1.49–1.30 (*m*, 5H, 6-H_b_, 6′-H_b_, 7-H_b_, 7′-H_b_), 1.30–1.18 (*m*, 1H, 8-H_b_) ppm; ^13^C NMR:δ = 152.4 (C-1), 137.7 (C-4), 127.5 (C-2), 126.6 (C-3), 67.6 (C-10), 43.6 (C-5), 41.5 (C-9), 33.5 (C-6), 26.2 (C-7), 25.4 (C-8) ppm; MS: *m*/*z* = 385.5 (100%, [M + Na]^+^); anal. calcd. for C_14_H_22_N_2_S_2_O_5_ (362.46): C 46.39, H 6.12, N 7.73; found: C 46.16, H 6.38, N 7.41.

#### 4.2.139. 4-Cyclohexyl-N-(3-hydroxypropyl)benzene Sulfonamide (**69a**) [919974-63-3]

Applying GPA: from 4-cyclohexylbenzenesulfonyl chloride (500 mg, 1.93 mmol) and 3-amino-propanol (218 mg, 2.90 mmol): **69a** (470 mg, 82%); white solid; R_f_ = 0.53 (CHCl_3_/EtOAc, 2:3); m.p. = 78–79 °C; UV–Vis: 229 nm (4.13); IR: ν = 3471*w*, 3167*w*, 2929*m*, 2851*m*, 1598*w*, 1494*vw*, 1472*w*, 1463*w*, 1448*w*, 1424*w*, 1407*w*, 1375*vw*, 1351*vw*, 1310*s*, 1283*w*, 1230*vw*, 1185*w*, 1157*vs*, 1096*m*, 1075*s*, 1005*m*, 964*m*, 943*w*, 876*m*, 853*w*, 838*w*, 827*m*, 803*w*, 781*w*, 740*m*, 698*m*, 595*vs*, 574*s*, 507*m*, 481*w*, 455*w* cm^−1^; ^1^H NMR:δ = 7.72–7.66 (*m*, 2H, 2-H, 2′-H), 7.47–7.37 (*m*, 3H, NH, 3-H, 3′-H), 4.39 (*t*, *J* = 5.1 Hz, 1H, OH), 3.39–3.33 (*m*, 2H, 11-H), 2.80–2.72 (*m*, 2H, 9-H), 2.59 (*td*, *J* = 9.4, 4.7 Hz, 1H, 5-H), 1.83–1.75 (*m*, 4H, 6-H_a_, 6′-H_a_, 7-H_a_, 7′-H_a_), 1.74–1.67 (*m*, 1H, 8-H_a_), 1.52 (*dt*, *J* = 13.3, 6.3 Hz, 2H, 10-H), 1.48–1.31 (*m*, 4H, 6-H_b_, 6′-H_b_, 7-H_b_, 7′-H_b_), 1.29–1.18 (*m*, 1H, 8-H_b_) ppm; ^13^C NMR:δ = 152.1 (C-4), 137.9 (C-1), 127.4 (C-2), 126.6 (C-3), 58.1 (C-11), 43.6 (C-5), 40.0 (C-9), 33.5 (C-6), 32.4 (C-10), 26.2 (C-7), 25.4 (C-8) ppm; MS: *m*/*z* = 319.9 (90%, [M + Na]^+^); anal. calcd. for C_15_H_23_NSO_3_ (297.41): C 60.58, H 7.80, N 4.71; found: C 60.44, H 8.02, N 4.54.

#### 4.2.140. 3-[(4-Cyclohexylphenyl)sulfonamido]propyl Sulfamate (**69b**)

Applying GPB: from **69a** (200 mg, 0.67 mmol): **69b** (210 mg, 83%); white solid; R_f_ = 0.74 (CHCl_3_/EtOAc, 2:3); m.p. = 113–114 °C; UV–Vis: 229 nm (4.27); IR: ν = 3351*w*, 3283*m*, 2929*m*, 2851*w*, 1598*w*, 1567*w*, 1474*w*, 1438*w*, 1403*w*, 1371*vs*, 1323*m*, 1307*s*, 1281*w*, 1256*w*, 1177*vs*, 1152*vs*, 1093*m*, 1070*m*, 1039*m*, 999*w*, 944*s*, 921*m*, 888*m*, 836*s*, 824*s*, 778*w*, 756*m*, 729*m*, 706*s*, 654*m*, 630*m*, 599*s*, 593*s*, 568*vs*, 548*s*, 527*m*, 515*m*, 506*m*, 486*w*, 428*w* cm^−1^; ^1^H NMR:δ = 7.74–7.67 (*m*, 2H, 2-H, 2′-H), 7.57–7.36 (*m*, 5H, 3-H, 3′-H, NH_2_, NH), 4.02 (*t*, *J* = 6.3 Hz, 2H, 11-H), 2.80 (*t*, *J* = 7.1 Hz, 2H, 9-H), 2.64–2.55 (*m*, 1H, 5-H), 1.84–1.67 (*m*, 7H, 6-H_a_, 6′-H_a_, 7-H_a_, 7′-H_a_, 8-H_a_, 10-H), 1.49–1.31 (*m*, 4H, 6-H_b_, 6′-H_b_, 7-H_b_, 7′-H_b_), 1.29–1.18 (*m*, 1H, 8-H_b_) ppm; ^13^C NMR:δ = 152.2 (C-1), 137.7 (C-4), 127.5 (C-2), 126.6 (C-3), 66.5 (C-11), 43.5 (C-5), 39.2 (C-9), 33.5 (C-6), 28.8 (C-10), 26.2 (C-7), 25.4 (C-8) ppm; MS: *m*/*z* = 399.5 (100%, [M + Na]^+^); anal. calcd. for C_15_H_24_N_2_S_2_O_5_ (376.49): C 47.85, H 6.43, N 7.44; found: C 47.57, H 6.61, N 7.21.

#### 4.2.141. 4-Cyclohexyl-N-(4-hydroxybutyl)benzene Sulfonamide (**70a**) [1976415-75-4]

Applying GPA: from 4-cyclohexylbenzenesulfonyl chloride (500 mg, 1.93 mmol) and 4-amino-butanol (258 mg, 2.90 mmol): **70a** (487 mg, 81%); white solid; R_f_ = 0.50 (CHCl_3_/EtOAc, 2:3); m.p. = 58–60 °C; UV–Vis: 229 nm (4.17); IR: ν = 3477*m*, 3101*w*, 2950*w*, 2922*m*, 2849*m*, 1446*m*, 1429*w*, 1396*w*, 1312*s*, 1257*w*, 1150*vs*, 1097*m*, 1081*m*, 1030*s*, 994*m*, 941*m*, 916*w*, 821*m*, 812*w*, 786*s*, 771*m*, 735*m*, 694*w*, 601*s*, 570*vs*, 537*m*, 508*w*, 485*w*, 438*w*, 490*m*, 476*w*, 441*w* cm^−1^; ^1^H NMR:δ = 7.71–7.66 (*m*, 2H, 2-H, 2′-H), 7.48–7.40 (*m*, 3H, NH, 3-H, 3′-H), 4.34 (*s*, 1H, OH), 3.33–3.28 (*m*, 2H, 12-H), 2.71 (*t*, *J* = 6.5 Hz, 2H, 9-H), 2.63–2.54 (*m*, 1H, 5-H), 1.83–1.75 (*m*, 4H, 6-H_a_, 6′-H_a_, 7-H_a_, 7′-H_a_), 1.75–1.66 (*m*, 1H, 8-H_a_), 1.47–1.30 (*m*, 8H, 6-H_b_, 6′-H_b_, 7-H_b_, 7′-H_b_, 10-H, 11-H), 1.29–1.20 (*m*, 1H, 8-H_b_) ppm; ^13^C NMR:δ = 152.0 (C-1), 138.1 (C-4), 127.4 (C-2), 126.5 (C-3), 60.2 (C-12), 43.6 (C-5), 42.5 (C-9), 33.5 (C-6), 29.5 (C-11), 26.2 (C-7), 25.8 (C-10), 25.4 (C-8) ppm; MS: *m*/*z* = 334.3 (100%, [M + Na]^+^); anal. calcd. for C_16_H_25_NSO_3_ (311.44): C 61.71, H 8.09, N 4.50; found: C 61.53, H 8.24, N 4.16.

#### 4.2.142. 4-[(4-Cyclohexylphenyl)sulfonamido]butyl Sulfamate (**70b**)

Applying GPB: from **70a** (200 mg, 0.64 mmol): **70b** (224 mg, 89%); white solid; R_f_ = 0.69 (CHCl_3_/EtOAc, 2:3); m.p. = 103–105 °C; UV–Vis: 229 nm (4.25); IR: ν = 3365*w*, 3308*w*, 3266*w*, 2923*m*, 2850*w*, 1598*w*, 1543*w*, 1466*w*, 1453*w*, 1411*m*, 1388*w*, 1366*s*, 1315*s*, 1279*w*, 1272*w*, 1180*m*, 1157*s*, 1132*m*, 1095*m*, 1065*w*, 1051*m*, 1019*m*, 997*w*, 958*m*, 921*m*, 872*w*, 827*m*, 790*s*, 737*m*, 710*s*, 656*m*, 631*m*, 597*m*, 588*m*, 571*s*, 550*vs*, 532*m*, 508*w*, 503*w*, 489*w*, 478*w*, 453*w*a cm^−1^; ^1^H NMR:δ = 7.73–7.67 (*m*, 2H, 2-H, 2′-H), 7.53 (*t*, *J* = 5.9 Hz, 1H, NH), 7.46–7.41 (*m*, 2H, 3-H, 3′-H), 7.38 (*s*, 2H, NH_2_), 3.96 (*t*, *J* = 6.3 Hz, 2H, 12-H), 2.75 (*q*, *J* = 6.7 Hz, 2H, 9-H), 2.64–2.55 (*m*, 1H, 5-H), 1.84–1.76 (*m*, 4H, 6-H_a_, 6′-H_a_, 7-H_a_, 7′-H_a_), 1.75–1.67 (*m*, 1H, 8-H_a_), 1.66–1.56 (*m*, 2H, 11-H), 1.51–1.31 (*m*, 6H, 6-H_b_, 6′-H_b_, 7-H_b_, 7′-H_b_, 10-H), 1.30–1.17 (*m*, 1H, 8-H_b_) ppm; ^13^C NMR:δ = 152.1 (C-1), 138.0 (C-4), 127.4 (C-2), 126.5 (C-3), 68.5 (C-12), 43.6 (C-5), 42.0 (C-9), 33.5 (C-6), 26.2 (C-7), 25.6 (C-10), 25.4 (C-8, C-11) ppm; MS: *m*/*z* = 413.7 (100%, [M + Na]^+^); anal. calcd. for C_16_H_26_N_2_S_2_O_5_ (390.51): C 49.21, H 6.71, N 7.17; found: C 48,97, H 6.97, N 6.86.

#### 4.2.143. 4-Cyclohexyl-N-(5-hydroxypentyl)benzene Sulfonamide (**71a**)

Applying GPA: from 4-cyclohexylbenzenesulfonyl chloride (500 mg, 1.93 mmol) and 5-amino-pentanol (299 mg, 2.90 mmol): **71a** (520 mg, 83%); oil; R_f_ = 0.49 (CHCl_3_/EtOAc, 2:3); UV–Vis: 229 nm (4.20); IR: ν = 3498*vw*, 3280*w*, 2924*m*, 2852*m*, 1598*w*, 1495*vw*, 1448*m*, 1410*w*, 1314*s*, 1283*w*, 1188*vw*, 1153*vs*, 1093*s*, 1040*m*, 999*w*, 920*vw*, 894*w*, 868*w*, 826*m*, 781*w*, 732*w*, 699*s*, 594*s*, 574*s*, 506*m*, 443*vw*, 508*w*, 485*w*, 438*w*, 490*m*, 476*w*, 441*w* cm^−1^; ^1^H NMR:δ = 7.71–7.66 (*m*, 2H, 2-H, 2′-H), 7.46–7.40 (*m*, 3H, 3-H, 3′-H, NH), 4.30 (*t*, *J* = 5.1 Hz, 1H, OH), 3.34–3.28 (*m*, 2H, 13-H), 2.70 (*td*, *J* = 7.0, 3.2 Hz, 2H, 9-H), 2.63–2.54 (*m*, 1H, 5-H), 1.83–1.76 (*m*, 4H, 6-H_a_, 6′-H_a_, 7-H_a_, 7′-H_a_), 1.74–1.66 (*m*, 1H, 8-H_a_), 1.48–1.15 (*m*, 11H, 6-H_b_, 6′-H_b_, 7-H_b_, 7′-H_b_, 8-H_b_, 10-H, 11-H, 12-H) ppm; ^13^C NMR (126 MHz, DMSO-*d*_6_): δ = 152.0 (C-4), 138.1 (C-1), 127.4 (C-3), 126.5 (C-2), 60.5 (C-13), 43.6 (C-5), 42.6 (C-9), 33.5 (C-6), 32.0 (C-12), 28.9 (C-10), 26.2 (C-7), 25.4 (C-8), 22.6 (C-11) ppm; MS: *m*/*z* = 348.4 (100%, [M + Na]^+^); anal. calcd. for C_17_H_27_NSO_3_ (325.47): C 62.74, H 8.36, N 4.30; found: C 62.51, H 8.63, N 4.17.

#### 4.2.144. 5-[(4-Cyclohexylphenyl)sulfonamido]pentyl Sulfamate (**71b**)

Applying GPB: from **71a** (200 mg, 0.61 mmol): **71b** (240 mg, 96%); white solid; R_f_ = 0.62 (CHCl_3_/EtOAc, 2:3); m.p. = 112–114 °C; UV–Vis: 229 nm (3.95); IR: ν = 3343*w*, 3277*m*, 3240*w*, 2934*m*, 2867*w*, 2848*w*, 1599*w*, 1471*w*, 1449*w*, 1444*w*, 1433*w*, 1404*w*, 1371*s*, 1356*m*, 1309*s*, 1278*w*, 1179*s*, 1154*vs*, 1093*m*, 1077*w*, 1052*m*, 968*s*, 920*s*, 886*m*, 866*w*, 821*vs*, 782*w*, 777*w*, 731*w*, 703*s*, 661*m*, 631*m*, 599*s*, 584*m*, 571*vs*, 558*s*, 537*s*, 521*m*, 504*m*, 478*w* cm^−1^; ^1^H NMR:δ = 7.71–7.67 (*m*, 2H, 2-H, 2′-H), 7.48 (*t*, *J* = 5.9 Hz, 1H, NH), 7.46–7.41 (*m*, 2H, 3-H, 3′-H), 7.37 (*s*, 2H, NH_2_), 3.96 (*t*, *J* = 6.5 Hz, 2H, 13-H), 2.72 (*q*, *J* = 6.6 Hz, 2H, 9-H), 2.64–2.55 (*m*, 1H, 5-H), 1.83–1.76 (*m*, 4H, 6-H_a_, 6′-H_a_, 7-H_a_, 7′-H_a_), 1.75–1.67 (*m*, 1H, 8-H_a_), 1.60–1.51 (*m*, 2H, 12-H), 1.49–1.34 (*m*, 6H, 6-H_b_, 6′-H_b_, 7-H_b_, 7′-H_b_, 10-H), 1.34–1.21 (*m*, 3H, 8-H_b_, 11-H) ppm; ^13^C NMR:δ = 152.1 (C-1), 138.0 (C-4), 127.4 (C-2), 126.5 (C-3), 68.8 (C-13), 43.5 (C-5), 42.3 (C-9), 33.5 (C-6), 28.5 (C-12), 27.8 (C-10), 26.2 (C-7), 25.4 (C-8), 22.2 (C-11) ppm; MS: *m*/*z* = 427.8 (100%, [M + Na]^+^); anal. calcd. for C_17_H_28_N_2_S_2_O_5_ (404.54): C 50.47, H 6.98, N 6.92; found: C 50.10, H 7.13, N 6.76.

#### 4.2.145. 4-Cyclohexyl-N-(6-hydroxyhexyl)benzene Sulfonamide (**72a**)

Applying GPA: from 4-cyclohexylbenzenesulfonyl chloride (500 mg, 1.93 mmol) and 6-amino-hexanol (340 mg, 2.90 mmol): **72a** (589 mg, 90%); white solid; R_f_ = 0.48 (CHCl_3_/EtOAc, 2:3); m.p. = 87–88 °C; UV–Vis: 229 nm (4.14); IR: ν = 3424*w*, 3365*vw*, 3261*m*, 2963*vw*, 2926*m*, 2855*m*, 1599*w*, 1479*w*, 1475*w*, 1462*vw*, 1451*w*, 1429*m*, 1411*w*, 1358*w*, 1316*s*, 1279*w*, 1187*w*, 1157*s*, 1103*w*, 1092*m*, 1063*m*, 1035*m*, 1016*w*, 996*w*, 984*vw*, 904*w*, 878*w*, 865*vw*, 844*w*, 827*m*, 800*w*, 780*w*, 735*m*, 709*s*, 679*m*, 598*s*, 571*vs*, 534*m*, 513*w*, 505*w*, 497*m* cm^−1^; ^1^H NMR:δ = 7.71–7.66 (*m*, 2H, 2-H, 2′-H), 7.47–7.39 (*m*, 3H, NH, 3-H, 3′-H), 4.29 (*s*, 1H, OH), 3.36–3.29 (*m*, 2H, 14-H), 2.75–2.66 (*m*, 2H, 9-H), 2.63–2.55 (*m*, 1H, 5-H), 1.82–1.75 (*m*, 4H, 6-H_a_, 6′-H_a_, 7-H_a_, 7′-H_a_), 1.74–1.67 (*m*, 1H, 8-H_a_), 1.48–1.29 (*m*, 8H, 6-H_b_, 6′-H_b_, 7-H_b_, 7′-H_b_, 10-H, 13-H), 1.28–1.21 (*m*, 1H, 8-H_b_), 1.21–1.14 (*m*, 4H, 11-H, 12-H) ppm; ^13^C NMR:δ = 152.0 (C-4), 138.1 (C-1), 127.4 (C-3), 126.5 (C-2), 60.6 (C-14), 43.6 (C-5), 42.5 (C-9), 33.5 (C-6), 32.3 (C-13), 29.0 (C-10), 26.2 (C-7), 25.9 (C-12), 25.4 (C-8), 25.0 (C-11) ppm; MS: *m*/*z* = 361.9 (100%, [M + Na]^+^); anal. calcd. for C_18_H_29_NSO_3_ (339.49): C 63.68, H 8.61, N 4.13; found: C 63.58, H 8.83, N 3.97.

#### 4.2.146. 6-[(4-Cyclohexylphenyl)sulfonamido]hexyl Sulfamate (**72b**)

Applying GPB: from **72a** (200 mg, 0.6 mmol): **72b** (186 mg, 75%); white solid; R_f_ = 0.63 (CHCl_3_/EtOAc, 2:3); m.p. = 86–88 °C; UV–Vis: 229 nm (3.81); IR: ν = 3370*w*, 3280*m*, 2929*w*, 2854*w*, 1558*w*, 1456*w*, 1356*s*, 1173*vs*, 1156*s*, 1094*w*, 995*s*, 922*s*, 828*w*, 770*vs*, 700*w*, 595*m*, 571*m*, 550*vs*, 493*m* cm^−1^; ^1^H NMR:δ = 7.71–7.66 (*m*, 2H, 2-H, 2′-H), 7.48–7.41 (*m*, 3H, NH, 3-H, 3′-H), 7.37 (*s*, 2H, NH_2_), 3.97 (*t*, *J* = 6.5 Hz, 2H, 14-H), 2.71 (*q*, *J* = 6.6 Hz, 2H, 9-H), 2.59 (*ddd*, *J* = 11.5, 8.4, 2.8 Hz, 1H, 5-H), 1.84–1.75 (*m*, 4H, 6-H_a_, 6′-H_a_, 7-H_a_, 7′-H_a_), 1.75–1.66 (*m*, 1H, 8-H_a_), 1.55 (*p*, *J* = 6.7 Hz, 2H, 13-H), 1.48–1.30 (*m*, 6H, 6-H_b_, 6′-H_b_, 7-H_b_, 7′-H_b_, 10-H), 1.29–1.17 (*m*, 5H, 8-H_b_, 11-H, 12-H); ^13^C NMR:δ = 152.3 (C-1), 138.2 (C-4), 127.5 (C-2), 126.7 (C-3), 69.1 (C-14), 43.7 (C-9), 42.5 (C-5), 33.7 (C-6), 29.0 (C-13), 28.3 (C-10), 26.3 (C-7), 25.6 (C-8), 24.7 (C-11) ppm; MS: *m*/*z* = 441.1 (100%, [M + Na]^+^), 419.1 (95%, [*M* + H]^+^); anal. calcd. for C_18_H_30_N_2_S_2_O_5_ (418.57): C 51.65, H 7.22, N 6.69; found: C 51.24, H 7.60, N 6.33. 

#### 4.2.147. 4-Cyclohexyl-N-(7-hydroxyheptyl)benzene Sulfonamide (**73a**)

Applying GPA: from 4-cyclohexylbenzenesulfonyl chloride (500 mg, 1.93 mmol) and 7-amino-heptanol (380 mg, 2.90 mmol): **73a** (592 mg, 87%); white solid; R_f_ = 0.47 (CHCl_3_/EtOAc, 2:3); m.p. = 67–68 °C; UV–Vis: 229 nm (4.15); IR: ν = 3272*m*, 2923*m*, 2852*m*, 1599*w*, 1474*w*, 1463*w*, 1449*w*, 1429*m*, 1409*w*, 1318*s*, 1281*w*, 1186*w*, 1156*vs*, 1093*m*, 1058*m*, 1034*m*, 999*w*, 894*w*, 867*w*, 847*w*, 823*m*, 780*w*, 707*s*, 665*m*, 632*m*, 598*s*, 570*vs*, 543*w*, 529*m*, 506*w* cm^−1^; ^1^H NMR:δ = 7.71–7.66 (*m*, 2H, 2-H, 2′-H), 7.46–7.40 (*m*, 3H, NH, 3H-, 3′-H), 4.29 (*t*, *J* = 5.1 Hz, 1H, OH), 3.35 (*td*, *J* = 6.7, 5.1 Hz, 2H, 15-H), 2.70 (*t*, *J* = 7.1 Hz, 2H, 9-H), 2.63–2.54 (*m*, 1H, 5-H), 1.84–1.75 (*m*, 4H, 6-H_a_, 6′-H_a_, 7-H_a_, 7′-H_a_), 1.74–1.66 (*m*, 1H, 8-H_a_), 1.48–1.26 (*m*, 8H, 6-H_b_, 6′-H_b_, 7-H_b_, 7′-H_b_, 10-H, 14-H), 1.26–1.11 (*m*, 7H, 8-H_b_, 11-H, 12-H, 13-H) ppm; ^13^C NMR:δ = 152.0 (C-1), 138.2 (C-4), 127.3 (C-2), 126.5 (C-3), 60.7 (C-15), 43.5 (C-5), 42.5 (C-9), 33.5 (C-6), 32.4 (C-14), 28.9 (C-10), 28.4 (C-11), 26.2 (C-7), 26.0 (C-13), 25.4 (C-8), 25.3 (C-12) ppm; MS: *m*/*z* = 375.8 (100%, [M + Na]^+^); anal. calcd. for C_19_H_31_NSO_3_ (353.52): C 64.55, H 8.84, N 3.96; found: C 64.12, H 9.12, N 3.64.

#### 4.2.148. 7-[(4-Cyclohexylphenyl)sulfonamido]heptyl Sulfamate (**73b**)

Applying GPB: from **73a** (200 mg, 0.57 mmol): **73b** (201 mg, 82%); white solid; R_f_ = 0.61 (CHCl_3_/EtOAc, 2:3); m.p. = 102–103 °C; UV–Vis: 229 nm (4.18); IR: ν = 3345*m*, 3273*m*, 3244*w*, 2928*m*, 2857*w*, 1598*w*, 1562*vw*, 1474*w*, 1452*w*, 1430*w*, 1411*vw*, 1394*w*, 1375*s*, 1309*s*, 1281*w*, 1177*s*, 1153*vs*, 1107*w*, 1093*m*, 1077*vw*, 1062*m*, 1049*w*, 1006*w*, 997*m*, 972*s*, 923*m*, 899*m*, 867*vw*, 835*w*, 813*s*, 781*w*, 731*m*, 708*s*, 668*m*, 598*m*, 590*m*, 568*s*, 550*s*, 532*m*, 515*m*, 507*w* cm^−1^; ^1^H NMR:δ = 7.71–7.66 (*m*, 2H, 2-H, 2′-H), 7.47–7.40 (*m*, 3H, 3-H, 3′-H, NH), 7.37 (*s*, 2H, NH_2_), 3.98 (*t*, *J* = 6.5 Hz, 2H, 15-H), 2.71 (*q*, *J* = 6.6 Hz, 2H, 9-H), 2.63–2.54 (*m*, 1H, 5-H), 1.84–1.75 (*m*, 4H, 6-H_a_, 6′-H_a_, 7-H_a_, 7′-H_a_), 1.74–1.67 (*m*, 1H, 8-H_a_), 1.63–1.53 (*m*, 2H, 14-H), 1.48–1.30 (*m*, 6H, 6-H_b_, 6′-H_b_, 7-H_b_, 7-H_b_, 10-H), 1.29–1.15 (*m*, 7H, 8-H_b_, 11-H, 12-H, 13-H) ppm; ^13^C NMR:δ = 152.1 (C-1), 138.1 (C-4), 127.4 (C-2), 126.6 (C-3), 69.0 (C-15), 43.6 (C-9), 42.5 (C-5), 33.6 (C-6), 28.9 (C-14), 28.2 (C-11), 28.0 (C-10), 26.2 (C-7), 25.9 (C-13), 25.5 (C-8), 24.9 (C-12) ppm; MS: *m*/*z* = 455.8 (100%, [M + Na]^+^); anal. calcd. for C_19_H_32_N_2_S_2_O_5_ (432.59): C 52.75, H 7.46, N 6.48; found: C 52.45, H 7.88, N 6.13.

#### 4.2.149. 4-Cyclohexyl-N-(8-hydroxyoctyl)benzene Sulfonamide (**74a**)

Applying GPA: from 4-cyclohexylbenzenesulfonyl chloride (500 mg, 1.93 mmol) and 8-amino-octanol (421 mg, 2.90 mmol): **74a** (618 mg, 87%); white solid; R_f_ = 0.45 (CHCl_3_/EtOAc, 2:3); UV–Vis: 229 nm (4.16); IR: ν = 3277*m*, 2924*s*, 2851*m*, 1599*w*, 1495*vw*, 1478*w*, 1466*w*, 1449*w*, 1427*m*, 1409*w*, 1318*s*, 1280*w*, 1185*w*, 1157*vs*, 1133*w*, 1094*m*, 1052*s*, 1016*w*, 997*w*, 982*w*, 901*m*, 883*w*, 866*w*, 849*w*, 823*m*, 780*w*, 755*m*, 733*m*, 706*s*, 651*m*, 632*m*, 598*s*, 570*vs*, 531*m*, 507*m*, 470*w* cm^−1^; ^1^H NMR (500 MHz, DMSO-*d*_6_): δ = 7.71–7.67 (*m*, 2H, 2-H, 2′-H), 7.46–7.42 (*m*, 2H, 3-H, 3′-H), 7.42–7.41 (*m*, 1H, NH), 4.29 (*t*, *J* = 5.2 Hz, 1H, OH), 3.36 (*td*, *J* = 6.6, 5.1 Hz, 2H, 16-H), 2.70 (*q*, *J* = 6.6 Hz, 2H, 9-H), 4.31–4.28 (*m*, 1H, 5-H), 1.83–1.75 (*m*, 4H, 6-H_a_, 6′-H_a_, 7-H_a_, 7′-H_a_), 1.74–1.67 (*m*, 1H, 8-H_a_), 1.47–1.28 (*m*, 8H, 6-H_b_, 6′-H_b_, 7-H_b_, 7′-H_b_, 10-H, 15-H), 1.27–1.10 (*m*, 9H, 8-H_b_, 11-H, 12-H, 13-H, 14-H) ppm; ^13^C NMR (126 MHz, DMSO-*d*_6_): δ = 152.0 (C-4), 138.2 (C-1), 127.3 (C-2), 126.5 (C-2), 60.7 (C-16), 43.5 (C-5), 42.5 (C-9), 33.5 (C-6), 32.5 (C-15), 28.9 (C-10), 28.8 (C-14), 28.6 (C-11), 26.2 (C-7), 25.9 (C-13), 25.4 (C-8), 25.4 (C-12) ppm; MS: *m*/*z* = 390.3 (100%, [M + Na]^+^); anal. calcd. for C_20_H_33_NSO_3_ (367.55): C 65.36, H 9.05, N 3.81; found: C 65.06, H 9.37, N 3.56.

#### 4.2.150. 8-[(4-Cyclohexylphenyl)sulfonamido]octyl Sulfamate (**74b**)

Applying GPB: from **74a** (200 mg, 0.54 mmol): **74b** (226 mg, 93%); white solid; R_f_ = 0.68 (CHCl_3_/EtOAc, 2:3); m.p. = 91–93 °C; UV–Vis: 229 nm (4.11); IR: ν = 3367*w*, 3299*w*, 3274*w*, 2953*w*, 2922*m*, 2851*m*, 1598*w*, 1544*w*, 1475*w*, 1463*w*, 1453*w*, 1409*m*, 1396*w*, 1372*s*, 1316*s*, 1183*m*, 1157*s*, 1149*s*, 1106*w*, 1093*m*, 1069*w*, 1057*m*, 1039*m*, 1016*w*, 1004*m*, 996*m*, 981*s*, 944*w*, 935*m*, 887*m*, 857*w*, 827*m*, 813*s*, 782*w*, 763*w*, 730*m*, 712*s*, 659*m*, 632*w*, 589*m*, 575*s*, 555*vs*, 528*s*, 507*m*, 481*w* cm^−1^; ^1^H NMR:δ = 7.71–7.66 (*m*, 2H, 2-H, 2′-H), 7.47–7.40 (*m*, 3H, 3-H, 3′-H, NH), 7.37 (*s*, 2H, NH_2_), 3.99 (*t*, *J* = 6.5 Hz, 2H, 16-H), 2.74–2.67 (*m*, 2H, 9-H), 2.64–2.55 (*m*, 1H, 5-H), 1.83–1.74 (*m*, 4H, 6-H_a_, 6′-H_a_, 7-H_a_, 7′-H_a_), 1.74–1.67 (*m*, 1H, 8-H_a_), 1.64–1.55 (*m*, 2H, 15-H), 1.49–1.12 (*m*, 15H, 6-H_b_, 6′-H_b_, 7-H_b_, 7′-H_b_, 8-H_b_, 10-H, 11-H, 12-H, 13-H, 14-H) ppm; ^13^C NMR:δ = 152.0 (C-1), 138.2 (C-4), 127.4 (C-2), 126.5 (C-3), 69.0 (C-16), 43.6 (C-5), 42.5 (C-9), 33.6 (C-6), 28.9 (C-15), 28.4 (C-10), 28.3 (C-13), 28.3 (C-12), 26.2 (C-7), 25.9 (C-11), 25.4 (C-8), 25.0 (C-14) ppm; MS: *m*/*z* = 469.9 (100%, [M + Na]^+^); anal. calcd. for C_20_H_34_N_2_S_2_O_5_ (446.62): C 53.79, H 7.67, N 6.27; found: C 53.35, H 7.96, N 5.98.

#### 4.2.151. 4-Cyclohexyl-N-(9-hydroxynonyl)benzene Sulfonamide (**75a**)

Applying GPA: from 4-cyclohexylbenzenesulfonyl chloride (500 mg, 1.93 mmol) and 9-amino-nonanol (462 mg, 2.90 mmol): **75a** (723 mg, 98%); white solid; R_f_ = 0.84 (CHCl_3_/EtOAc, 2:3); m.p. = 55–57 °C; UV–Vis: 229 nm (4.11); IR: ν = 3279*m*, 2922*s*, 2851*m*, 1599*w*, 1475*w*, 1467*w*, 1448*w*, 1426*m*, 1409*w*, 1318*s*, 1281*w*, 1269*w*, 1186*w*, 1157*vs*, 1095*m*, 1054*m*, 1036*m*, 1016*w*, 998*w*, 974*w*, 902*w*, 883*w*, 851*vw*, 822*m*, 780*w*, 733*m*, 706*s*, 654*m*, 632*m*, 599*s*, 569*vs*, 529*m*, 508*w*, 472*vw*, 460*vw* cm^−1^; ^1^H NMR:δ = 7.70–7.66 (*m*, 2H, 2-H, 2′-H), 7.46–7.40 (*m*, 3H, NH, 3-H, 3′-H), 4.30 (*td*, *J* = 5.1, 1.1 Hz, 1H, OH), 3.36 (*td*, *J* = 6.5, 5.1 Hz, 2H, 17-H), 2.71 (*q*, *J* = 6.7 Hz, 2H, 9-H), 2.63–2.55 (*m*, 1H, 5-H), 1.83–1.75 (*m*, 4H, 6-H_a_, 6′-H_a_, 7-H_a_, 7′-H_a_), 1.74–1.67 (*m*, 1H, 8-H_a_), 1.48–1.27 (*m*, 6H, 7-H_b_, 7′-H_b_, 8-H_b_, 10-H, 16-H), 1.26–1.11 (*m*, 11H, 6-H_b_, 6′-H_b_, 11-H, 12-H, 13-H, 14-H, 15-H) ppm; ^13^C NMR:δ = 152.0 (C-4), 138.2 (C-1), 127.3 (C-2), 126.5 (C-3), 60.7 (C-17), 43.6 (C-5), 42.5 (C-9), 33.6 (C-6), 32.5 (C-16), 28.9 (C-10, C-14), 28.8 (C-12), 28.5 (C-13), 26.2 (C-7), 26.0 (C-11), 25.5 (C-8), 25.4 (C-15) ppm; MS: *m*/*z* = 457.1 (100%, [M + Na]^+^); anal. calcd. for C_21_H_35_NSO_3_ (381.58): C 54.76, H 7.88, N 6.08; found: C 54.31, H 8.06, N 5.76. 

#### 4.2.152. 9-[(4-Cyclohexylphenyl)sulfonamido]nonyl Sulfamate (**75b**)

Applying GPB: from **75a** (200 mg, 0.52 mmol): **75b** (190 mg, 79%); white solid; R_f_ = 0.87 (CHCl_3_/EtOAc, 2:3); m.p. = 88–89 °C; UV–Vis: 230 nm (4.04); IR: ν = 3379*w*, 3278*m*, 2962*w*, 2924*m*, 2855*m*, 1600*w*, 1542*w*, 1477*w*, 1461*vw*, 1451*w*, 1424*m*, 1399*w*, 1376*s*, 1326*w*, 1310*s*, 1281*w*, 1214*vw*, 1186*s*, 1154*vs*, 1118*w*, 1094*m*, 1078*vw*, 1060*m*, 1034*m*, 993*w*, 967*s*, 907*s*, 885*m*, 866*w*, 819*vs*, 781*w*, 734*w*, 704*s*, 652*m*, 631*m*, 598*m*, 587*m*, 573*s*, 555*vs*, 534*m*, 524*m*, 505*m*, 483*m*, 454*vw* cm^−1^; ^1^H NMR:δ = 7.68–7.63 (*m*, 2H, 2-H, 2′-H), 7.44–7.38 (*m*, 3H, NH, 3-H, 3′-H), 7.34 (*s*, 2H, NH_2_), 3.97 (*t*, *J* = 6.5 Hz, 2H, 17-H), 2.68 (*q*, *J* = 6.7 Hz, 2H, 9-H), 2.61–2.52 (*m*, 1H, 5-H), 1.82–1.72 (*m*, 4H, 6-H_a_, 6′-H_a_, 7-H_a_, 7′-H_a_), 1.72–1.63 (*m*, 1H, 8-H_a_), 1.62–1.54 (*m*, 2H, 16-H), 1.46–1.08 (*m*, 17H, 6-H_b_, 6′-H_b_, 7-H_b_, 7′-H_b_, 8-H_b_, 10-H, 11-H, 12-H, 13-H, 14-H, 15-H) ppm; ^13^C NMR:δ = 152.0 (C-4), 138.2 (C-1), 127.4 (C-2), 126.5 (C-3), 69.0 (C-17), 43.6 (C-5), 42.5 (C-9), 33.6 (C-6), 28.9 (C-16), 28.7 (C-10), 28.4 (C-12), 28.4 (C-14), 28.3 (C-13), 26.2 (C-7), 25.9 (C-11), 25.4 (C-8), 25.0 (C-15) ppm; MS: *m*/*z* = 457.1 (100%, [M + Na]^+^); anal. calcd. for C_21_H_36_N_2_S_2_O_5_ (460.65): C 54.76, H 7.88, N 6.08; found: C 54.35, H 8.01, N 5.74. 

#### 4.2.153. 4-Cyclohexyl-N-(10-hydroxydecyl)benzene Sulfonamide (**76a**)

Applying GPA: from 4-cyclohexylbenzenesulfonyl chloride (450 mg, 1.74 mmol) and 10-amino-decanol (452 mg, 2.61 mmol): **76a** (625 mg, 91%); white solid; R_f_ = 0.82 (CHCl_3_/EtOAc, 2:3); m.p. = 71–72 °C; UV–Vis: 229 nm (4.17); IR: ν = 3494*w*, 3130*w*, 2915*s*, 2877*w*, 2849*s*, 1598*w*, 1476*w*, 1466*w*, 1448*m*, 1440*m*, 1408*w*, 1397*w*, 1346*w*, 1310*s*, 1288*w*, 1267*w*, 1188*w*, 1150*vs*, 1114*m*, 1095*m*, 1074*s*, 1043*w*, 1031*w*, 1020*m*, 999*w*, 973*w*, 914*m*, 876*w*, 865*vw*, 840*w*, 825*m*, 781*w*, 734*w*, 720*m*, 701*s*, 606*vs*, 575*s*, 523*m*, 505*w*, 468*m* cm^−1^; ^1^H NMR:δ = 7.70–7.66 (*m*, 2H, 2-H, 2′-H), 7.46–7.39 (*m*, 3H, NH, 3-H, 3′-H), 4.30 (*t*, *J* = 5.1 Hz, 1H, OH), 3.36 (*td*, *J* = 6.6, 5.2 Hz, 2H, 18-H), 2.74–2.67 (*m*, 2H, 9-H), 2.64–2.54 (*m*, 1H, 5-H), 1.84–1.75 (*m*, 4H, 6-H_a_, 6′-H_a_, 7-H_a_, 7′-H_a_), 1.74–1.67 (*m*, 1H, 8-H_a_), 1.47–1.27 (*m*, 8H, 6-H_b_, 6′-H_b_, 7-H_b_, 7′-H_b_, 10-H, 17-H), 1.27–1.09 (*m*, 13H, 8-H_b_, 11-H, 12-H, 13-H, 14-H, 15-H, 16-H) ppm;´^13^C NMR:δ = 152.0 (C-4), 138.2 (C-1), 127.3 (C-2), 126.6 (C-3), 60.7 (C-18), 43.6 (C-5), 42.5 (C-9), 33.6 (C-6), 32.5 (C-17), 29.0 (C-10), 28.9 (C-12), 28.9 (C-15), 28.8 (C-13), 28.5 (C-14), 26.2 (C-7), 26.0 (C-11), 25.5 (C-16), 25.4 (C-8) ppm; MS: *m*/*z* = 457.1 (100%, [M + Na]^+^); anal. calcd. for C_22_H_37_NSO_3_ (395.60): C 66.79, H 9.43, N 3.54; found: C 66.25, H 9.81, N 3.16. 

#### 4.2.154. 10-[(4-Cyclohexylphenyl)sulfonamido]decyl Sulfamate (**76b**)

Applying GPB: from **76a** (180 mg, 0.46 mmol): **76b** (161 mg, 75%); white solid; R_f_ = 0.9 (CHCl_3_/EtOAc, 2:3); m.p. = 100–101 °C; UV–Vis: 229 nm (4.33); IR: ν = 3369*w*, 3298*w*, 3275*w*, 2953*w*, 2919*m*, 2849*m*, 1598*w*, 1543*w*, 1475*w*, 1464*w*, 1454*w*, 1409*w*, 1396*w*, 1373*s*, 1317*s*, 1183*m*, 1157*s*, 1150*s*, 1093*m*, 1060*w*, 1052*m*, 1037*w*, 1021*m*, 1001*m*, 978*m*, 937*m*, 915*w*, 882*m*, 845*w*, 827*m*, 815*s*, 798*m*, 782*w*, 763*w*, 731*m*, 713*s*, 661*m*, 632*w*, 591*m*, 575*s*, 555*vs*, 530*m*, 504*w*, 476*w*, 471*w*, 417*w* cm^−1^; ^1^H NMR:δ = 7.71–7.66 (*m*, 2H, 2-H, 2′-H), 7.46–7.40 (*m*, 3H, NH, 3-H, 3′-H), 7.37 (*s*, 2H, NH_2_), 4.00 (*t*, *J* = 6.5 Hz, 2H, 18-H), 2.71 (*q*, *J* = 6.7 Hz, 2H, 9-H), 2.63–2.54 (*m*, 1H, 5-H), 1.83–1.75 (*m*, 4H, 6-H_a_, 6′-H_a_, 7-H_a_, 7′-H_a_), 1.74–1.67 (*m*, 1H, 8-H_a_), 1.61 (*p*, *J* = 6.5 Hz, 2H, 17-H), 1.48–1.37 (*m*, 3H, 8-H_b_, 10-H), 1.36–1.12 (*m*, 16H, 6-H_b_, 6′-H_b_, 7-H_b_, 7′-H_b_, 11-H, 12-H, 13-H, 14-H, 15-H, 16-H) ppm; ^13^C NMR:δ = 152.0 (C-4), 138.2 (C-1), 127.4 (C-2), 126.5 (C-3), 69.0 (C-18), 43.6 (C-5), 42.5 (C-9), 33.6 (C-6), 28.9 (C-10), 28.8 (C-13), 28.8 (C-17), 28.5 (C-12), 28.5 (C-15), 28.3 (C-14), 26.2 (C-7), 26.0 (C-11), 25.4 (C-8), 25.1 (C-16) ppm; MS: *m*/*z* = 457.1 (100%, [M + Na]^+^); anal. calcd. for C_22_H_38_N_2_S_2_O_5_ (474.68): C 55.67, H 8.07, N 5.90; found: C 55.25, H 8.41, N 5.65. 

#### 4.2.155. 4-(Adamantan-1-yl)-N-(8-hydroxyoctyl)benzene Sulfonamide (**77a**)

Applying GPA: from 4-(adamantan-1-yl)benzenesulfonyl chloride (300 mg, 0.96 mmol) and 8-amino-octanol (212 mg, 1.45 mmol): **77a** (363 mg, 90%); white solid; R_f_ = 0.70 (CHCl_3_/EtOAc, 2:3); m.p. = 101–102 °C; UV–Vis: 231 nm (4.15); IR: ν = 3463*w*, 3147*w*, 2917*s*, 2903*m*, 2849*m*, 1477*w*, 1465*w*, 1447*w*, 1399*w*, 1381*w*, 1367*w*, 1360*w*, 1345*w*, 1316*s*, 1304*m*, 1291*m*, 1158*s*, 1095*m*, 1085*m*, 1058*m*, 1029*m*, 1014*w*, 975*w*, 957*w*, 951*w*, 939*m*, 846*w*, 832*w*, 806*m*, 774*w*, 742*m*, 721*w*, 683*m*, 674*m*, 599*s*, 579*vs*, 534*m*, 483*m* cm^−1^; ^1^H NMR:δ = 7.73–7.69 (*m*, 2H, 2-H, 2′-H), 7.58–7.54 (*m*, 2H, 3-H, 3′-H), 7.44 (*t*, *J* = 5.8 Hz, 1H, NH), 4.29 (*td*, *J* = 5.1, 1.0 Hz, 1H, OH), 3.35 (*td*, *J* = 6.6, 5.2 Hz, 2H, 22-H), 2.74–2.66 (*m*, 2H, 15-H), 2.10–2.03 (*m*, 3H, 7-H, 9-H, 11-H), 1.88 (*d*, *J* = 3.0 Hz, 6H, 6-H, 12-H, 13-H), 1.79–1.69 (*m*, 6H, 8-H, 10-H, 14-H), 1.42–1.27 (*m*, 4H, 16-H, 21-H), 1.26–1.10 (*m*, 8H, 17-H, 18-H, 19-H, 20-H) ppm; ^13^C NMR:δ = 155.2 (C-4), 137.8 (C-1), 126.4 (C-2), 125.5 (C-3), 60.7 (C-22), 42.5 (C-15), 42.2 (C-12, C-6, C-13), 36.2 (C-5), 36.0 (C-10, C-8, C-14), 32.5 (C-21), 28.9 (C-16), 28.8 (C-18), 28.6 (C-19), 28.2 (C-7, C-9, C-11), 25.9 (C-17), 25.4 (C-20) ppm; MS: *m*/*z* = 457.1 (100%, [M + Na]^+^); anal. calcd. for C_24_H_37_NSO_3_ (419.62): C 68.70, H 8.89, N 3.34; found: C 68.47, H 9.19, N 2.96. 

#### 4.2.156. 8-[(4-(Adamantan-1-yl)phenyl)sulfonamido]octyl Sulfamate (**77b**)

Applying GPB: from **77a** (190 mg, 0.42 mmol): **77b** (147 mg, 66%); white solid; R_f_ = 0.87 (CHCl_3_/EtOAc, 2:3); m.p. = 85–86 °C; UV–Vis: 231 nm (4.20); IR: ν = 3360*w*, 3254*m*, 2929*m*, 2899*s*, 2847*m*, 1568*w*, 1448*m*, 1398*w*, 1361*m*, 1344*w*, 1316*vs*, 1293*m*, 1183*m*, 1145*vs*, 1111*w*, 1092*s*, 1076*m*, 1065*m*, 1044*w*, 1031*w*, 1013*m*, 999*w*, 982*w*, 976*w*, 964*m*, 953*m*, 933*s*, 906*m*, 898*m*, 877*m*, 861*w*, 837*w*, 795*s*, 773*w*, 756*m*, 744*s*, 730*w*, 721*m*, 694*s*, 665*w*, 633*w*, 595*s*, 575*vs*, 551*vs*, 537*m*, 526*m*, 505*w*, 480*m*, 473*m* cm^−1^; ^1^H NMR:δ = 7.73–7.68 (*m*, 2H, 2-H, 2′-H), 7.59–7.54 (*m*, 2H, 3-H, 3′-H), 7.45 (*t*, *J* = 5.9 Hz, 1H, NH), 7.37 (*s*, 2H, NH_2_), 3.99 (*t*, *J* = 6.5 Hz, 2H, 22-H), 2.71 (*q*, *J* = 6.7 Hz, 2H, 15-H), 2.07 (*p*, *J* = 3.1 Hz, 3H, 7-H, 9-H, 11-H), 1.88 (*d*, *J* = 2.9 Hz, 6H, 6-H, 12-H, 13-H), 1.79–1.69 (*m*, 6H, 8-H, 10-H, 14-H), 1.64–1.55 (*m*, 2H, 21-H), 1.37–1.25 (*m*, 2H, 16-H), 1.23–1.13 (*m*, 8H, 17-H, 18-H, 19-H, 20-H) ppm; ^13^C NMR:δ = 155.2 (C-4), 137.8 (C-1), 126.4 (C-2), 125.5 (C-3), 69.0 (C-22), 42.5 (C-15), 42.2 (C-6, C-12, C-13), 36.2 (C-5), 36.0 (C-8, C-10, C-14), 28.9 (C-21), 28.4 (C-19), 28.3 (C-16), 28.3 (C-18), 28.2 (C-7, C-9, C-11), 25.9 (C-17), 25.0 (C-20) ppm; MS: *m*/*z * = 457.1 (100%, [M + Na]^+^); anal. calcd. for C_24_H_38_N_2_S_2_O_5_ (498.70): C 57.80, H 7.68, N 5.62; found: C 57.64, H 7.43, N 5.45. 

#### 4.2.157. 4-(Adamantan-1-yl)-N-(9-hydroxynonyl)benzene Sulfonamide (**78a**)

Applying GPA: from 4-(adamantan-1-yl)benzenesulfonyl chloride (150 mg, 0.48 mmol) and 9-amino-nonanol (115 mg, 0.72 mmol): **78a** (201 mg, 96%); white solid; R_f_ = 0.72 (CHCl_3_/EtOAc, 2:3); m.p. = 84–85 °C; UV–Vis: 231 nm (4.28); IR: ν = 3503*vw*, 3281*w*, 2903*s*, 2849*m*, 1597*w*, 1496*vw*, 1449*m*, 1400*w*, 1319*s*, 1293*m*, 1157*vs*, 1094*m*, 1031*m*, 1014*w*, 976*w*, 839*w*, 806*m*, 752*m*, 741*m*, 674*s*, 596*vs*, 576*s*, 475*w* cm^−1^; ^1^H NMR:δ = 7.71 (*d*, *J* = 8.6 Hz, 2H, 2-H, 2′-H), 7.56 (*d*, *J* = 8.6 Hz, 2H, 3-H, 3′-H), 7.44 (*t*, *J* = 5.8 Hz, 1H, NH), 4.29 (*t*, *J* = 5.1 Hz, 1H, OH), 3.40–3.33 (*m*, 2H, 23-H), 2.71 (*q*, *J* = 6.8 Hz, 2H, 15-H), 2.07 (*s*, 3H, 7-H, 9-H, 11-H), 1.88 (*d*, *J* = 2.7 Hz, 6H, 6-H, 12-H, 13-H), 1.79–1.69 (*m*, 6H, 8-H, 10-H, 14-H), 1.43–1.35 (*m*, 2H, 22-H), 1.34–1.27 (*m*, 2H, 16-H), 1.27–1.10 (*m*, 10H, 17-H, 18-H, 19-H, 20-H, 21-H) ppm; ^13^C NMR:δ = 155.2 (C-4), 137.9 (C-1), 126.4 (C-2), 125.5 (C-3), 60.7 (C-23), 42.5 (C-15), 42.2 (C-6, C-12, C-13), 36.2 (C-5), 36.0 (C-8, C-10, C-14), 32.5 (C-22), 28.9 (C-16, C-18), 28.9 (C-20), 28.5 (C-19), 28.2 (C-7, C-9, C-11), 26.0 (C-17), 25.5 (C-21) ppm; MS: *m*/*z* = 457.1 (100%, [M + Na]^+^); anal. calcd. for C_25_H_39_NSO_3_ (433.65): C 69.24, H 9.07, N 3.23; found: C 68.97, H 9.33, N 2.95. 

#### 4.2.158. 9-[(4-(Adamantan-1-yl)phenyl)sulfonamido]nonyl Sulfamate (**78b**)

Applying GPB: from **78a** (110 mg, 0.25 mmol): **78b** (110 mg, 84%); white solid; R_f_ = 0.92 (CHCl_3_/EtOAc, 2:3); m.p. = 94–96 °C; UV–Vis: 231 nm (4.19); IR: ν = 3347*w*, 3269*w*, 2922*m*, 2903*m*, 2850*m*, 1429*w*, 1401*w*, 1370*m*, 1317*s*, 1297*m*, 1176*m*, 1156*vs*, 1117*w*, 1103*w*, 1093*w*, 1076*w*, 1059*w*, 1033*w*, 1013*w*, 963*m*, 926*m*, 879*w*, 848*w*, 839*w*, 823*m*, 805*m*, 775*w*, 747*m*, 729*w*, 697*m*, 648*w*, 598*s*, 572*s*, 564*s*, 534*w*, 522*w*, 513*w*, 484*w* cm^−1^; ^1^H NMR:δ = 7.71 (*d*, *J* = 8.2 Hz, 2H, 2-H, 2′-H), 7.57 (*d*, *J* = 8.2 Hz, 2H, 3-H, 3′-H), 7.44 (*t*, *J* = 5.9 Hz, 1H, NH), 7.37 (*s*, 2H, NH_2_), 3.99 (*t*, *J* = 6.5 Hz, 2H, 23-H), 2.71 (*q*, *J* = 6.7 Hz, 2H, 15-H), 2.07 (*s*, 3H, 7-H, 9-H, 11-H), 1.88 (*d*, *J* = 2.9 Hz, 6H, 6-H, 12-H, 13-H), 1.74 (*s*, 6H, 8-H, 10-H, 14-H), 1.60 (*p*, *J* = 6.7 Hz, 2H, 22-H), 1.37–1.27 (*m*, 4H, 16-H, 21-H), 1.27–1.11 (*m*, 8H, 17-H, 18-H, 19-H, 20-H) ppm; ^13^C NMR:δ = 155.7 (C-4), 138.3 (C-1), 126.8 (C-2), 126.0 (C-3), 69.4 (C-23), 42.9 (C-15), 42.7 (C-6, C-12, C-13), 36.6 (C-5), 36.5 (C-8, C-10, C-14), 29.4 (C-22), 29.2 (C-16), 28.9 (C-18), 28.9 (C-20), 28.8 (C-19), 26.4 (C-17), 25.5 (C-21) ppm; MS: *m*/*z* = 457.1 (100%, [M + Na]^+^); anal. calcd. for C_25_H_40_N_2_S_2_O_5_ (512.72): C 58.56, H 7.86, N 5.46; found: C 58.27, H 8.05, N 5.16. 

#### 4.2.159. 4-(Adamantan-1-yl)-N-(10-hydroxydecyl)benzene Sulfonamide (**79a**)

Applying GPA: from 4-(adamantan-1-yl)benzenesulfonyl chloride (300 mg, 0.97 mmol) and 10-amino-decanol (251 mg, 1.45 mmol): **79a** (377 mg, 87%); white solid; R_f_ = 0.74 (CHCl_3_/EtOAc, 2:3); m.p. = 76–79 °C; UV–Vis: 231 nm (4.20); IR: ν = 3496*vw*, 3282*w*, 2903*s*, 2849*s*, 1597*w*, 1449*m*, 1400*w*, 1369*w*, 1320*s*, 1293*m*, 1157*vs*, 1094*m*, 1056*m*, 1031*m*, 1014*w*, 976*w*, 881*vw*, 839*w*, 806*m*, 740*m*, 721*w*, 674*s*, 596*vs*, 577*s*, 495*w*, 487*w*, 476*w*, 451*vw* cm^−1^; ^1^H NMR:δ = 7.73–7.68 (*m*, 2H, 2-H, 2′-H), 7.59–7.53 (*m*, 2H, 3-H, 3′-H), 7.44 (*t*, *J* = 5.8 Hz, 1H, NH), 4.30 (*t*, *J* = 5.1 Hz, 1H, OH), 3.36 (*td*, *J* = 6.5, 5.2 Hz, 2H, 24-H), 2.71 (*q*, *J* = 6.6 Hz, 2H, 15-H), 2.09–2.03 (*m*, 3H, 7-H, 9-H, 11-H), 1.88 (*d*, *J* = 3.0 Hz, 6H, 6-H, 12-H, 13-H), 1.73 (*d*, *J* = 4.4 Hz, 6H, 8-H, 10-H, 14-H), 1.43–1.34 (*m*, 2H, 23-H), 1.34–1.26 (*m*, 2H, 16-H), 1.26–1.09 (*m*, 12H, 17-H, 18-H, 19-H, 20-H, 21-H, 22-H) ppm; ^13^C NMR:δ = 155.2 (C-4), 137.9 (C-1), 126.4 (C-2), 125.5 (C-3), 60.7 (C-24), 42.5 (C-15), 42.2 (C-6, C-12, C-13), 36.2 (C-5), 36.0 (C-8, C-10, C-14), 32.5 (C-23), 29.0 (C-16), 28.9 (C-19), 28.9 (C-21), 28.8 (C-20), 28.5 (C-18), 28.2 (C-7, C-9, C-11), 26.0 (C-17), 25.5 (C-22) ppm; MS: *m*/*z* = 457.1 (100%, [M + Na]^+^); anal. calcd. for C_26_H_41_NSO_3_ (447.68): C 69.76, H 9.23, N 3.13; found: C 69.41, H 9.55, N 2.94. 

#### 4.2.160. 10-[(4-(Adamantan-1-yl)phenyl)sulfonamido]decyl Sulfamate (**79b**)

Applying GPB: from **79a** (170 mg, 0.38 mmol): **79b** (92 mg, 46%); white solid; R_f_ = 0.85 (CHCl_3_/EtOAc, 2:3); m.p. = 87–89 °C; UV–Vis: 231 nm (4.18); IR: ν = 3291*w*, 2919*m*, 2903*m*, 2850*m*, 1597*vw*, 1567*vw*, 1563*vw*, 1471*w*, 1450*w*, 1426*w*, 1400*w*, 1366*m*, 1322*s*, 1294*m*, 1179*m*, 1158*vs*, 1119*w*, 1103*w*, 1093*m*, 1064*w*, 1051*w*, 1031*w*, 1014*w*, 954*m*, 936*m*, 880*w*, 831*m*, 805*m*, 775*w*, 762*w*, 747*m*, 731*w*, 722*w*, 682*m*, 628*w*, 597*s*, 572*s*, 561*s*, 523*w*, 474*w* cm^−1^; ^1^H NMR:δ = 7.74–7.69 (*m*, 2H, 2-H, 2′-H), 7.59–7.54 (*m*, 2H, 3-H, 3′-H), 7.44 (*t*, *J* = 5.8 Hz, 1H, NH), 7.37 (*s*, 2H, NH_2_), 4.00 (*t*, *J* = 6.5 Hz, 2H, 24-H), 2.71 (*q*, *J* = 6.6 Hz, 2H, 15-H), 2.07 (*p*, *J* = 3.1 Hz, 3H, 7-H, 9-H, 11-H), 1.88 (*d*, *J* = 2.9 Hz, 6H, 6-H, 12-H, 13-H), 1.80–1.68 (*m*, 6H, 8-H, 10-H, 14-H), 1.61 (*p*, *J* = 6.6 Hz, 2H, 23-H), 1.36–1.27 (*m*, 4H, 16-H, 22-H), 1.27–1.11 (*m*,10H, 17-H, 18-H, 19-H, 20-H, 21-H) ppm; ^13^C NMR:δ = 155.6 (C-4), 138.3 (C-1), 126.8 (C-2), 126.0 (C-3), 69.4 (C-24), 42.9 (C-15), 42.7 (C-6, C-12, C-13), 36.6 (C-5), 36.5 (C-8, C-10, C-14), 29.4 (C-16), 29.3 (C-19), 29.3 (C-23), 29.0 (C-18), 28.9 (C-21), 28.8 (C-20), 28.6 (C-7, C-9, C-11), 26.4 (C-17), 25.5 (C-22) ppm; MS: *m*/*z* = 457.1 (100%, [M + Na]^+^); anal. calcd. for C_26_H_42_N_2_S_2_O_5_ (526.75): C 59.29, H 8.04, N 5.32; found: C 58.97, H 8.31, N 5.03. 

### 4.3. Molecular Modeling

For the molecular docking studies, MOE 2020 software (2020 0901) was employed. The enzyme structure, obtained from the PDB Database (PDB ID: 3HS4), was prepared using the QuickPrep tool (version 2020). Ligands were deprotonated to align with the crystal structure of the co-crystallized ligand. Docking utilized a pharmacophore model focusing on the interaction between the sulfonamide group and the zinc ion. Ligands were initially positioned with the Triangle Matcher algorithm, producing 30 poses, which were scored with the London dG scoring function. The top five poses underwent refinement with a rigid receptor model and were rescored using the GBVI/WSA dG method. The best pose was assessed by comparing it to the expected logical structure and interactions. The docking protocol was validated by successfully redocking the co-crystallized ligand, acetazolamide.

### 4.4. Enzymatic Assay

The enzymatic assays have been performed with carbonic anhydrase II (*b*CAII, ≥3000 W-A units/mg from bovine erythrocytes) from Sigma (Taufkirchen, Germany) using a BMG Labtech Spectrostar Omega apparatus (BMG Labtech, Ortenberg, Germany) measuring λ = 415 nm. Conditions have been described previously [72]. K_i_ and K_i_′ values were determined from Lineweaver–Burk, Dixon and Cornish Bowden plots, respectively. 

## Figures and Tables

**Table 1:** Inhibition (I in %) of *b*CA II by compounds **1b**–**79b** (at 1 μM concentration of the inhibitor); acetazolamide (**AAZ**) was used as a positive standard. All experiments were performed in triplicate with three technical replicas.

Cmp.	Inhibition [%]	Cmp.	Inhibition [%]	Cmp.	Inhibition [%]
**AAZ**	99.2 ± 0.2	27	50.0 ± 0.8	54	28.4 ± 0.4
**1**	59.2 ± 0.8	28	37.9 ± 0.7	55	21.3 ± 1
**2**	39.9 ± 0.4	29	57.2 ± 0.7	56	10.0 ± 0.6
**3**	32.2 ± 0.3	30	68.1 ± 0.3	57	20.6 ± 0.6
**4**	51.8 ± 0.7	31	55.6 ± 0.7	58	54.0 ± 0.9
**5**	56.0 ± 0.5	32	56.2 ± 0.8	59	53.9 ± 0.3
**6**	56.0 ± 0.8	33	63.2 ± 0.4	60	43.3 ± 0.6
**7**	60.2 ± 0.7	34	60.3 ± 0.9	61	39.2 ± 0.1
**8**	55.4 ± 0.7	35	43.7 ± 0.4	62	41.7 ± 0.8
**9**	10.7 ± 0.4	36	40.8 ± 0.6	63	33.3 ± 0.3
**10**	7.2 ± 0.9	37	45.9 ± 0.7	64	16.0 ± 0.4
**11**	5.9 ± 0.9	38	30.3 ± 0.1	65	56.2 ± 0.8
**12**	75.8 ± 0.6	39	28.1 ± 0.5	66	34.5 ± 0.4
**13**	63.8 ± 0.8	40	63.7 ± 0.1	67	27.5 ± 0.3
**14**	42.9 ± 0.1	41	50.1 ± 0.9	68	52.1 ± 1.2
**15**	58.9 ± 0.2	42	35.3 ± 0.5	69	43.1 ± 1.5
**16**	40.3 ± 0.7	43	44.4 ± 0.3	70	34.7 ± 2.5
**17**	42.8 ± 0.5	44	45.5 ± 0.7	71	18.6 ± 1.0
**18**	54.7 ± 0.6	45	37 ± 0.3	72	8.6 ± 0.6
**19**	59.5 ± 0.1	46	29.5 ± 0.9	73	14.8 ± 0.4
**20**	48.4 ± 0.1	47	38.4 ± 0.2	74	14.2 ± 1.1
**21**	32.2 ± 0.6	48	34.2 ± 0.7	75	32.1 ± 0.8
**22**	49.1 ± 0.2	49	75.6 ± 0.4	76	19.7 ± 1.6
**23**	51.8 ± 0.7	50	63.4 ± 0.6	77	32.6 ± 0.4
**24**	27.1 ± 0.9	51	44.9 ± 0.4	78	36.1 ± 1.8
**25**	38.8 ± 0.1	52	35.9 ± 0.4	79	40.1 ± 0.8
**26**	69.1 ± 0.7	53	29.1 ± 0.9		

**Table 2:** Inhibition constants (K_i_) for the most active compounds **12b**, **26b**, **30b**, **33b**, **40b**, and **49b** and CA II.

Cmp	12b	26b	30b	33b	40b	49b
K_i_ [μM]	0.76 ± 0.03	1.1 ± 0.03	1.58 ± 0.02	0.84 ± 0.02	1.64 ± 0.03	0.67 ± 0.05

## Data Availability

The data presented in this study are available on request from the corresponding author.

## Supplementary Materials

The following supporting information can be downloaded at: https://www.mdpi.com/article/10.3390/molecules29133015/s1, page 1 to page 53: ^1^H and ^13^C NMR spectra of all compounds; page 54 to page 58: representative HRMS spectra; page 59 to page 64: 2D and 3D depiction (docking calculations).
