# Supplementary material for: Synthesis and Enzymatic Evaluation of a Small Library of Substituted Phenylsulfonamido-Alkyl Sulfamates towards Carbonic Anhydrase II
Source: Molecules. 2024 Jun 25;29(13):3015. doi: 10.3390/molecules29133015 (PMC11243685; doi:10.3390/molecules29133015)

Supplementary material

Synthesis and Enzymatic Evaluation of a Small Library of Substituted Phenylsulfonamido-alkyl Sulfamates towards Carbonic Anhydrase II

Toni C. Denner [1], Niels V. Heise [1], Ahmed Al-Harrasi [2] and René Csuk [1,*]

Correspondence: rene.csuk@chemie.uni-halle.de (R.C.); Tel.: +49-345-55-25660

Compound 1a

^{1}H NMR (400 MHz, DMSO-d6); ^{13}C APT-NMR (101 MHz, DMSO-d6); APT...attached proton test

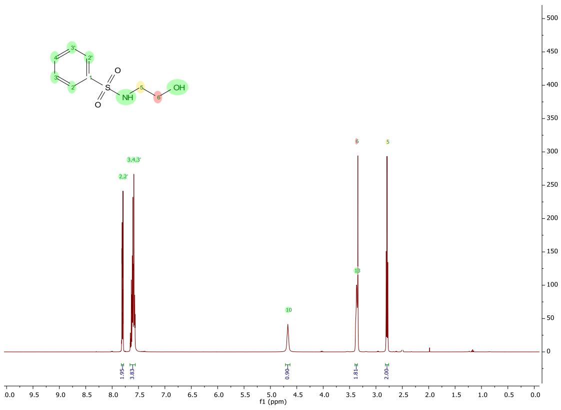

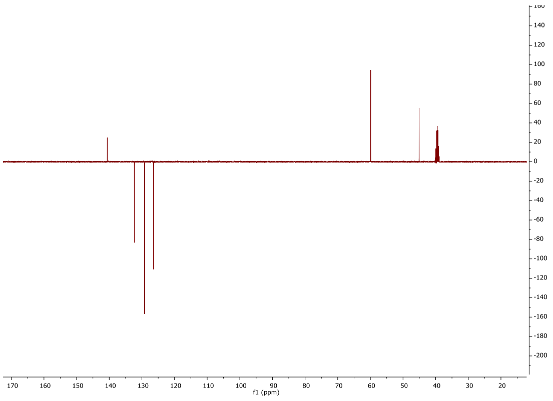

Compound 1b

^{1}H NMR (400 MHz, DMSO-d6); ^{13}C NMR (126 MHz, DMSO-d6)

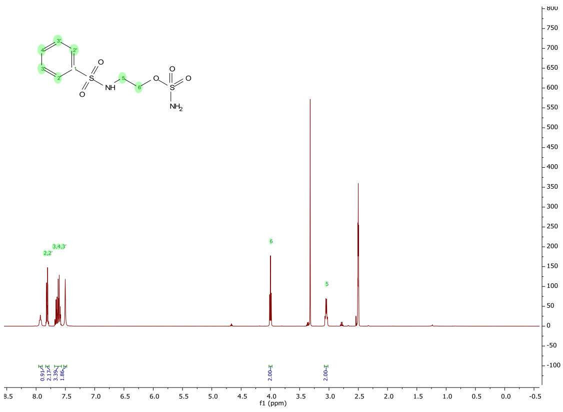

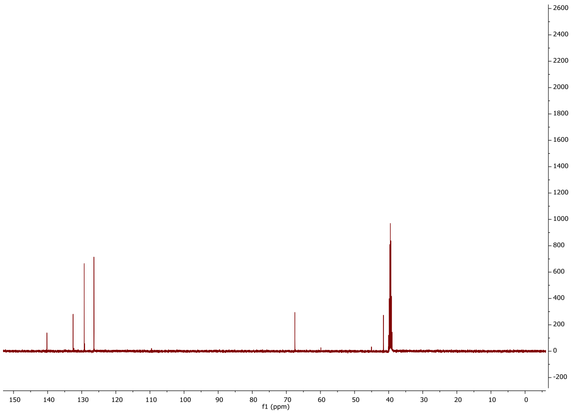

Compound 2a

^{1}H NMR (400 MHz, DMSO-d6); ^{13}C NMR (101 MHz, DMSO-d6)

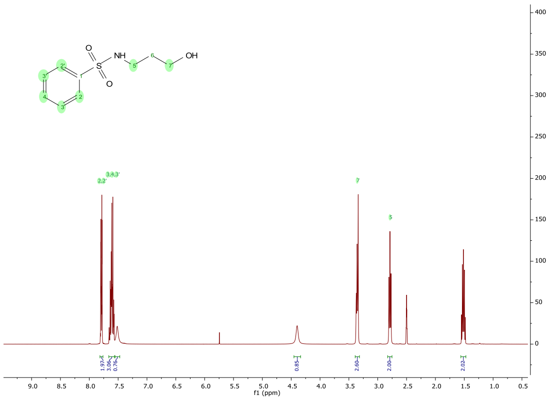

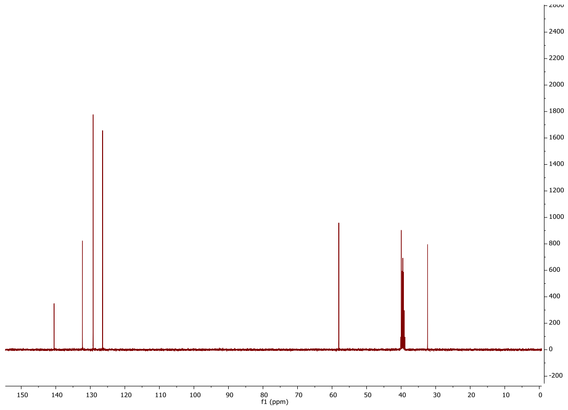

Compound 2b

^{1}H NMR (400 MHz, DMSO-d6); ^{13}C NMR (126 MHz, DMSO-d6)

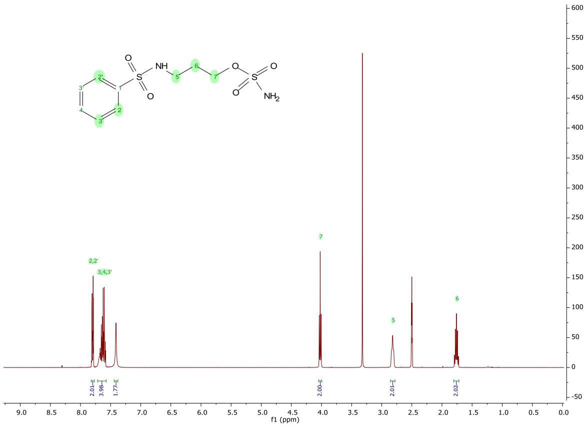

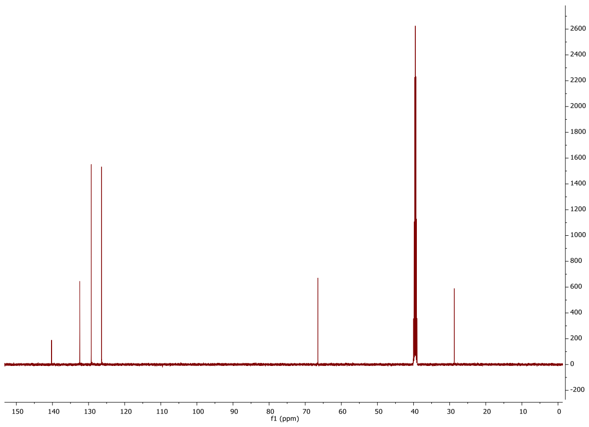

Compound 3a

^{1}H NMR (500 MHz, DMSO-d6); ^{13}C NMR (126 MHz, DMSO-d6)

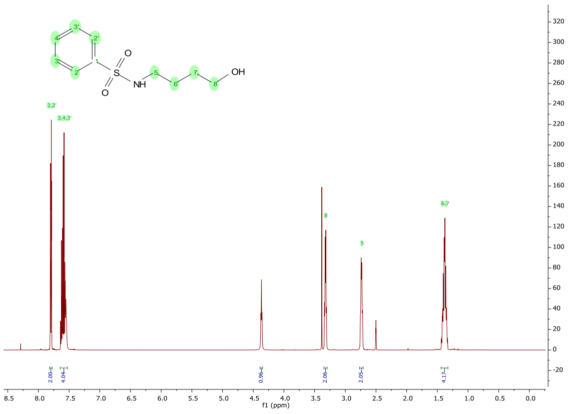

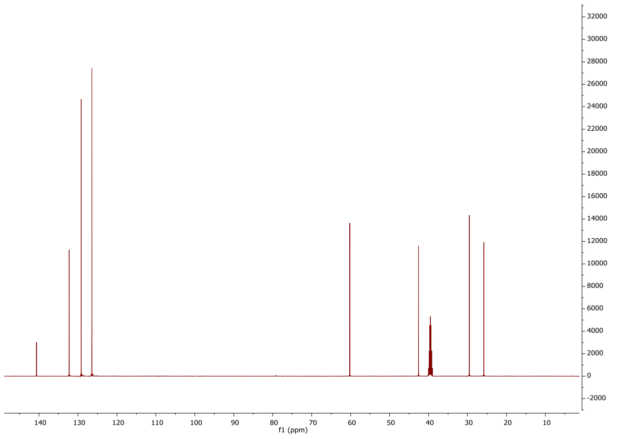

Compound 3b

^{1}H NMR (400 MHz, DMSO-d6); ^{13}C NMR (101 MHz, DMSO-d6)

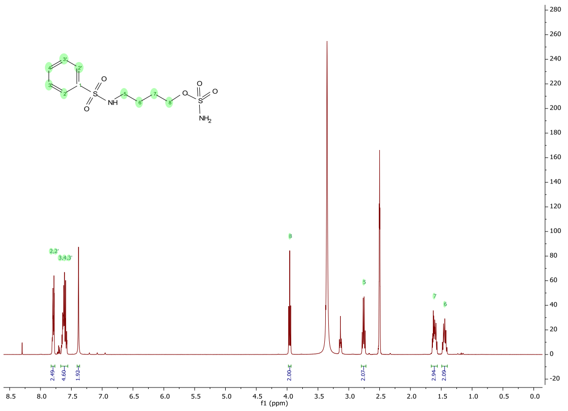

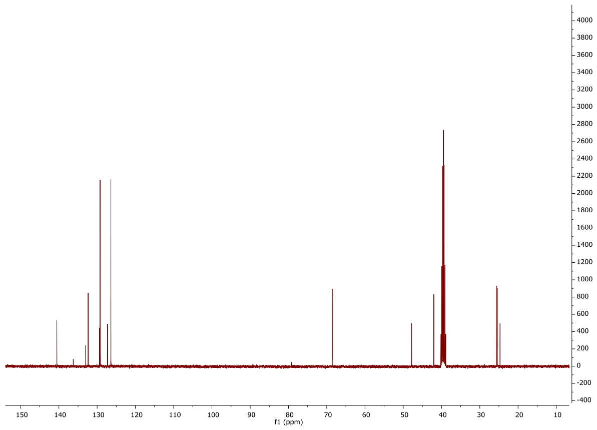

Compound 4a

^{1}H NMR (400 MHz, DMSO-d6); ^{13}C APT-NMR (101 MHz, DMSO-d6)

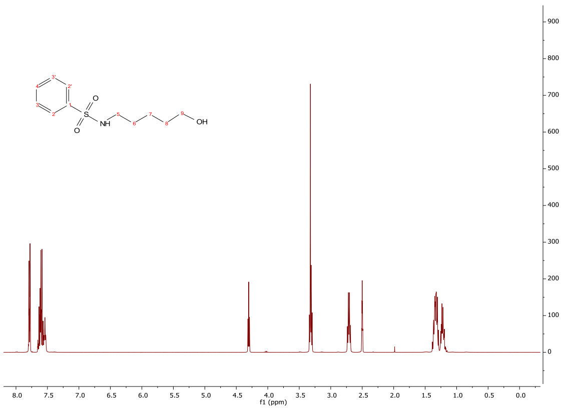

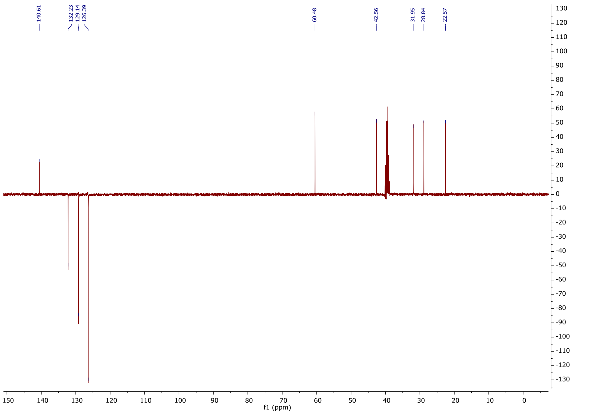

Compound 4b

^{1}H NMR (400 MHz, DMSO-d6); ^{13}C NMR (101 MHz, DMSO-d6)

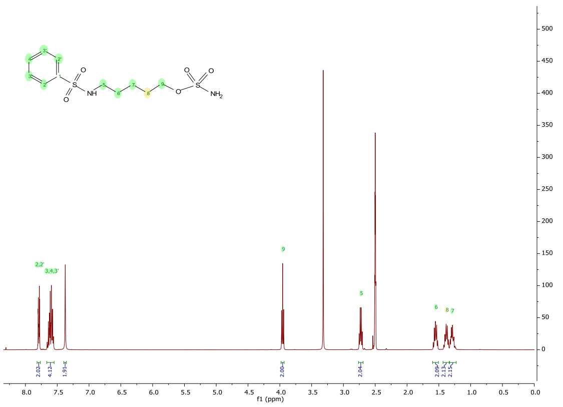

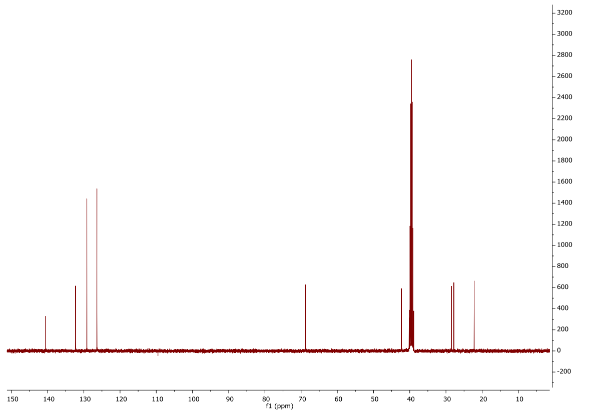

Compound 5a

^{1}H NMR (500 MHz, DMSO-d6); ^{13}C APT-NMR (126 MHz, DMSO-d6)

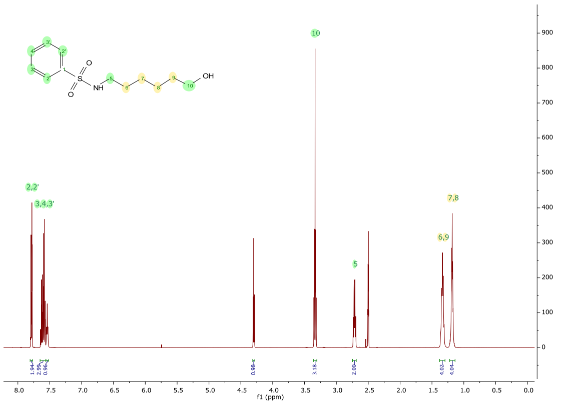

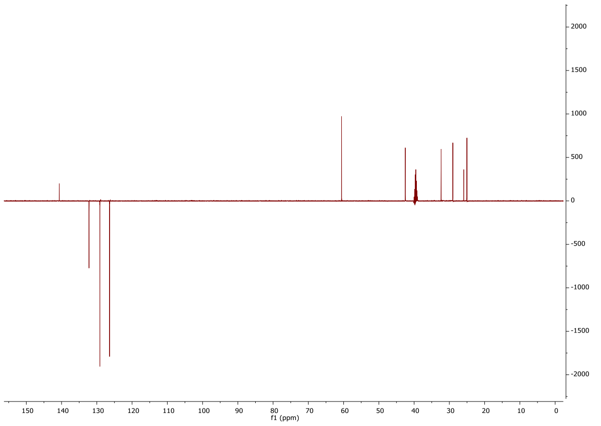

Compound 5b

^{1}H NMR (400 MHz, DMSO-d6); ^{13}C NMR (126 MHz, DMSO-d6)

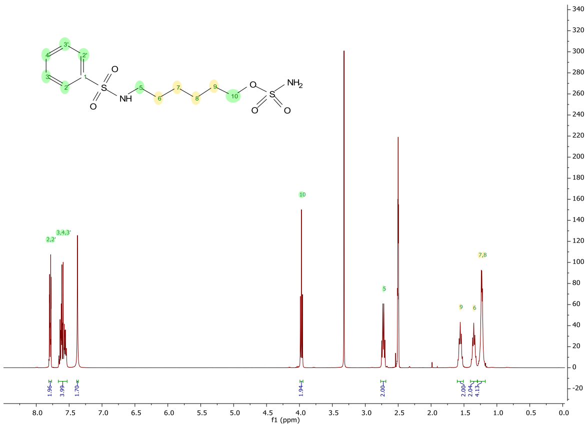

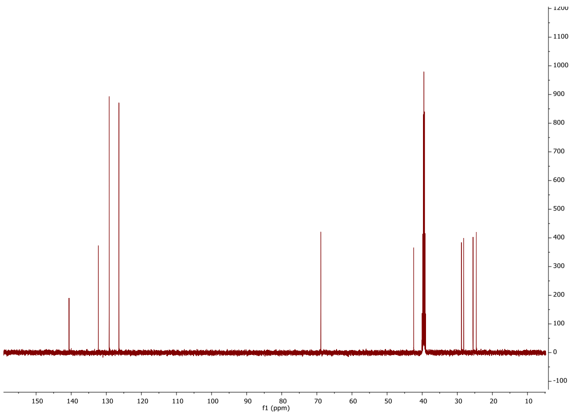

Compound 6a

^{1}H NMR (500 MHz, DMSO-d6); ^{13}C NMR (101 MHz, DMSO-d6)

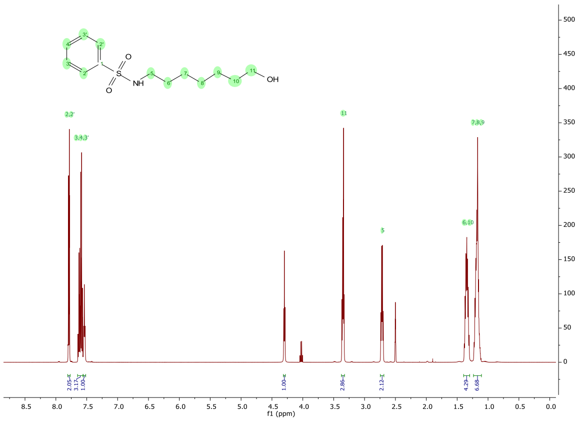

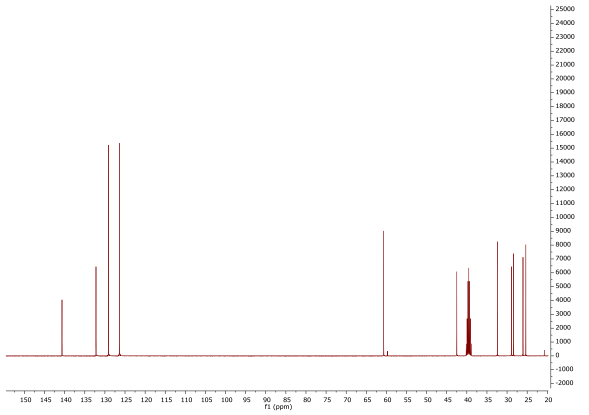

Compound 6b

^{1}H NMR (400 MHz, DMSO-d6); ^{13}C NMR (101 MHz, DMSO-d6)

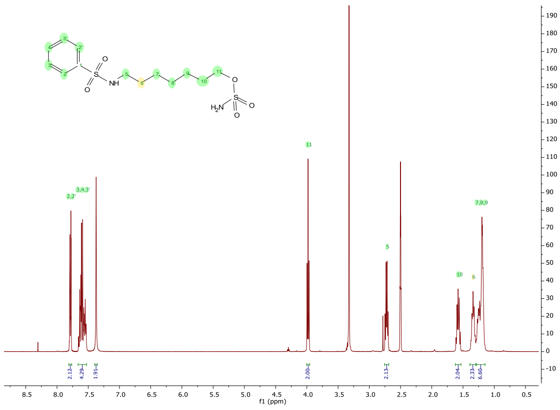

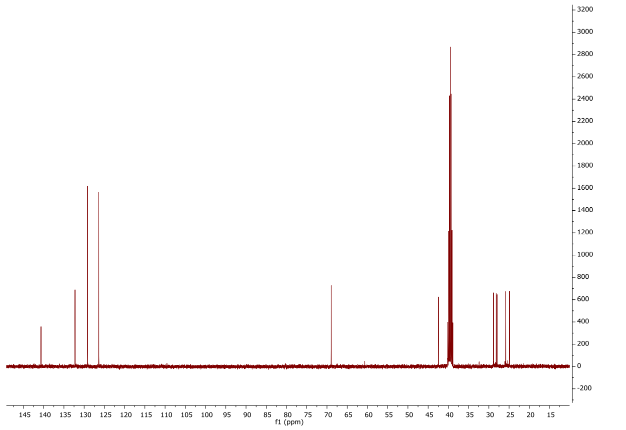

Compound 7a

^{1}H NMR (400 MHz, DMSO-d6); ^{13}C NMR (126 MHz, DMSO-d6)

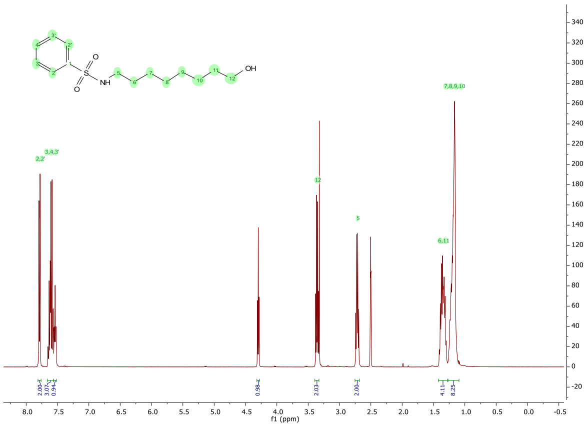

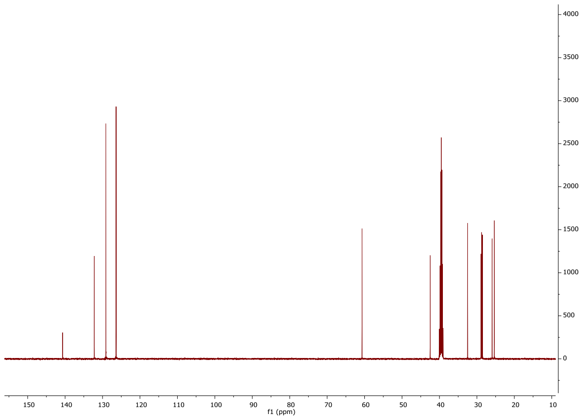

Compound 7b

^{1}H NMR (400 MHz, DMSO-d6); ^{13}C NMR (101 MHz, DMSO-d6)

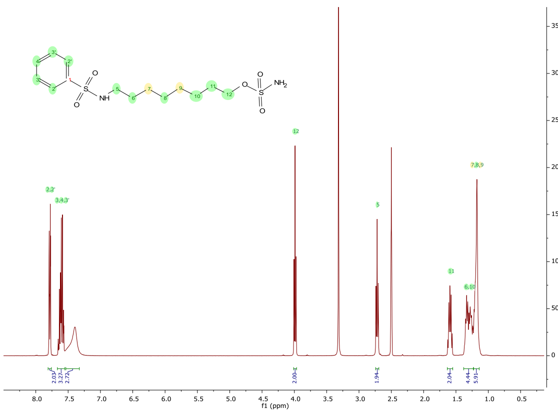

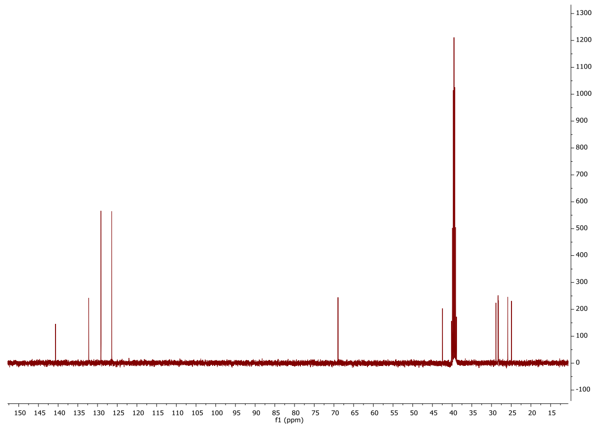

Compound 8a

^{1}H NMR (400 MHz, DMSO-d6); ^{13}C NMR (101 MHz, DMSO-d6)

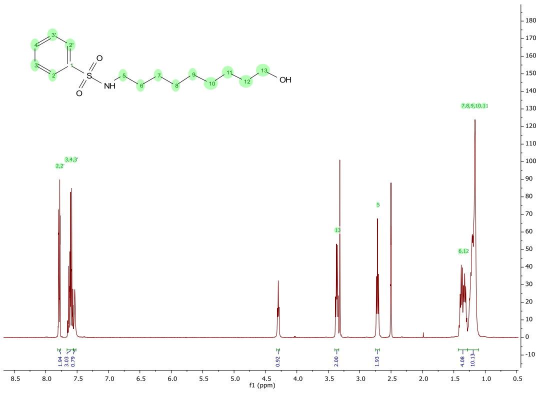

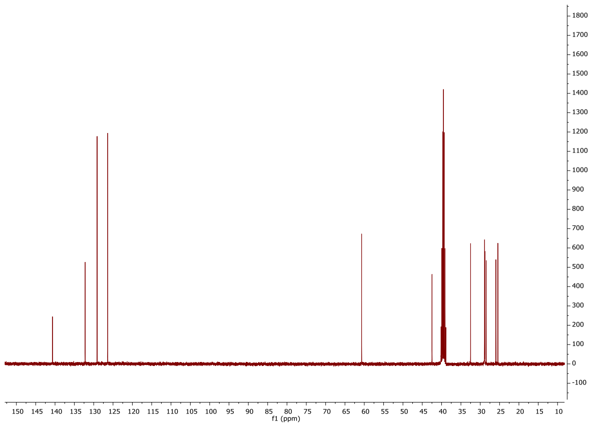

Compound 8b

^{1}H NMR (400 MHz, DMSO-d6); ^{13}C NMR (101 MHz, DMSO-d6)

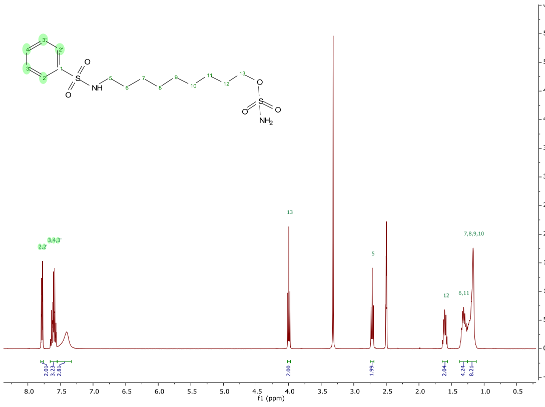

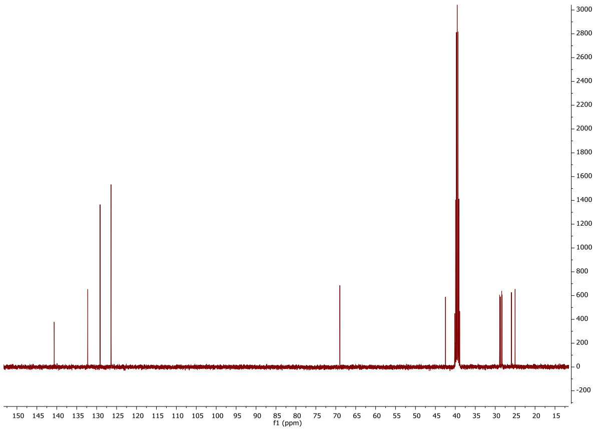

Compound 9a

^{1}H NMR (400 MHz, DMSO-d6); ^{13}C NMR (126 MHz, DMSO-d6)

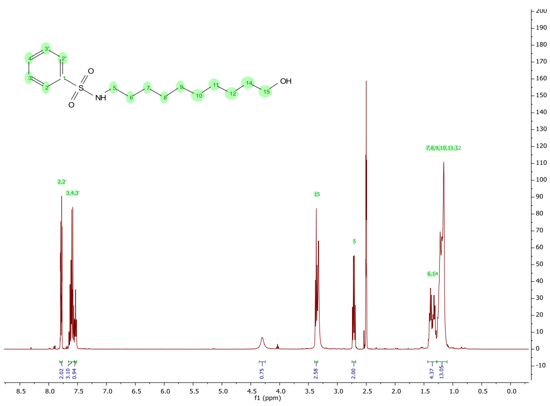

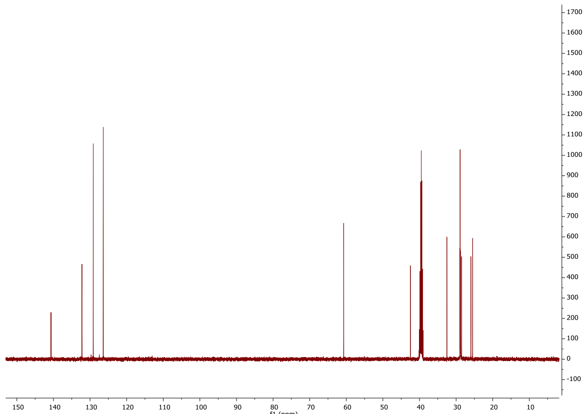

Compound 9b

^{1}H NMR (400 MHz, DMSO-d6); ^{13}C NMR (101 MHz, DMSO-d6)

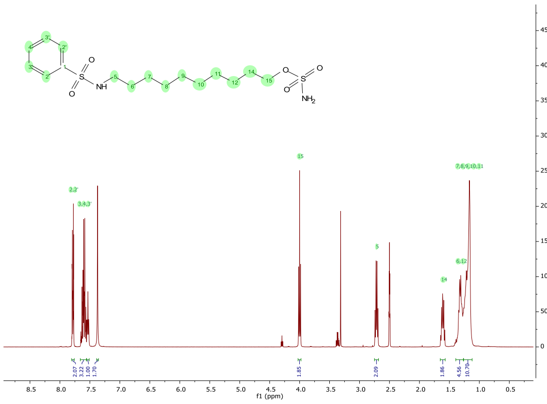

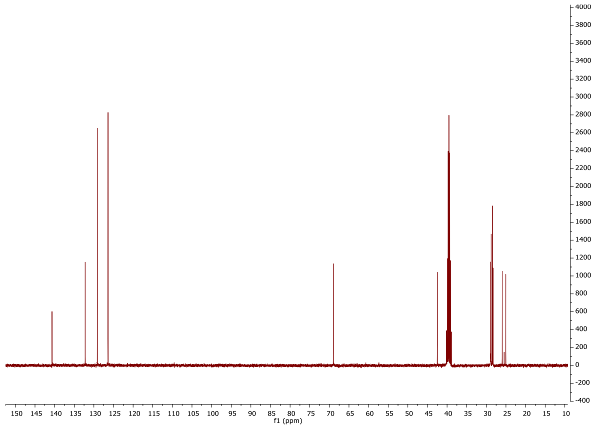

Compound 10a

^{1}H NMR (400 MHz, DMSO-d6); ^{13}C NMR (126 MHz, DMSO-d6)

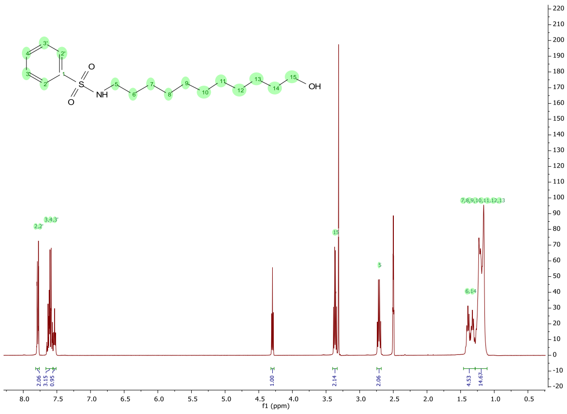

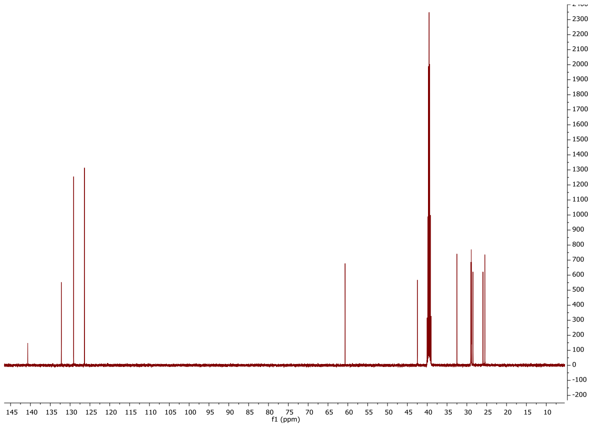

Compound 10b

^{1}H NMR (400 MHz, DMSO-d6); ^{13}C NMR (101 MHz, DMSO-d6)

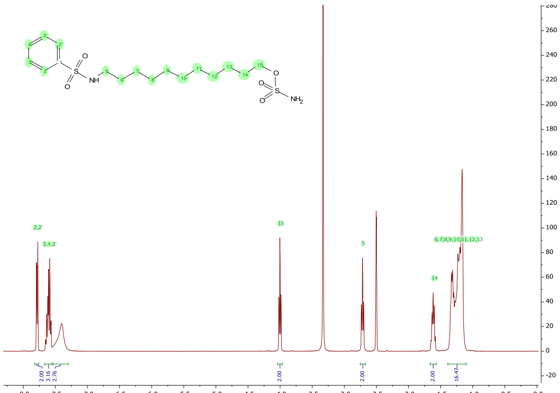

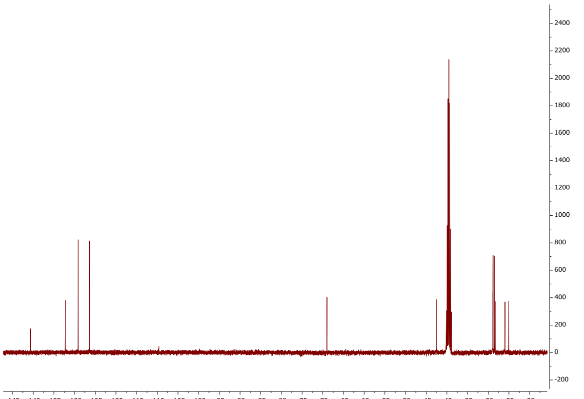

Compound 11a

^{1}H NMR (400 MHz, DMSO-d6); ^{13}C NMR (126 MHz, DMSO-d6)

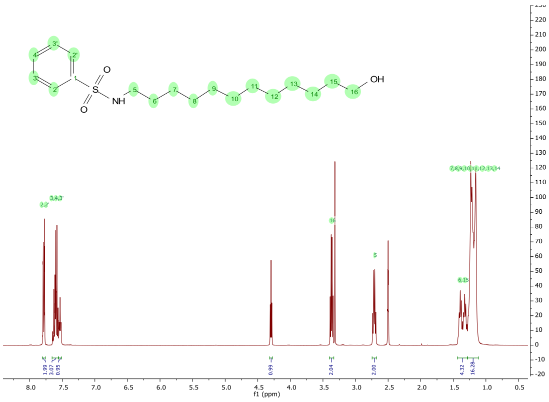

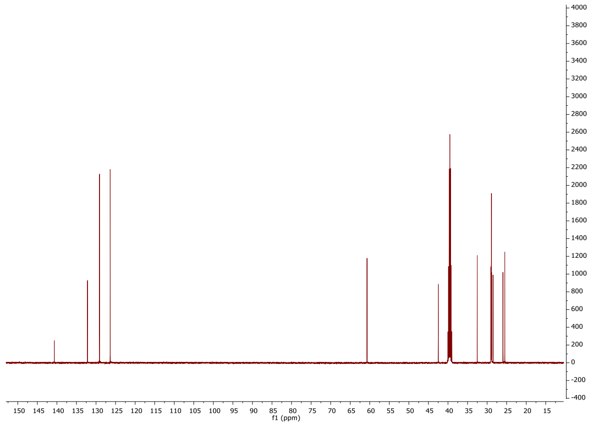

Compound 11b

^{1}H NMR (400 MHz, DMSO-d6); ^{13}C NMR (101 MHz, DMSO-d6)

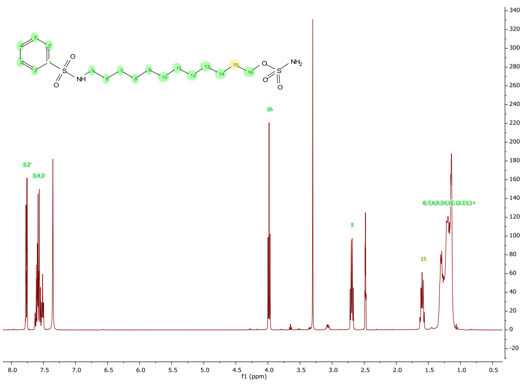

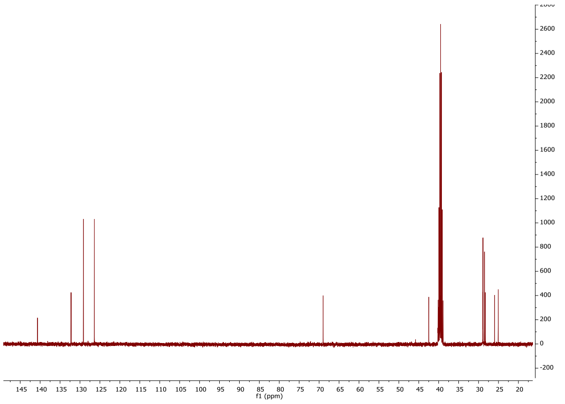

Compound 12a

^{1}H NMR (400 MHz, DMSO-d6); ^{13}C APT-NMR (101 MHz, DMSO-d6)

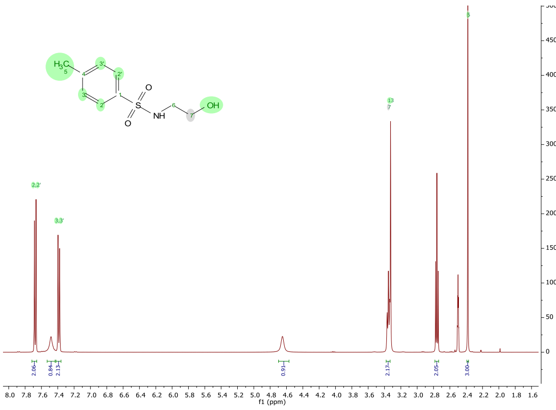

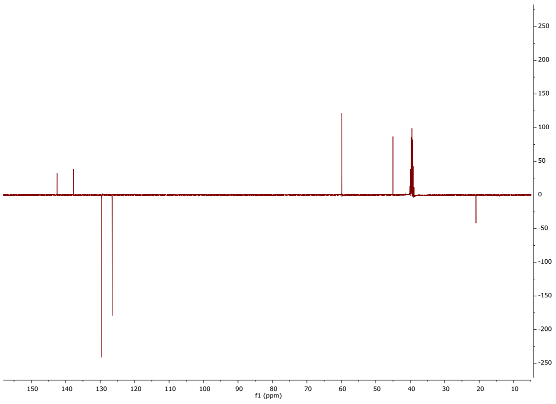

Compound 12b

^{1}H NMR (400 MHz, DMSO-d6); ^{13}C NMR (101 MHz, DMSO-d6)

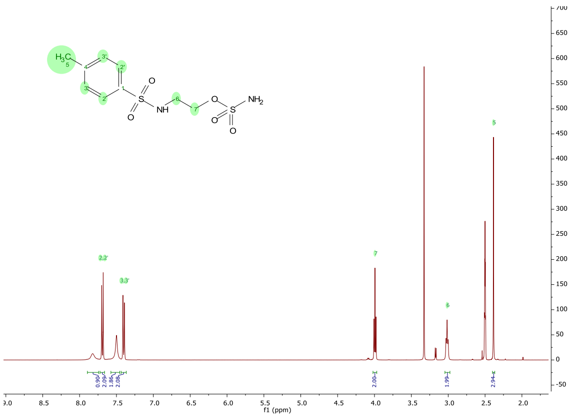

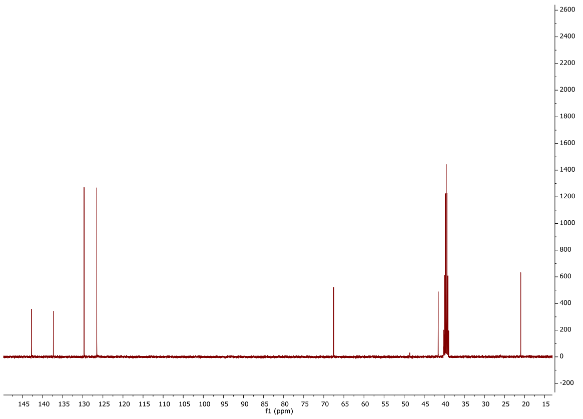

Compound 13a

^{1}H NMR (400 MHz, DMSO-d6); ^{13}C NMR (101 MHz, DMSO-d6)

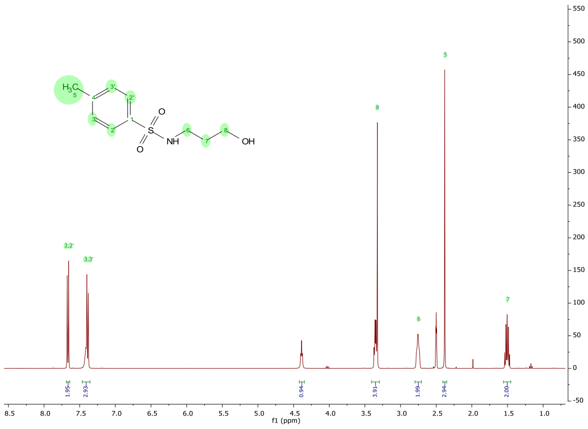

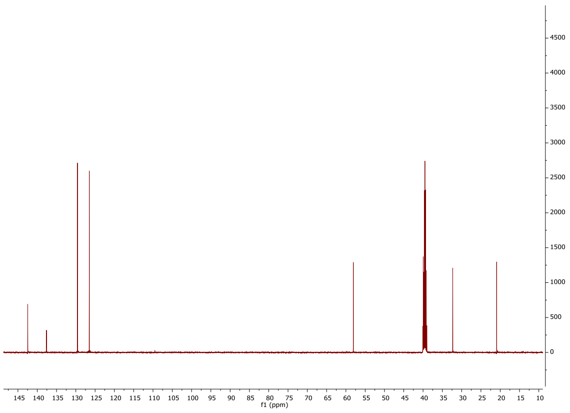

Compound 13b

^{1}H NMR (400 MHz, DMSO-d6); ^{13}C NMR (126 MHz, DMSO-d6)

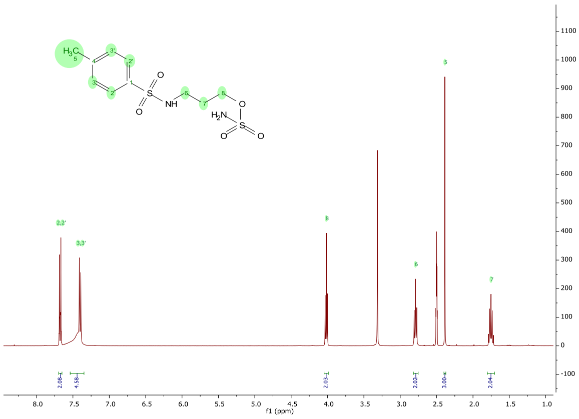

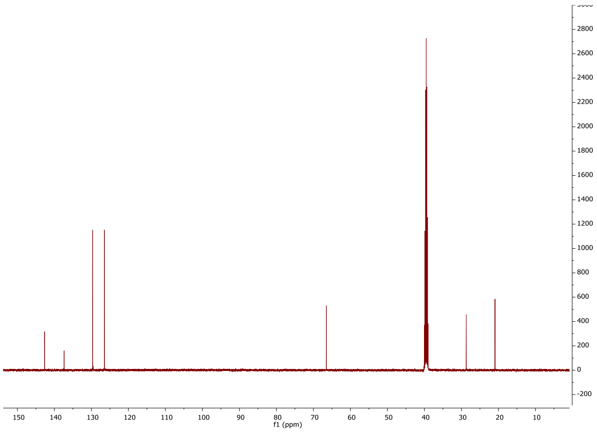

Compound 14a

^{1}H NMR (500 MHz, DMSO-d6); ^{13}C NMR (126 MHz, DMSO-d6)

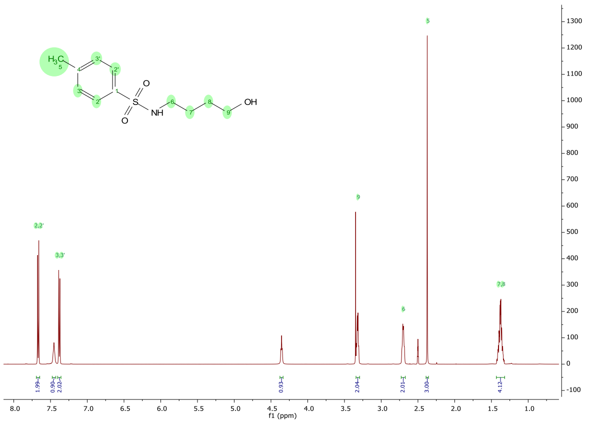

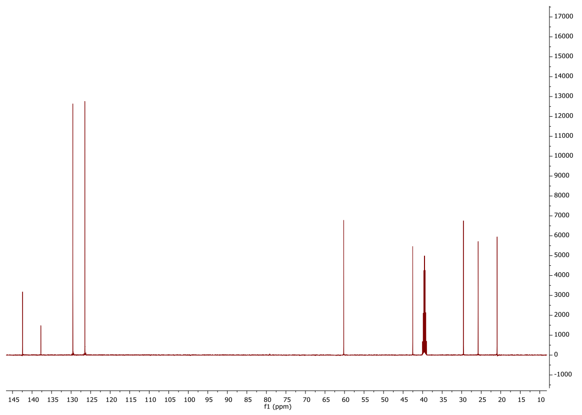

Compound 14b

^{1}H NMR (400 MHz, DMSO-d6); ^{13}C NMR (101 MHz, DMSO-d6)

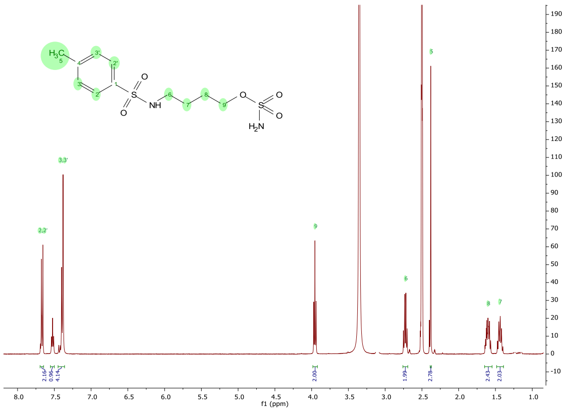

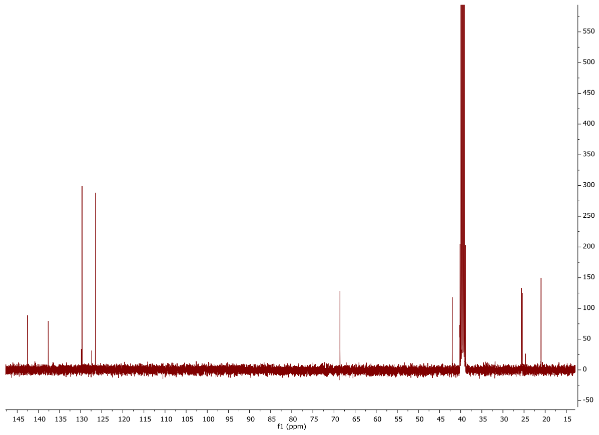

Compound 15a

^{1}H NMR (400 MHz, DMSO-d6); ^{13}C APT-NMR (101 MHz, DMSO-d6)

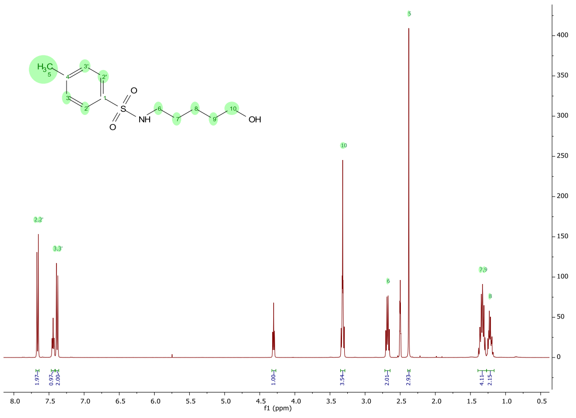

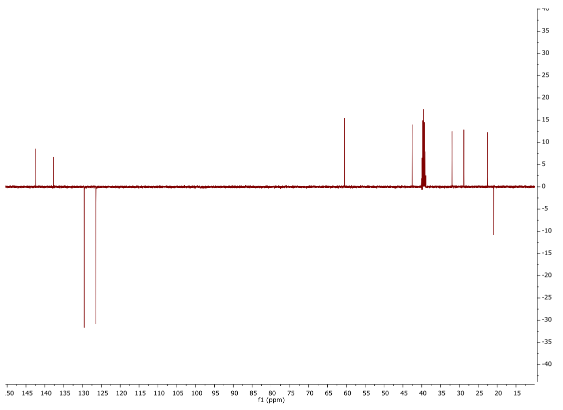

Compound 15b

^{1}H NMR (400 MHz, DMSO-d6); ^{13}C NMR (101 MHz, DMSO-d6)

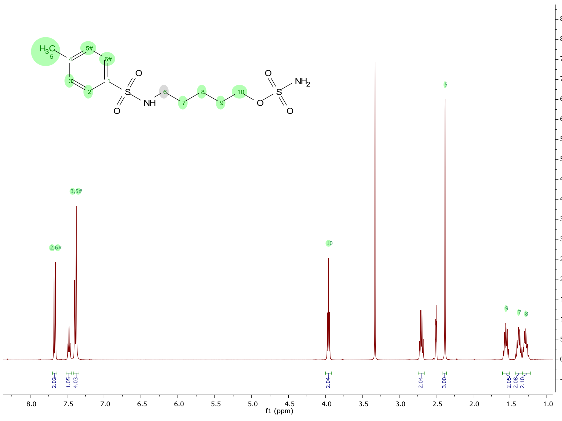

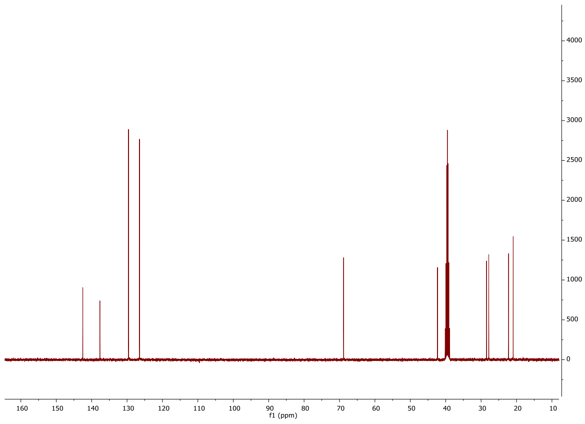

Compound 16a

^{1}H NMR (400 MHz, DMSO-d6); ^{13}C NMR (101 MHz, DMSO-d6)

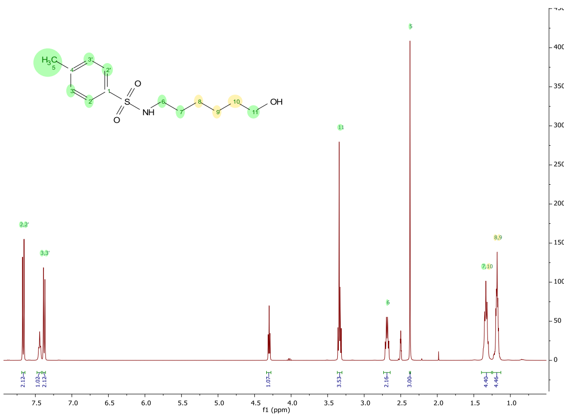

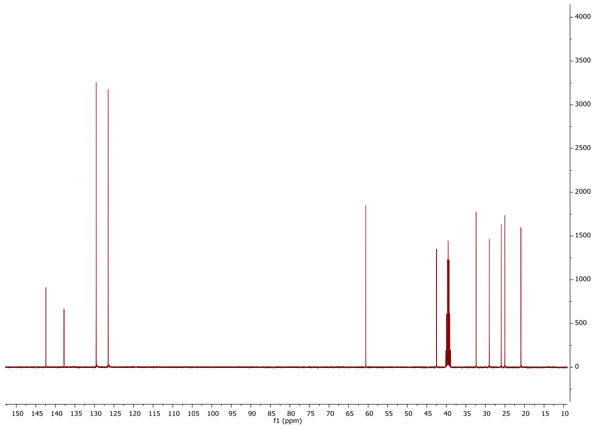

Compound 16b

^{1}H NMR (500 MHz, DMSO-d6); ^{13}C NMR (101 MHz, DMSO-d6)

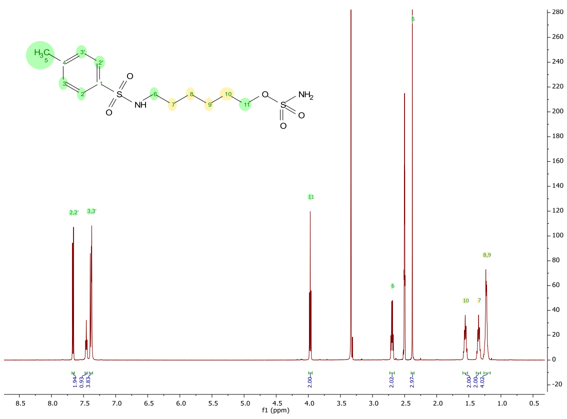

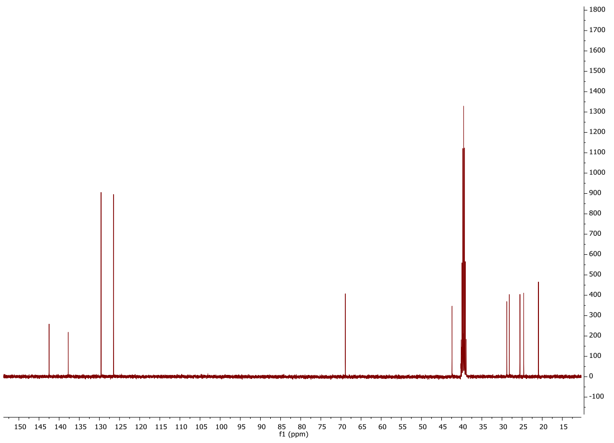

Compound 17a

^{1}H NMR (400 MHz, DMSO-d6); ^{13}C NMR (101 MHz, DMSO-d6)

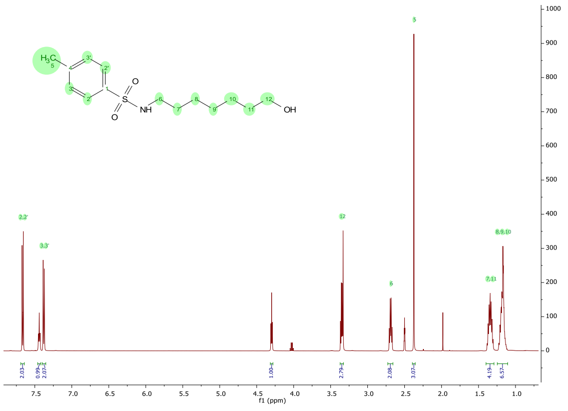

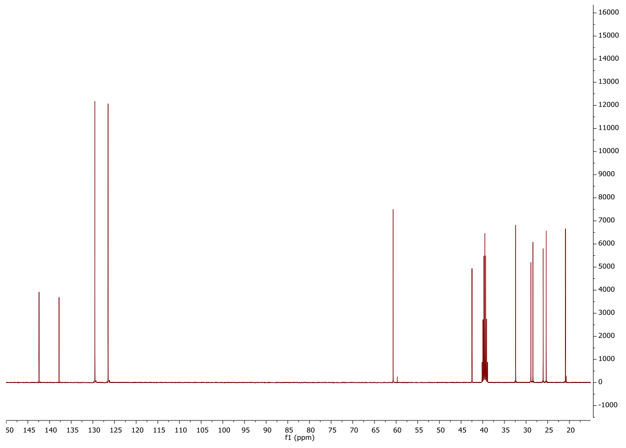

Compound 17b

^{1}H NMR (400 MHz, DMSO-d6); ^{13}C NMR (101 MHz, DMSO-d6)

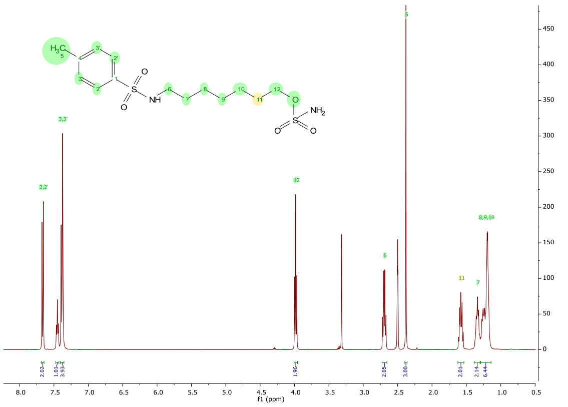

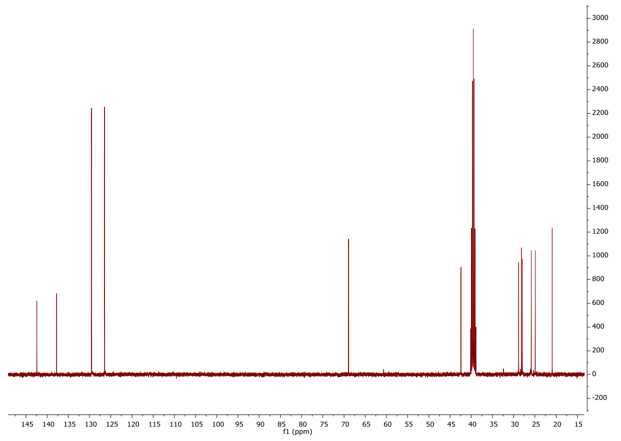

Compound 18a

^{1}H NMR (400 MHz, DMSO-d6); ^{13}C NMR (101 MHz, DMSO-d6)

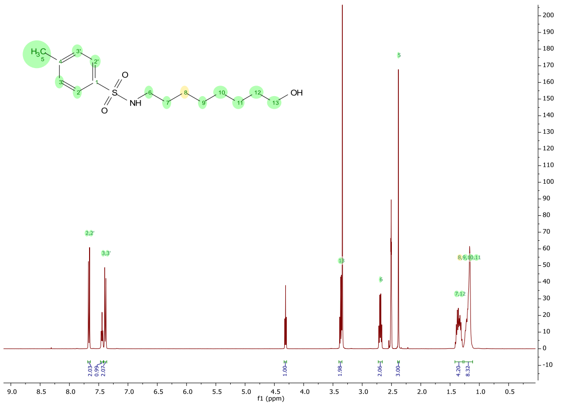

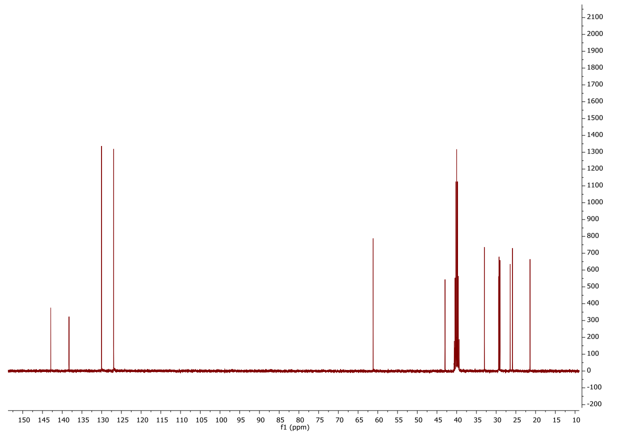

Compound 18b

^{1}H NMR (400 MHz, DMSO-d6); ^{13}C APT-NMR (101 MHz, DMSO-d6)

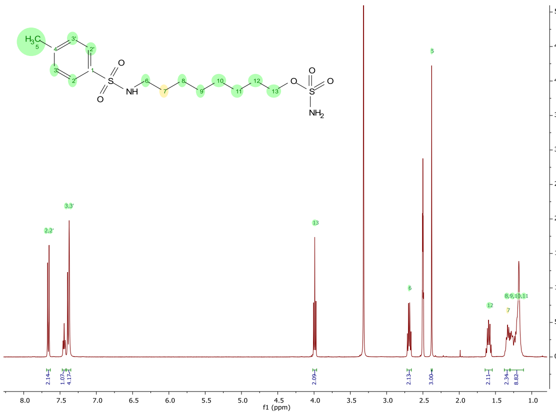

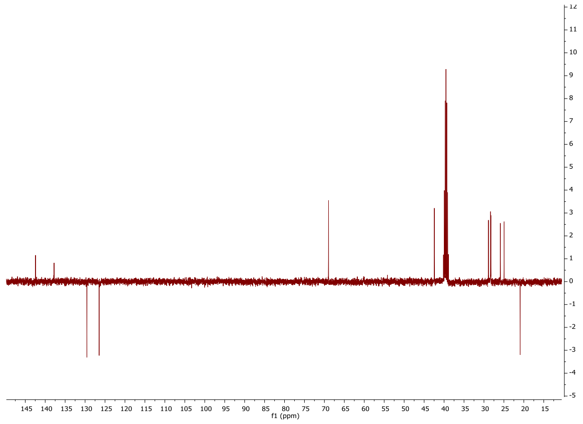

Compound 19a

^{1}H NMR (400 MHz, DMSO-d6); ^{13}C NMR (101 MHz, DMSO-d6)

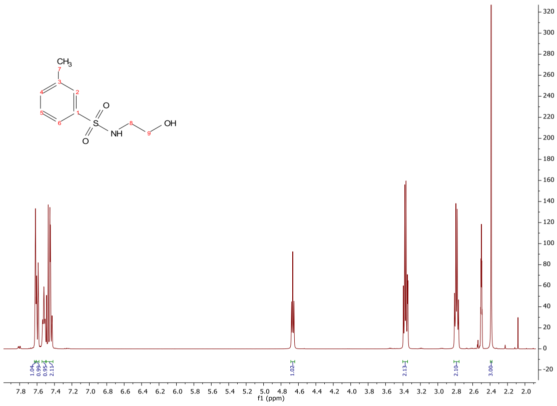

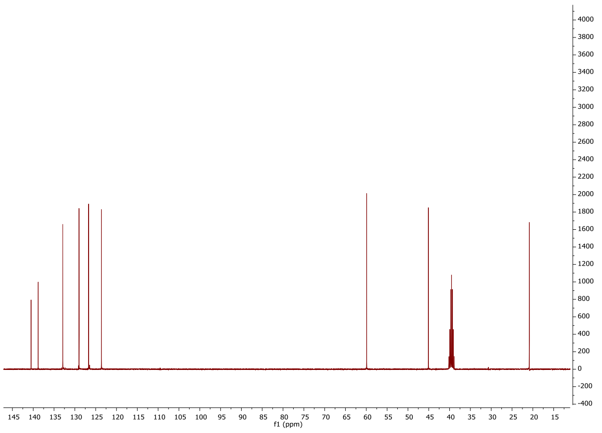

Compound 19b

^{1}H NMR (400 MHz, DMSO-d6); ^{13}C NMR (101 MHz, DMSO-d6)

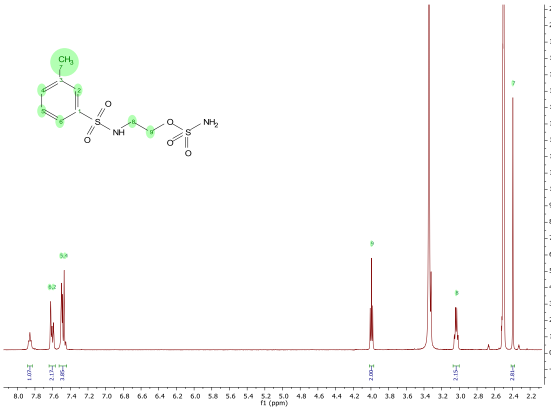

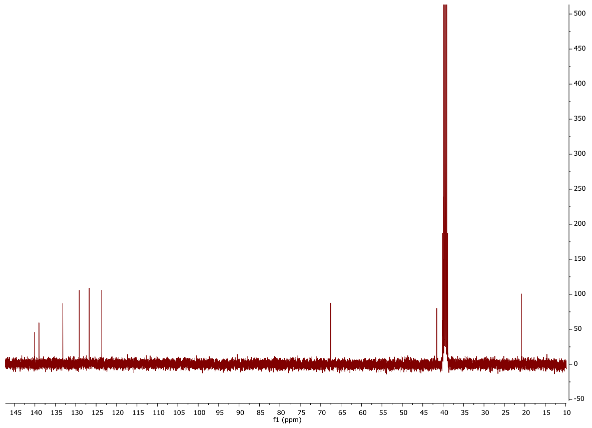

Compound 20a

^{1}H NMR (400 MHz, DMSO-d6); ^{13}C NMR (101 MHz, DMSO-d6)

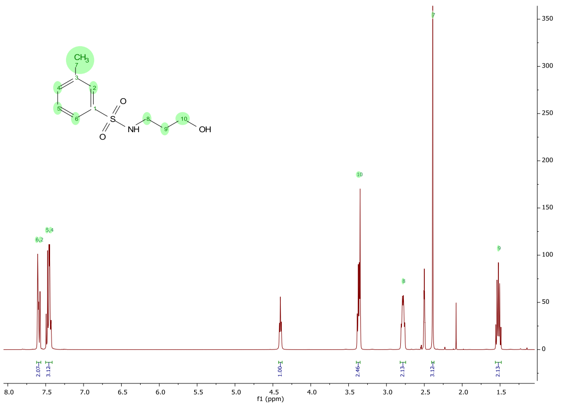

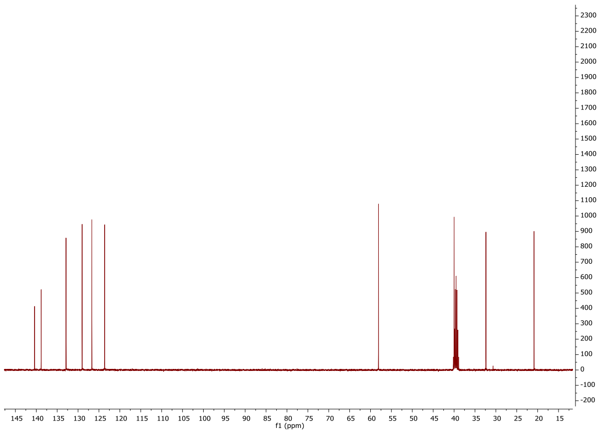

Compound 20b

^{1}H NMR (400 MHz, DMSO-d6); ^{13}C NMR (101 MHz, DMSO-d6)

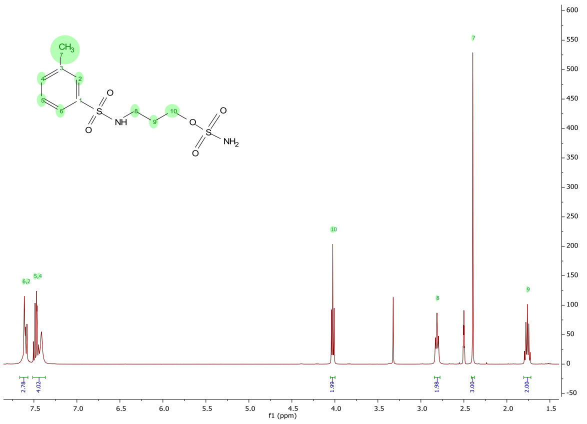

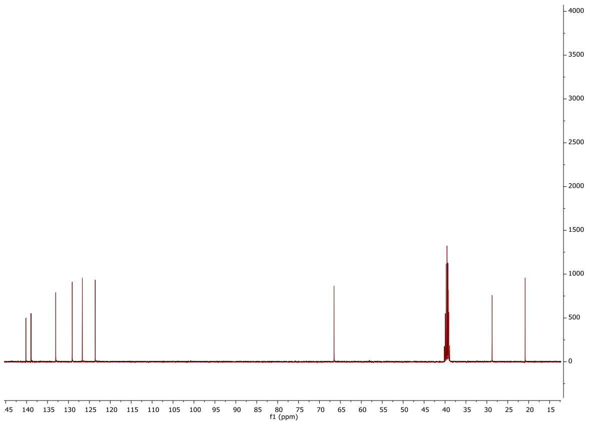

Compound 21a

^{1}H NMR (400 MHz, DMSO-d6); ^{13}C NMR (101 MHz, DMSO-d6)

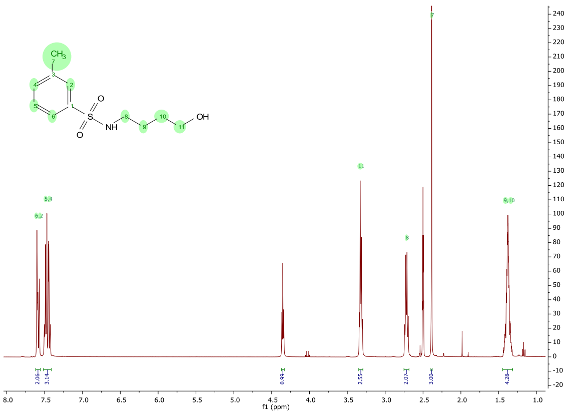

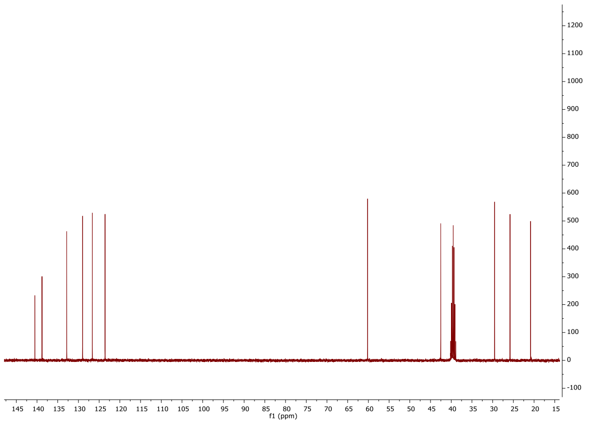

Compound 21b

^{1}H NMR (400 MHz, DMSO-d6); ^{13}C NMR (101 MHz, DMSO-d6)

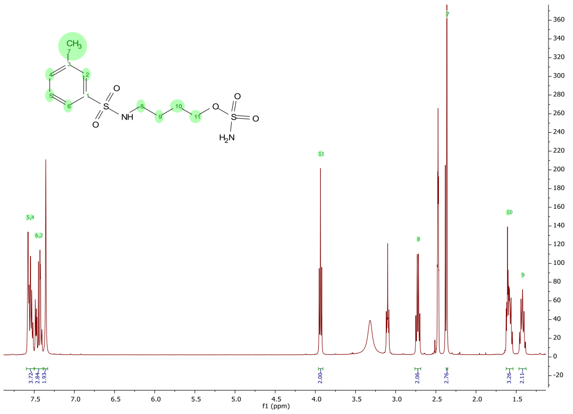

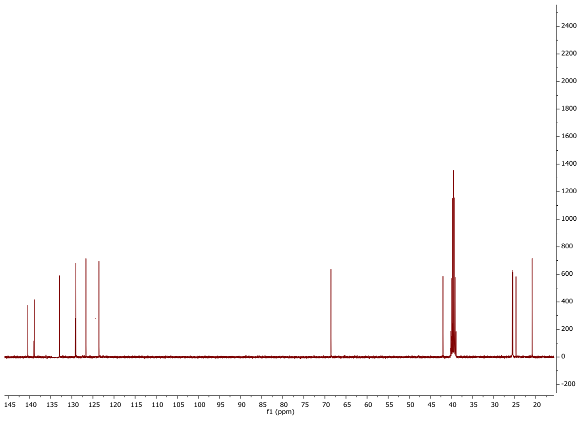

Compound 22a

^{1}H NMR (400 MHz, DMSO-d6); ^{13}C NMR (101 MHz, DMSO-d6)

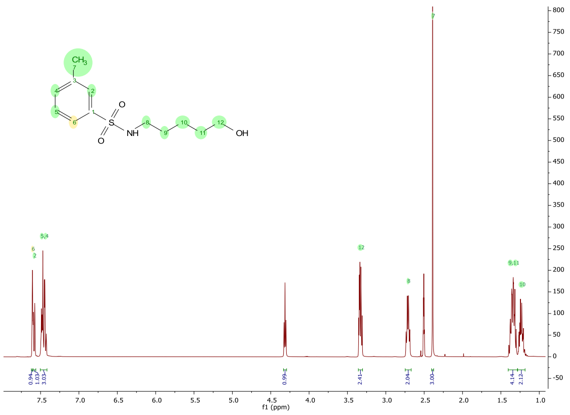

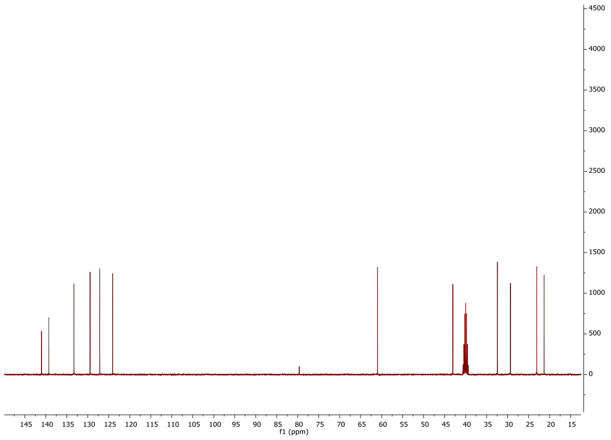

Compound 22b

^{1}H NMR (400 MHz, DMSO-d6); ^{13}C APT-NMR (101 MHz, DMSO-d6)

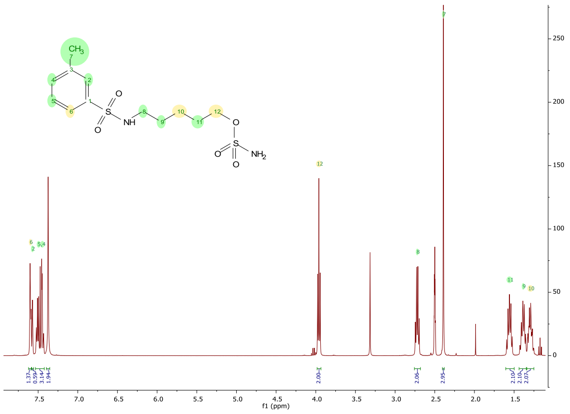

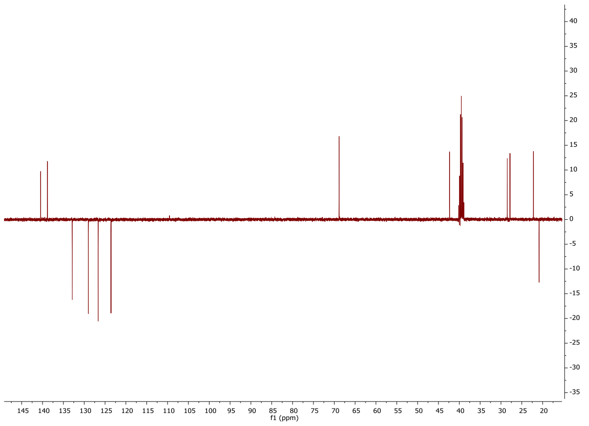

Compound 23a

^1H NMR (400 MHz, DMSO-d6); ^{13}C NMR (101 MHz, DMSO-d6)

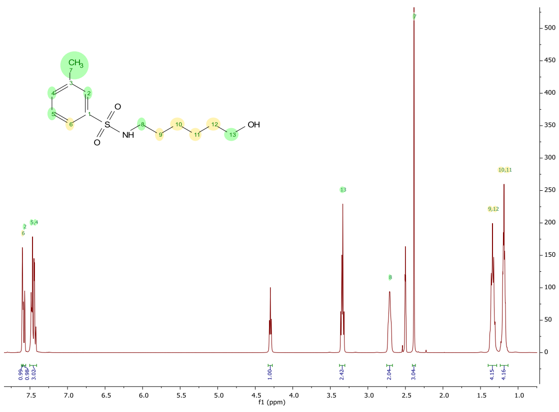

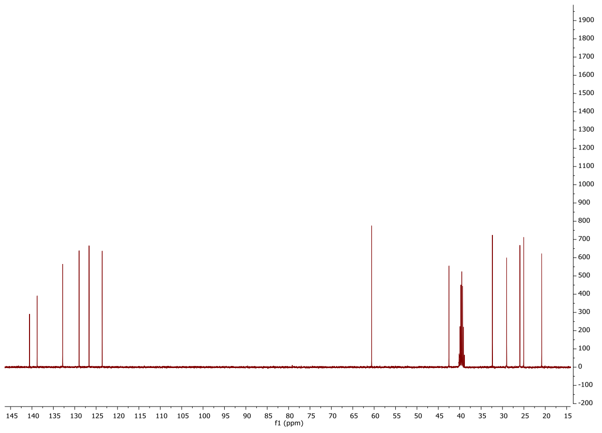

Compound 23b

^1H NMR (400 MHz, DMSO-d6); ^{13}C NMR (101 MHz, DMSO-d6)

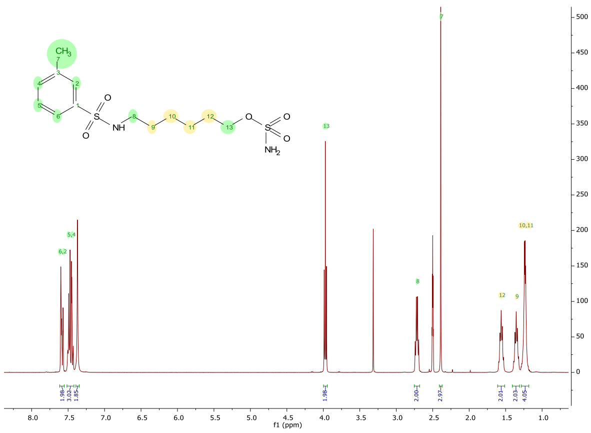

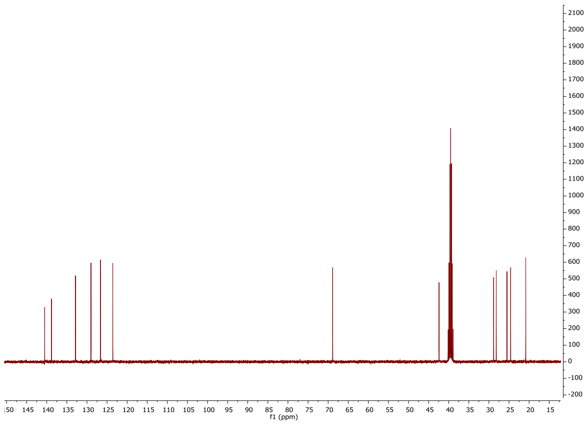

Compound 24a

^1H NMR (400 MHz, DMSO-d6); ^{13}C NMR (126 MHz, DMSO-d6)

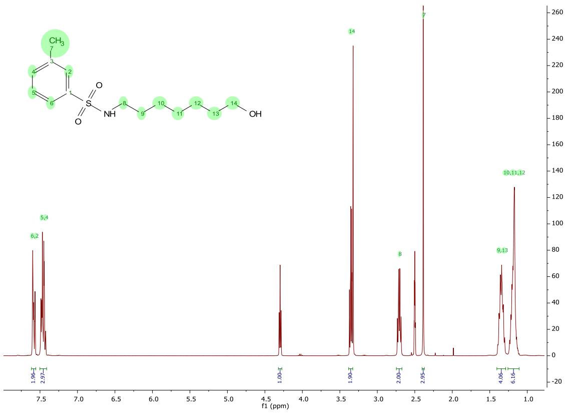

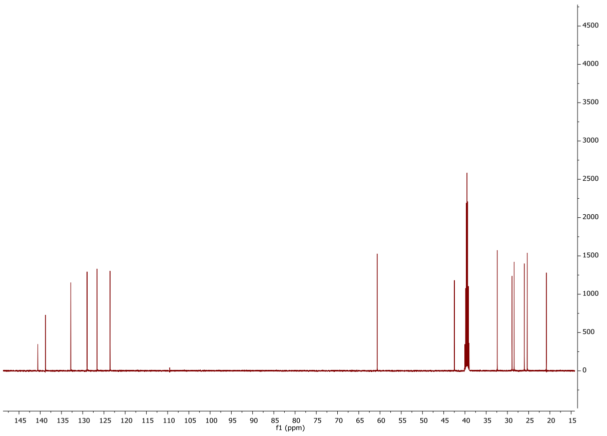

Compound 24b

^{1}H NMR (400 MHz, DMSO-d6); ^{13}C NMR (126 MHz, DMSO-d6)

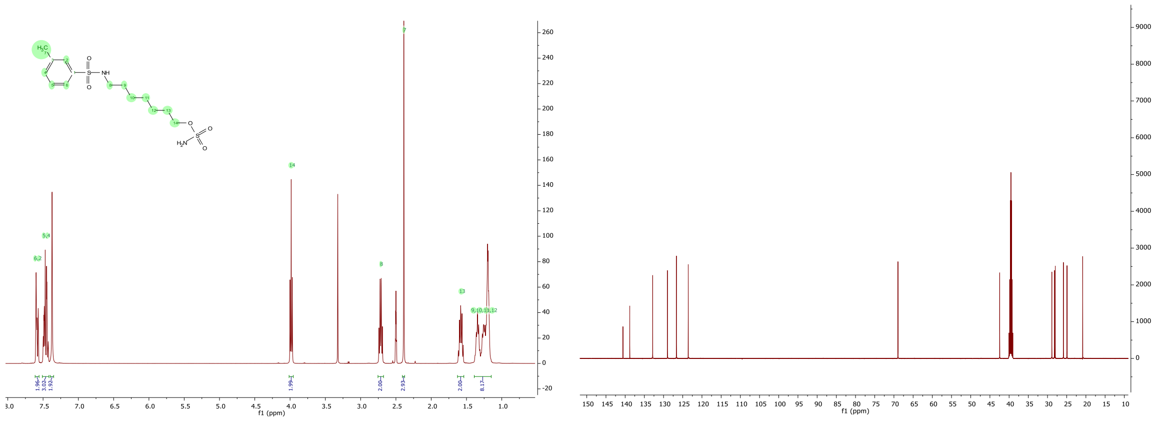

Compound 25a

^{1}H NMR (400 MHz, DMSO-d6); ^{13}C NMR (101 MHz, DMSO-d6)

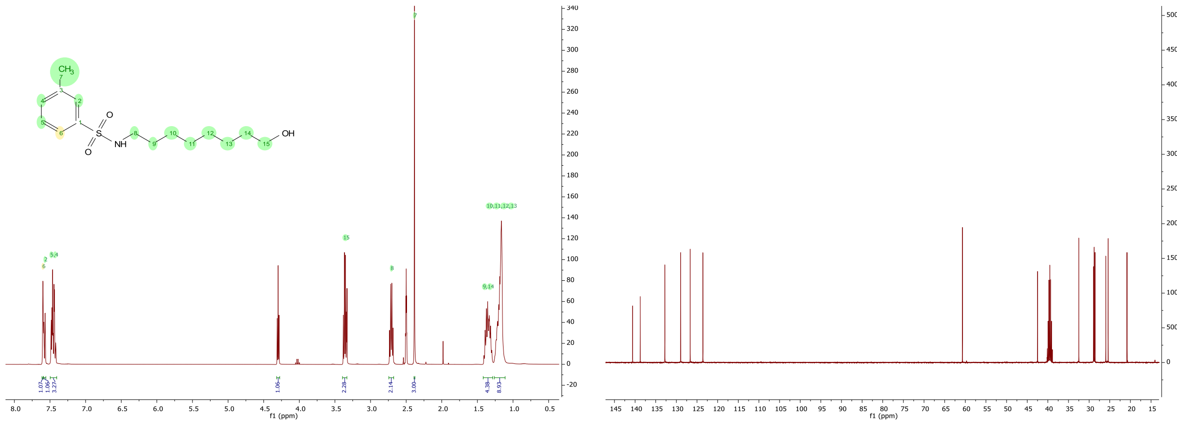

Compound 25b

^{1}H NMR (400 MHz, DMSO-d6); ^{13}C NMR (126 MHz, DMSO-d6)

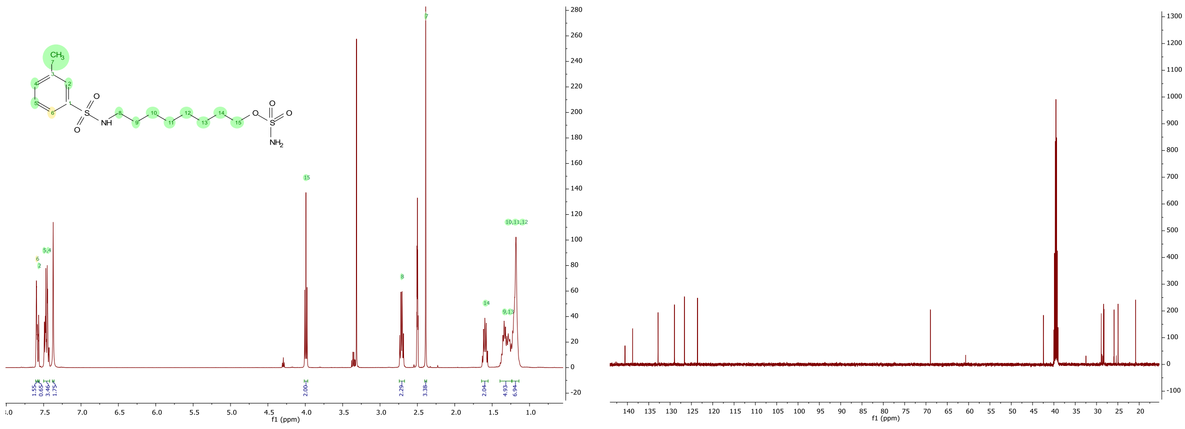

Compound 26a

^{1}H NMR (500 MHz, DMSO-d6); ^{13}C NMR (126 MHz, DMSO-d6)

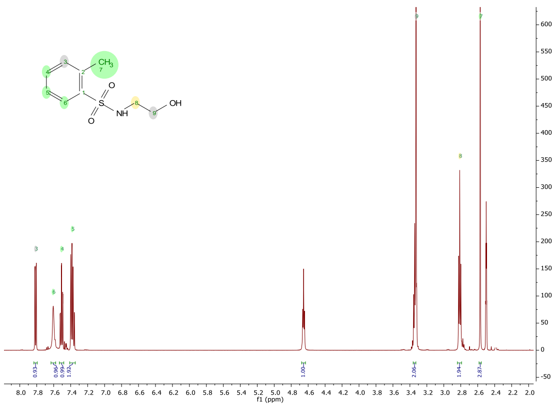

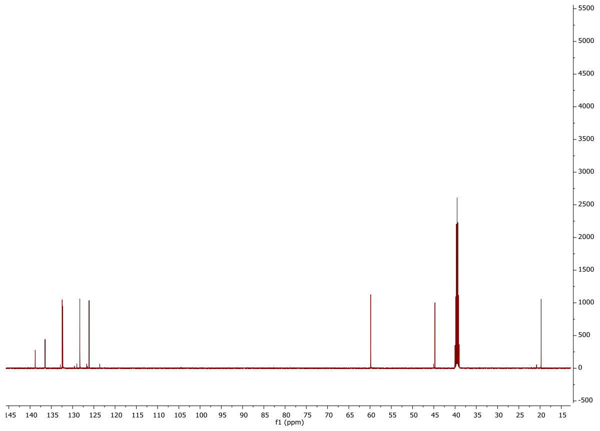

Compound 26b

^{1}H NMR (400 MHz, DMSO-d6); ^{13}C NMR (126 MHz, DMSO-d6)

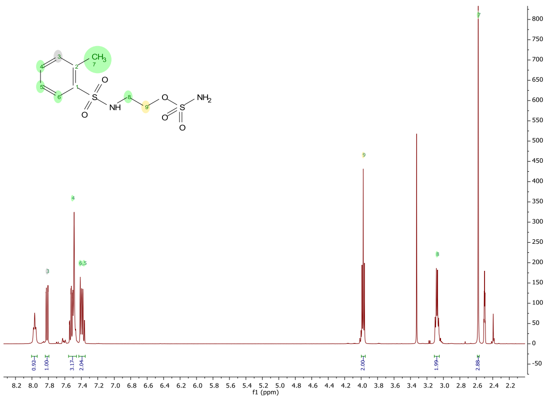

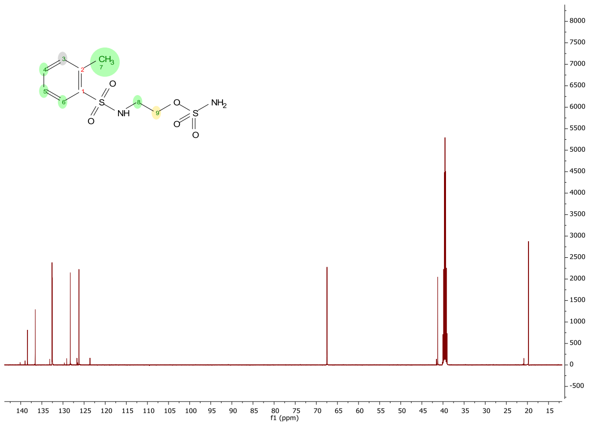

Compound 27a

^{1}H NMR (400 MHz, DMSO-d6); ^{13}C NMR (101 MHz, DMSO-d6)

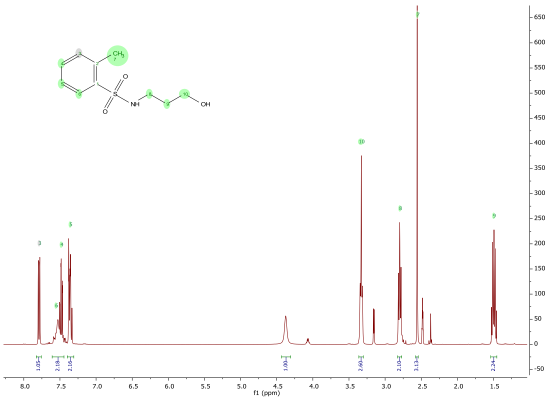

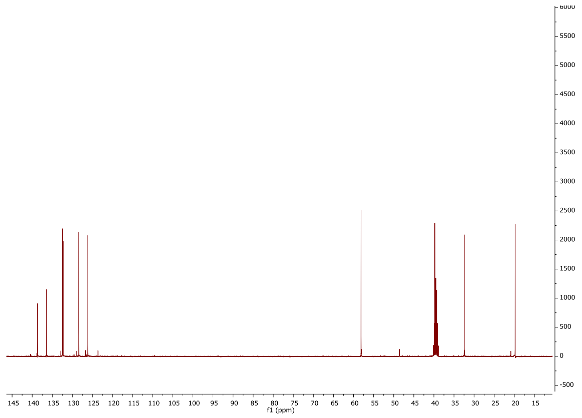

Compound 27b

^{1}H NMR (400 MHz, DMSO-d6); ^{13}C NMR (126 MHz, DMSO-d6)

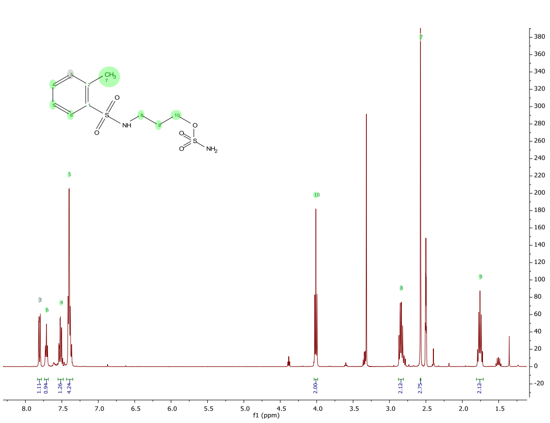

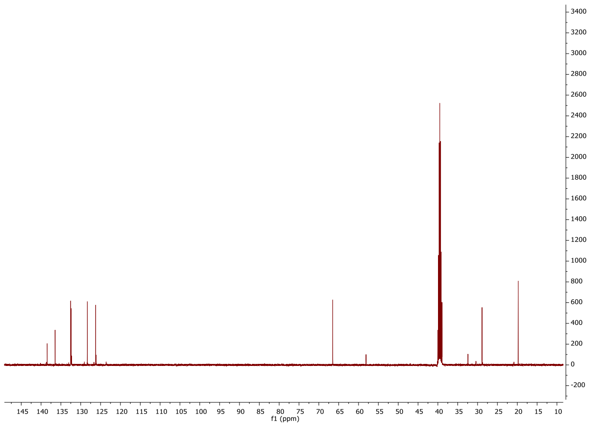

Compound 28a

^{1}H NMR (400 MHz, DMSO-d6); ^{13}C NMR (101 MHz, DMSO-d6)

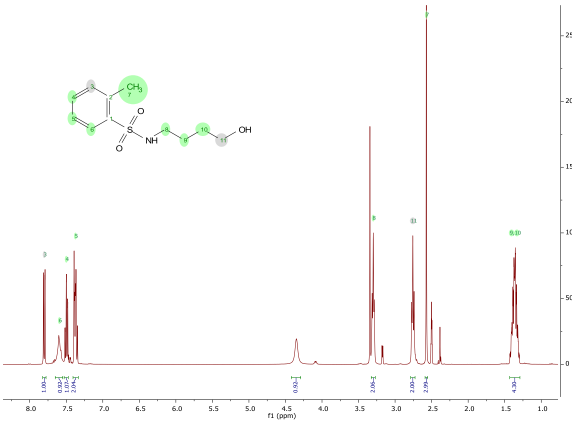

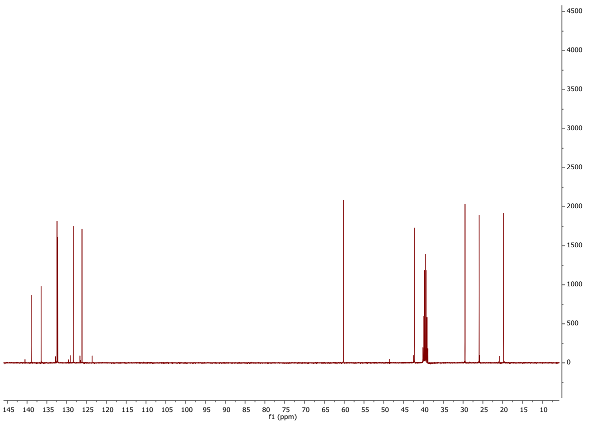

Compound 28b

^{1}H NMR (400 MHz, DMSO-d6); ^{13}C APT-NMR (101 MHz, DMSO-d6)

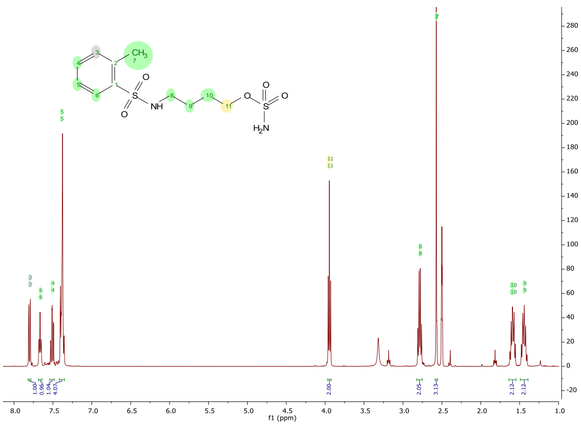

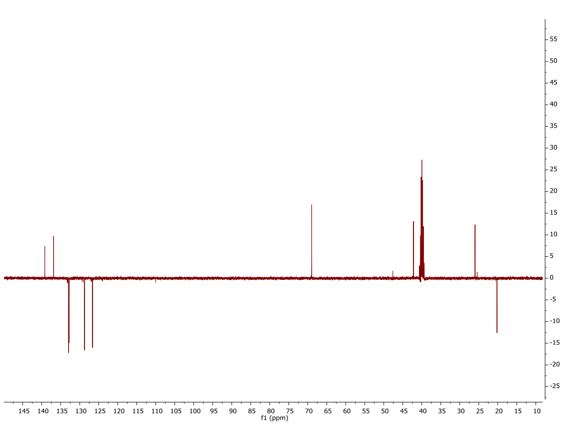

Compound 29a

^{1}H NMR (400 MHz, DMSO-d6); ^{13}C NMR (126 MHz, DMSO-d6)

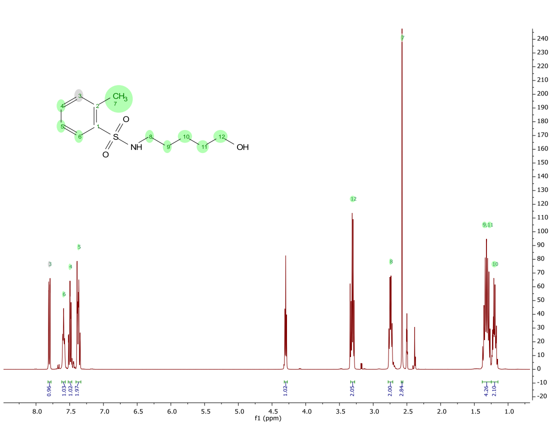

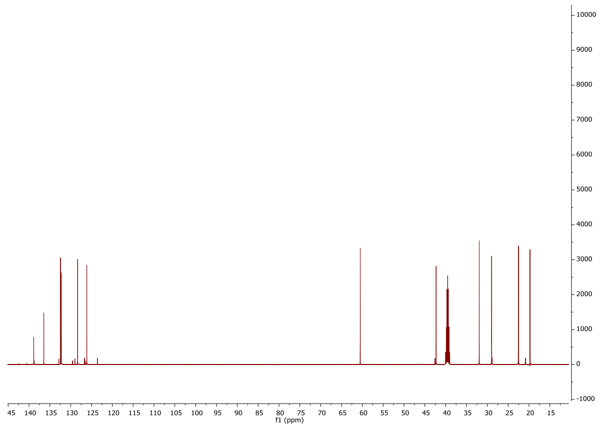

Compound 29b

^{1}H NMR (400 MHz, DMSO-d6); ^{13}C NMR (101 MHz, DMSO-d6)

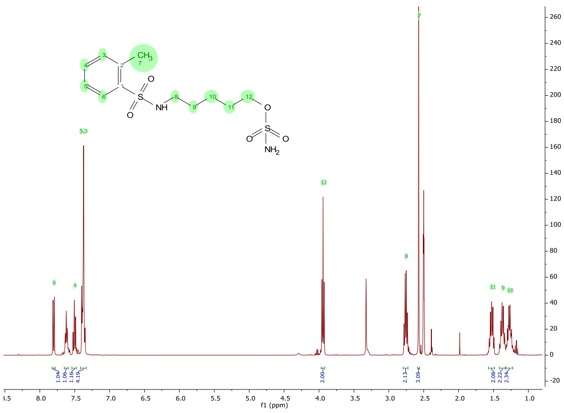

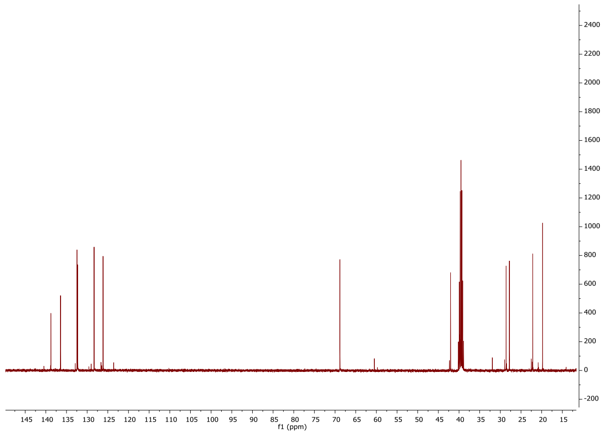

Compound 30a

^{1}H NMR (400 MHz, DMSO-d6); ^{13}C NMR (101 MHz, DMSO-d6)

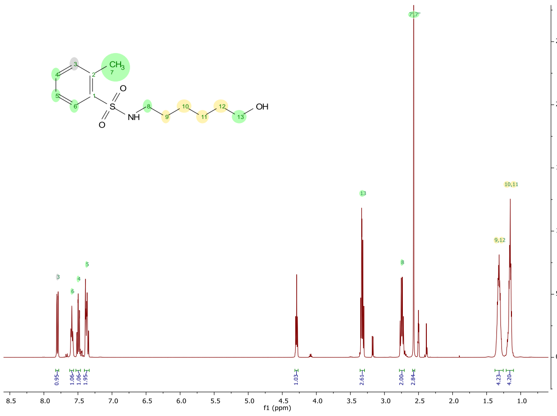

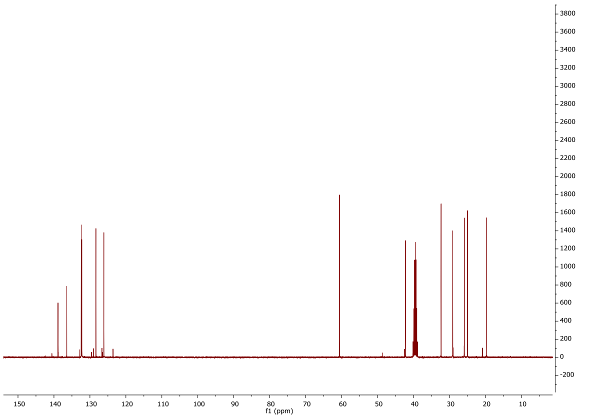

Compound 30b

^{1}H NMR (400 MHz, DMSO-d6); ^{13}C NMR (101 MHz, DMSO-d6)

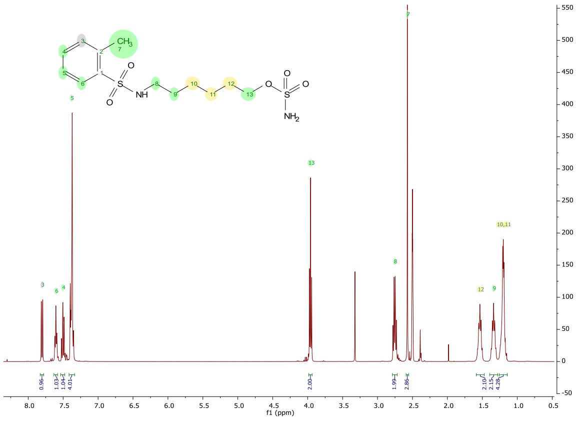

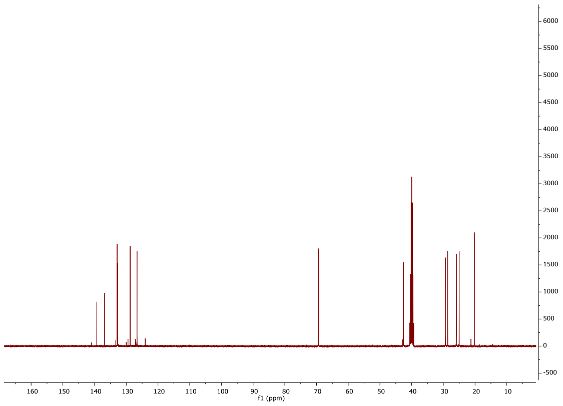

Compound 31a

^{1}H NMR (400 MHz, DMSO-d6); ^{13}C NMR (126 MHz, DMSO-d6)

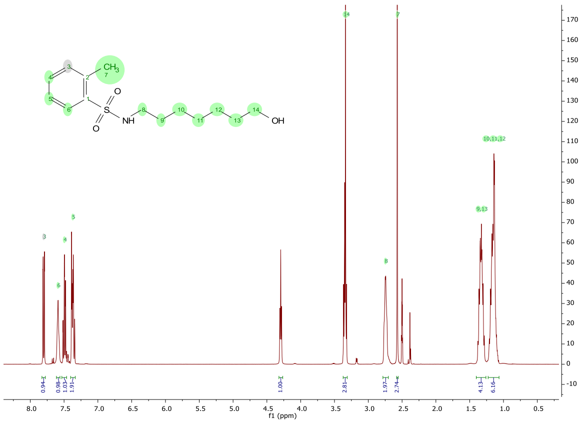

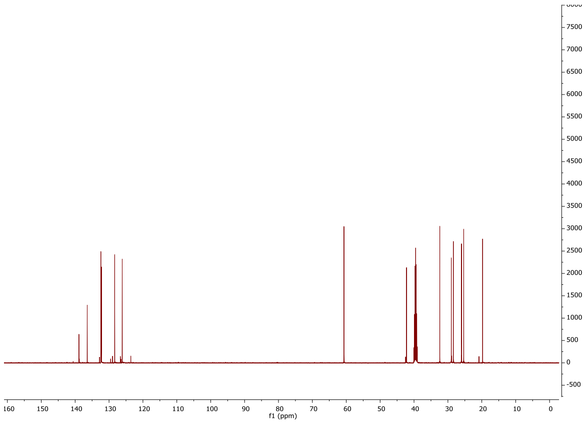

Compound 31b

^{1}H NMR (500 MHz, DMSO-d6); ^{13}C NMR (101 MHz, DMSO-d6)

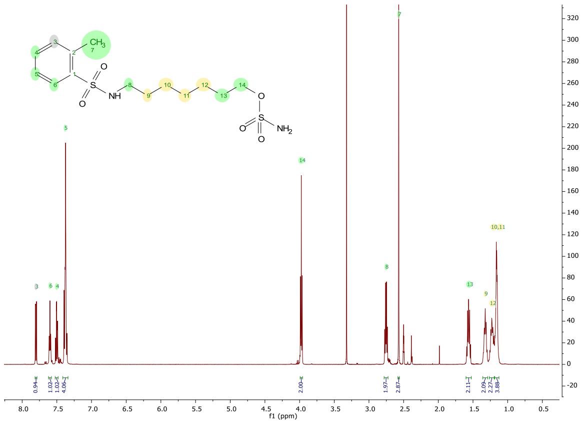

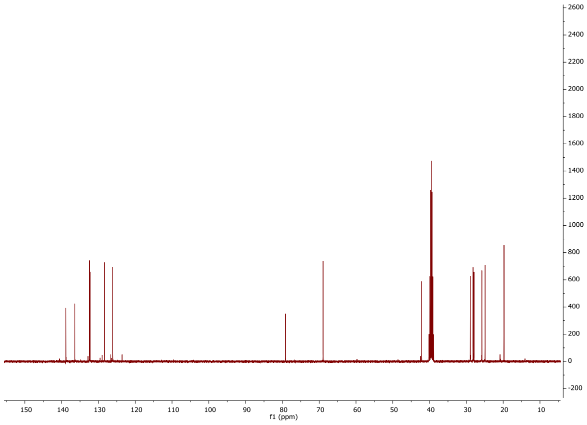

Compound 32a

^1H NMR (400 MHz, DMSO-d6); ^{13}C NMR (101 MHz, DMSO-d6)

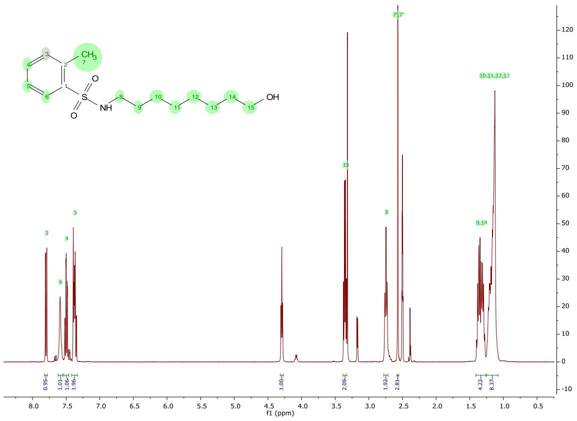

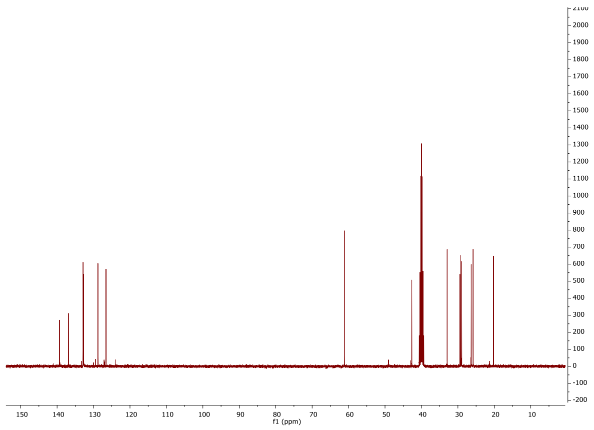

Compound 32b

^1H NMR (500 MHz, DMSO-d6); ^{13}C NMR (101 MHz, DMSO-d6)

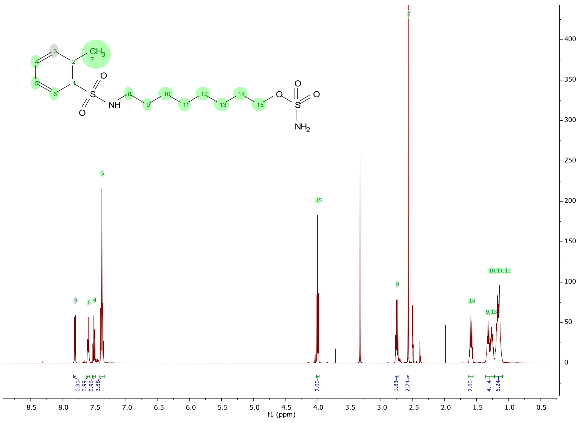

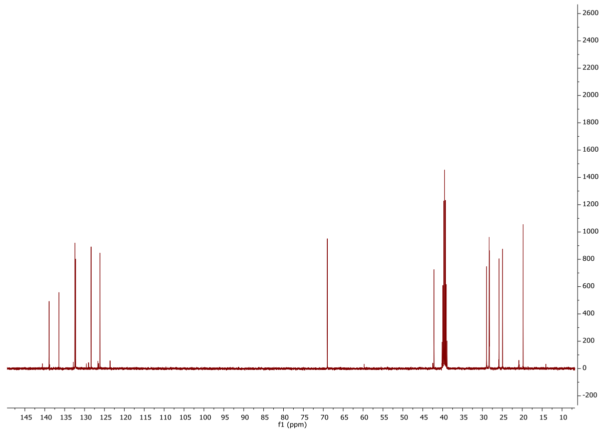

Compound 33a

^1H NMR (400 MHz, DMSO-d6); ^{13}C NMR (101 MHz, DMSO-d6)

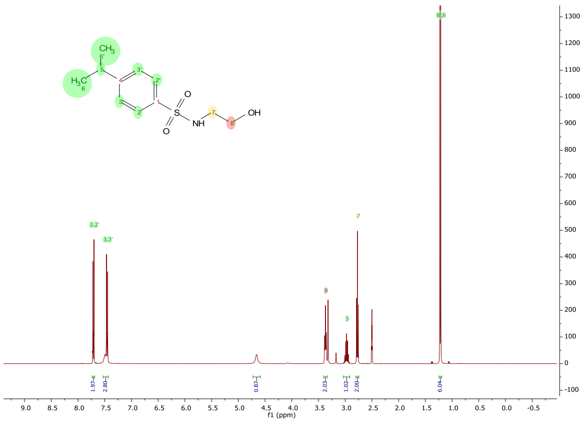

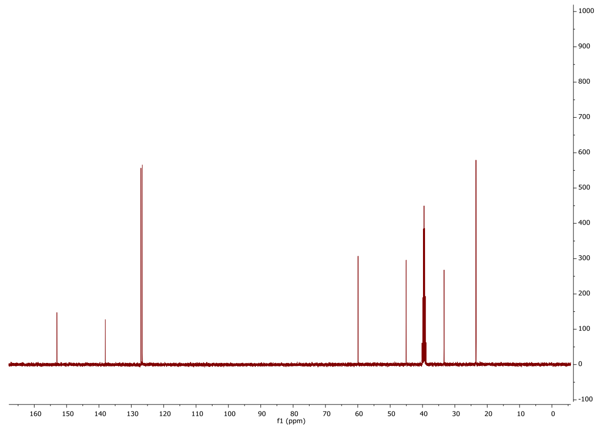

Compound 33b

^{1}H NMR (400 MHz, DMSO-d6); ^{13}C NMR (101 MHz, DMSO-d6)

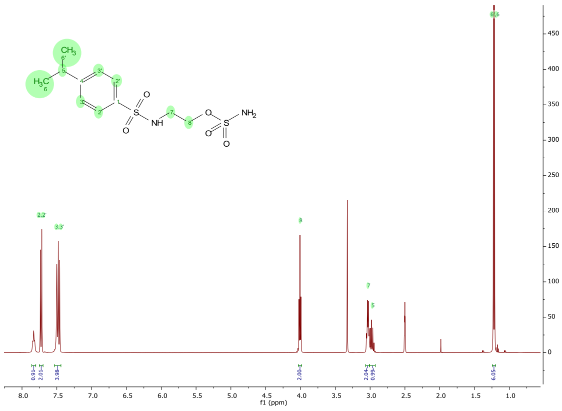

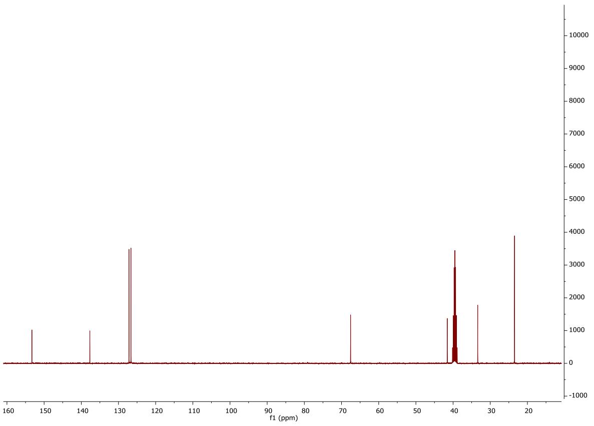

Compound 34a

^{1}H NMR (400 MHz, DMSO-d6); ^{13}C NMR (101 MHz, DMSO-d6)

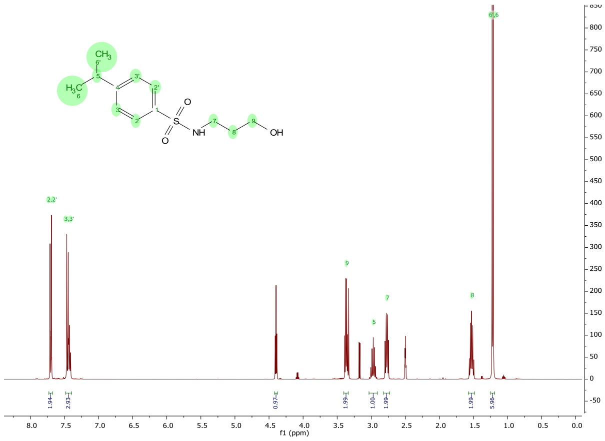

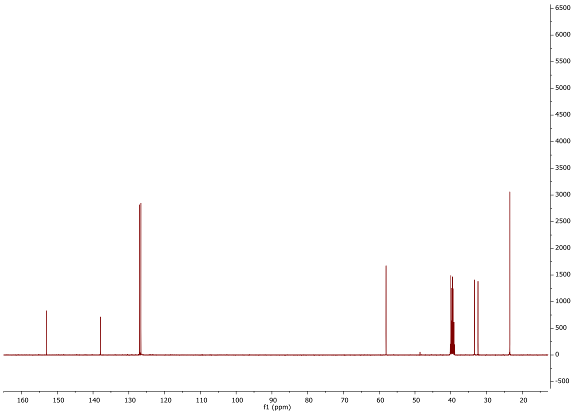

Compound 34b

^{1}H NMR (400 MHz, DMSO-d6); ^{13}C NMR (101 MHz, DMSO-d6)

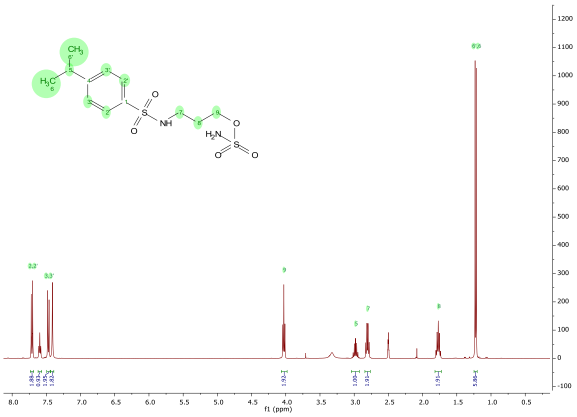

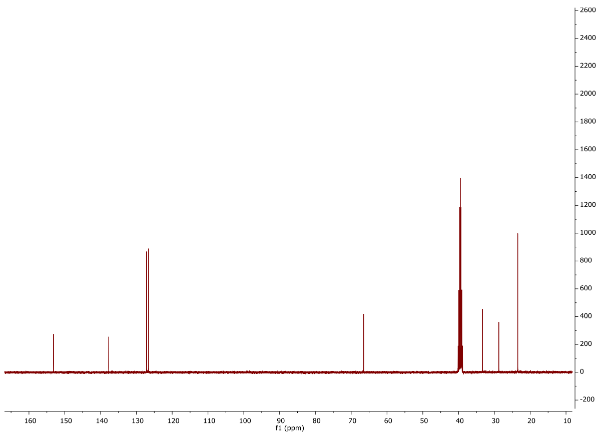

Compound 35a

^{1}H NMR (400 MHz, DMSO-d6); ^{13}C NMR (101 MHz, DMSO-d6)

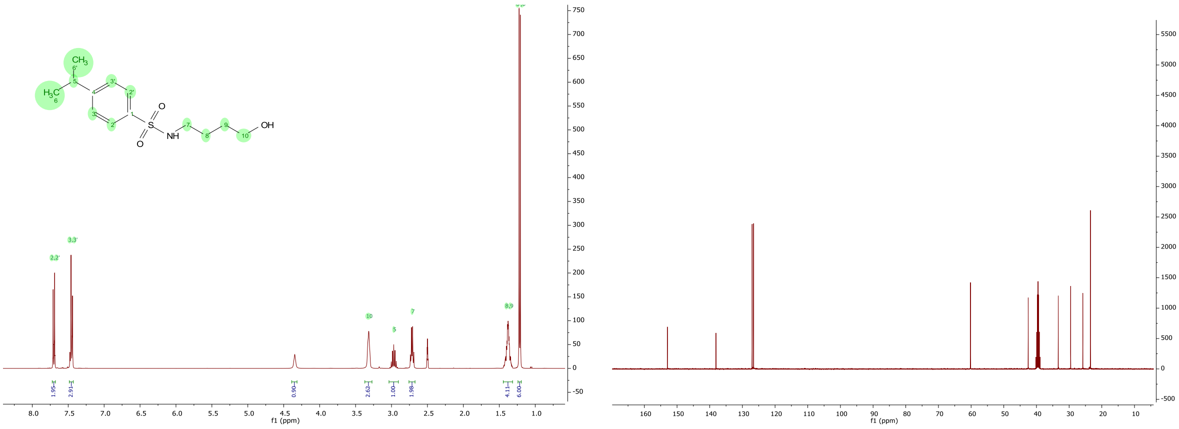

Compound 35b

^{1}H NMR (400 MHz, DMSO-d6); ^{13}C NMR (126 MHz, DMSO-d6)

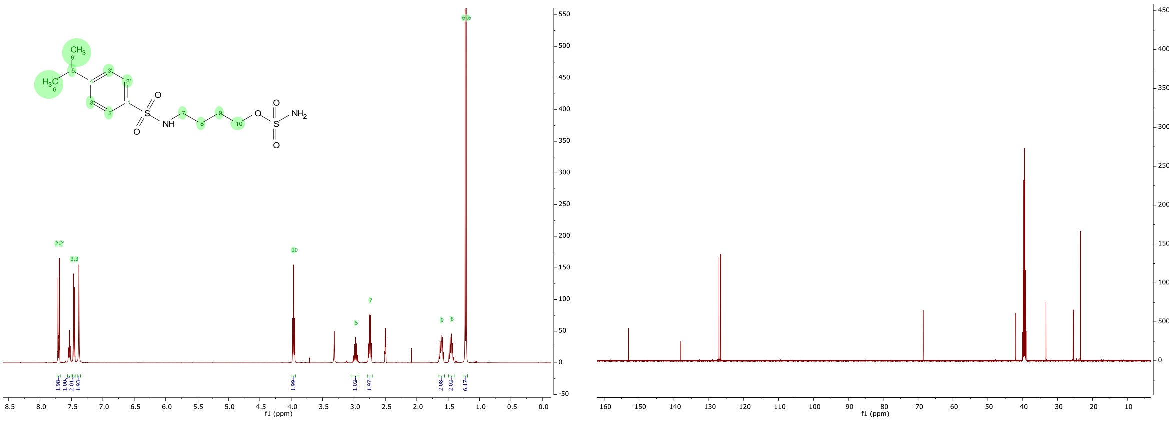

Compound 36a

^{1}H NMR (400 MHz, DMSO-d6); ^{13}C NMR (101 MHz, DMSO-d6)

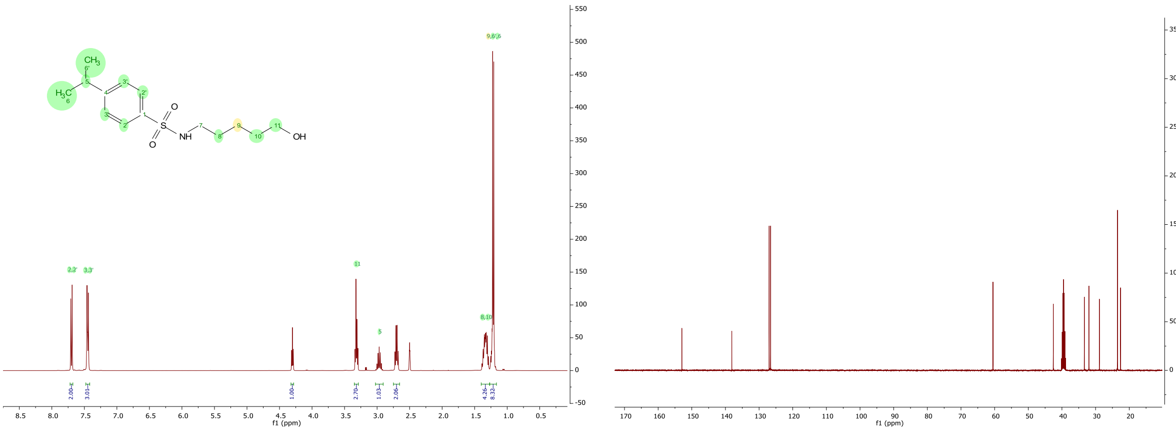

Compound 36b

^{1}H NMR (400 MHz, DMSO-d6); ^{13}C APT-NMR (101 MHz, DMSO-d6)

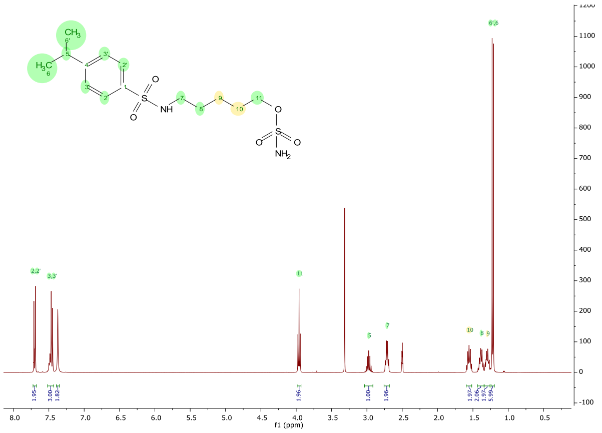

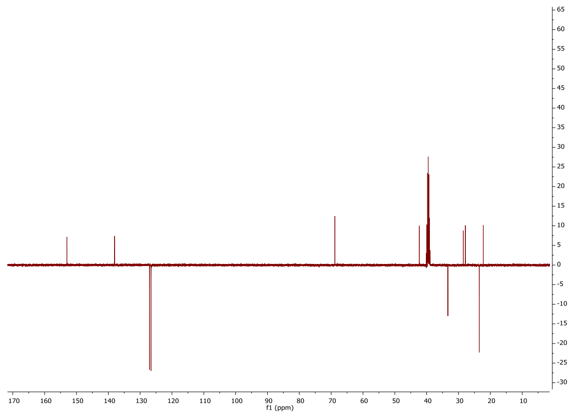

Compound 37a

^{1}H NMR (400 MHz, DMSO-d6); ^{13}C NMR (101 MHz, DMSO-d6)

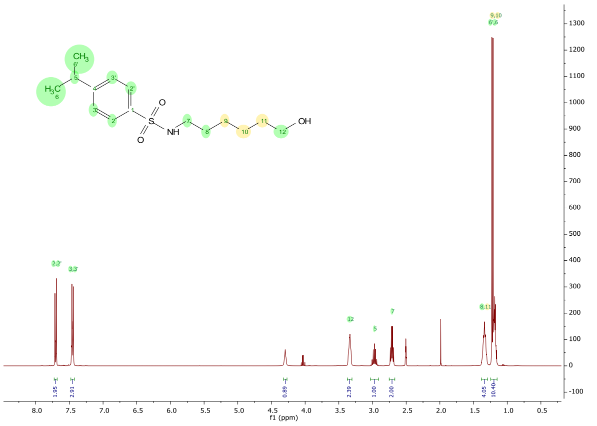

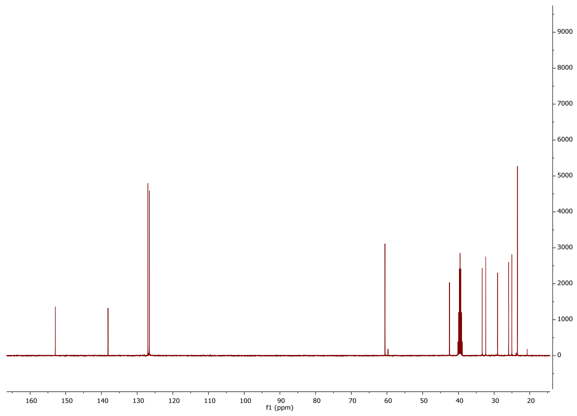

Compound 37b

^{1}H NMR (400 MHz, DMSO-d6); ^{13}C NMR (101 MHz, DMSO-d6)

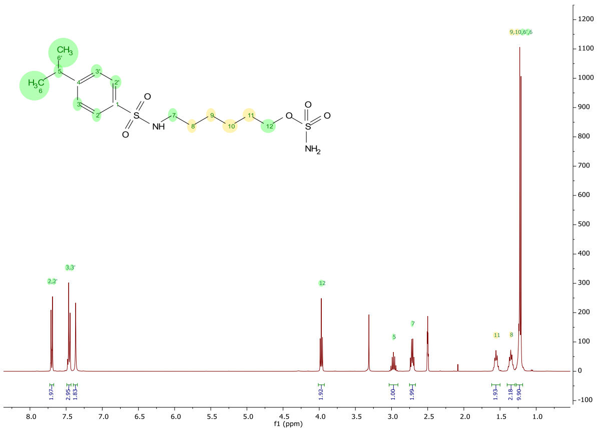

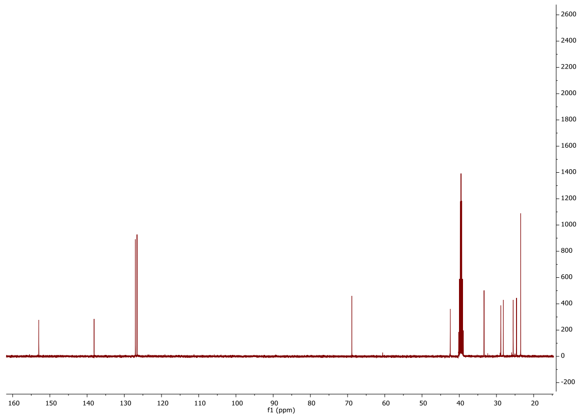

Compound 38a

^{1}H NMR (400 MHz, DMSO-d6); ^{13}C NMR (126 MHz, DMSO-d6)

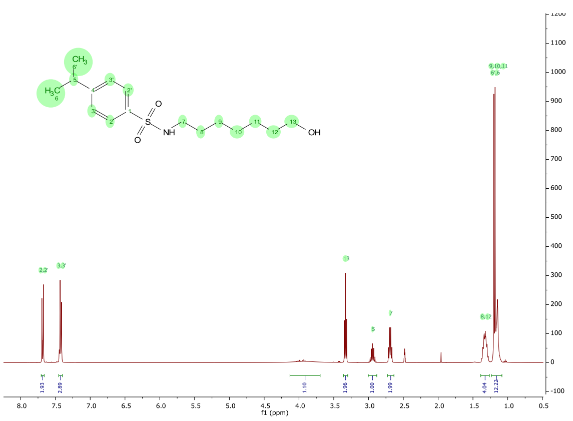

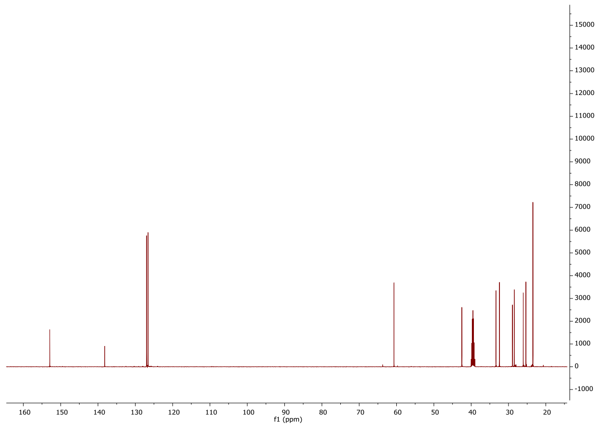

Compound 38b

^{1}H NMR (400 MHz, DMSO-d6); ^{13}C NMR (126 MHz, DMSO-d6)

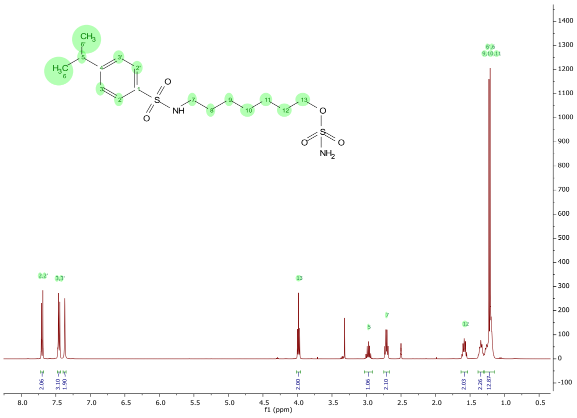

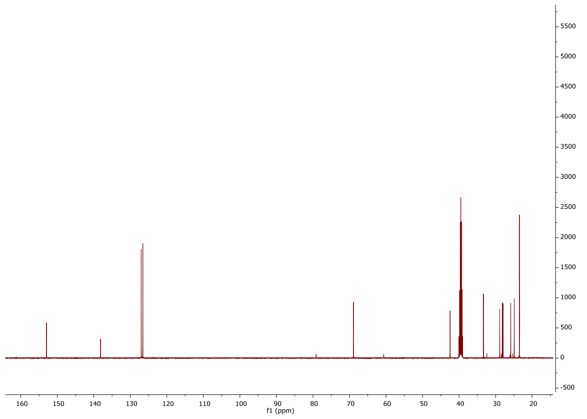

Compound 39a

^{1}H NMR (500 MHz, DMSO-d6); ^{13}C NMR (126 MHz, DMSO-d6)

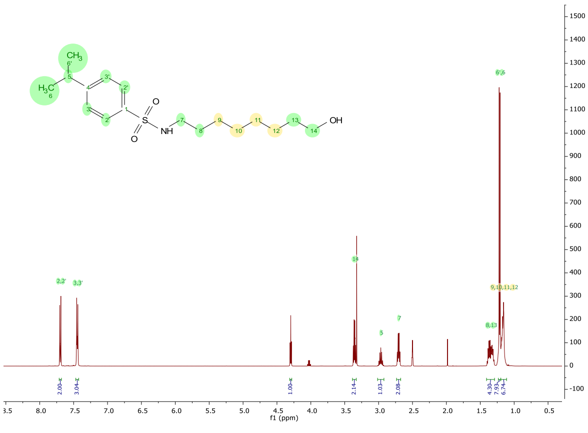

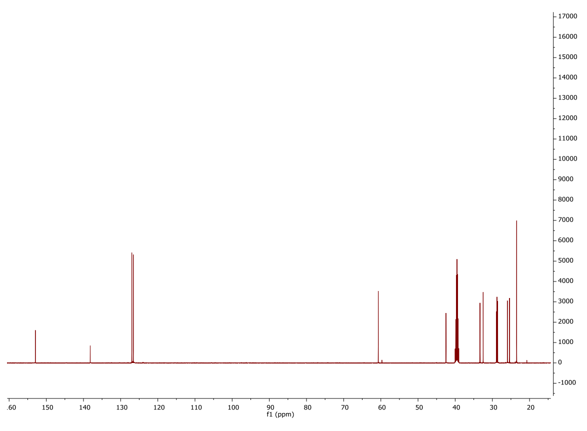

Compound 39b

^{1}H NMR (400 MHz, DMSO-d6); ^{13}C NMR (126 MHz, DMSO-d6)

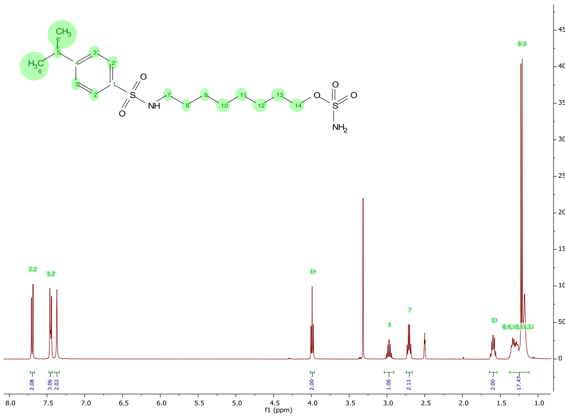

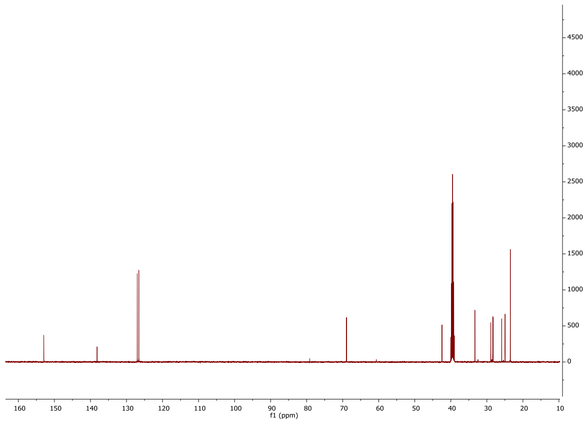

Compound 40a

^{1}H NMR (400 MHz, DMSO-d6); ^{13}C NMR (126 MHz, DMSO-d6)

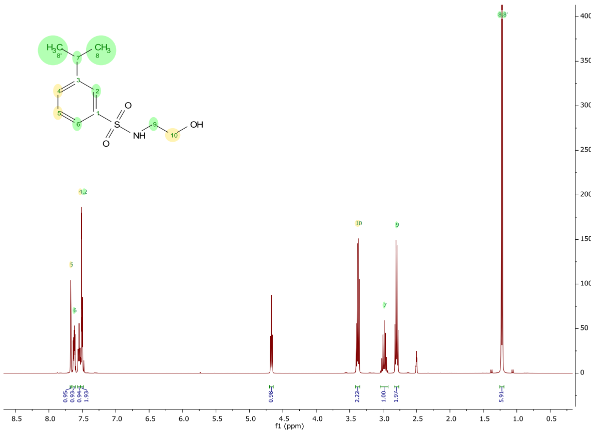

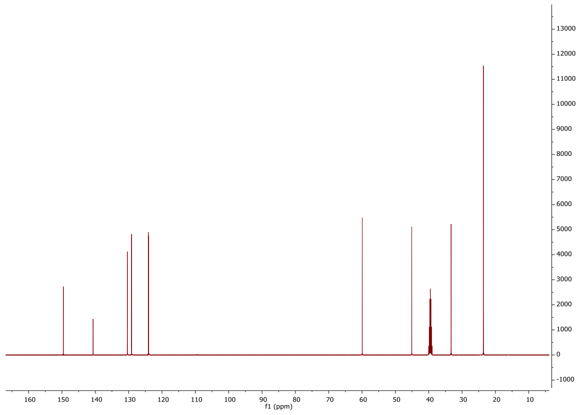

Compound 40b

^{1}H NMR (400 MHz, DMSO-d6); ^{13}C NMR (126 MHz, DMSO-d6)

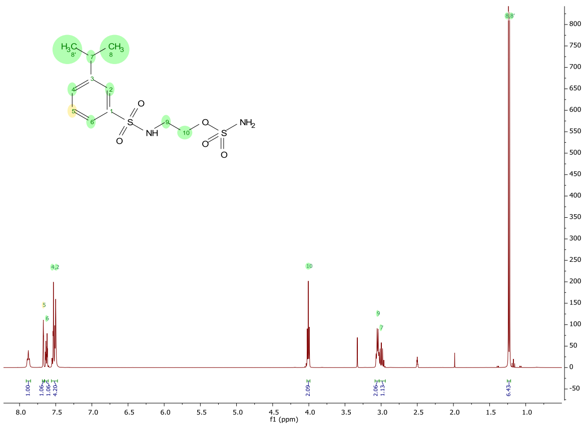

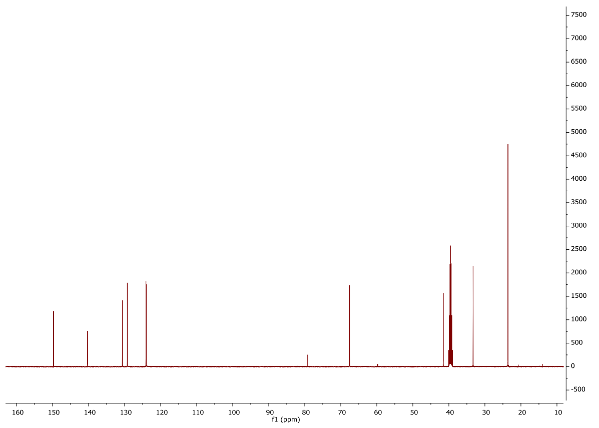

Compound 41a

^{1}H NMR (400 MHz, DMSO-d6); ^{13}C NMR (101 MHz, DMSO-d6)

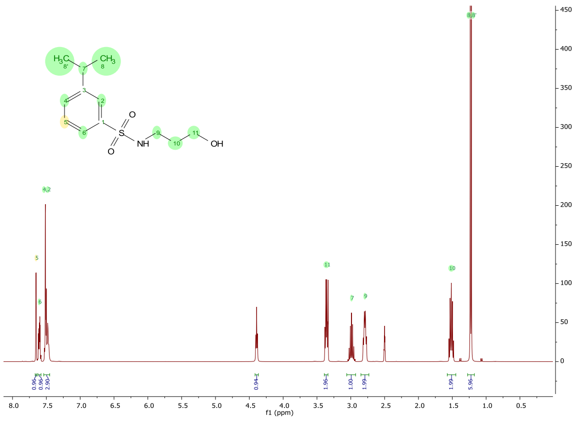

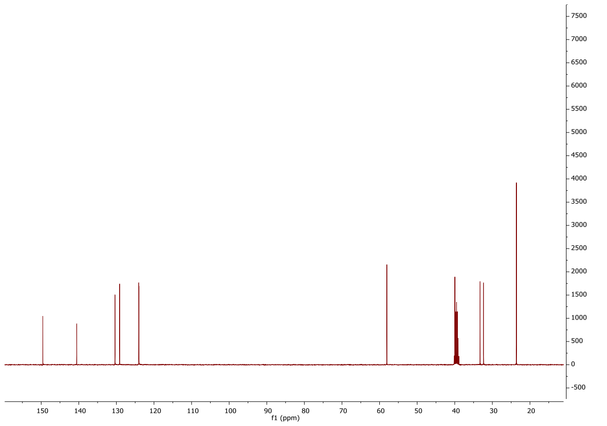

Compound 41b

^{1}H NMR (400 MHz, DMSO-d6); ^{13}C NMR (126 MHz, DMSO-d6)

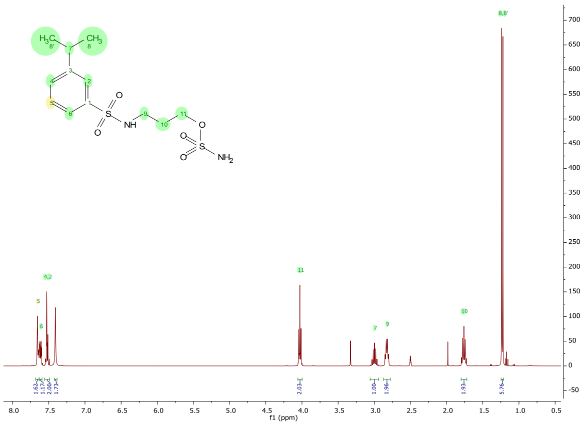

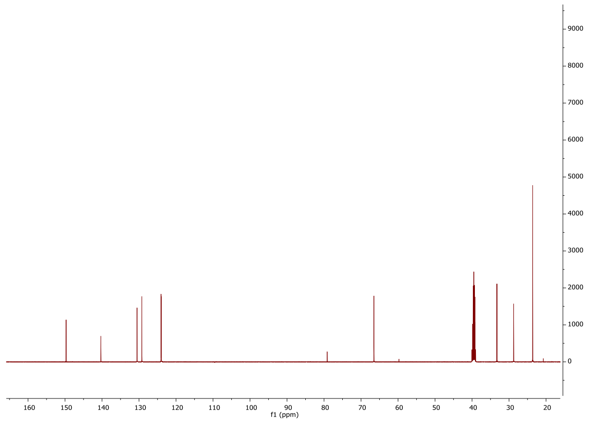

Compound 42a

^{1}H NMR (400 MHz, DMSO-d6); ^{13}C NMR (126 MHz, DMSO-d6)

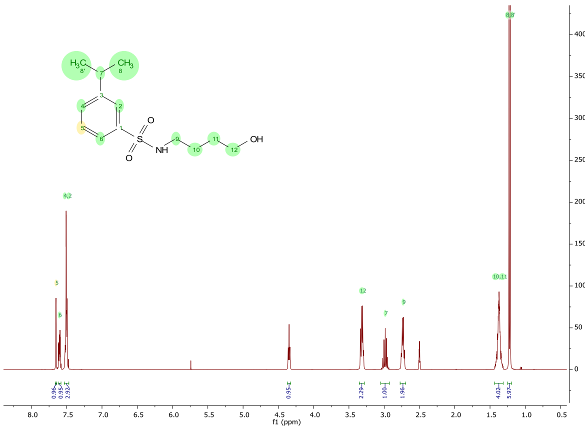

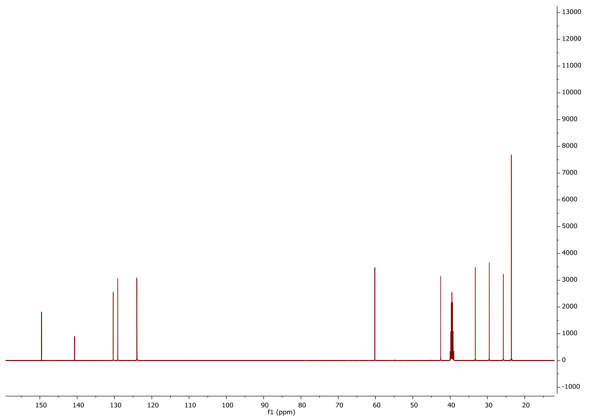

Compound 42b

^{1}H NMR (400 MHz, DMSO-d6); ^{13}C NMR (126 MHz, DMSO-d6)

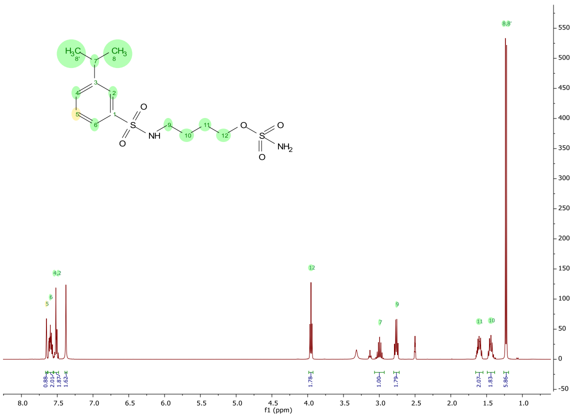

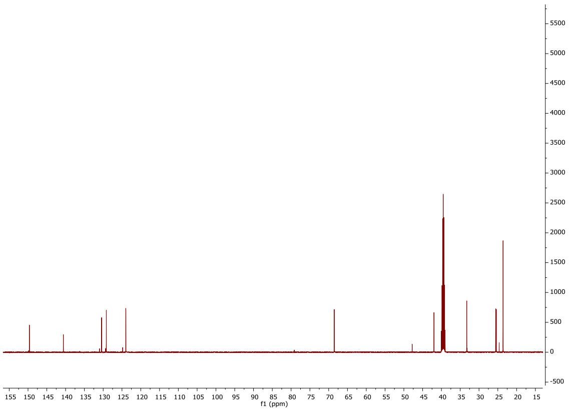

Compound 43a

^{1}H NMR (400 MHz, DMSO-d6); ^{13}C NMR (101 MHz, DMSO-d6)

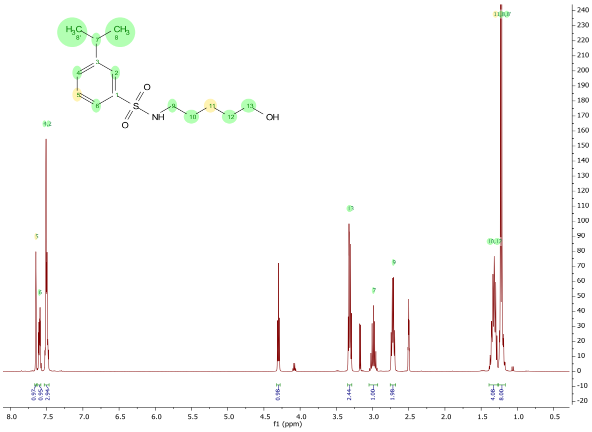

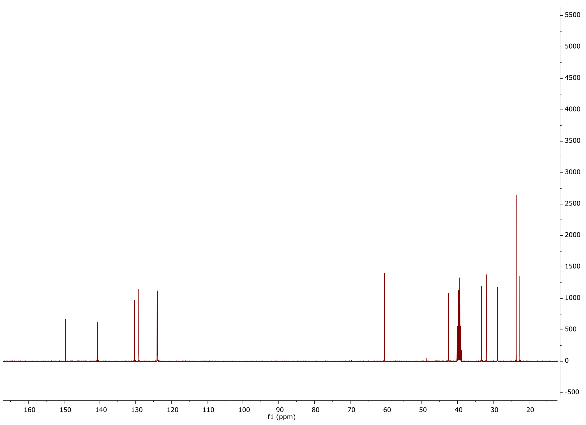

Compound 43b

^{1}H NMR (400 MHz, DMSO-d6); ^{13}C NMR (101 MHz, DMSO-d6)

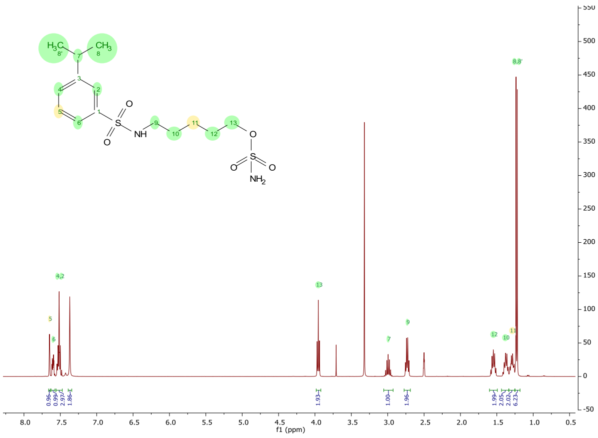

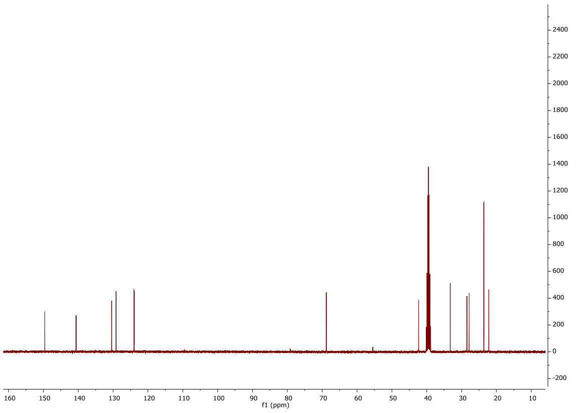

Compound 44a

^1H NMR (400 MHz, DMSO-d6); ^{13}C NMR (101 MHz, DMSO-d6)

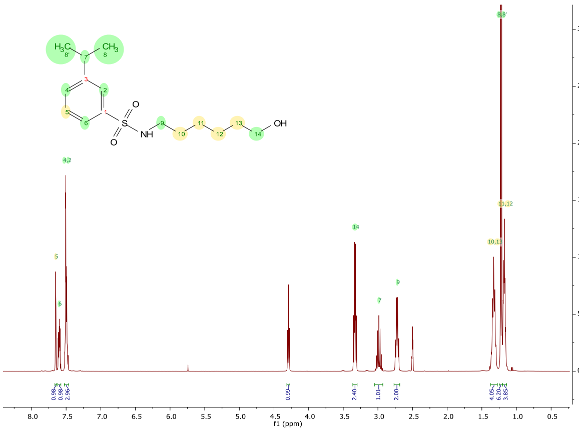

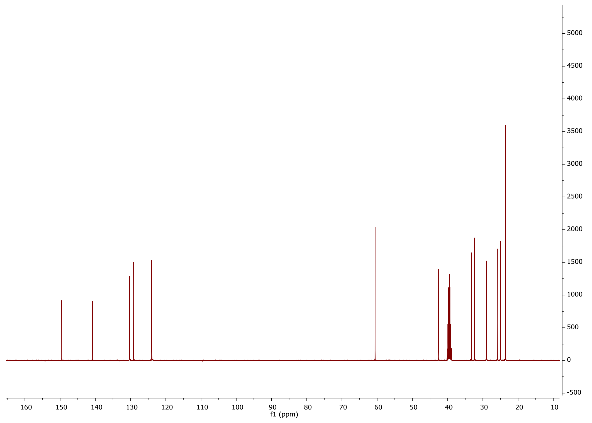

Compound 44b

^1H NMR (400 MHz, DMSO-d6); ^{13}C NMR (101 MHz, DMSO-d6)

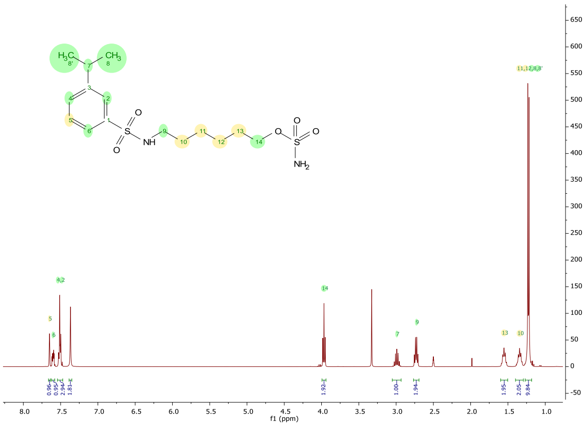

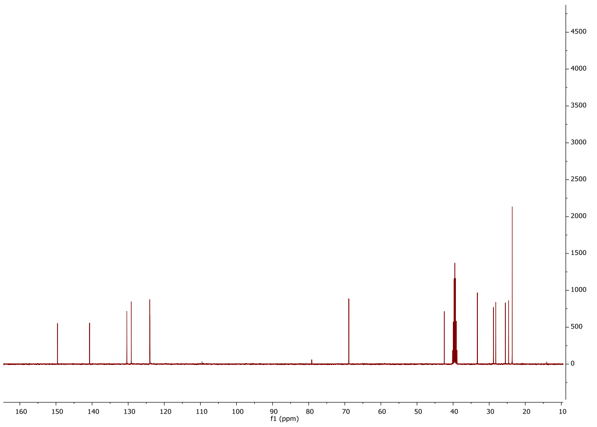

Compound 45a

^1H NMR (400 MHz, DMSO-d6); ^{13}C APT-NMR (101 MHz, DMSO-d6)

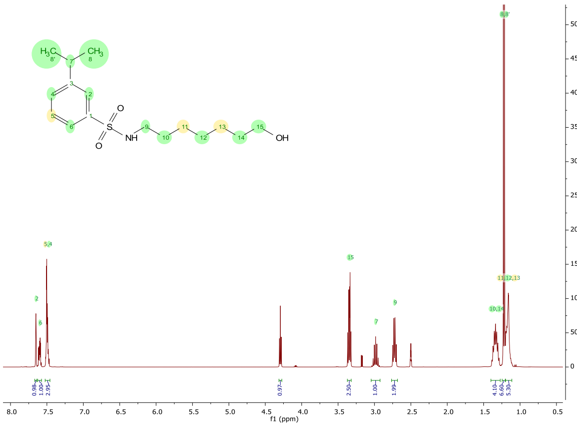

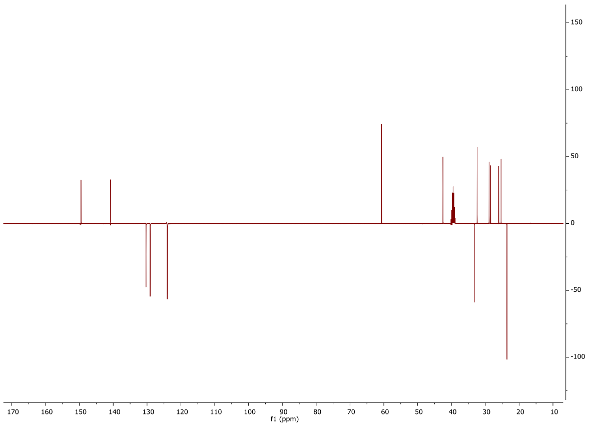

Compound 45b

^{1}H NMR (400 MHz, DMSO-d6); ^{13}C NMR (101 MHz, DMSO-d6)

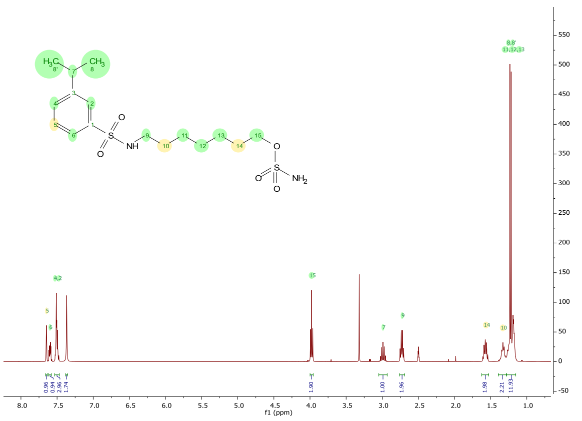

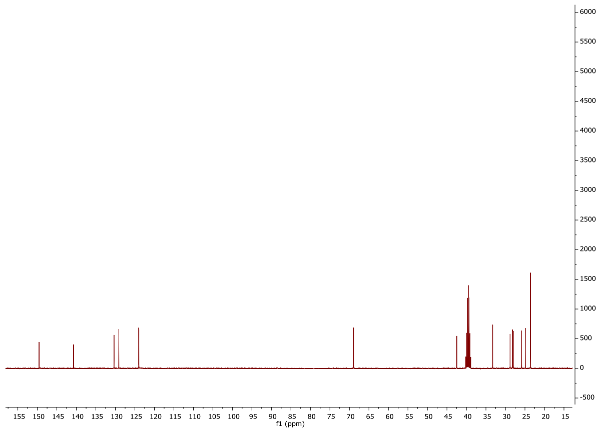

Compound 46a

^{1}H NMR (500 MHz, DMSO-d6); ^{13}C NMR (126 MHz, DMSO-d6)

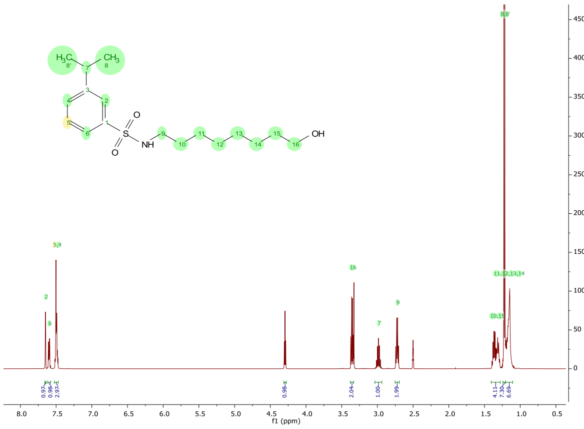

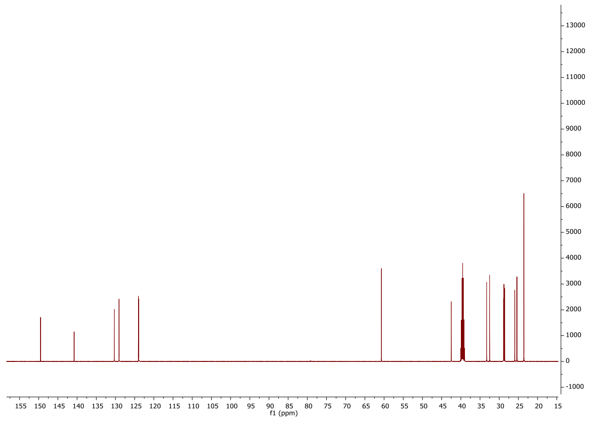

Compound 46b

^{1}H NMR (400 MHz, DMSO-d6); ^{13}C NMR (101 MHz, DMSO-d6)

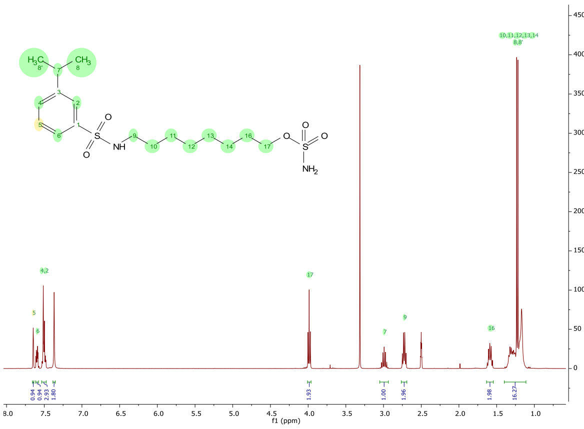

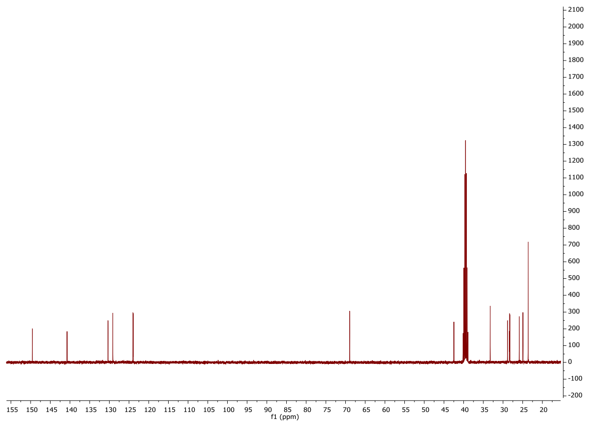

Compound 47a

^{1}H NMR (400 MHz, DMSO-d6); ^{13}C APT-NMR (101 MHz, DMSO-d6)

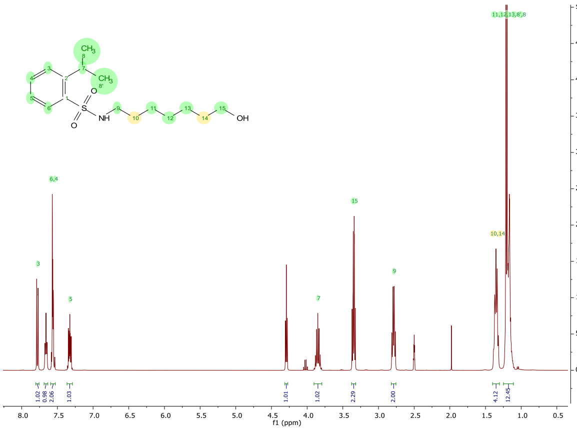

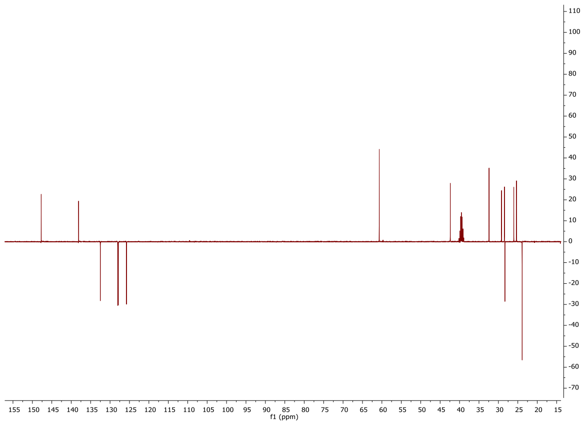

Compound 47b

^{1}H NMR (400 MHz, DMSO-d6); ^{13}C NMR (101 MHz, DMSO-d6)

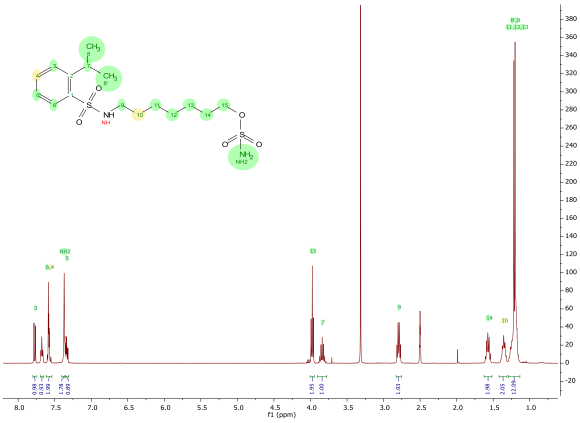

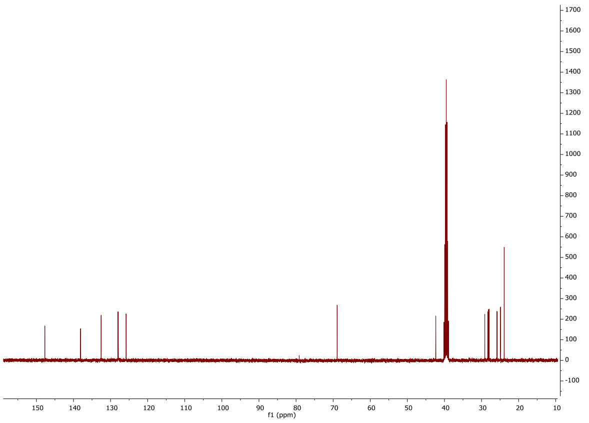

Compound 48a

^{1}H NMR (400 MHz, DMSO-d6); ^{13}C APT-NMR (101 MHz, DMSO-d6)

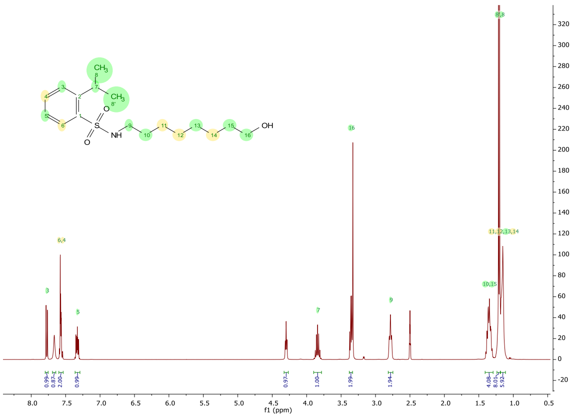

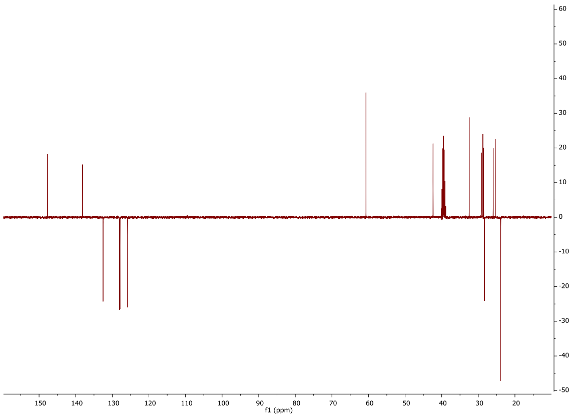

Compound 48b

^{1}H NMR (400 MHz, DMSO-d6); ^{13}C NMR (126 MHz, DMSO-d6)

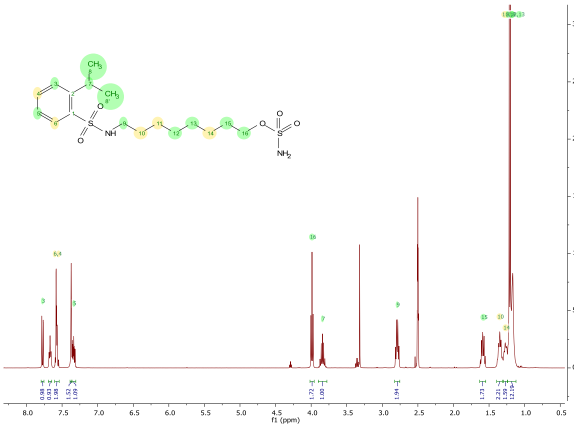

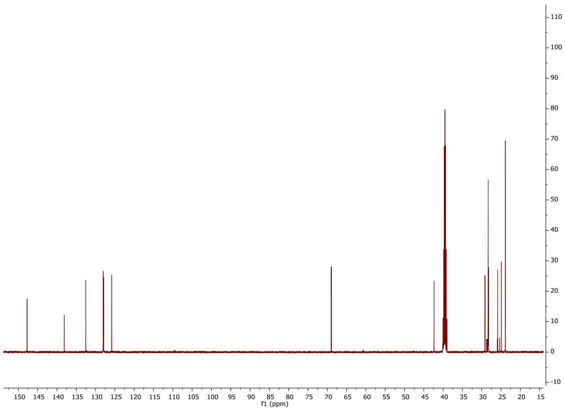

Compound 49a

^{1}H NMR (400 MHz, DMSO-d6); ^{13}C NMR (101 MHz, DMSO-d6)

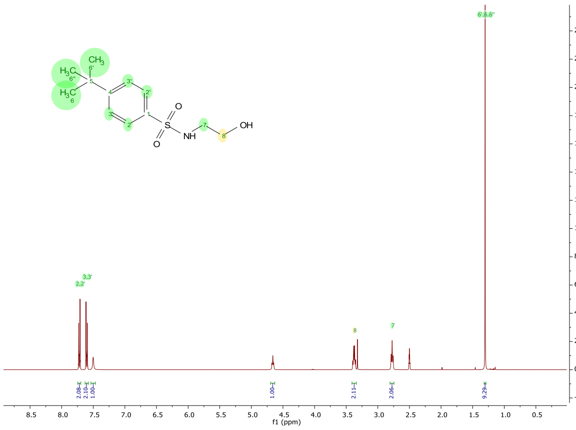

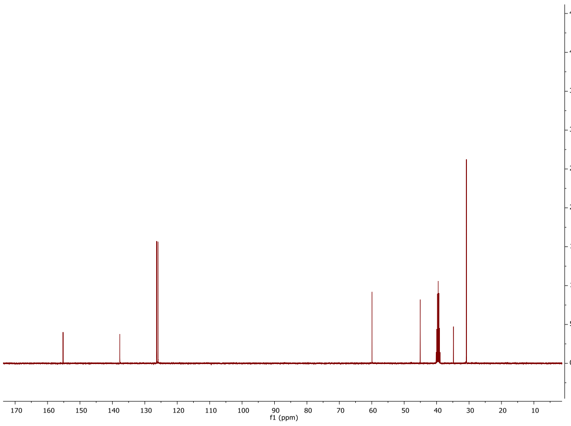

Compound 49b

^{1}H NMR (400 MHz, DMSO-d6); ^{13}C NMR (126 MHz, DMSO-d6)

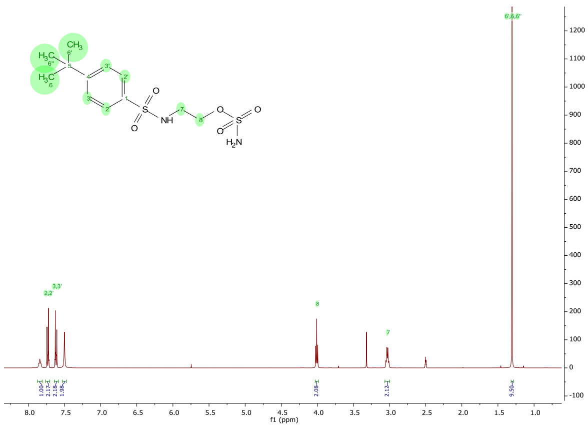

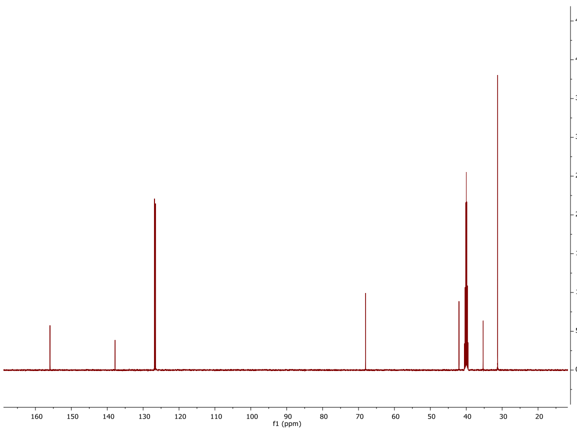

Compound 50a

^{1}H NMR (400 MHz, DMSO-d6); ^{13}C NMR (101 MHz, DMSO-d6)

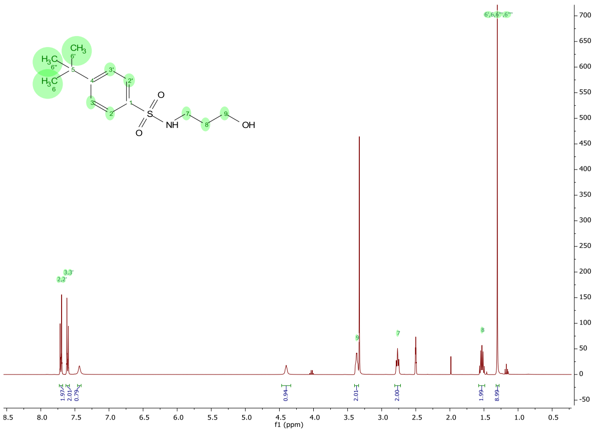

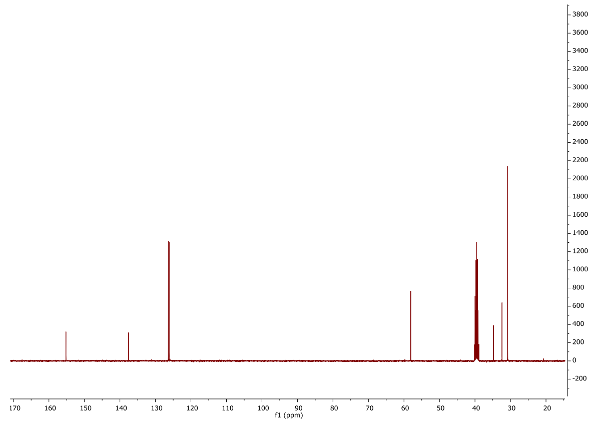

Compound 50b

^{1}H NMR (400 MHz, DMSO-d6); ^{13}C NMR (126 MHz, DMSO-d6)

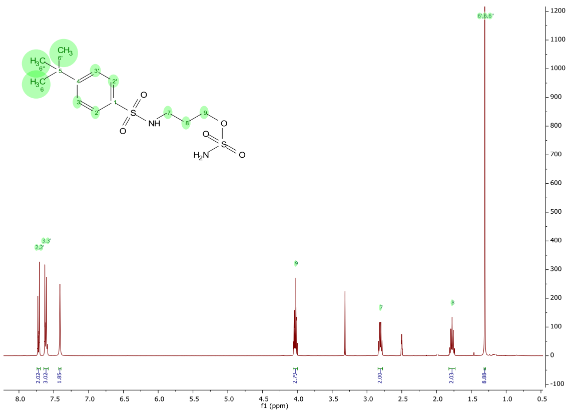

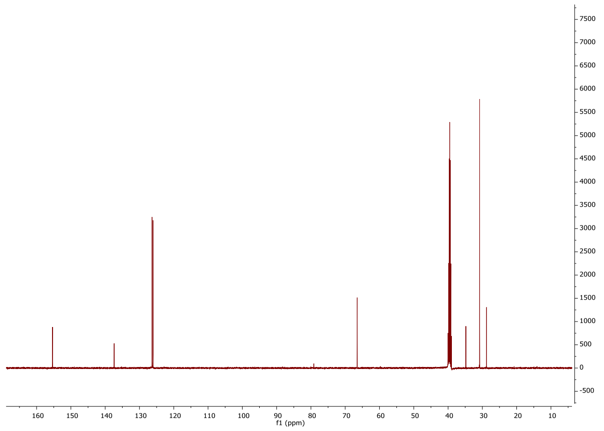

Compound 51a

^{1}H NMR (400 MHz, DMSO-d6); ^{13}C NMR (101 MHz, DMSO-d6)

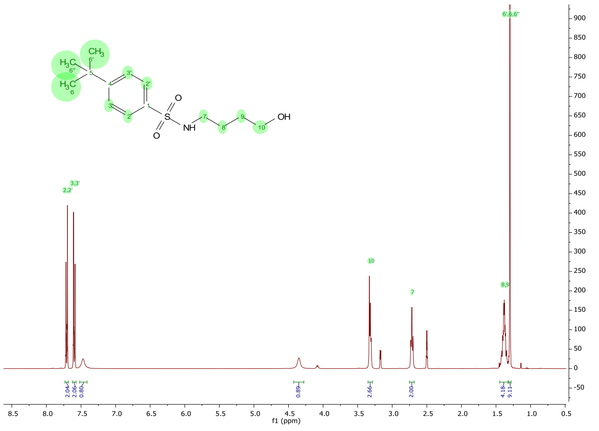

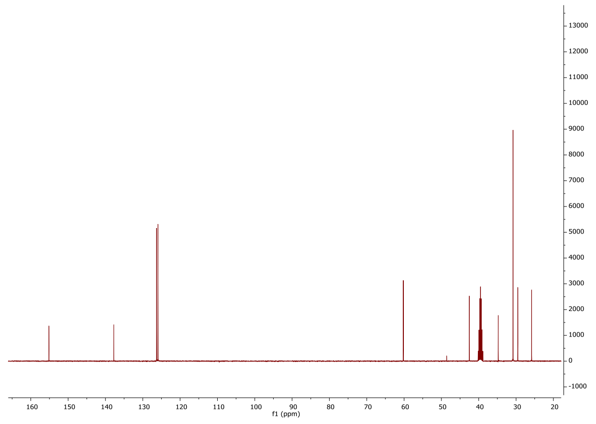

Compound 51b

^{1}H NMR (400 MHz, DMSO-d6); ^{13}C NMR (126 MHz, DMSO-d6)

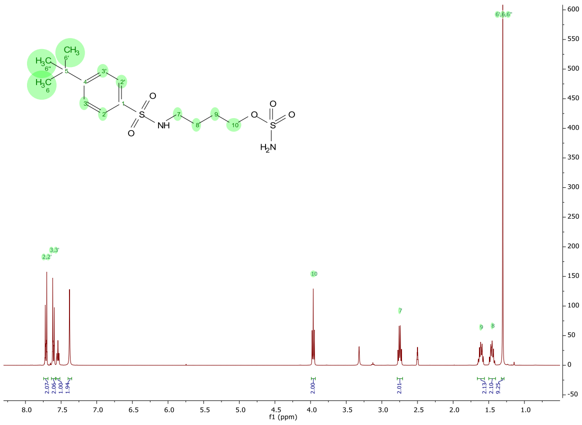

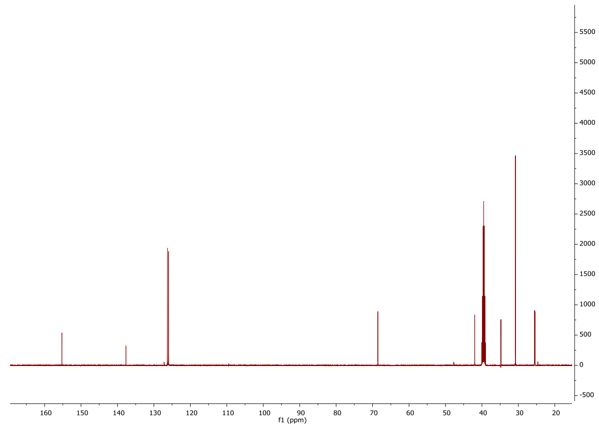

Compound 52a

^{1}H NMR (500 MHz, DMSO-d6); ^{13}C NMR (101 MHz, DMSO-d6)

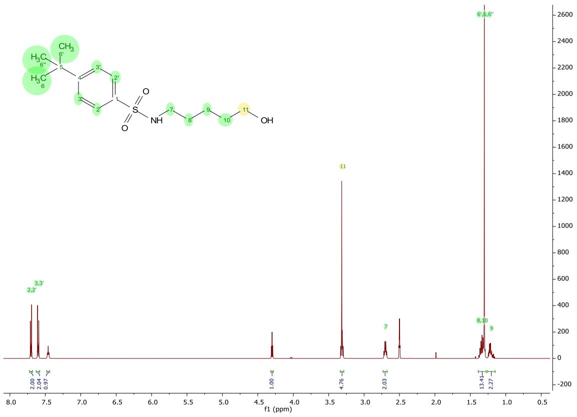

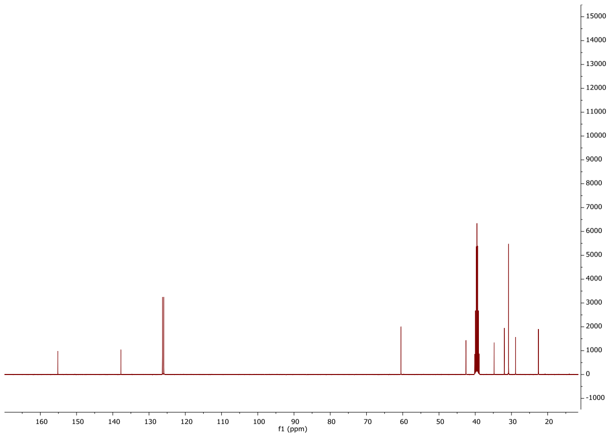

Compound 52b

^{1}H NMR (400 MHz, DMSO-d6); ^{13}C NMR (101 MHz, DMSO-d6)

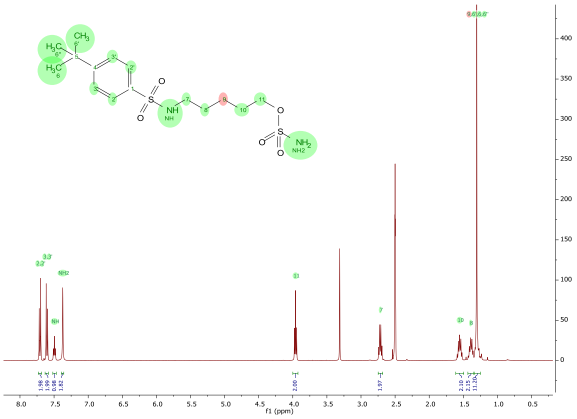

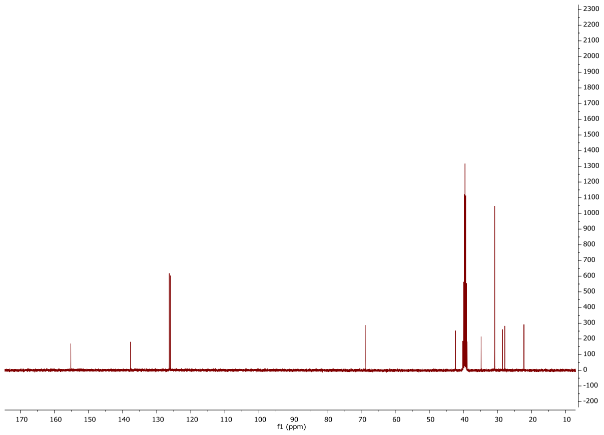

Compound 53a

^{1}H NMR (500 MHz, DMSO-d6); ^{13}C NMR (101 MHz, DMSO-d6)

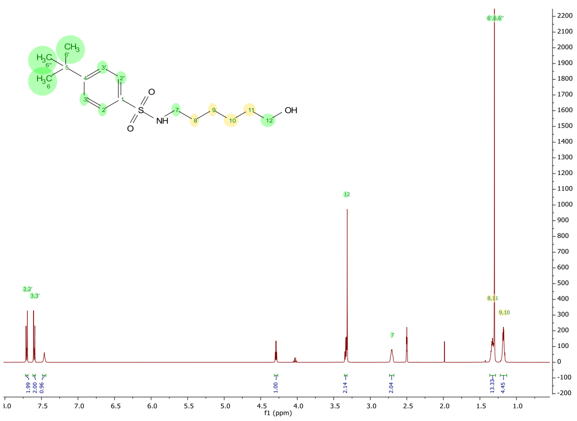

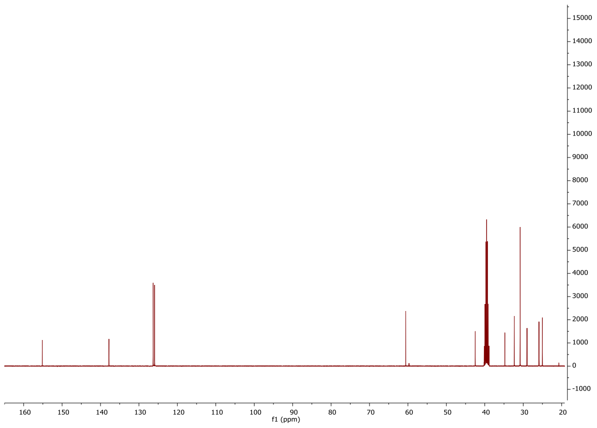

Compound 53b

^{1}H NMR (400 MHz, DMSO-d6); ^{13}C NMR (126 MHz, DMSO-d6)

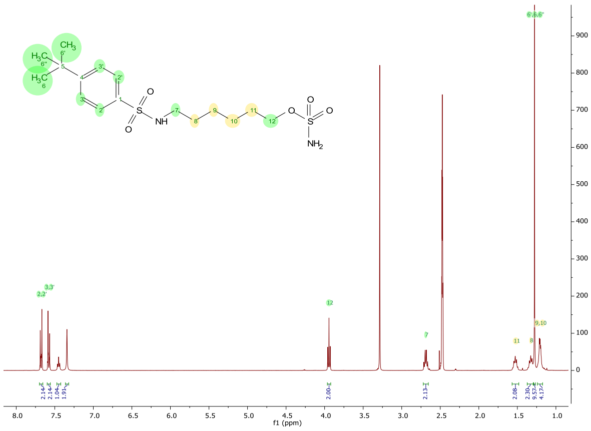

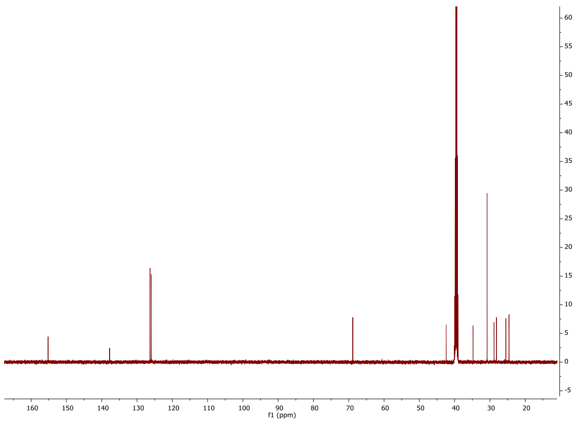

Compound 54a

^{1}H NMR (400 MHz, DMSO-d6); ^{13}C NMR (126 MHz, DMSO-d6)

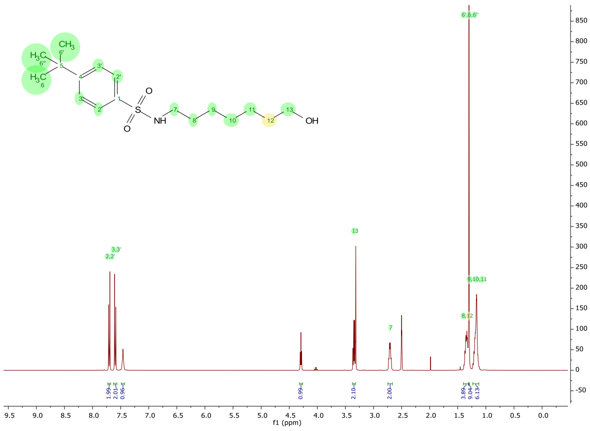

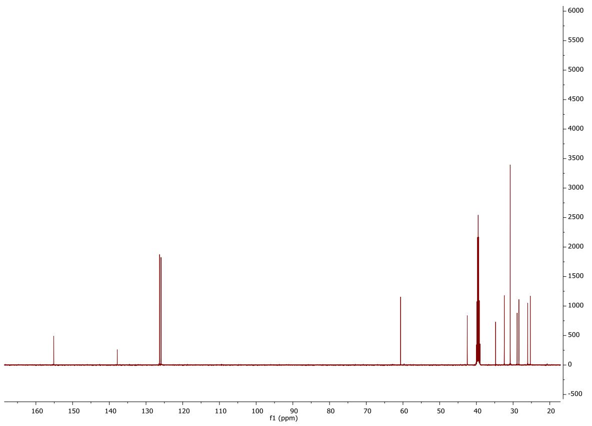

Compound 54b

^{1}H NMR (400 MHz, DMSO-d6); ^{13}C NMR (126 MHz, DMSO-d6)

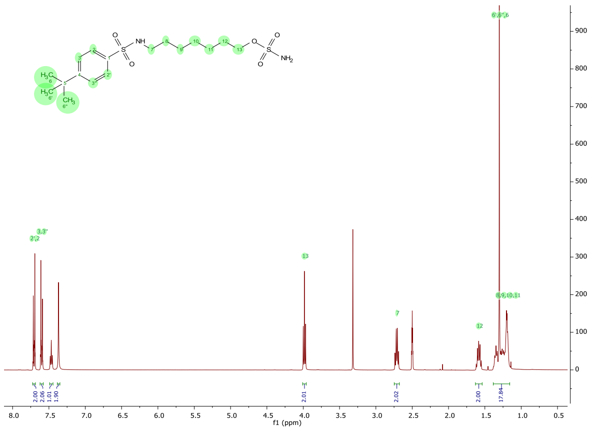

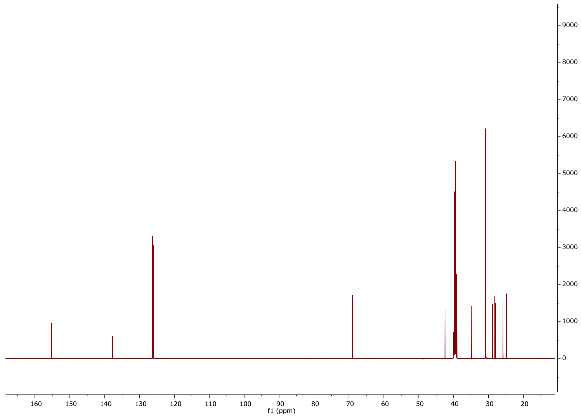

Compound 55a

^{1}H NMR (400 MHz, DMSO-d6); ^{13}C NMR (126 MHz, DMSO-d6)

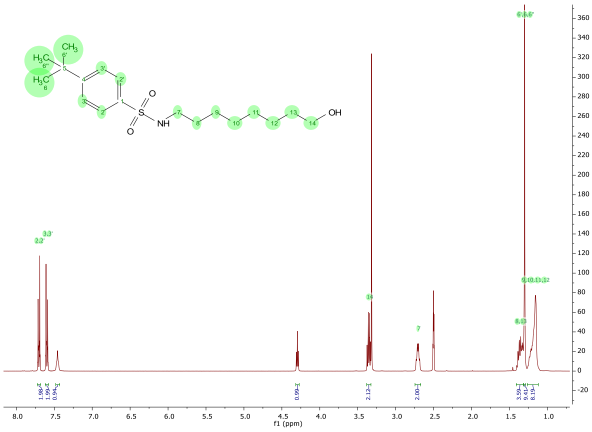

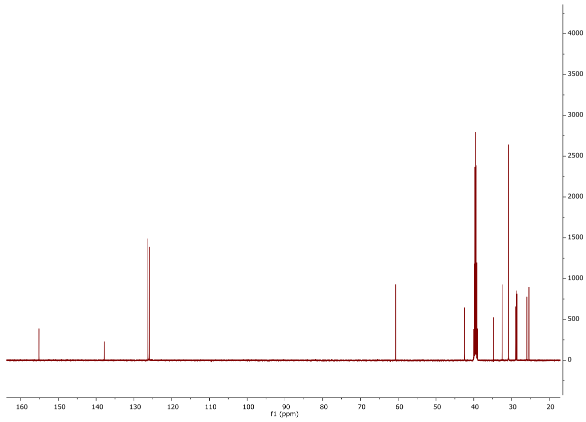

Compound 55b

^{1}H NMR (400 MHz, DMSO-d6); ^{13}C NMR (101 MHz, DMSO-d6)

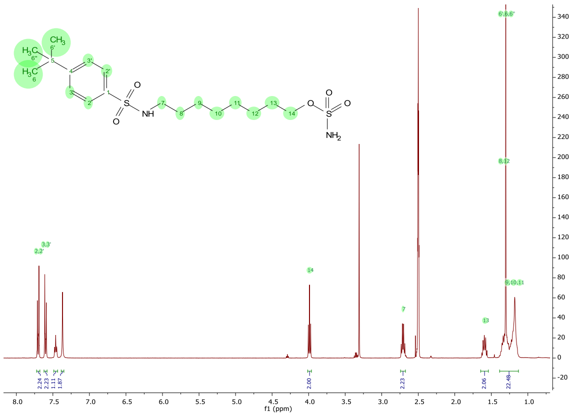

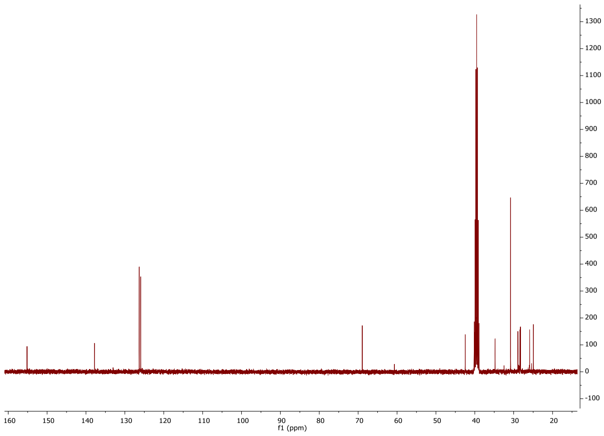

Compound 56a

^{1}H NMR (400 MHz, DMSO-d6); ^{13}C NMR (101 MHz, DMSO-d6)

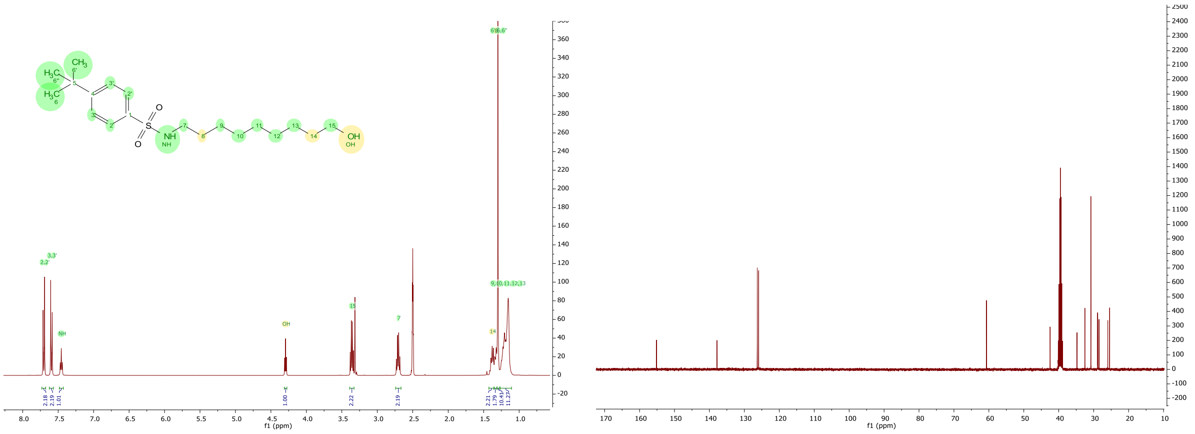

Compound 56b

^{1}H NMR (400 MHz, DMSO-d6); ^{13}C NMR (101 MHz, DMSO-d6)

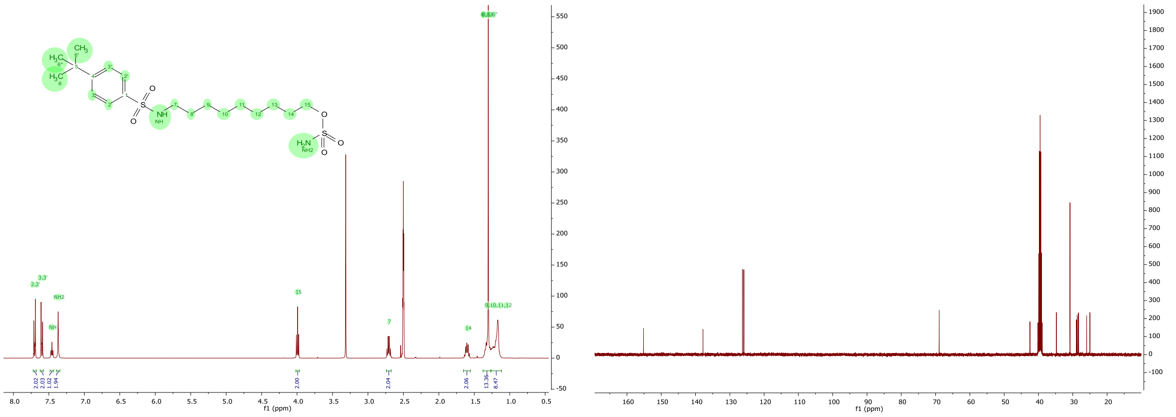

Compound 57a

^{1}H NMR (400 MHz, DMSO-d6); ^{13}C NMR (101 MHz, DMSO-d6)

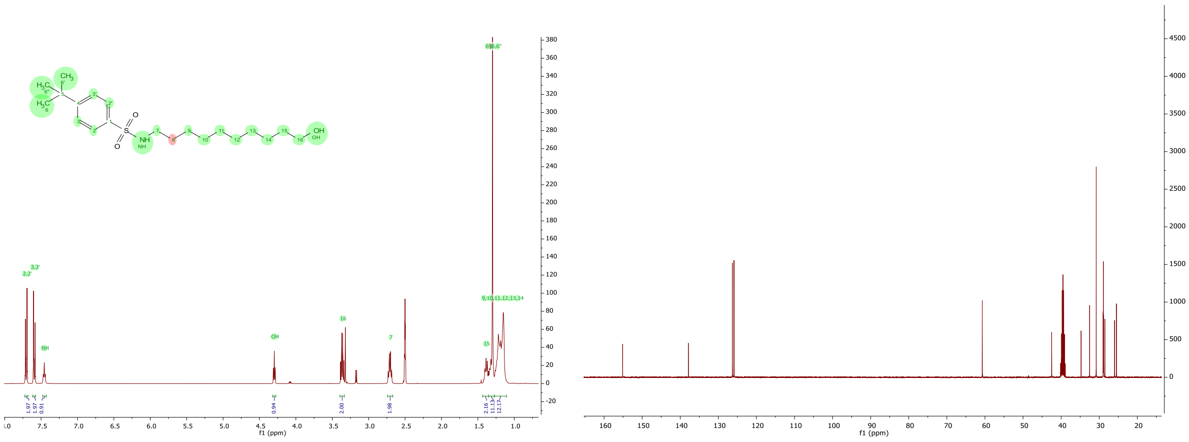

Compound 57b

^{1}H NMR (400 MHz, DMSO-d6); ^{13}C NMR (101 MHz, DMSO-d6)

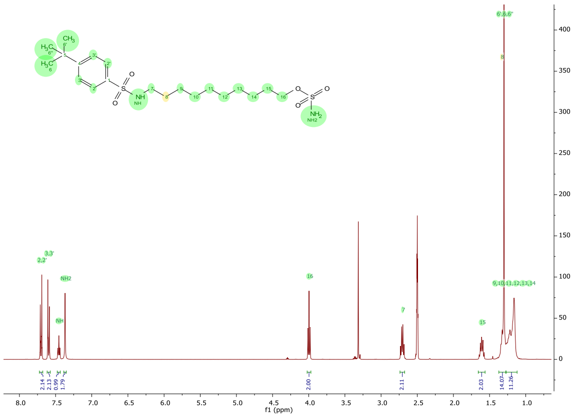

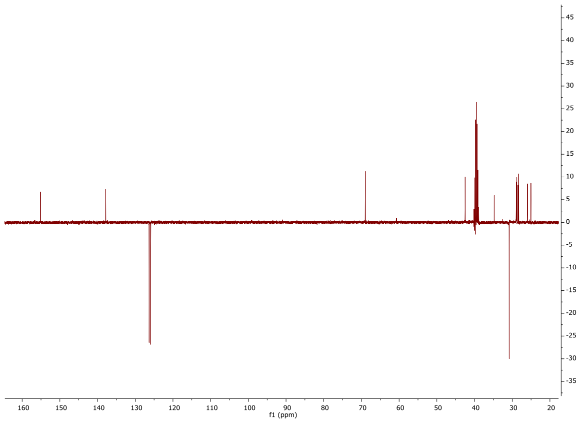

Compound 58a

^{1}H NMR (400 MHz, DMSO-d6); ^{13}C NMR (101 MHz, DMSO-d6)

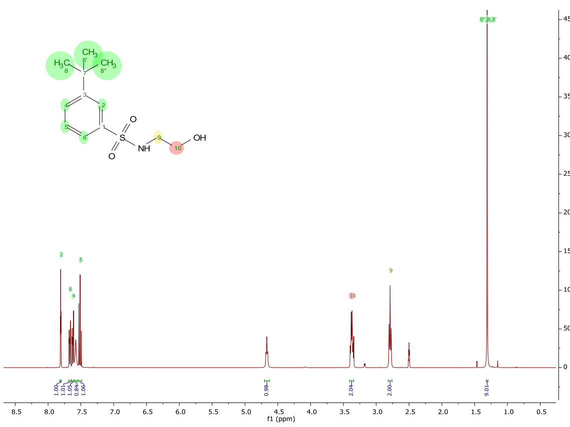

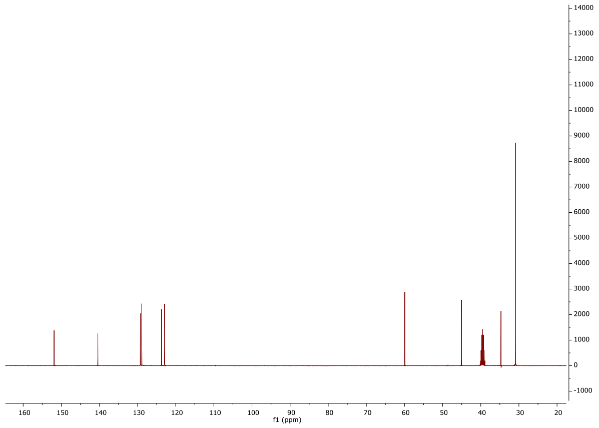

Compound 58b

^{1}H NMR (400 MHz, DMSO-d6); ^{13}C NMR (101 MHz, DMSO-d6)

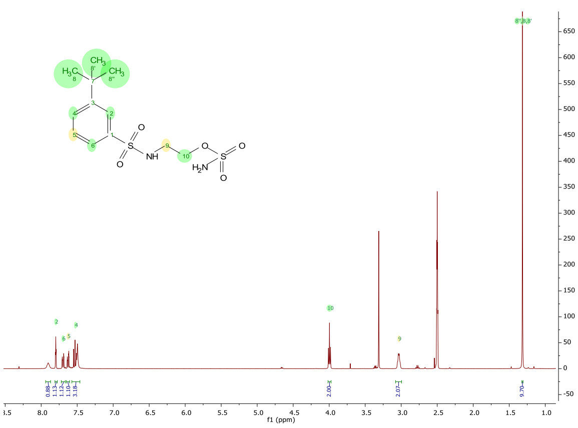

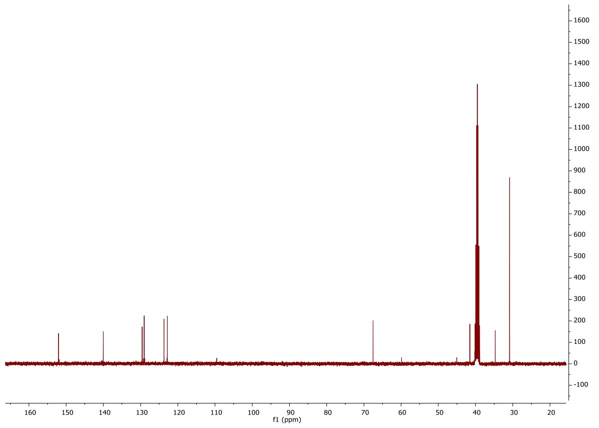

Compound 59a

^{1}H NMR (400 MHz, DMSO-d6); ^{13}C NMR (101 MHz, DMSO-d6)

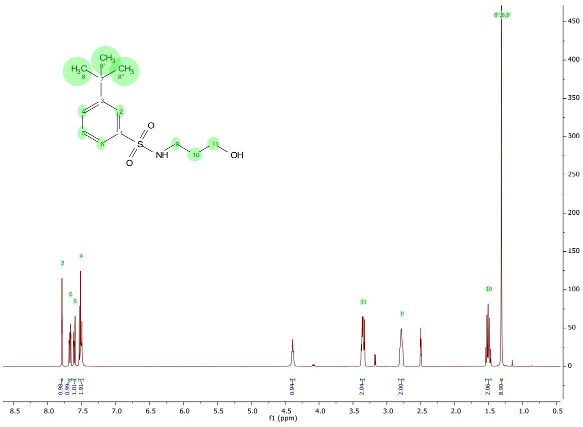

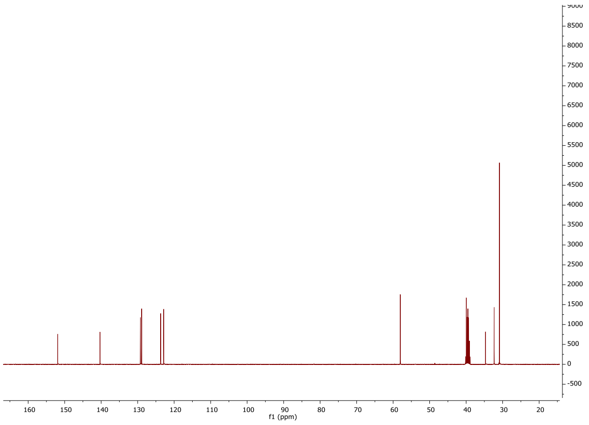

Compound 59b

^{1}H NMR (400 MHz, DMSO-d6); ^{13}C NMR (101 MHz, DMSO-d6)

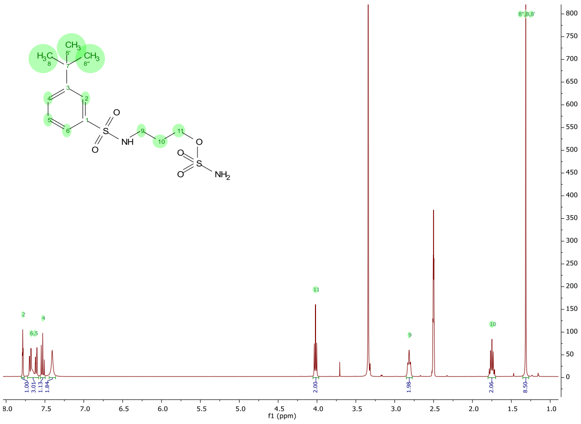

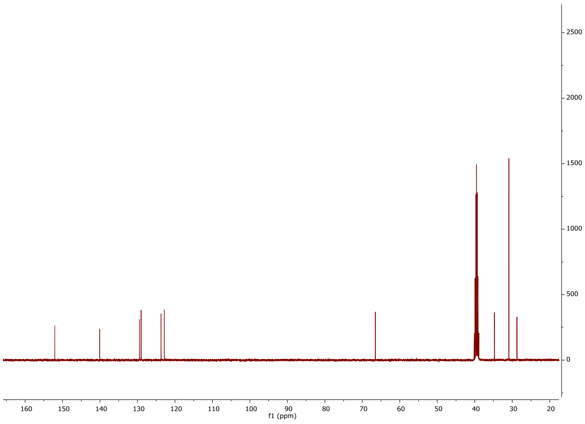

Compound 60a

^{1}H NMR (400 MHz, DMSO-d6); ^{13}C NMR (101 MHz, DMSO-d6)

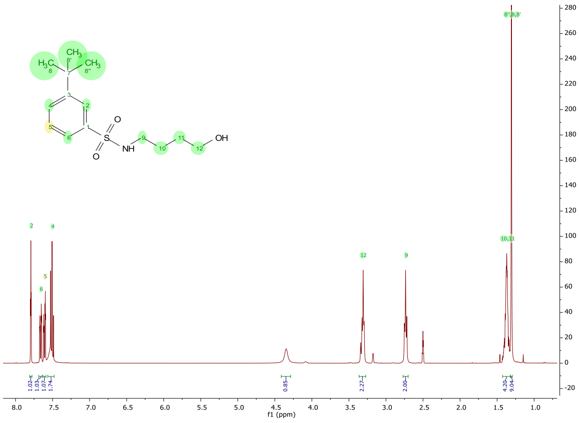

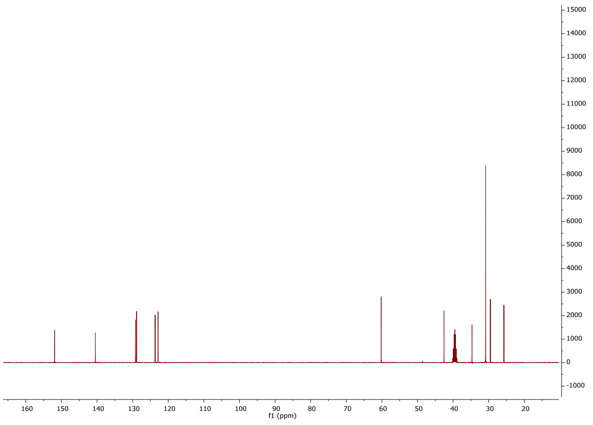

Compound 60b

^{1}H NMR (400 MHz, DMSO-d6); ^{13}C NMR (101 MHz, DMSO-d6)

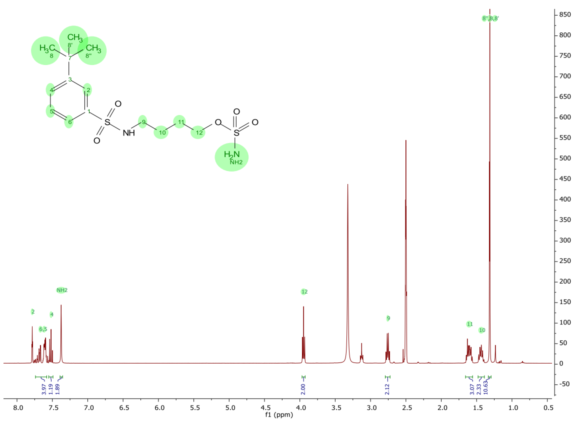

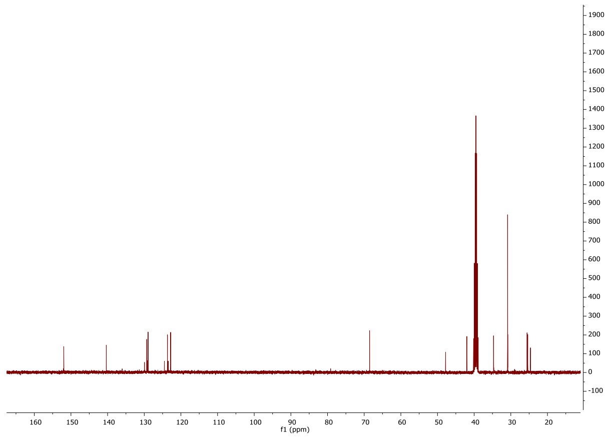

Compound 61a

^{1}H NMR (400 MHz, DMSO-d6); ^{13}C NMR (126 MHz, DMSO-d6)

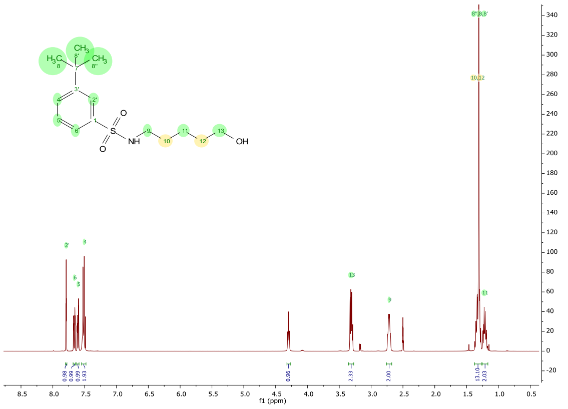

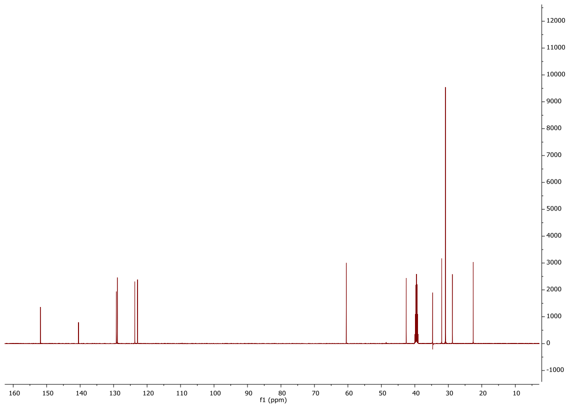

Compound 61b

^{1}H NMR (400 MHz, DMSO-d6); ^{13}C NMR (101 MHz, DMSO-d6)

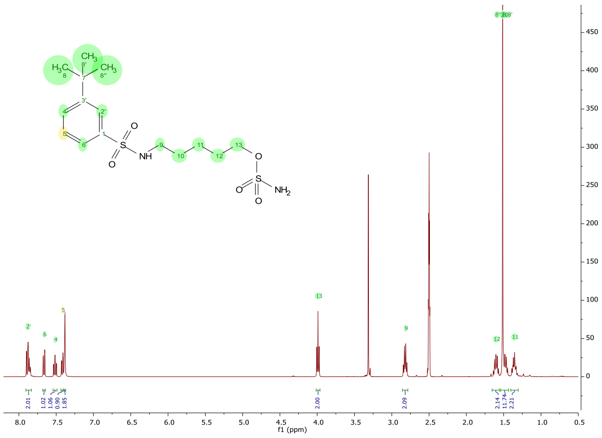

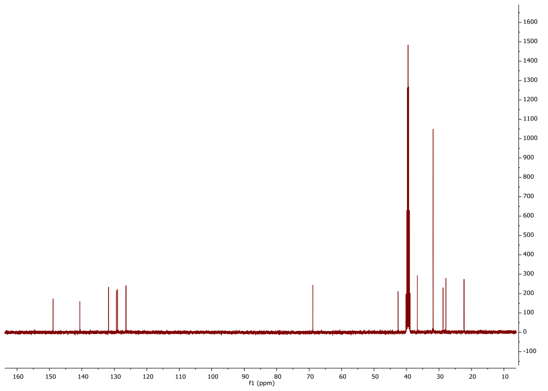

Compound 62a

^1H NMR (400 MHz, DMSO-d6); ^{13}C NMR (101 MHz, DMSO-d6)

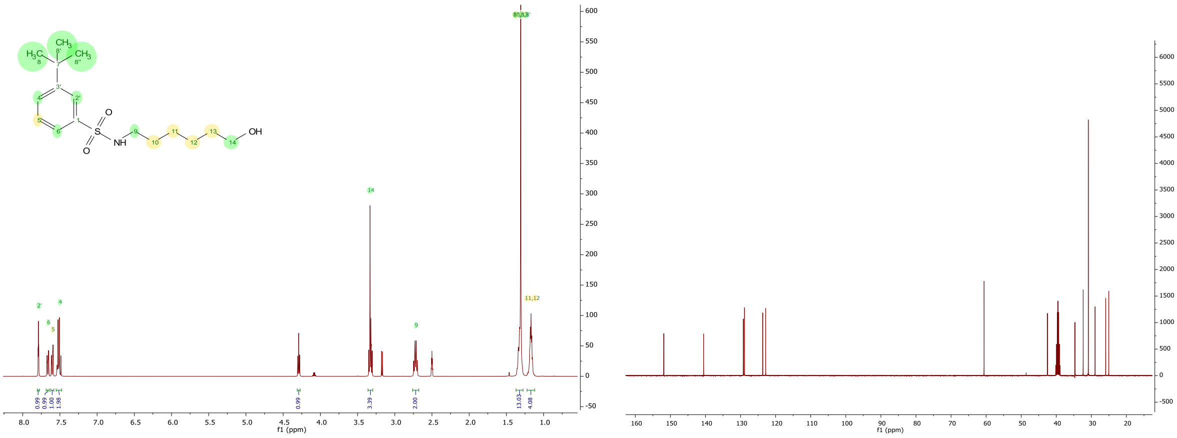

Compound 62b

^1H NMR (400 MHz, DMSO-d6); ^{13}C NMR (126 MHz, DMSO-d6)

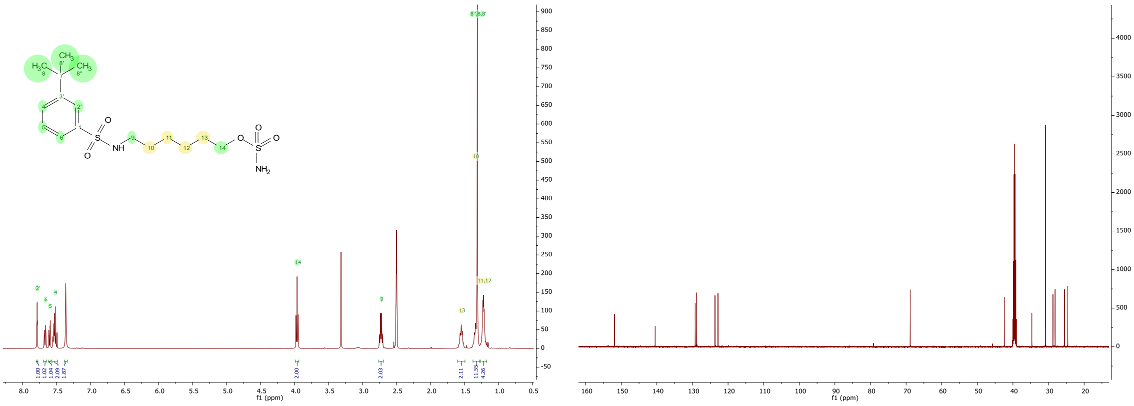

Compound 63a

^1H NMR (400 MHz, DMSO-d6); ^{13}C NMR (126 MHz, DMSO-d6)

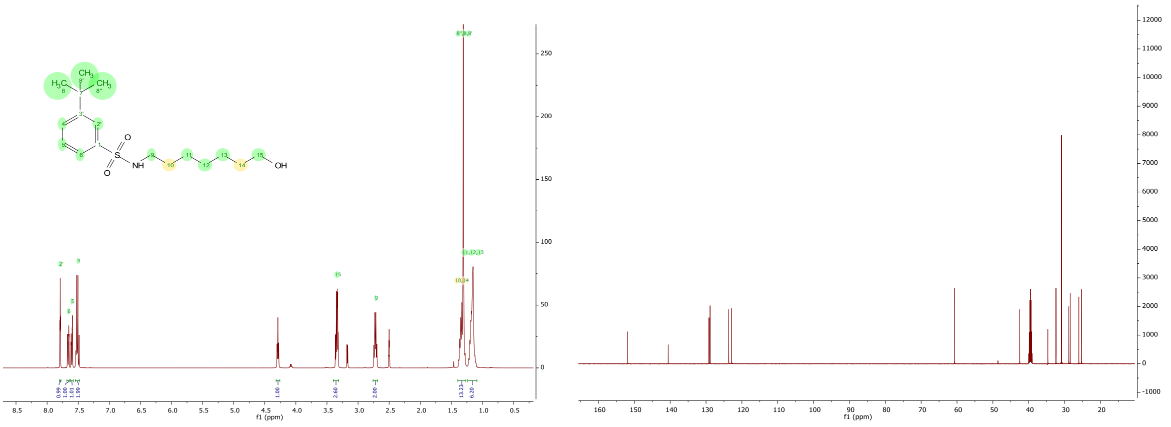

Compound 63b

^{1}H NMR (400 MHz, DMSO-d6); ^{13}C NMR (101 MHz, DMSO-d6)

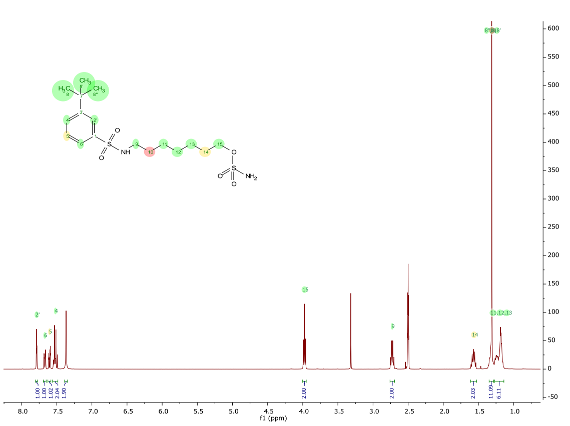

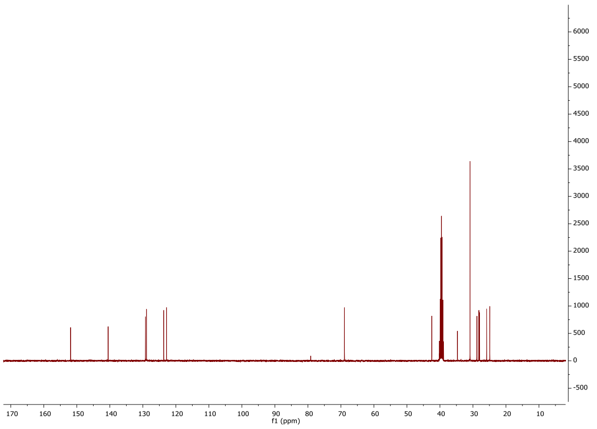

Compound 64a

^{1}H NMR (400 MHz, DMSO-d6); ^{13}C NMR (101 MHz, DMSO-d6)

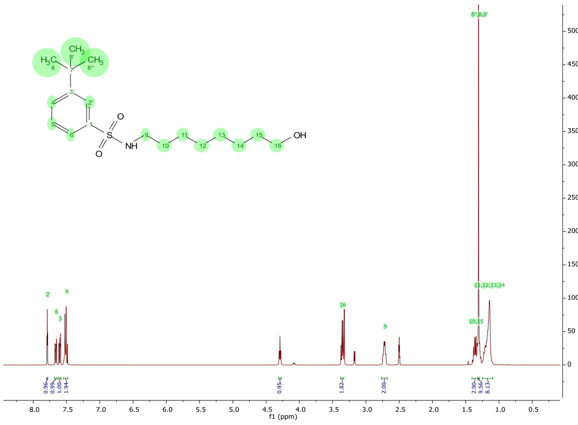

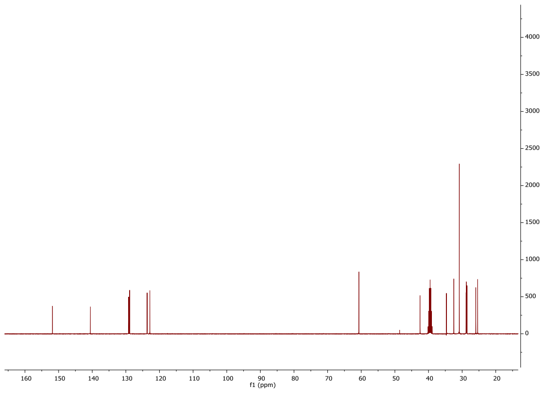

Compound 64b

^{1}H NMR (400 MHz, DMSO-d6); ^{13}C NMR (126 MHz, DMSO-d6)

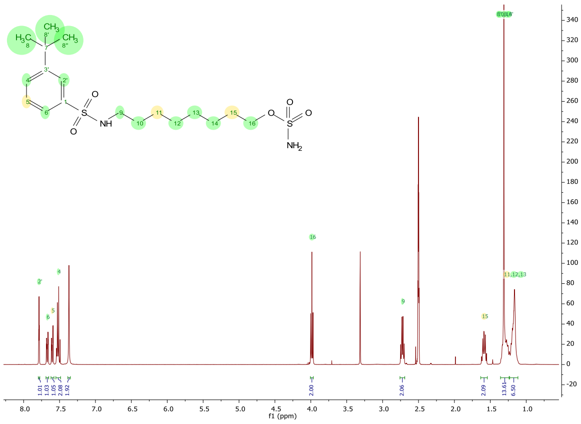

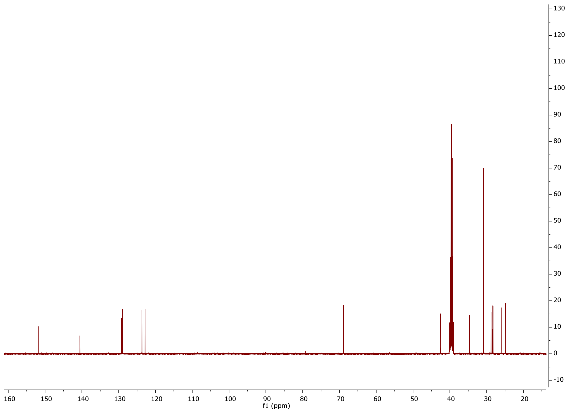

Compound 65a

^{1}H NMR (500 MHz, DMSO-d6); ^{13}C APT-NMR (126 MHz, DMSO-d6)

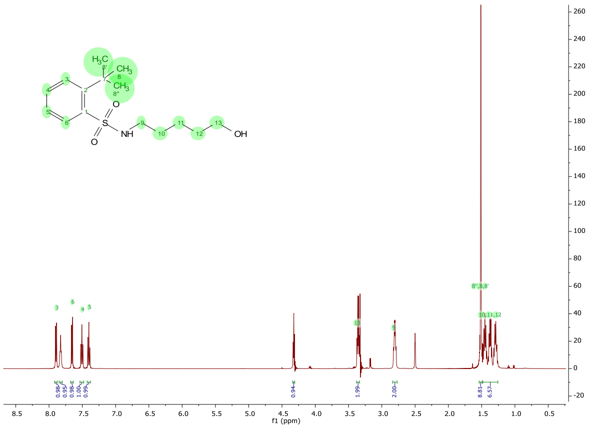

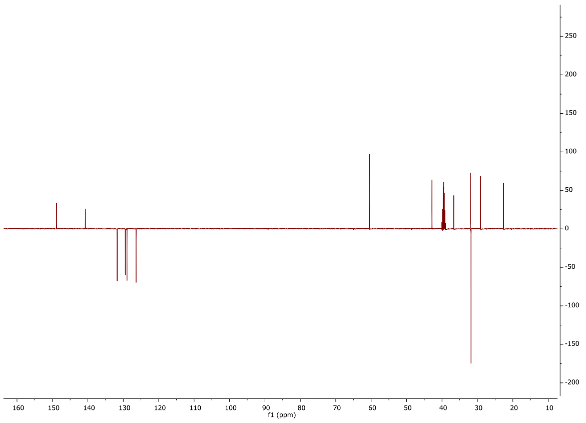

Compound 65b

^{1}H NMR (400 MHz, DMSO-d6); ^{13}C NMR (101 MHz, DMSO-d6)

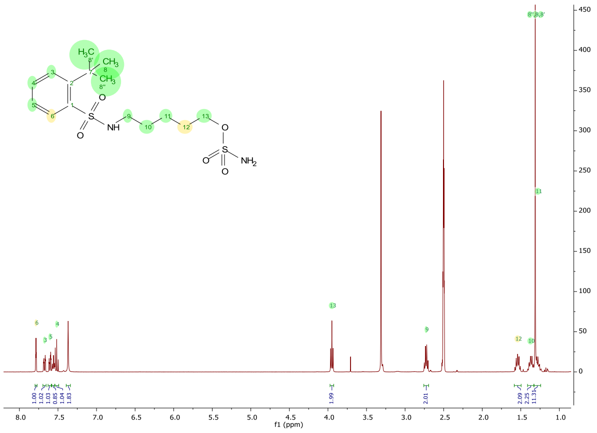

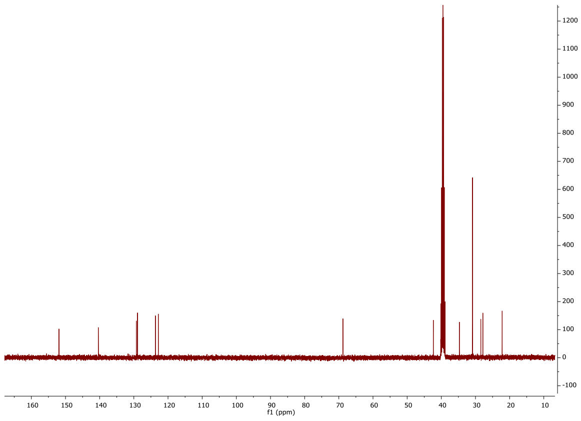

Compound 66a

^{1}H NMR (400 MHz, DMSO-d6); ^{13}C NMR (126 MHz, DMSO-d6)

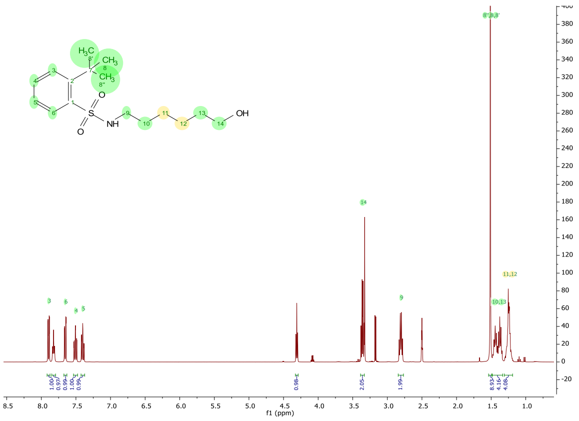

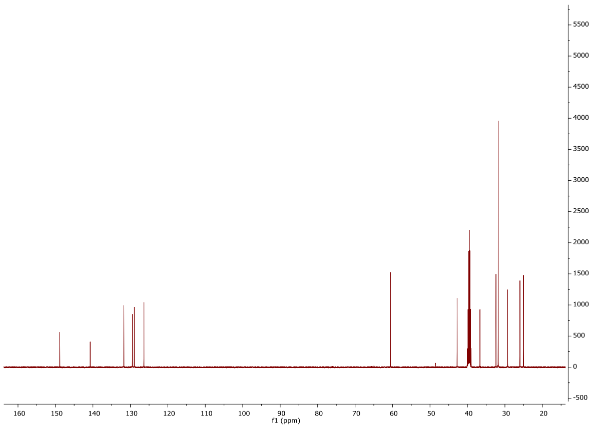

Compound 66b

^{1}H NMR (400 MHz, DMSO-d6); ^{13}C NMR (101 MHz, DMSO-d6)

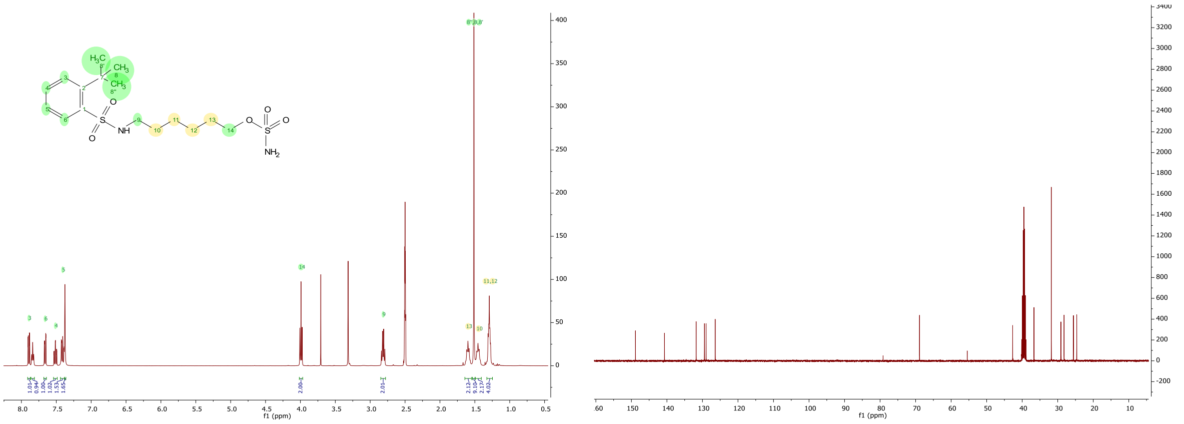

Compound 67a

^{1}H NMR (400 MHz, DMSO-d6); ^{13}C NMR (126 MHz, DMSO-d6)

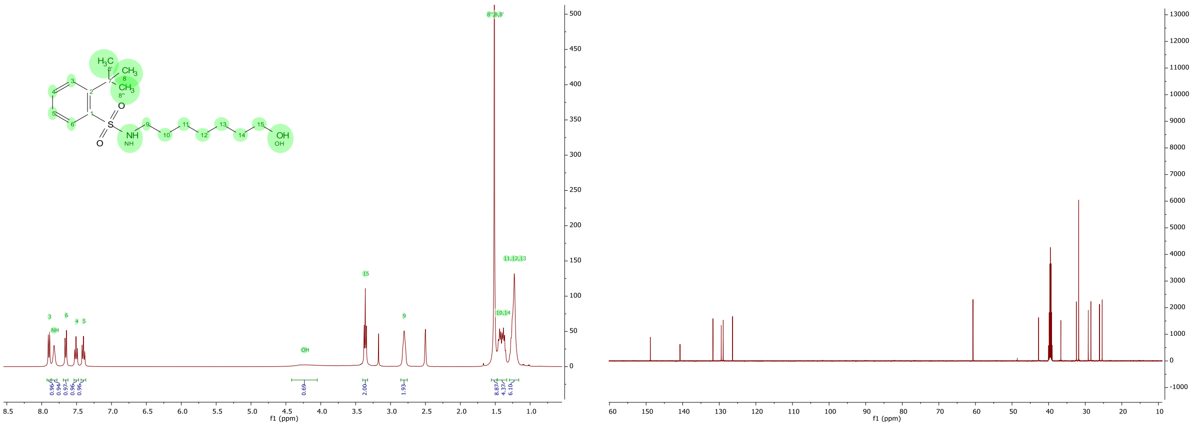

Compound 67b

^{1}H NMR (400 MHz, DMSO-d6); ^{13}C NMR (101 MHz, DMSO-d6)

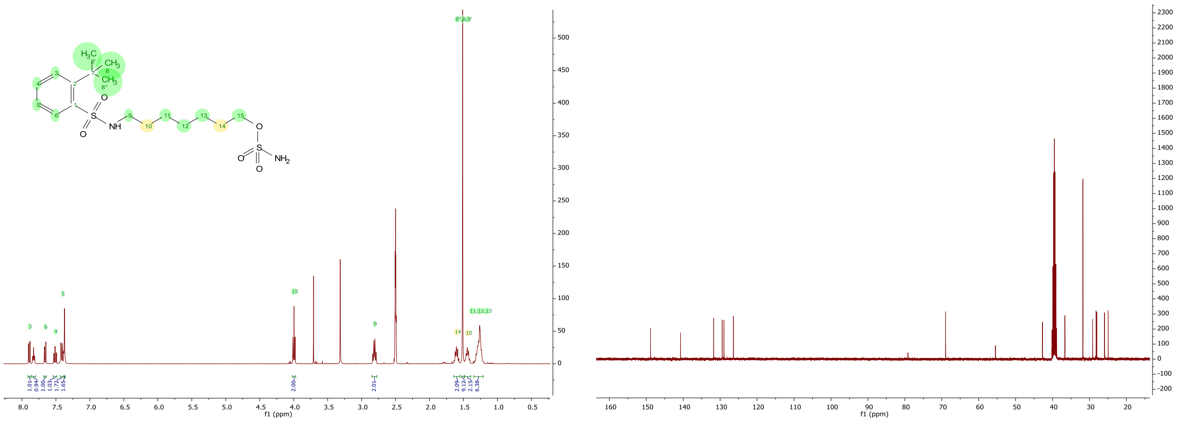

Compound 68a

^{1}H NMR (400 MHz, DMSO-d6); ^{13}C NMR (101 MHz, DMSO-d6)

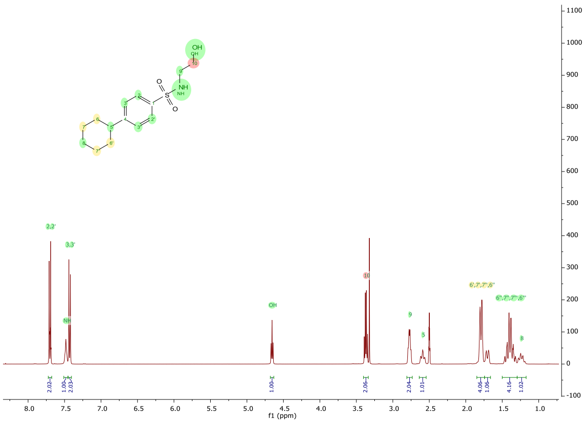

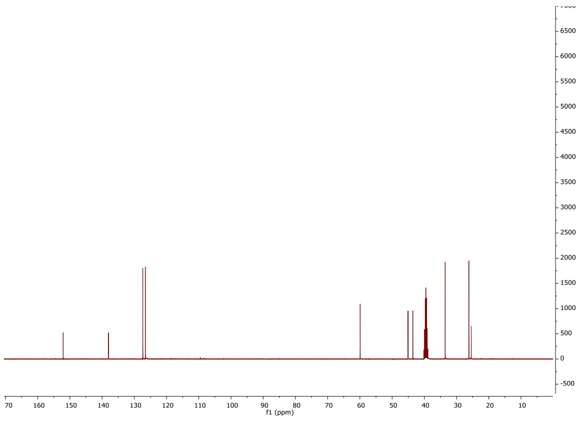

Compound 68b

^{1}H NMR (400 MHz, DMSO-d6); ^{13}C NMR (101 MHz, DMSO-d6)

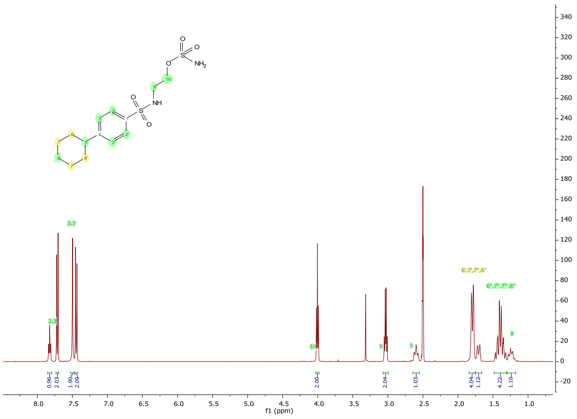

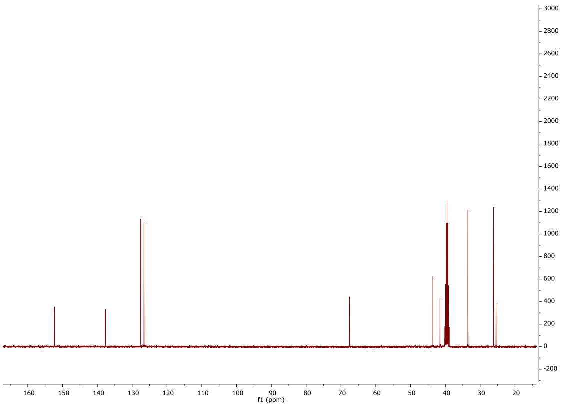

Compound 69a

^{1}H NMR (400 MHz, DMSO-d6); ^{13}C NMR (101 MHz, DMSO-d6)

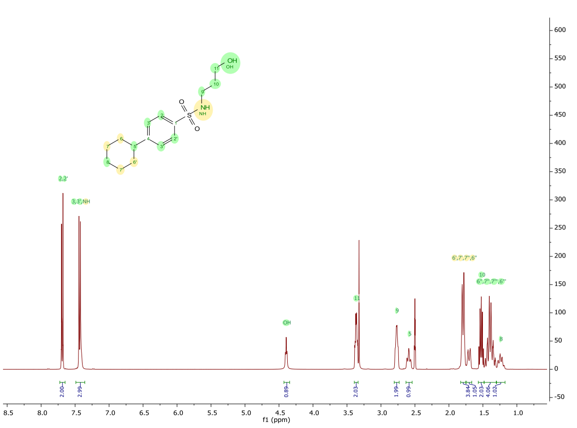

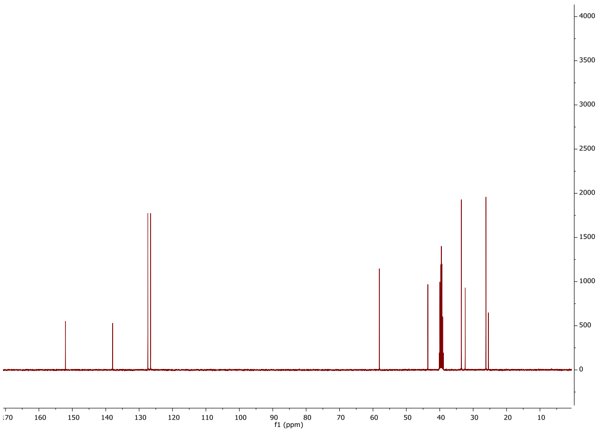

Compound 69b

^{1}H NMR (400 MHz, DMSO-d6); ^{13}C NMR (101 MHz, DMSO-d6)

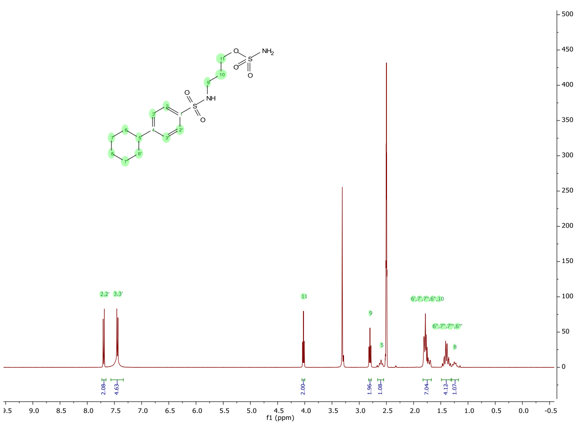

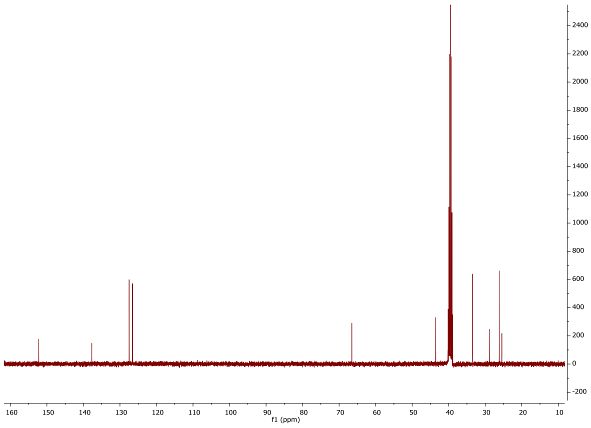

Compound 70a

^{1}H NMR (400 MHz, DMSO-d6); ^{13}C NMR (101 MHz, DMSO-d6)

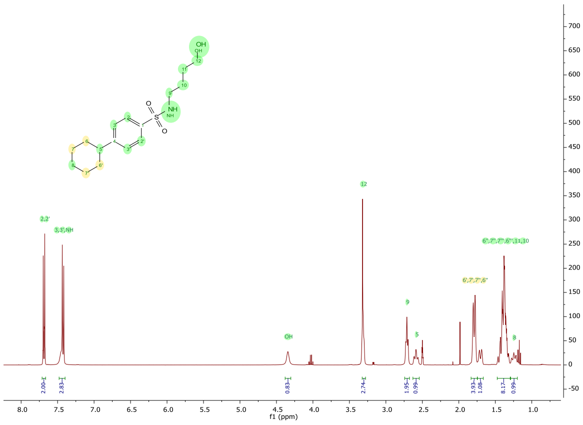

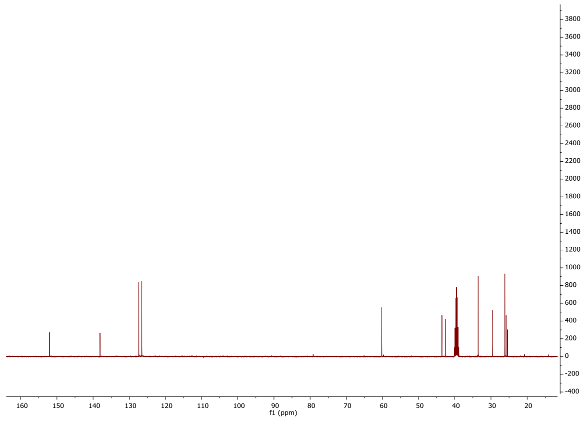

Compound 70b

^{1}H NMR (400 MHz, DMSO-d6); ^{13}C NMR (101 MHz, DMSO-d6)

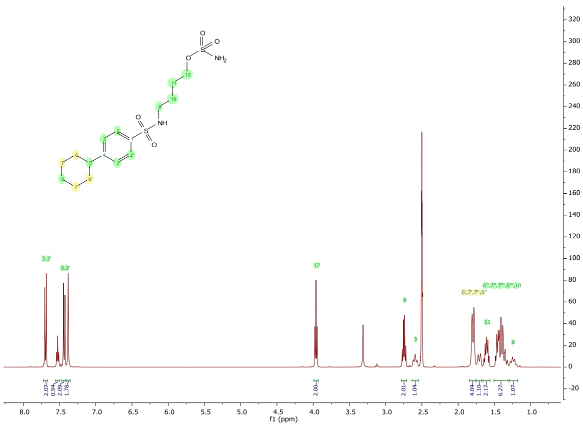

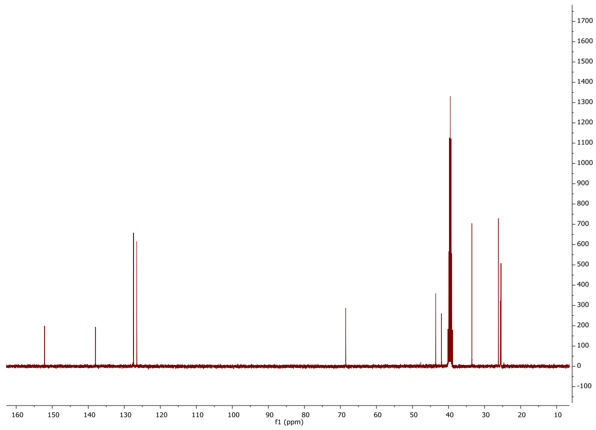

Compound 71a

^{1}H NMR (400 MHz, DMSO-d6); ^{13}C APT-NMR (126 MHz, DMSO-d6)

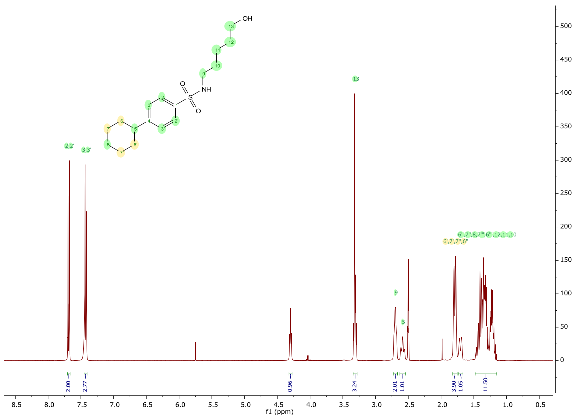

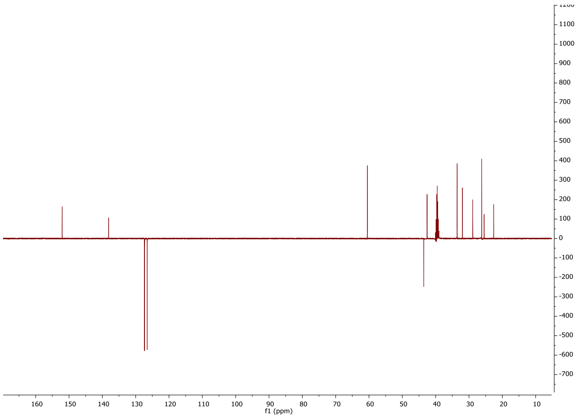

Compound 71b

^{1}H NMR (400 MHz, DMSO-d6); ^{13}C NMR (101 MHz, DMSO-d6)

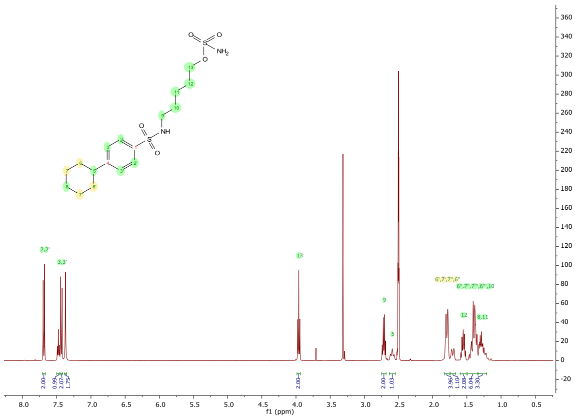

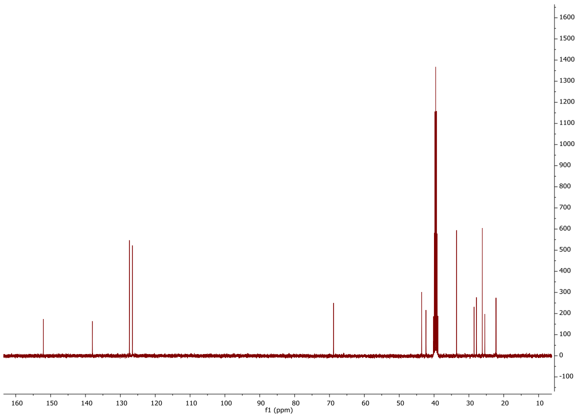

Compound 72a

^{1}H NMR (400 MHz, DMSO-d6); ^{13}C APT-NMR (101 MHz, DMSO-d6)

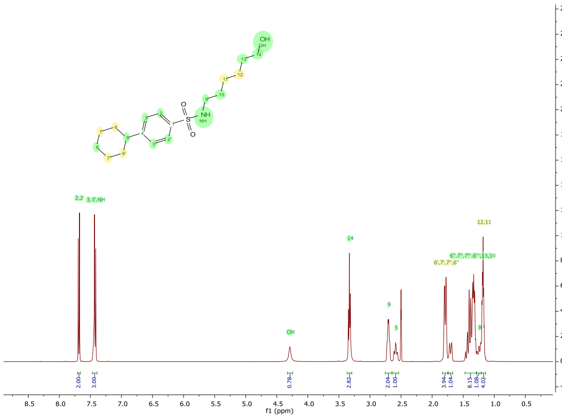

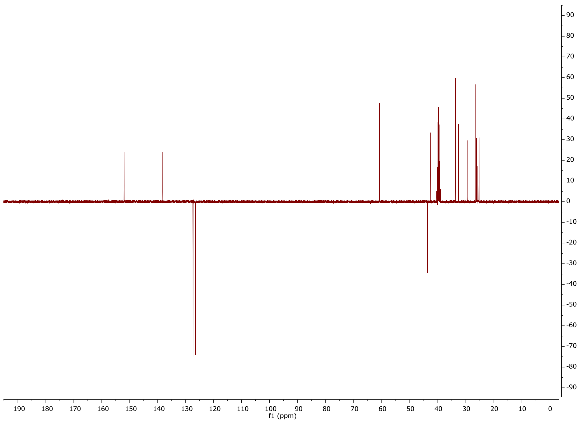

Compound 72b

^{1}H NMR (400 MHz, DMSO-d6); ^{13}C APT-NMR (101 MHz, DMSO-d6)

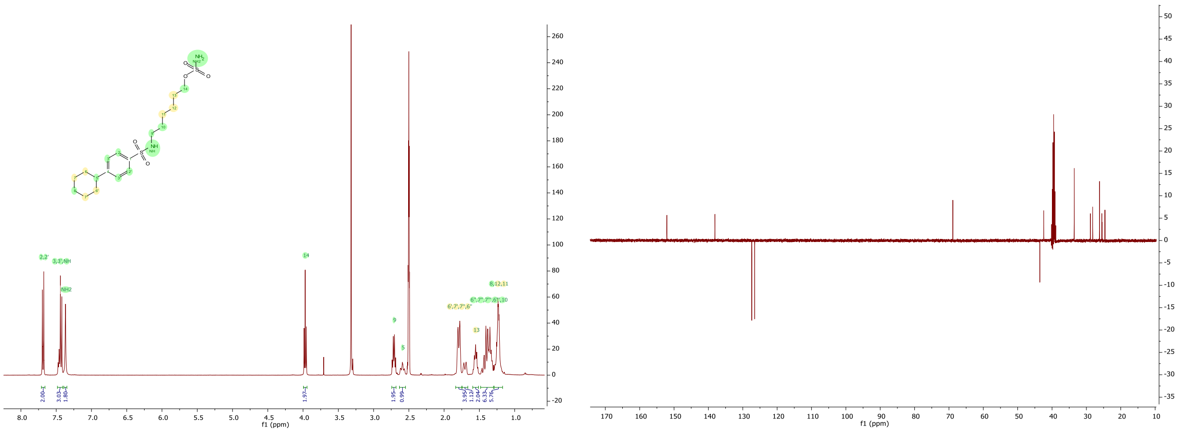

Compound 73a

^{1}H NMR (400 MHz, DMSO-d6); ^{13}C NMR (101 MHz, DMSO-d6)

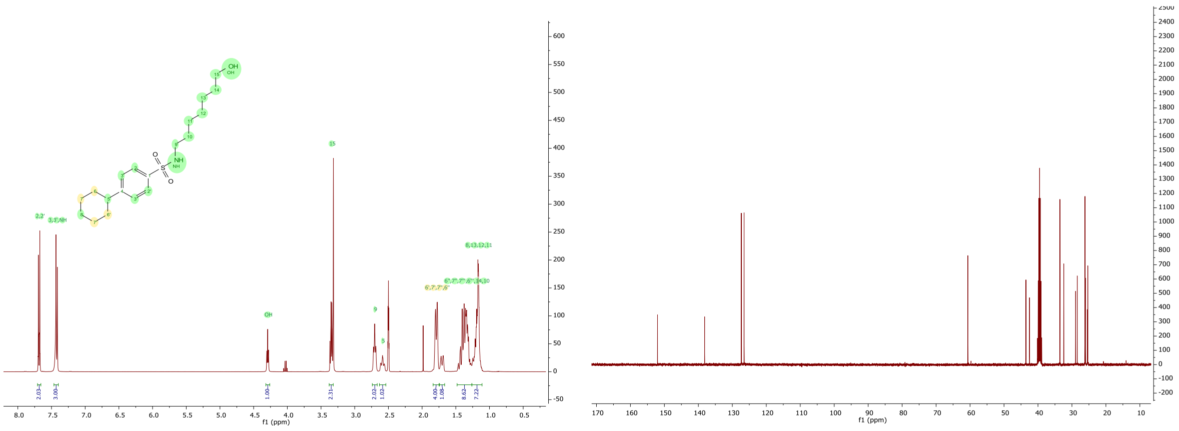

Compound 73b

^{1}H NMR (400 MHz, DMSO-d6); ^{13}C NMR (101 MHz, DMSO-d6)

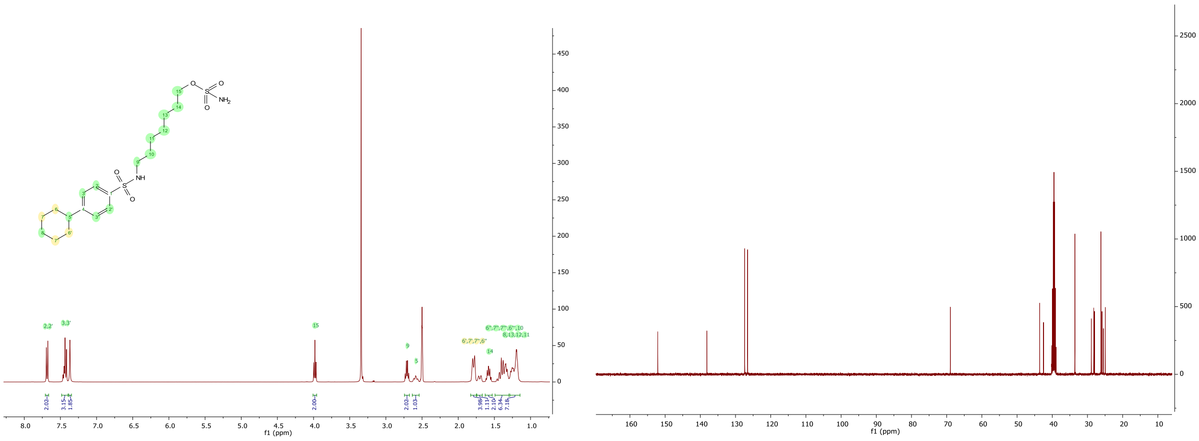

Compound 74a

^{1}H NMR (500 MHz, DMSO-d6); ^{13}C NMR (126 MHz, DMSO-d6)

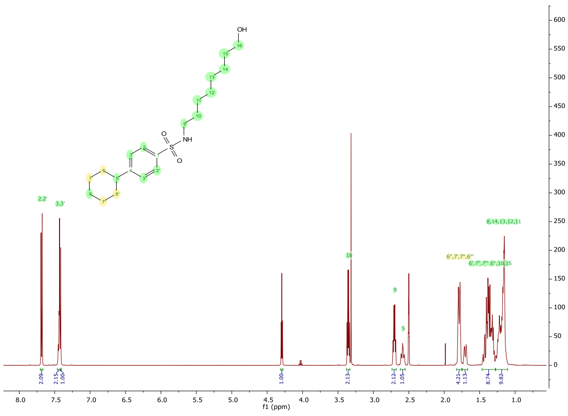

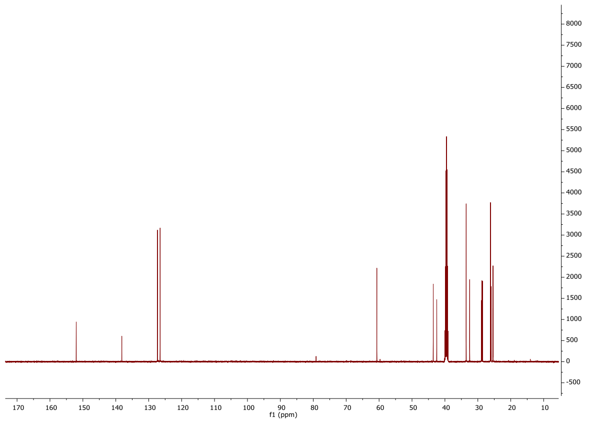

Compound 74b

^{1}H NMR (400 MHz, DMSO-d6); ^{13}C NMR (101 MHz, DMSO-d6)

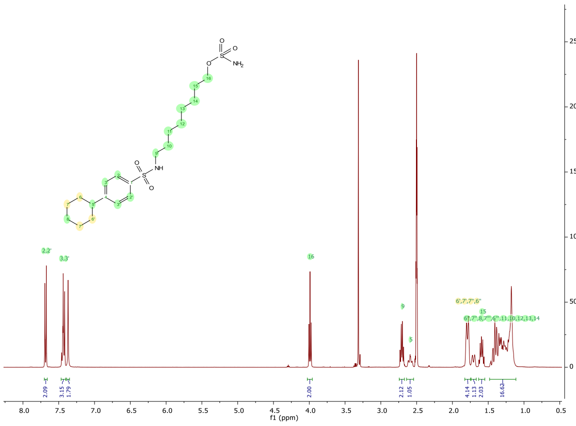

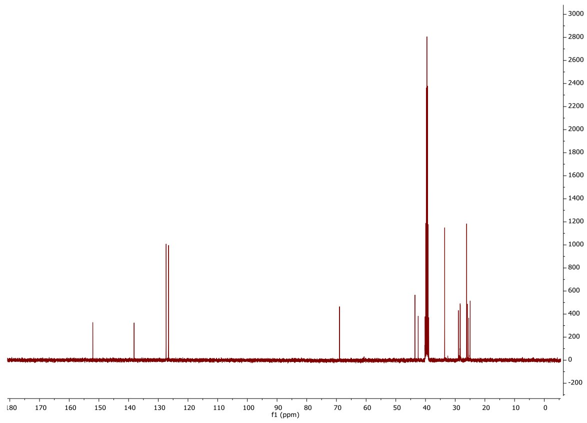

Compound 75a

^{1}H NMR (400 MHz, DMSO-d6); ^{13}C APT-NMR (101 MHz, DMSO-d6)

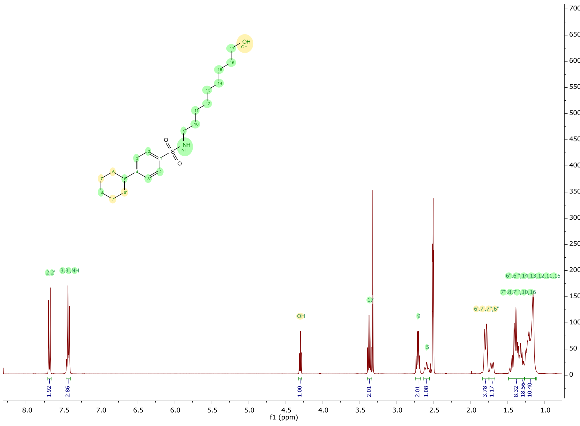

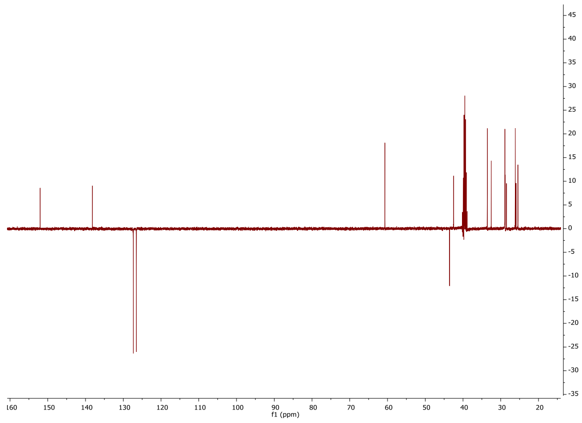

Compound 75b

^{1}H NMR (400 MHz, DMSO-d6); ^{13}C APT-NMR (101 MHz, DMSO-d6)

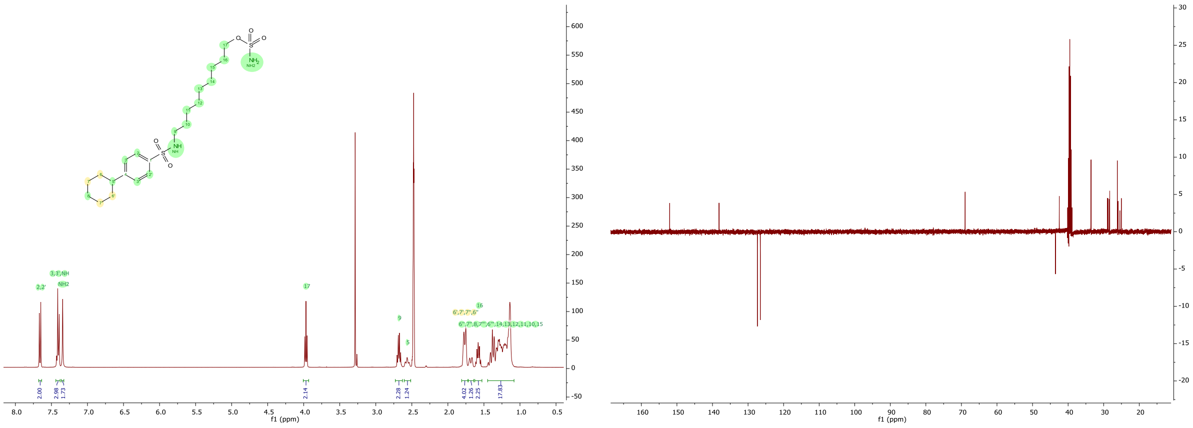

Compound 76a

^{1}H NMR (400 MHz, DMSO-d6); ^{13}C APT-NMR (101 MHz, DMSO-d6)

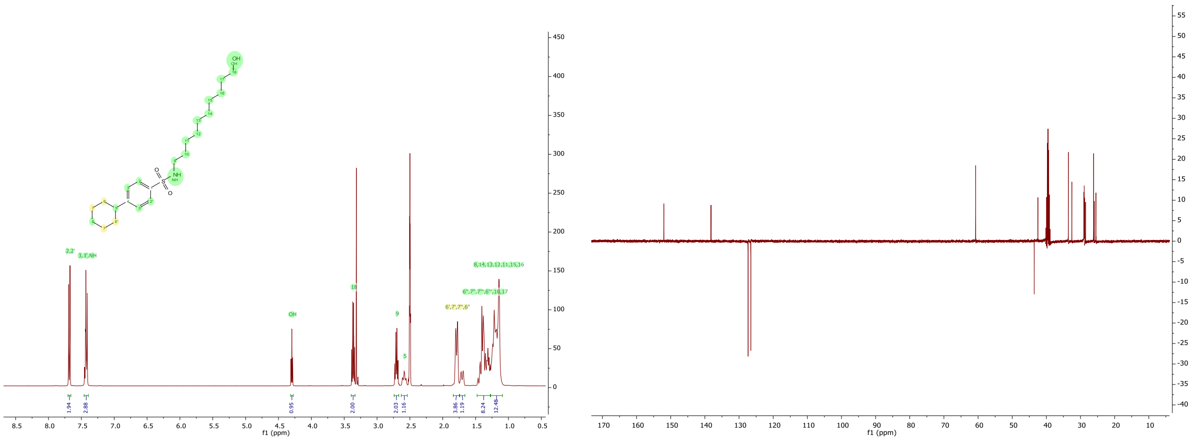

Compound 76b

^{1}H NMR (400 MHz, DMSO-d6); ^{13}C APT-NMR (101 MHz, DMSO-d6)

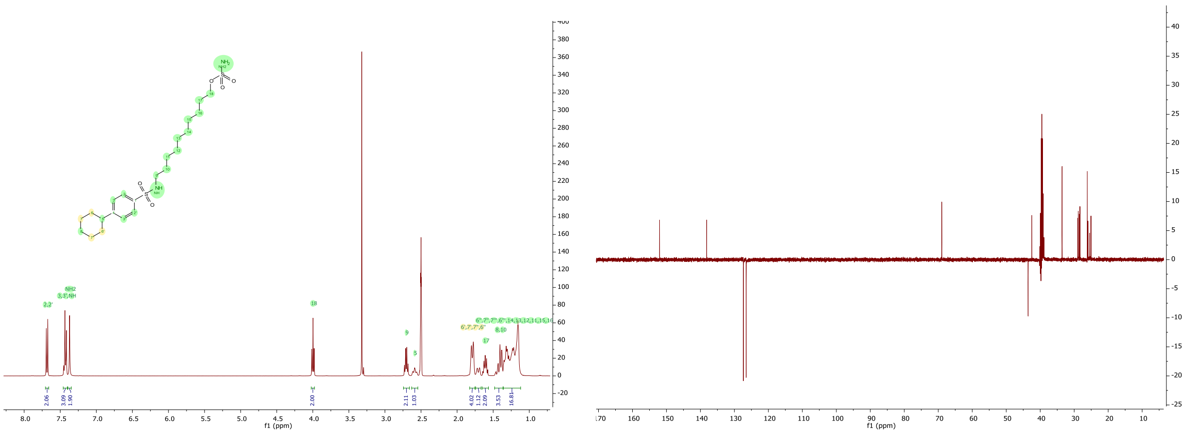

Compound 77a

^{1}H NMR (400 MHz, DMSO-d6); ^{13}C APT-NMR (101 MHz, DMSO-d6)

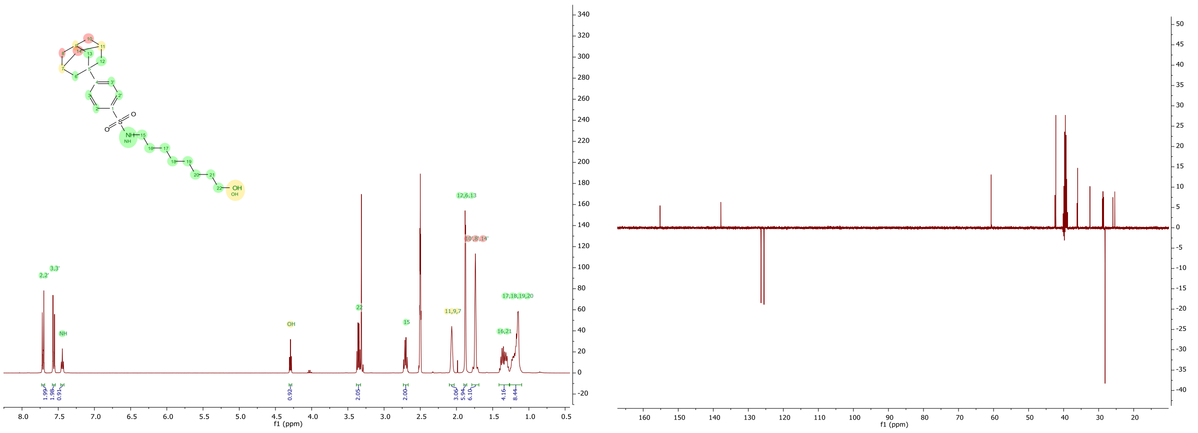

Compound 77b

^{1}H NMR (400 MHz, DMSO-d6); ^{13}C APT-NMR (101 MHz, DMSO-d6)

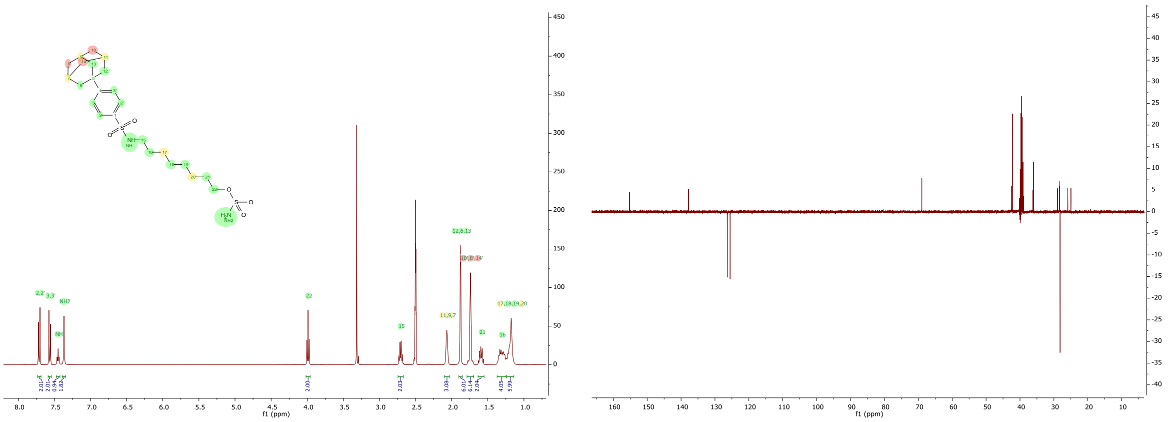

Compound 78a

^{1}H NMR (400 MHz, DMSO-d6); ^{13}C APT-NMR (101 MHz, DMSO-d6)

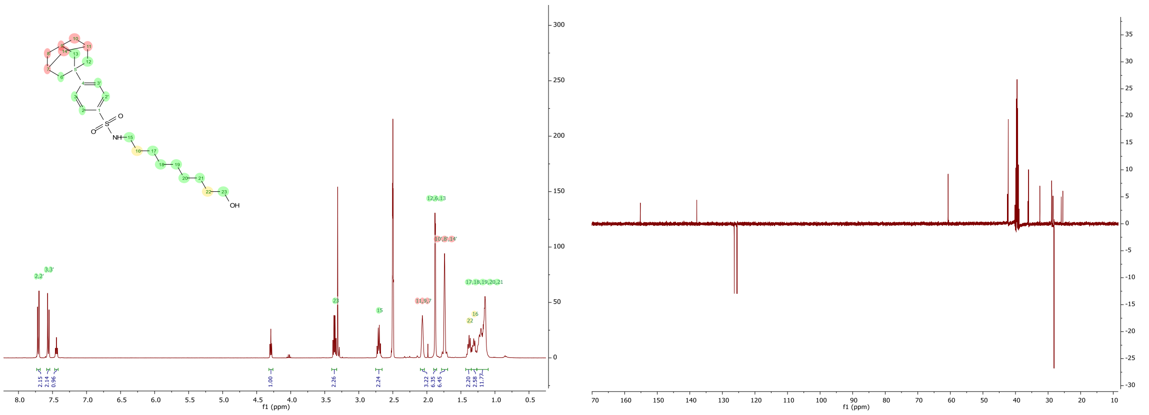

Compound 78b

^{1}H NMR (400 MHz, DMSO-d6); ^{13}C APT-NMR (101 MHz, DMSO-d6)

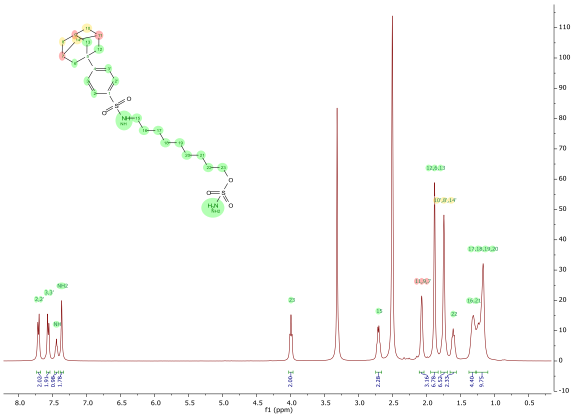

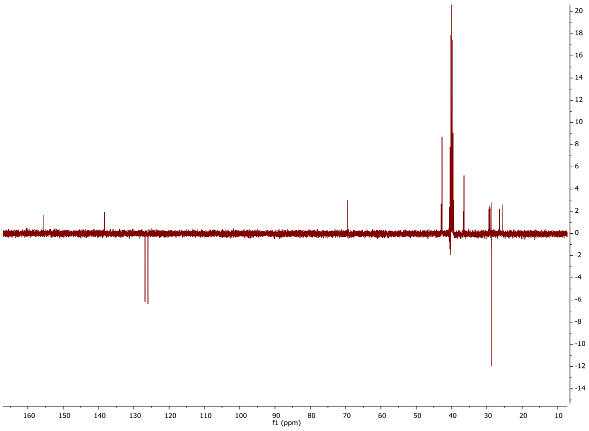

Compound 79a

^{1}H NMR (400 MHz, DMSO-d6); ^{13}C APT-NMR (101 MHz, DMSO-d6)

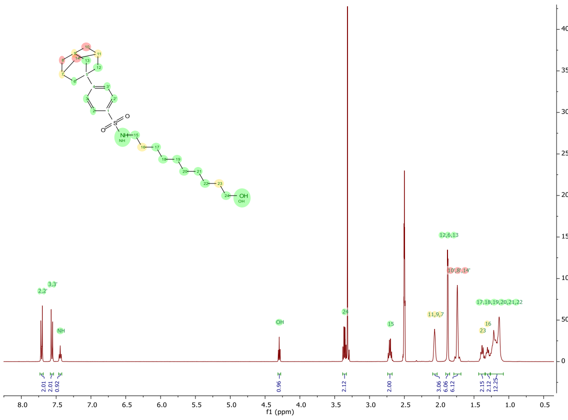

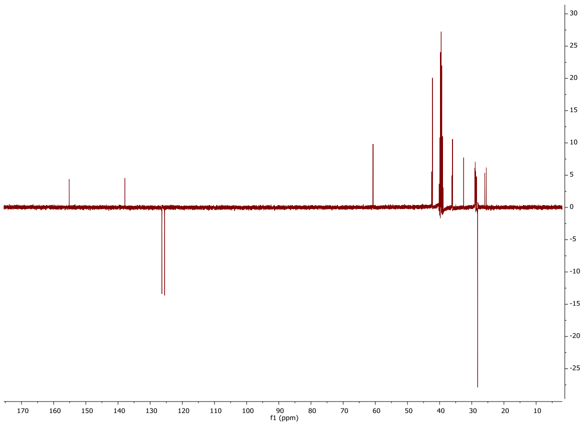

Compound 79b

^{1}H NMR (400 MHz, DMSO-d6); ^{13}C APT-NMR (101 MHz, DMSO-d6)

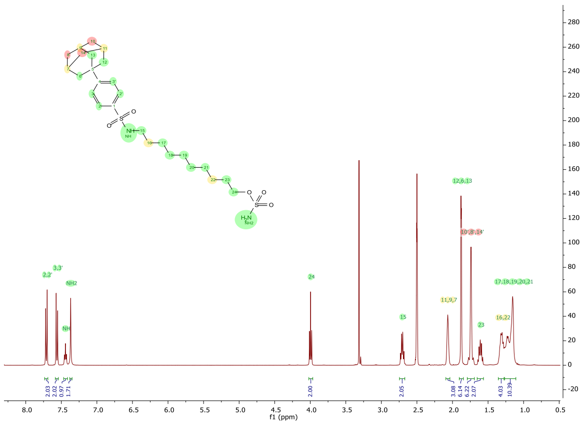

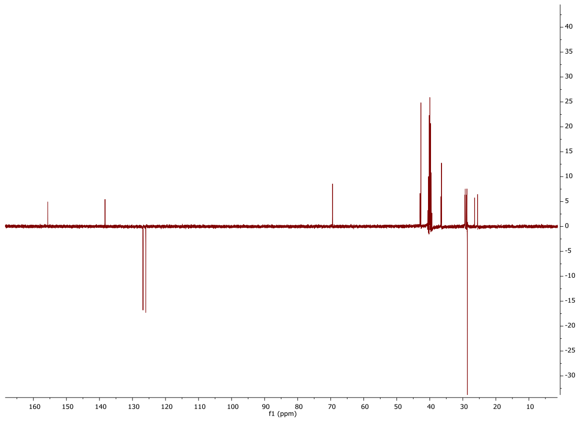

Representative HRMS spectra (ESI, MeOH)

Cmp **3a**

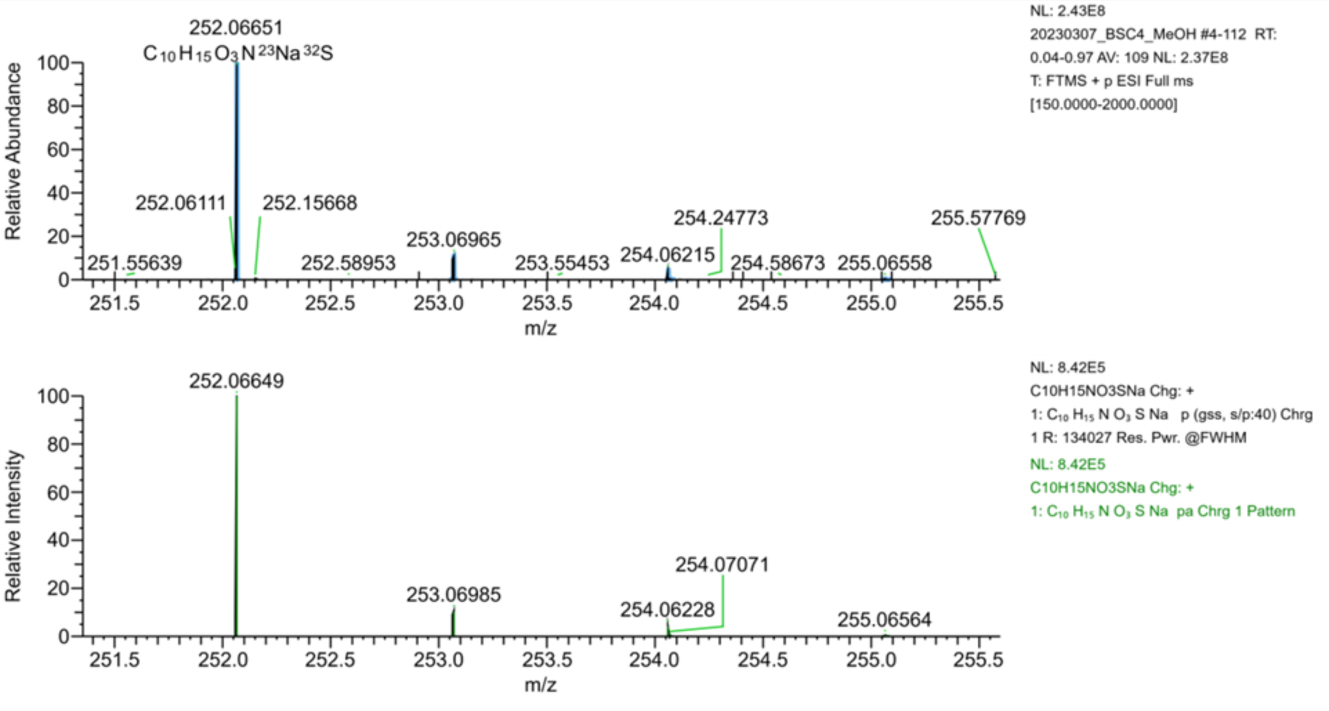

Cmp **16a**

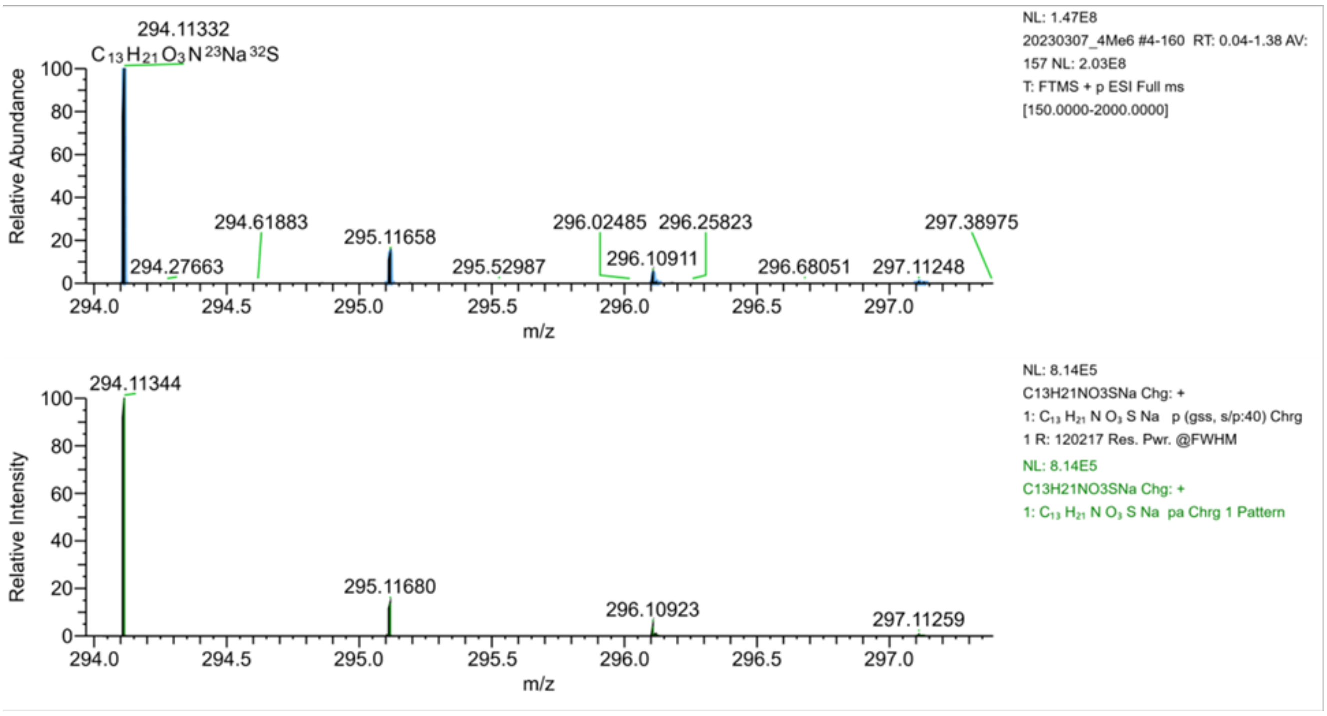

Cmp **49a**

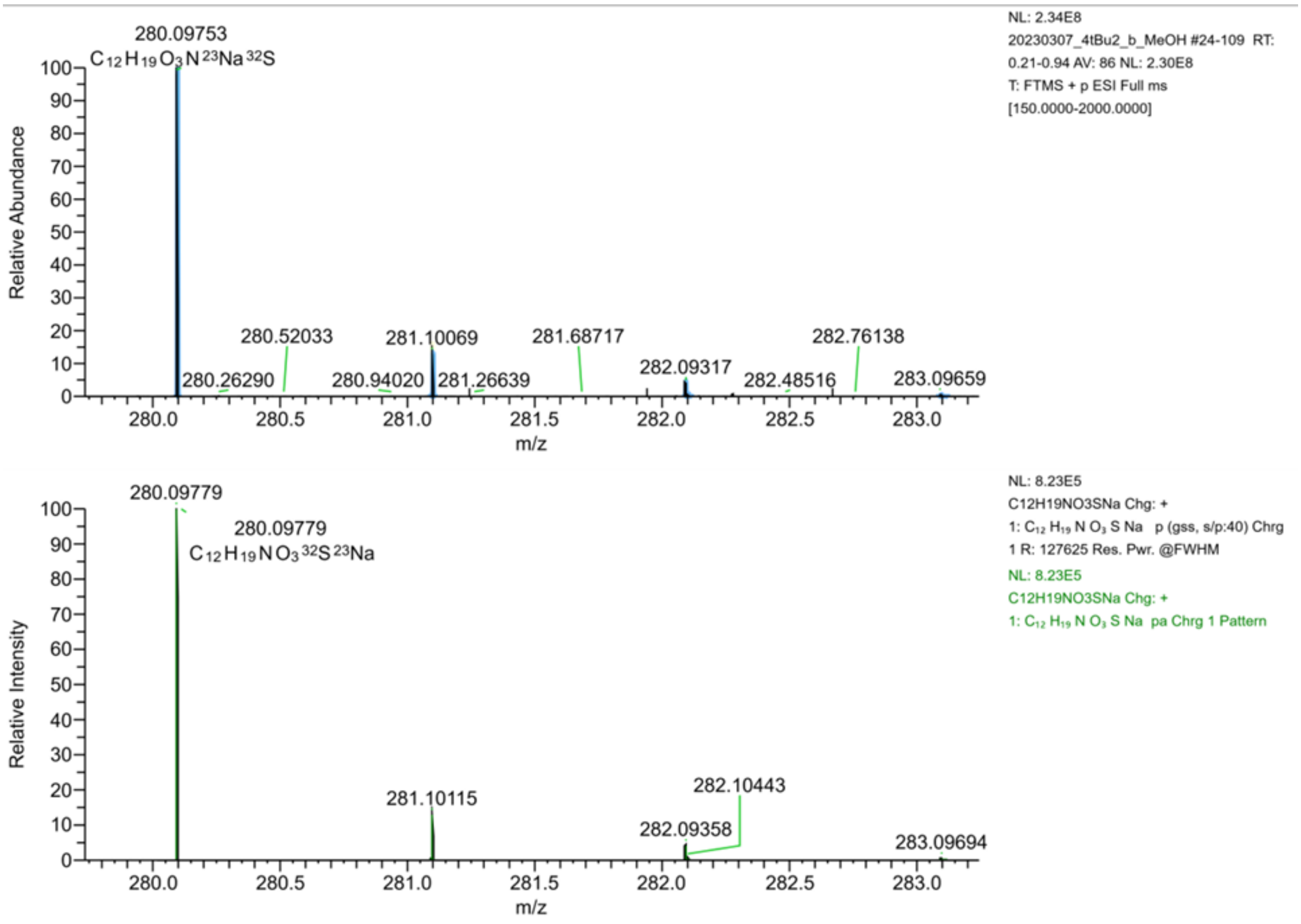

Cmp **50a**

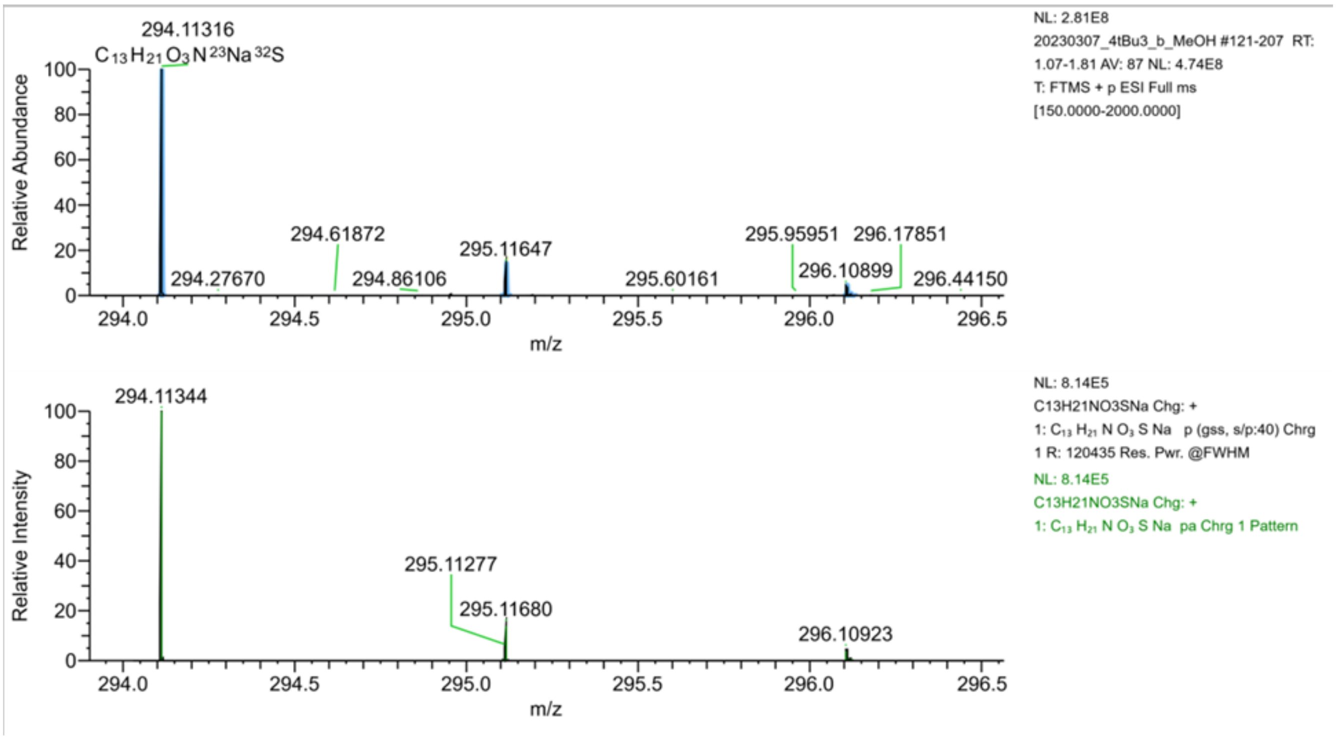

Cmp **51a**

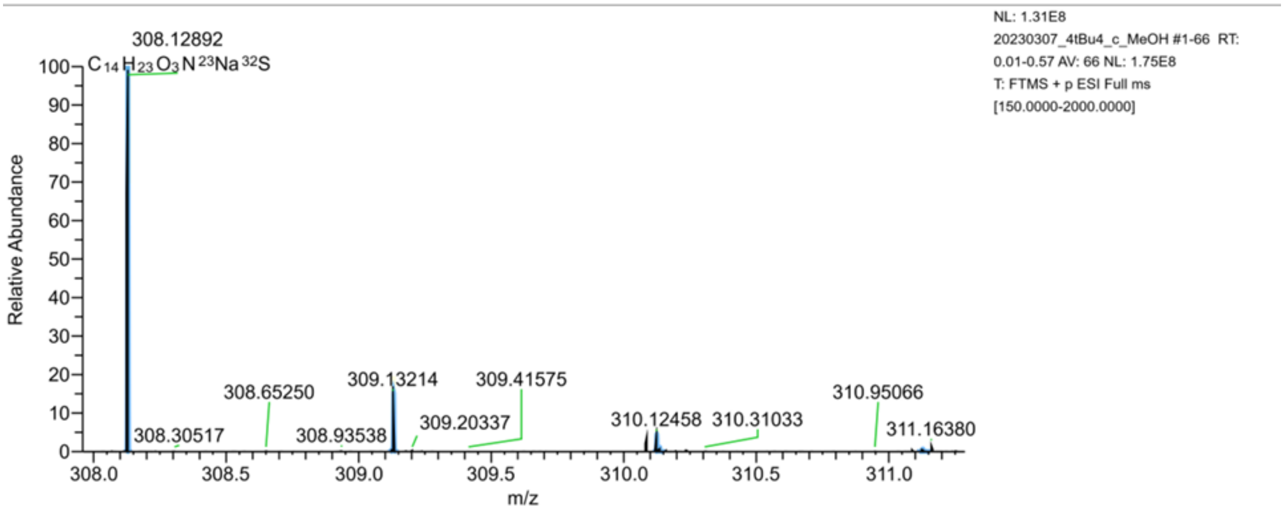

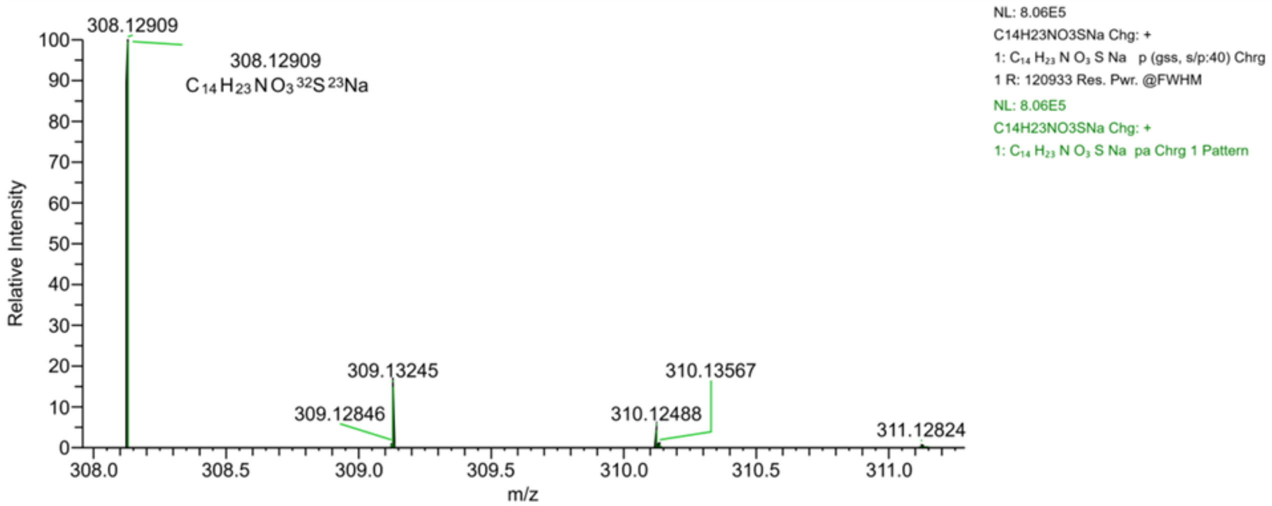

Cmp **52a**

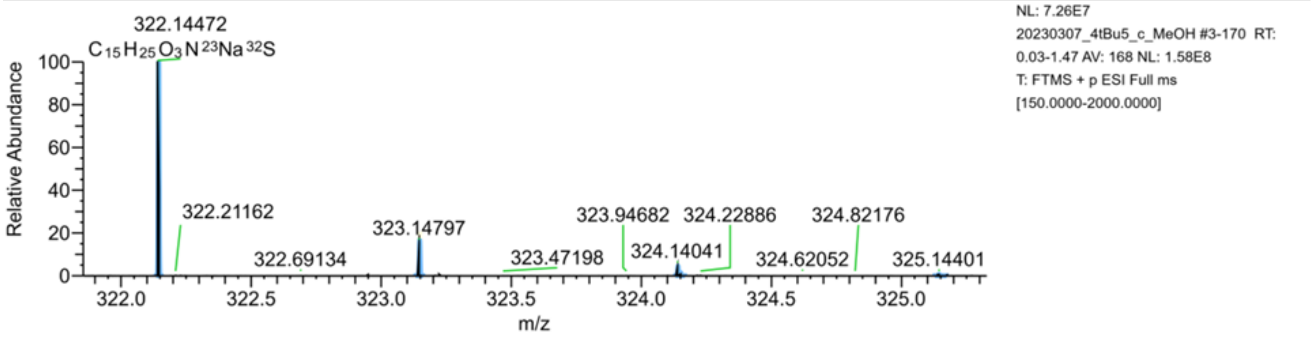

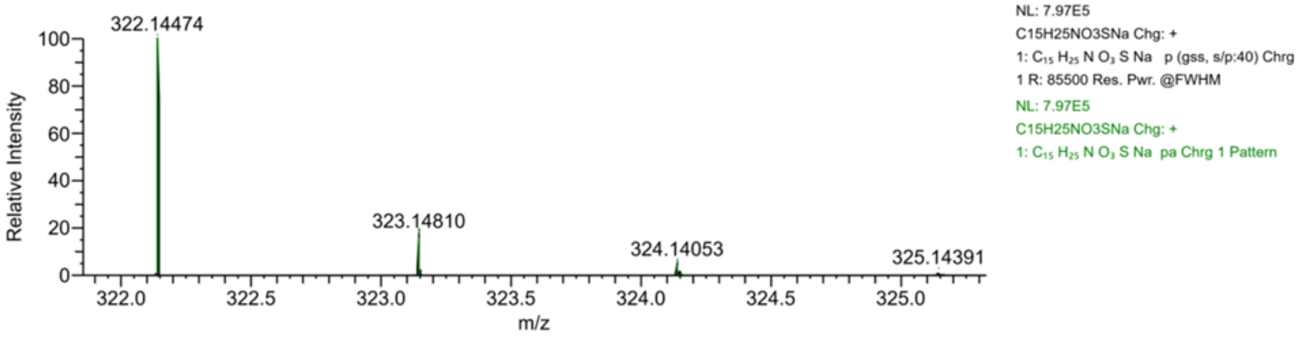

Cmp **58a**

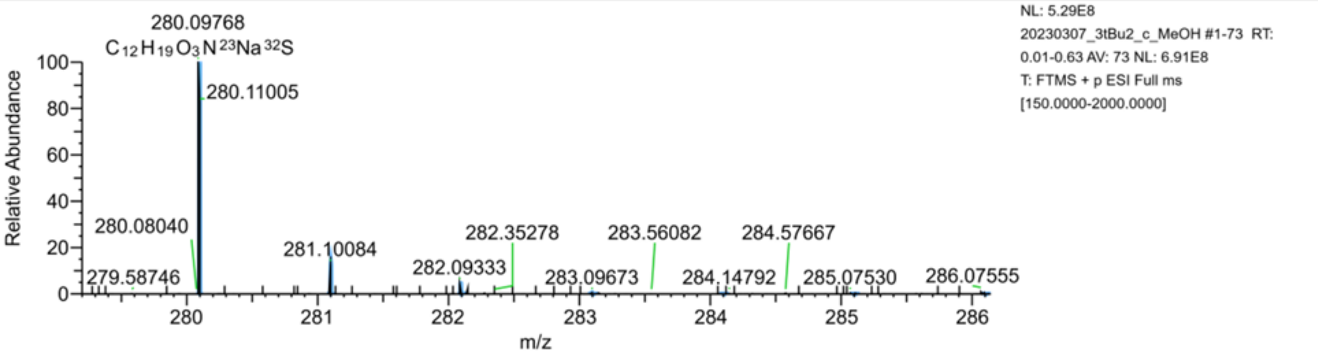

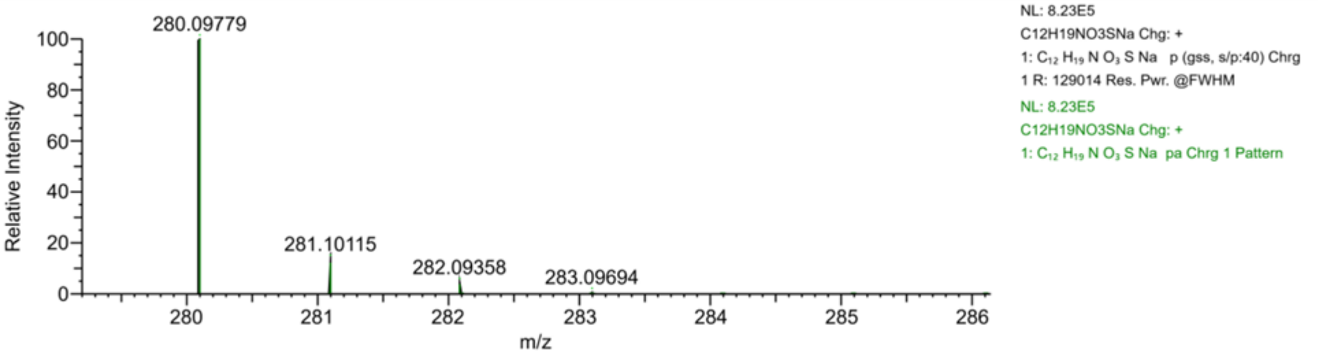

Cmp **59a**

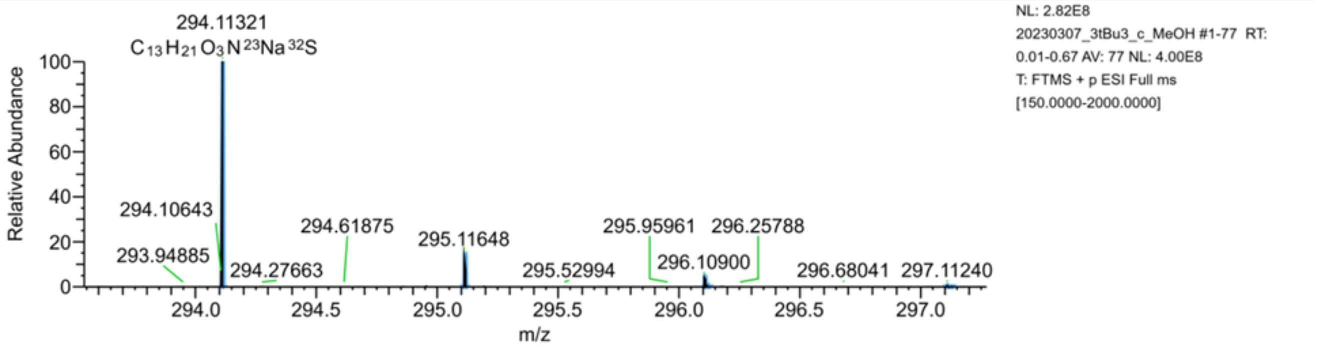

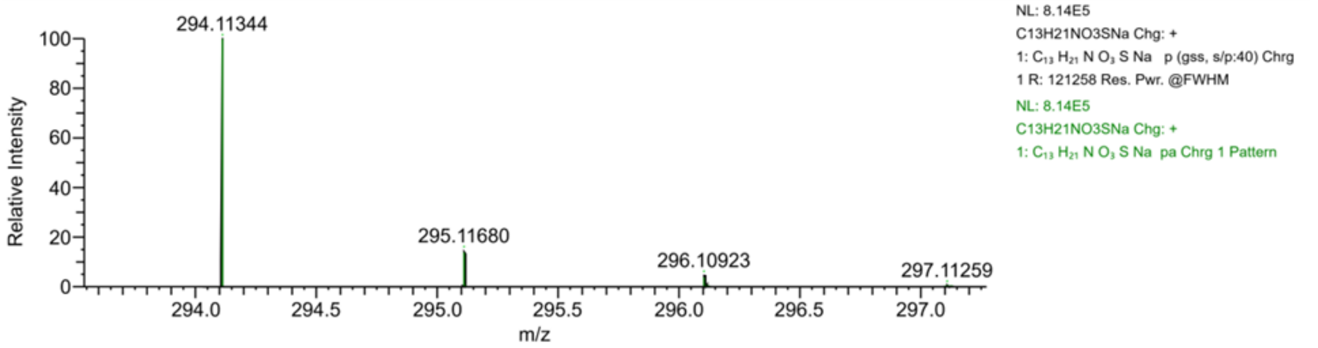

Cmp **60a**

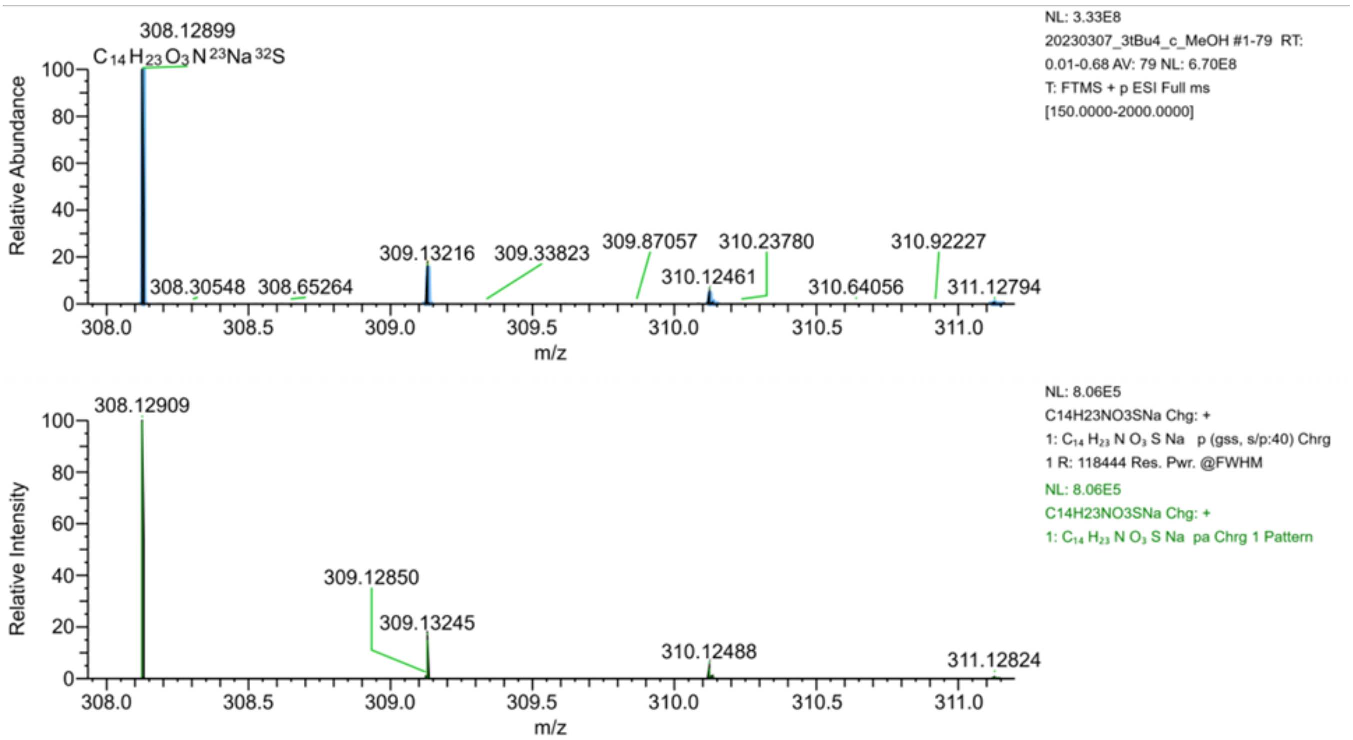

Cmp **61a**

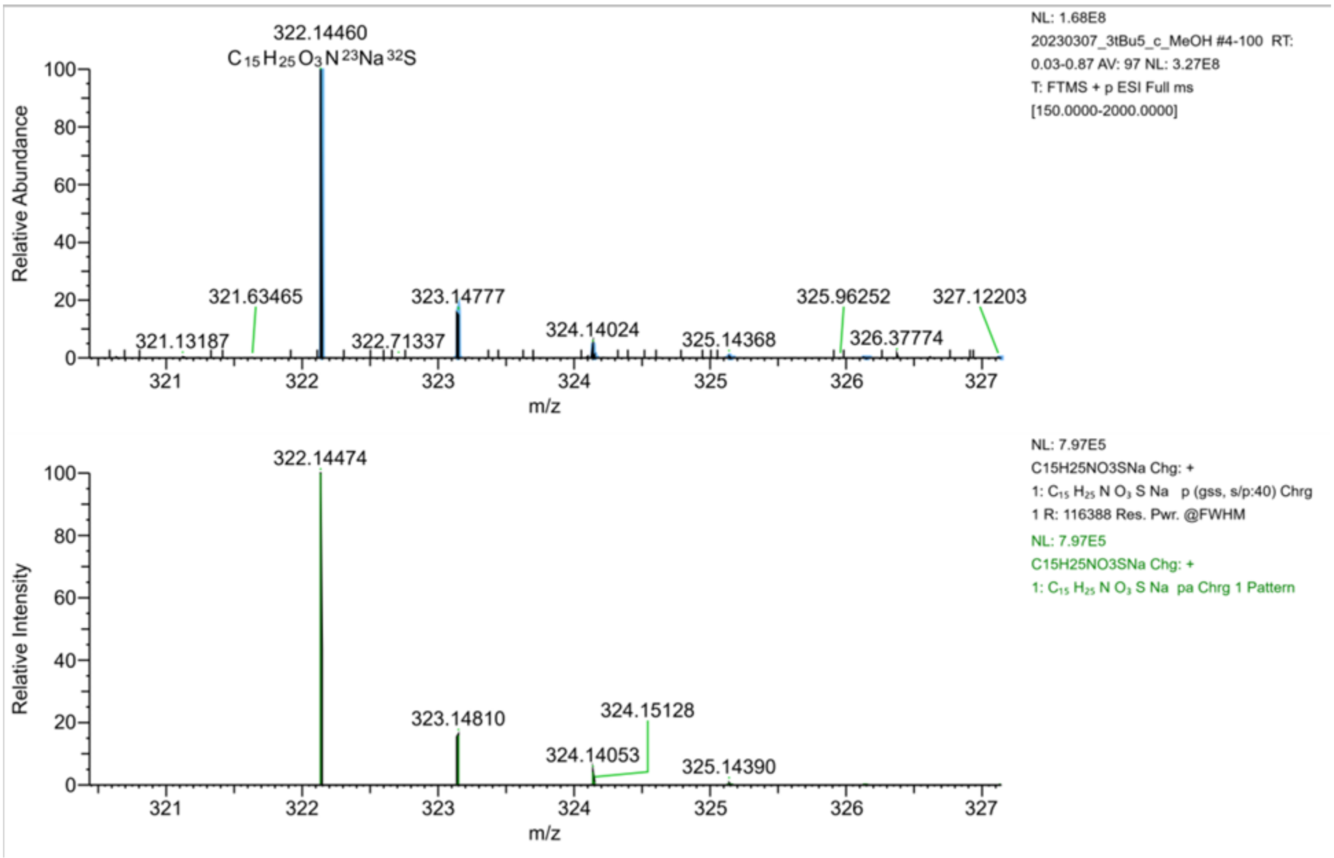

Depiction of the results (2D and 3D) from the docking calculations

For the most active compounds docking studies were performed.

12b

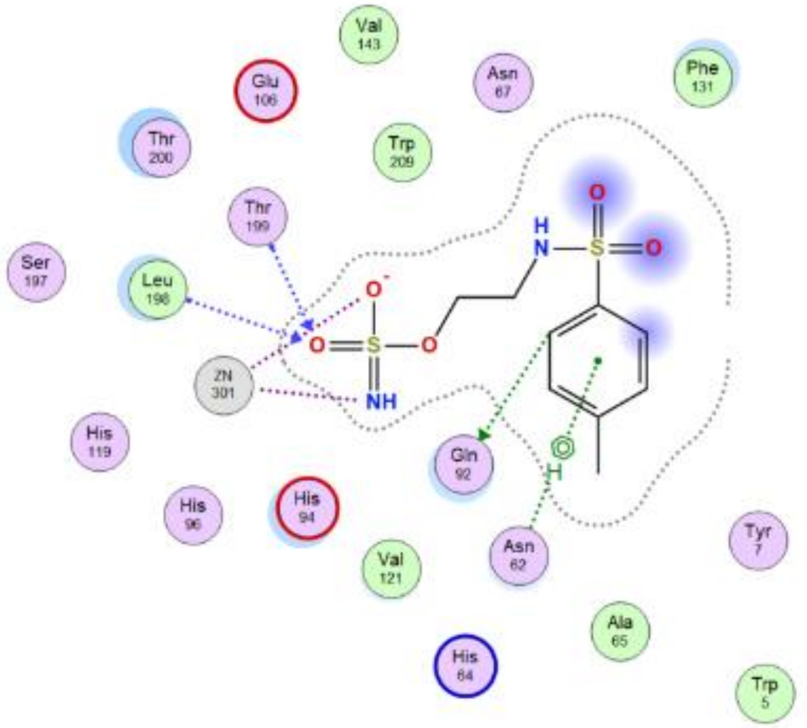

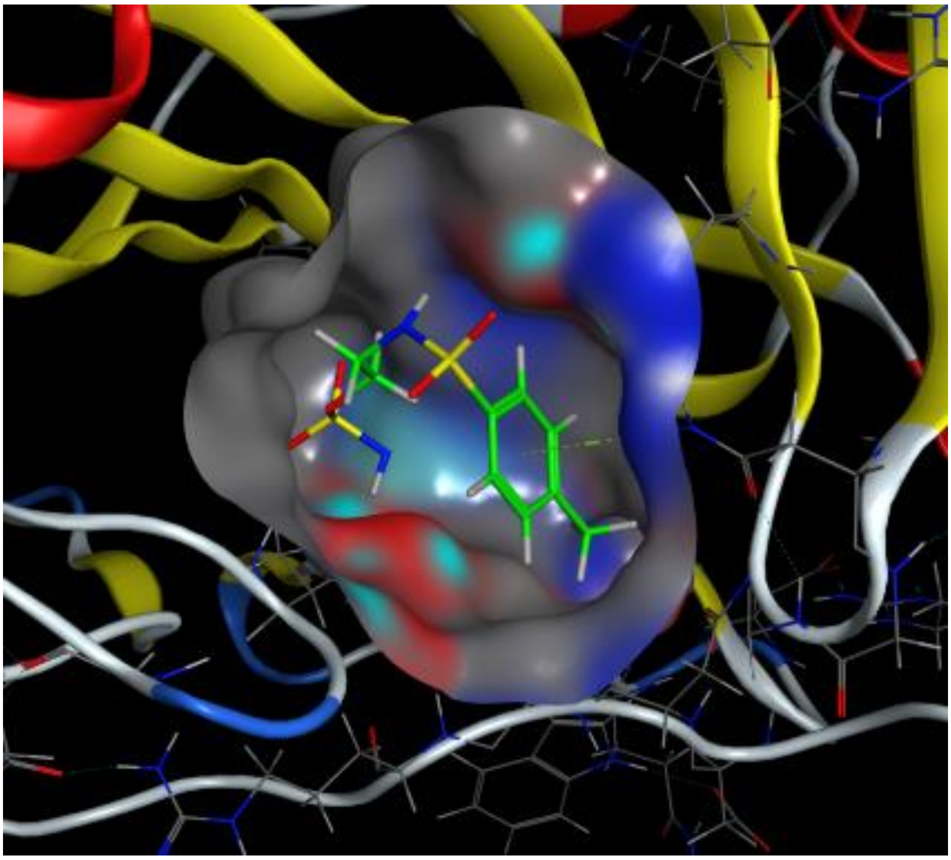

26b

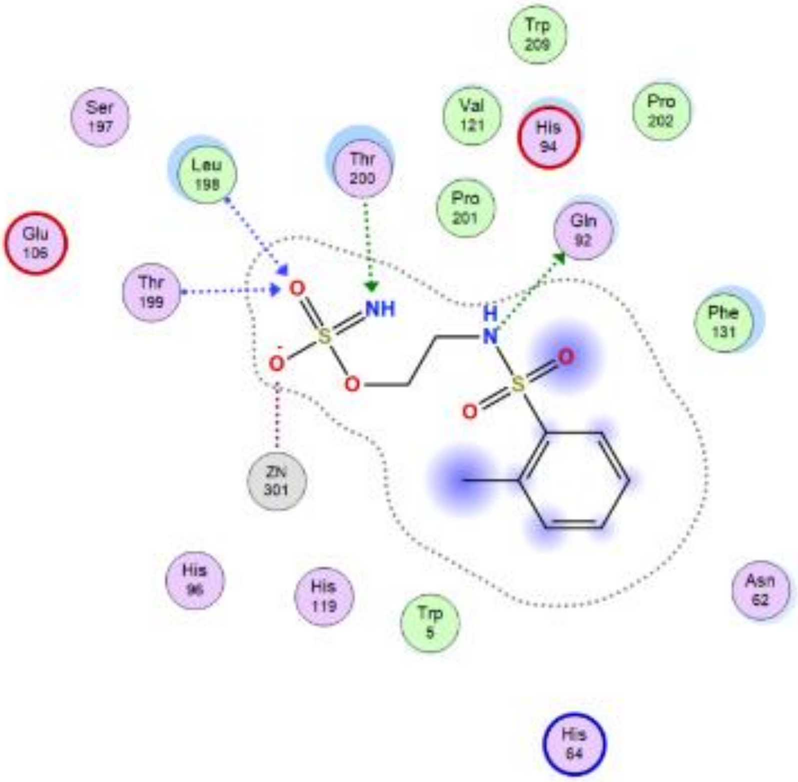
Trp 209
Ser 197
Val 121
His 94
Pro 202
Leu 198
Thr 200
Pro 201
Gln 92
Glu 106
Thr 199
Phe 131
O
NH
S
O
O
H
N
S
O
O
ZN 301
His 96
His 119
Trp 5
Asn 62
His 64

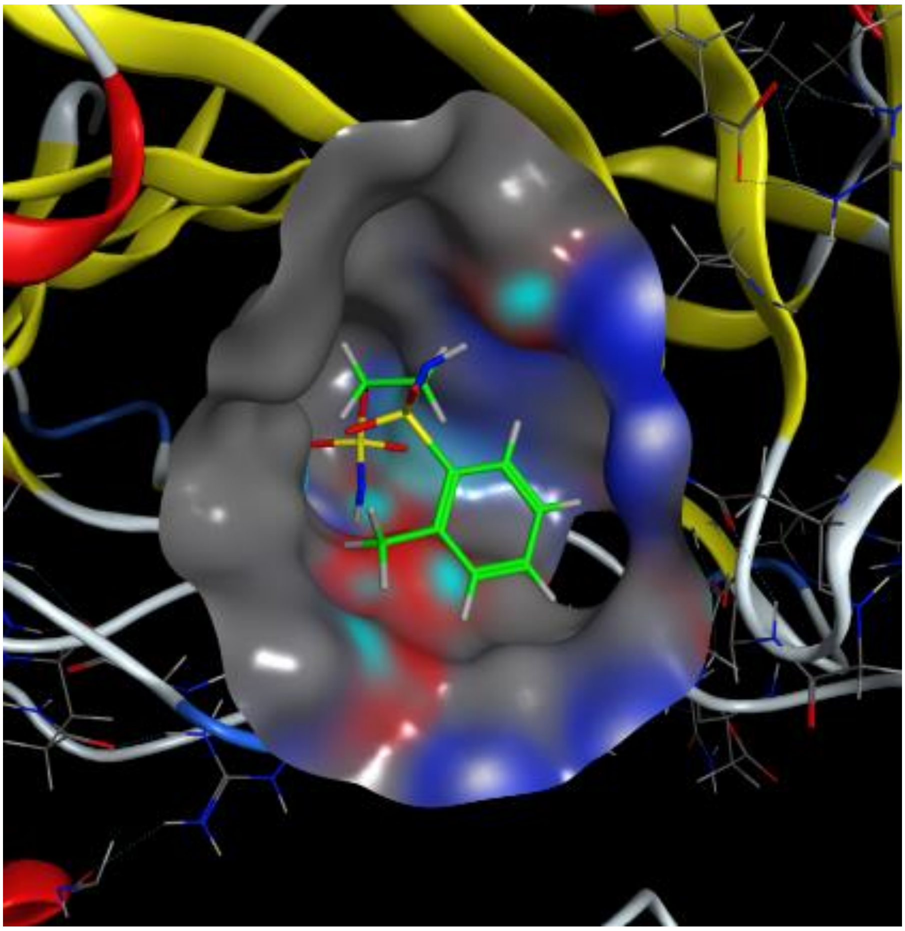

30b

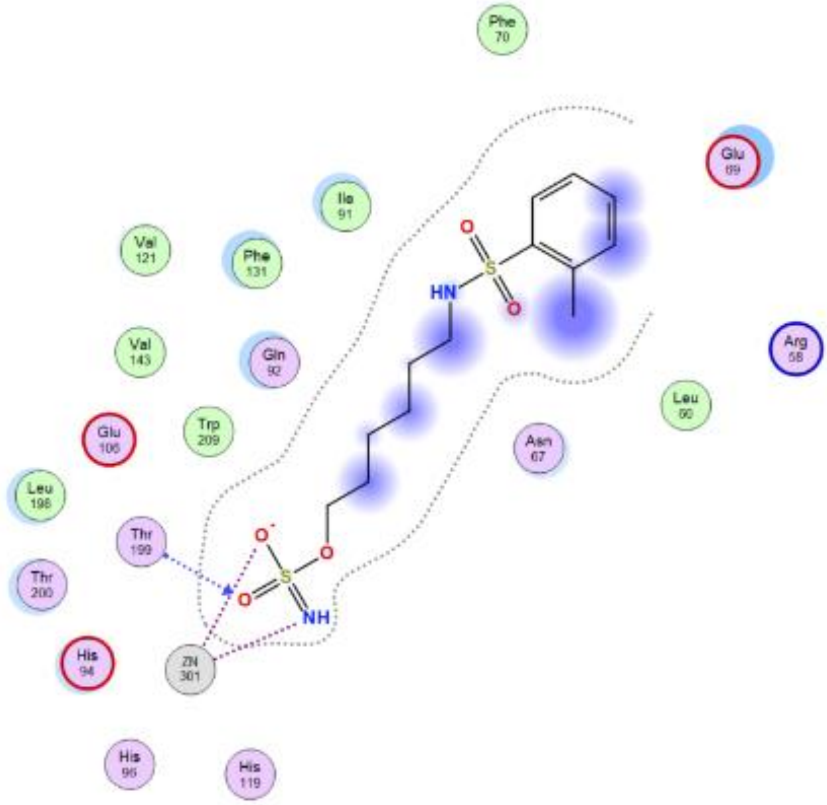
Phe 70
Glu 69
Ile 91
Val 121
Phe 131
Arg 58
Val 143
Gln 92
Leu 60
Trp 209
Glu 106
Asn 67
Leu 198
Thr 199
Thr 200
His 94
ZN 301
His 96
His 119
HN
NH
O
S

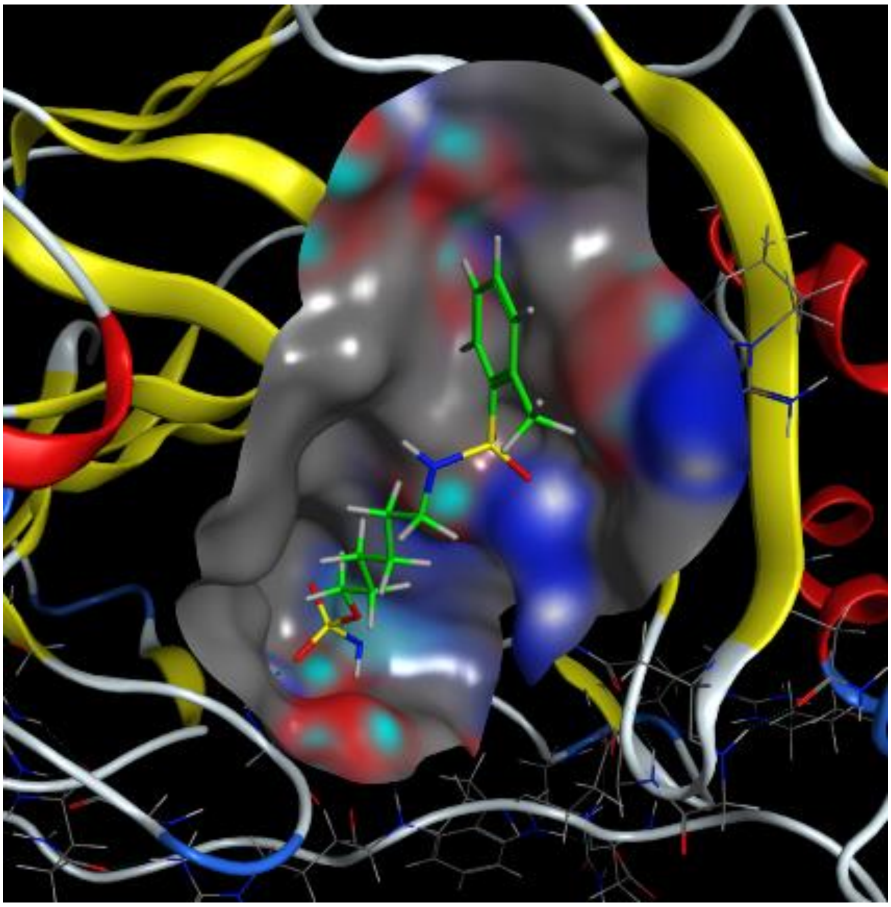

33b

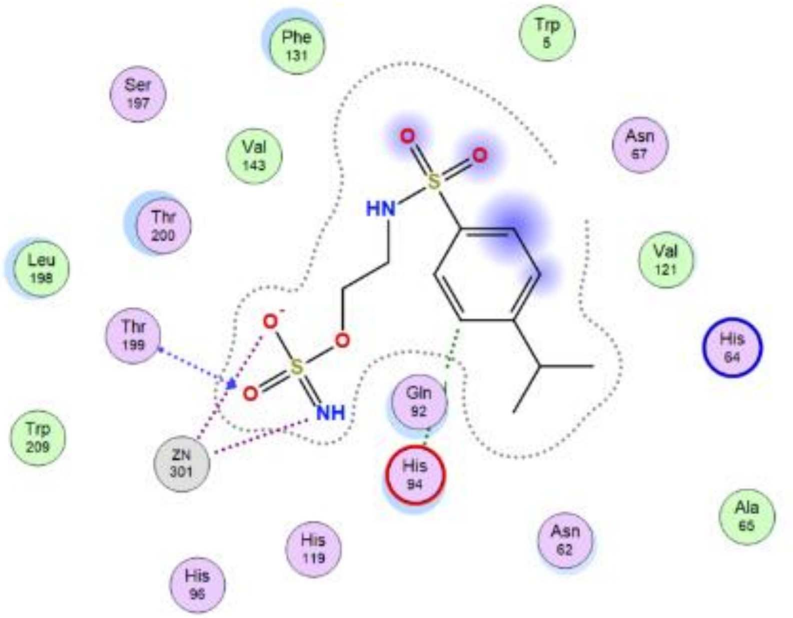

Phe 131
Trp 5
Ser 197
Val 143
Asn 67
Thr 200
HN
Leu 198
Val 121
Thr 199
His 64
Gln 92
NH
Trp 209
ZN 301
His 94
Ala 65
Asn 62
His 119
His 96

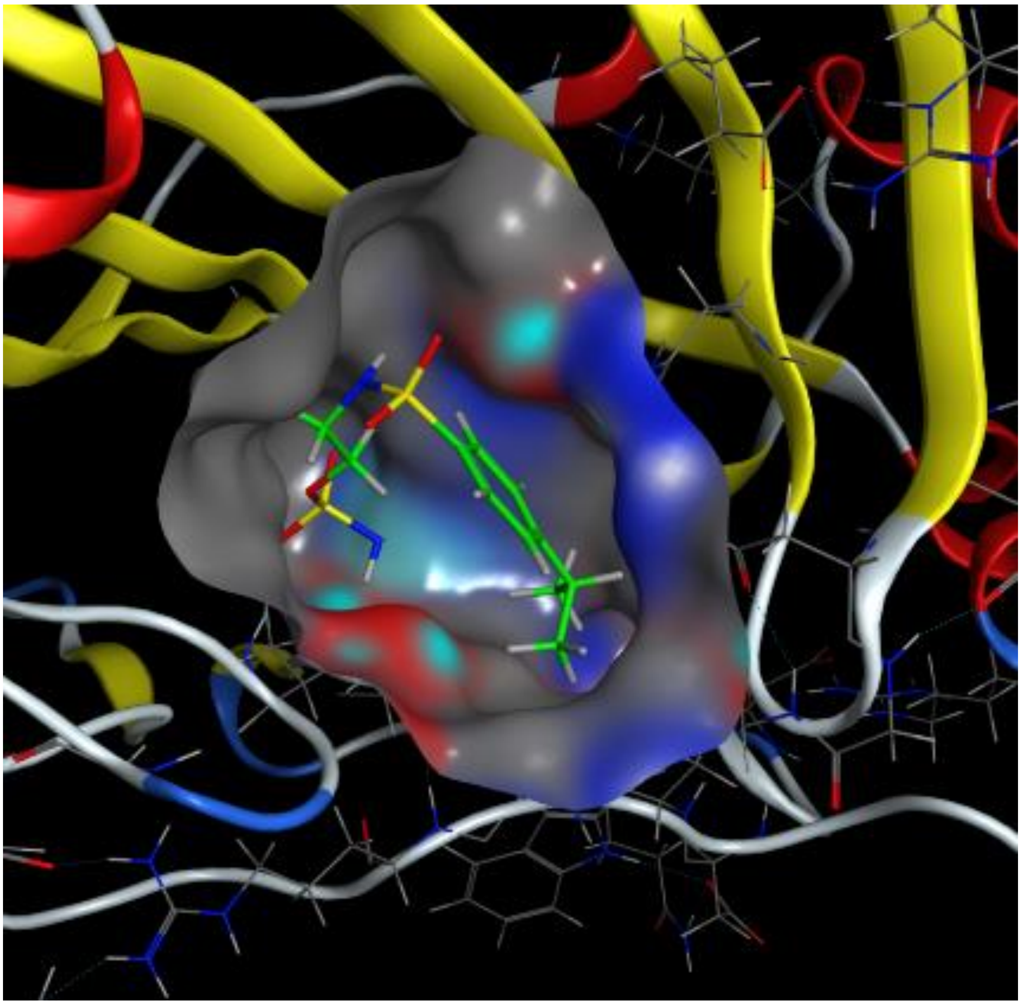

40b

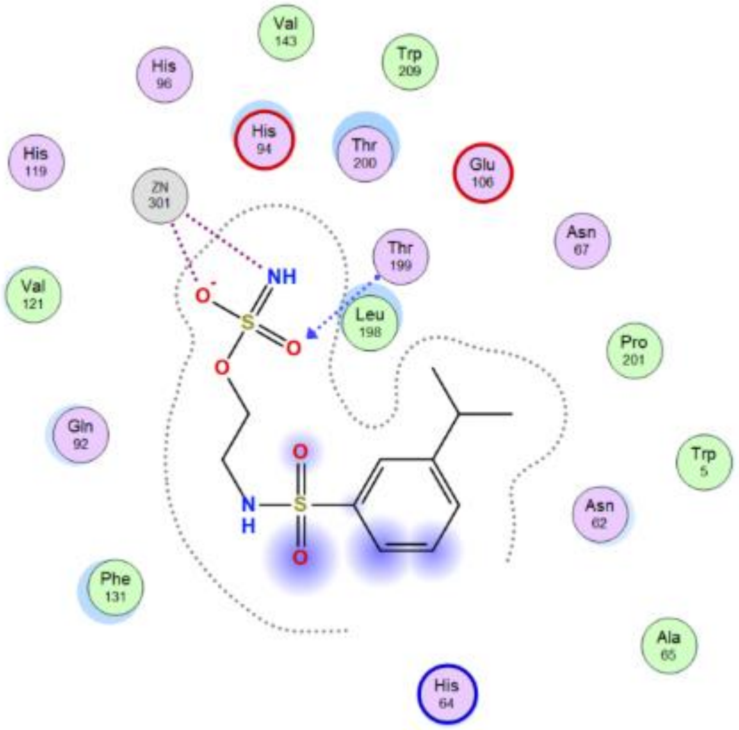
Val 143
His 96
Trp 209
His 94
Thr 200
His 119
Glu 106
ZN 301
Asn 67
Thr 199
NH
Val 121
O
S
O
Leu 198
Pro 201
O
Gln 92
O
Trp 5
N
S
H
O
Asn 62
Phe 131
Ala 65
His 64

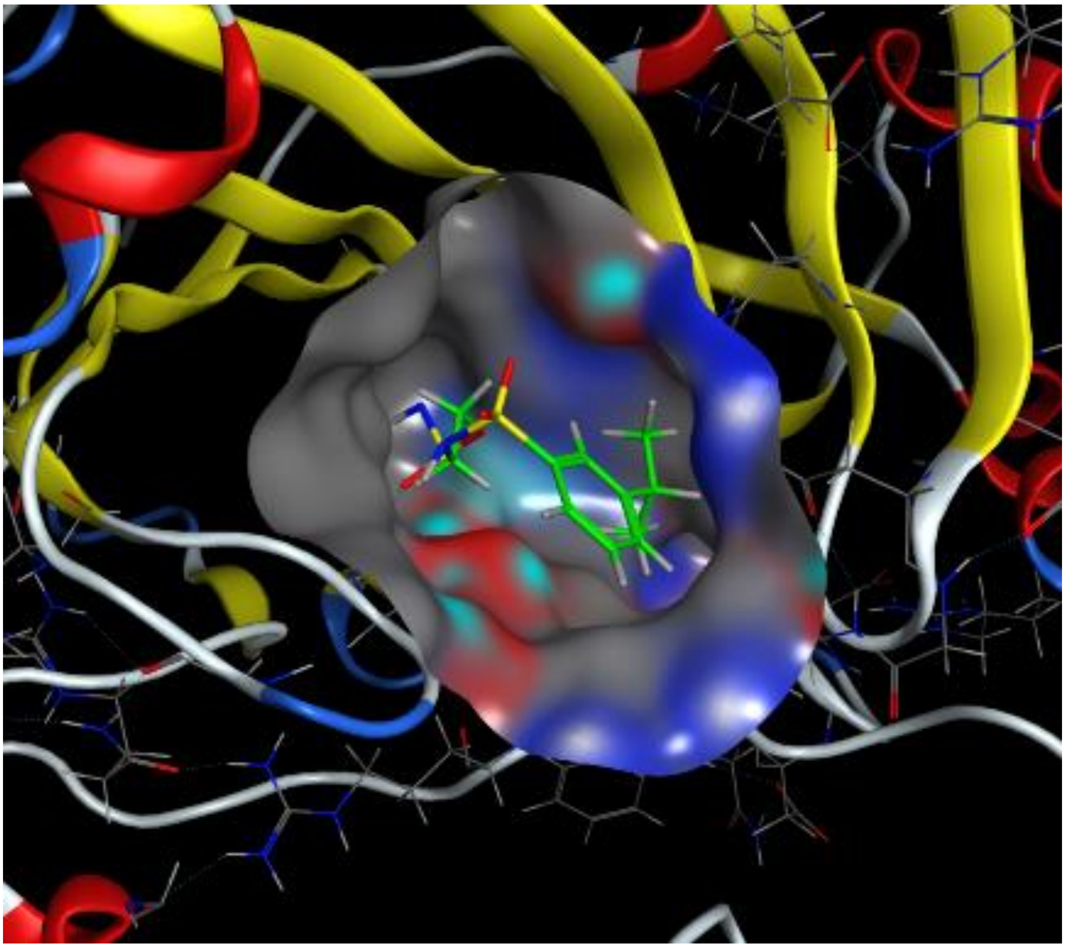

49b

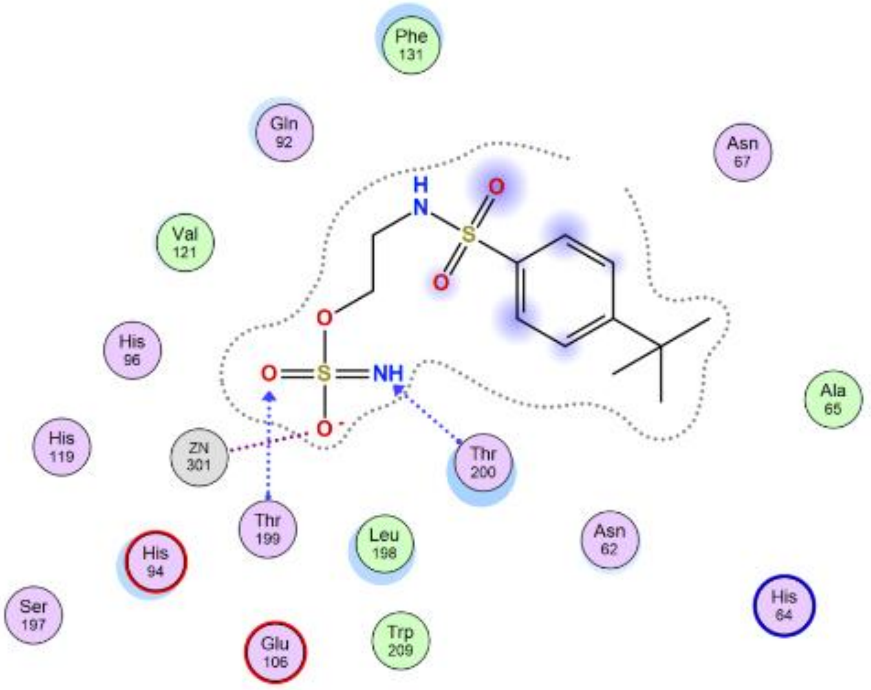
Phe
131
Gln
92
Asn
67
Val
121
H
N
O
S
O
O
His
96
O
S
NH
Ala
65
His
119
ZN
301
O-
Thr
200
Thr
199
Leu
198
Asn
62
His
94
His
64
Ser
197
Glu
106
Trp
209

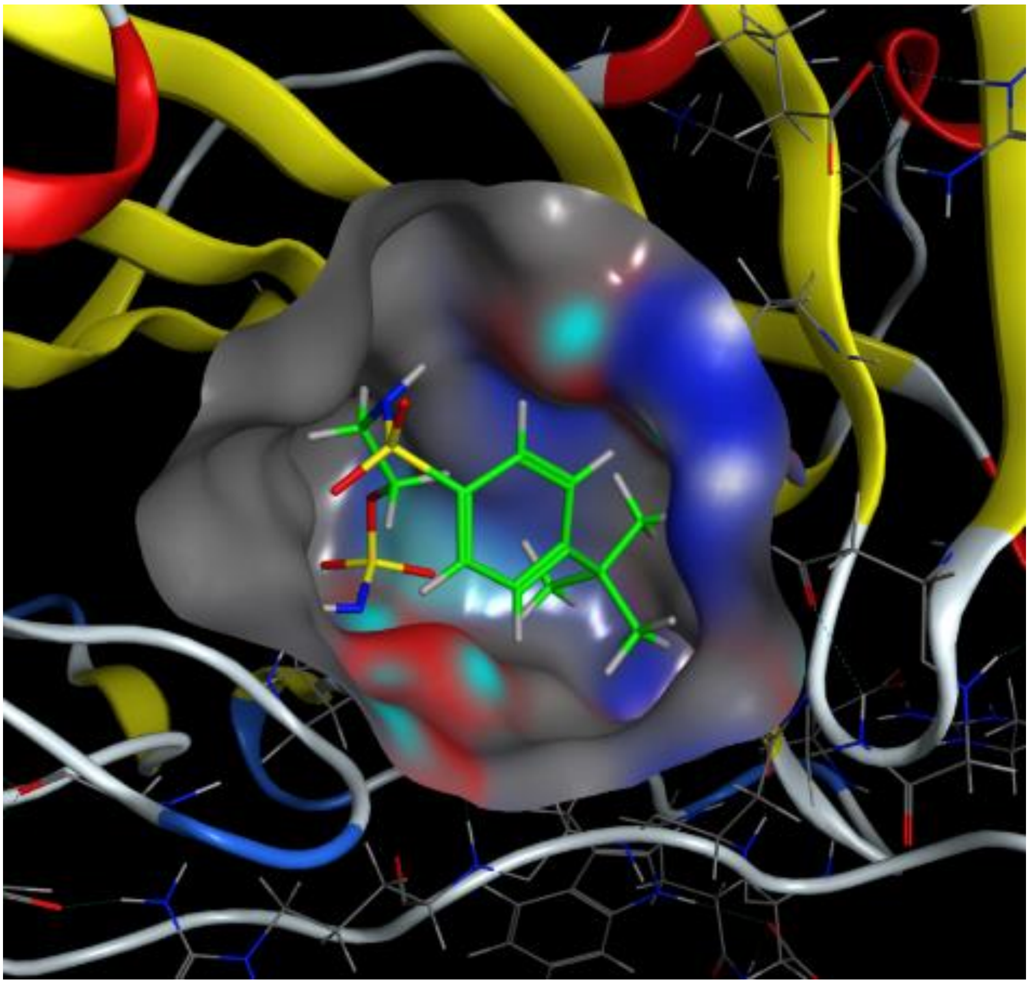

Supplement: Supplementary file 1 [file molecules-29-03015-s001.zip › molecules-3032581-supplementary.pdf]
